# Research on tissue-resident macrophages in the field of cancer research: a bibliometric analysis from 2004 to 2025

**DOI:** 10.1080/19336918.2026.2624204

**Published:** 2026-02-02

**Authors:** Qingya Song, Zongliang Yu, Wenping Lu, Qingyuan Chi

**Affiliations:** a Department of Oncology, Guang’anmen Hospital, China Academy of Chinese Medical Sciences, Beijing, China; b Graduate School, Beijing University of Chinese Medicine, Beijing, China

**Keywords:** Tissue resident macrophages, cancer, bibliometric analysis, citespace

## Abstract

In the tumor microenvironment, tissue-resident macrophages (TRMs) promote malignant tumor progression, yet their tissue-specific heterogeneity and complex functions bring research challenges. This study analyzes the research status and trends of TRMs in oncology. Via VOSviewer, CiteSpace, R software and WoSCC, a visual bibliometric network was built for quantitative analysis, with future research directions explored in depth. The US leads in publications and academic influence, and the University of Washington tops in paper output. Research focuses on TRMs’ origin, classification and tumor microenvironment functions; microglia and Kupffer cells are the most studied subsets. Current research centers on pathway exploration, immunotherapy and single-cell sequencing. This study summarizes TRMs’ research status, hotspots and trends in oncology, providing valuable insights for relevant collaborators and institutions.

## Introduction

Cancer is a major global public health problem, which seriously endangers people’s lives and health and lowers their quality of life. The report of the International Agency for Research on Cancer, which belongs to the World Health Organization, showed that in 2022, there were about 20 million new cancer cases and around 9.7 million cancer deaths around the world [1]. The treatment of malignant tumors remains a current hot topic and a difficult challenge. Tumor-associated macrophages (TAMs) are part of the tumor microenvironment and play a crucial role in tumor progression [2]. According to their different origins, tumor-associated macrophages can be classified into embryo-derived tissue-resident macrophages (TRMs) and monocyte-derived macrophages. TRMs are derived from yolk sac progenitor cells. In the course of embryogenesis, they undergo differentiation into diverse macrophage subtypes either directly or through fetal liver monocyte intermediates. They migrate to various tissues and settle down during embryonic development. After birth, these cells are capable of self-renewal and maintenance within local tissues, showing minimal dependence on the supplementation from bone marrow-derived hematopoietic stem cells and circulating monocytes [3–10]. TRMs primarily function to initiate immune responses and sustain tissue-specific homeostasis. They are capable of secreting multiple cytokines and chemokines, thereby regulating inflammatory reactions and orchestrating the recruitment and activation of immune cells. TRMs play a pivotal role in the immune defense, immune surveillance, and immune self-stability mechanisms of the organism [11].

In the tumor microenvironment, TRMs play a driving role in the progression of malignant tumors and thus represent ideal targets for the development of cancer-targeted therapies [12–14]. However, the phenotype and function specificities exhibited by TRMs within diverse tissues and organs constitute a notable research challenge. With the advancement of technologies like single-cell RNA sequencing and cell fate mapping, research on TRMs’ classification, functions, and their roles in malignant tumorigenesis has been rapidly updated [15,16]. Therefore, it is necessary to conduct a systematic compilation of the publications concerning TRMs in the area of malignant tumors. The aim of this paper is to construct a global knowledge map of TRM publications in the malignant tumor field, visually presenting its research progress, hotspots and frontier studies.

## Date and methods

### Data collection

The data were retrieved from the core collection databases of the Web of Science Core Collection (WoSCC) database. For further analysis, we extracted relevant literature information. Each downloaded study included the authors, title, abstract, descriptors, and identifiers. We saved them in the plain.txt format (including full records and cited references) [17]. After obtaining the consent of senior literature retrieval experts and all authors, the search terms for literature extraction were adopted as: TS = (‘tissue-resident macrophage’ OR ‘tissue-resident macrophages’ OR ‘tissue resident macrophage’ OR ‘tissue resident macrophages’) AND (‘neoplasia’ OR ‘neoplasias’ OR ‘neoplasm’ OR ‘tumor’ OR ‘tumors’ OR ‘cancer’ OR ‘cancers’ OR ‘malignant neoplasm’ OR ‘malignancy’ OR ‘malignancies’ OR ‘neoplasm, malignant’ OR ‘malignant neoplasms’ OR ‘neoplasms, malignant’ OR ‘neoplasms’). The article types included articles and reviews, and the publication language was limited to English. The retrieval time span was from the establishment of the database to 7 February 2025. To avoid deviations caused by the daily updates of the database, the publications were independently verified by two authors.

### Inclusion criteria

The inclusion criteria were as follows (1): Original publications on ‘tissue resident macrophages’ and ‘cancer’ published through peer review, including basic research, clinical research, and review articles (2); Publications indexed in the database before 7 February 2025 (3); Publications retrieved from the Web of Science.

### Exclusion criteria

The exclusion criteria were as follows (1): Publications that had not been formally published (2); Conference papers and abstract collections (3); Publications with duplicate publications (4); Publications that were irrelevant to the theme.

### Quality assessment

All English articles that were relevant to the theme and met the inclusion criteria were included in the analysis.

## Methods

We employed a diverse array of visualization tools for data analysis, namely Citespace (Version 5.7.R5), VOSviewer (Version 1.6.18), and the online platform (https://bibliometric.com/), along with two R software packages, Bibliometrix and Biblioshiny (Version 4.1.2) [18]. In the current study, CiteSpace was harnessed to compute the centrality of nodes, thereby uncovering their significance within the network and identifying highly cited references as well as keywords that exhibited citation peaks during specific time frames. VOSviewer is capable of constructing and visualizing the co-occurrence network of crucial terms culled from scientific literature [19]. In this research endeavor, we predominantly utilized co-authorship analysis and co-occurrence analysis to probe into the collaborative ties between authors and institutions. Moreover, we also resorted to the online bibliometric website (https://bibliometric.com/) to carry out a visual exploration of international cooperation among different nations.

## Results

### General information of the publication

**Figure 1.**
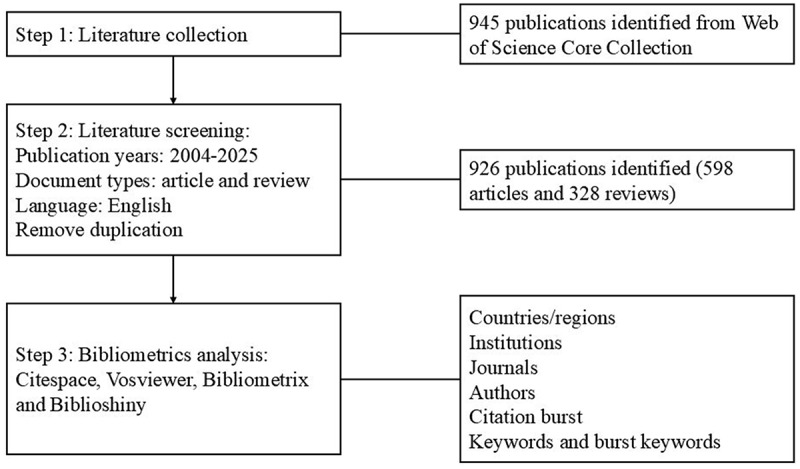
Process and key steps of the study. Tissue-resident macrophages (TRMs) in cancer bibliometric study workflow, including Web of Science Core Collection literature retrieval, screening (2004–2025, English, articles/reviews, duplicate removal), key information extraction (countries/regions, institutions), and analysis, covering journals, authors, citation bursts, keywords.

An overview of the research steps is shown in Figure 1. A total of 926 publications on tissue resident macrophages and cancer were included, consisting of 598 articles and 328 reviews. These publications originated from 1433 institutions in 57 countries and were published in 446 journals, with contributions from 6369 authors. The average annual growth rate of these publications was 13.02%. And 29.04% of the paper authors had international collaborations. The sum of the cited times was 72,969. The Average citations of per item was 78.80, and the h-index was 130.

**Figure 2.**
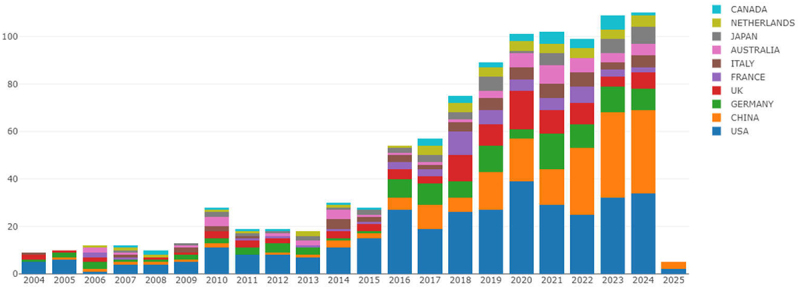
Annual publication trend of publications in the top 10 countries. Line chart of annual publication trends (2004–2025) for top 10 contributing countries in TRMs and cancer research, with vertical axis as publication count and horizontal axis as year.

**Figure 3.**
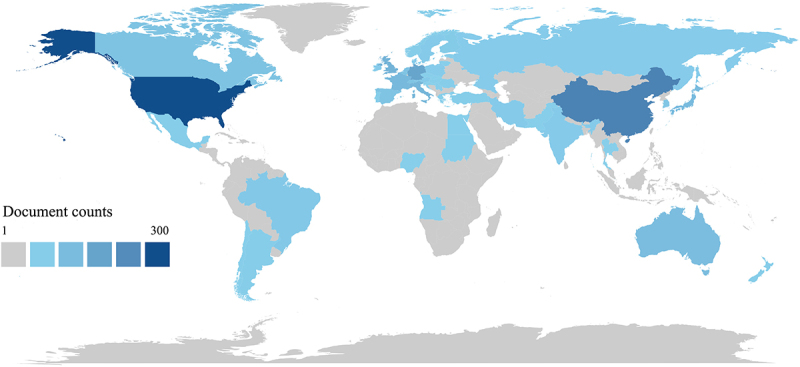
Distribution of countries with publications. Statistical chart of global country/region distribution of publications on TRMs and cancer.

Figure 2 illustrates the trend of annual publications in the top 10 countries in terms of the volume of published papers. Publications concerning TRMs and cancer emerged in 2004. The distribution of the number of publications manifested two conspicuous growth phases: a rapid expansion stage from 2016 to 2020 and a more subdued, yet steady growth stage from 2020 to 2024. From 2004 to 2021, the United States predominantly held the leading position in terms of annual publication volume. Table 1 showcases the publication numbers and citation frequencies of the top 10 countries. The United States topped the list with 274 publications and 29,120 citations, trailed closely by China, and then followed by Germany, the United Kingdom, and others. In terms of Citations per Publication, countries surpassing 100 citations include Italy, the Netherlands, the United States, and the United Kingdom (Table 1). The global distribution of publication volumes by country is depicted in Figure 3. The intensity of color denotes the number of publications, with darker hues signifying a greater number and lighter shades indicating fewer.

**Table 1.** The number of publications and citation frequencies.

Rank	Country	Articles	Citations	Citations of per publication
1	USA	274	29,120	106.3
2	CHINA	168	5943	35.4
3	GERMANY	76	5688	74.8
4	UNITED KINGDOM	53	5897	111.3
5	ITALY	41	5038	122.9
6	JAPAN	34	1106	32.5
7	AUSTRALIA	33	2839	86
8	FRANCE	32	2454	76.7
9	NETHERLANDS	26	2933	112.8
10	CANADA	25	2363	94.5

**Figure 4.**
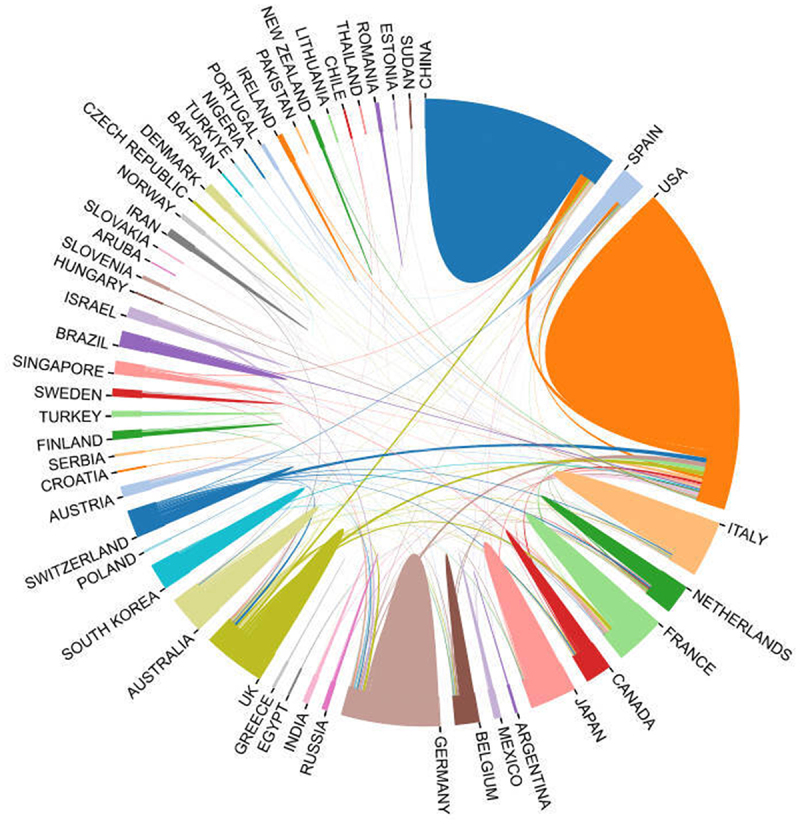
Cooperation between countries. Country/region cooperation network map in TRMs and cancer research, with nodes representing countries/regions and connections indicating collaborative relationships.

The cooperation relationships among various countries were depicted in Figure 4. Different colors were used to represent distinct countries, where the size of each colored area correlated with the number of publications. Meanwhile, the lines served to illustrate the collaborative ties between countries. The thicker a given line, the more frequent the cooperation between the affiliated countries. The United States engaged in rather active cooperation with other nations. Among its partners, China stood out as the closest collaborator, trailed by Switzerland, Germany, France, the United Kingdom, and others.

### Publication situation of institutions and cooperative relationships

Table 2 presents the number of publications and citation counts of top 10 institutions. Washington University tops the list with the highest number of publications, amounting to 54 articles. Next in line were Fudan University, the University of Edinburgh, and Shanghai Jiao Tong University. The total citation counts of Memorial Sloan Kettering Cancer Center, Icahn School of Medicine at Mount Sinai, and the University of Edinburgh all surpassed 400.

**Table 2.** Number of publications and citations of top 10 institutions.

Rank	Institution	Number	Cited number	Number of first authors	Cited number of first authors
1	Washington University	54	230	10	80
2	Fudan University	47	47	10	17
3	the University of Edinburgh	45	410	16	125
4	Shanghai Jiao Tong University	41	31	12	2
5	Memorial Sloan Kettering Cancer Center	38	435	8	108
6	Icahn School of Medicine at Mount Sinai	36	498	6	44
7	University of Minnesota	34	34	9	8
8	Baylor College of Medicine	34	16	6	0
9	Harvard Medical School	33	40	6	1
10	University of Oxford	30	13	10	5

**Figure 5.**
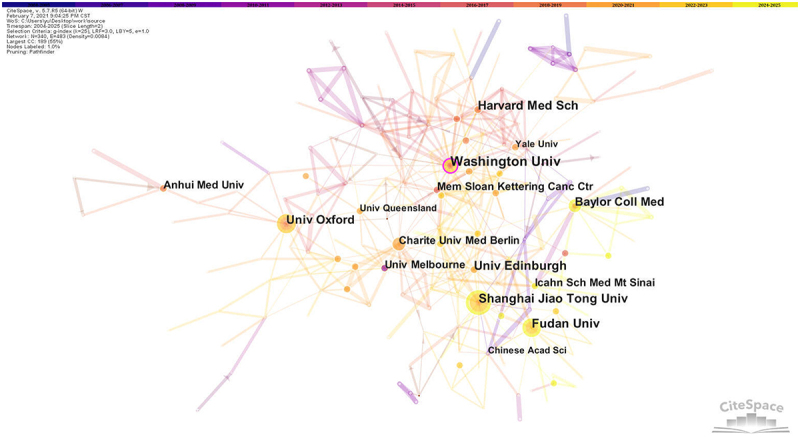
Institution co-occurrence map of TRMs in cancer research. Institution co-occurrence map for TRMs in cancer research, with nodes representing institutions, reflecting inter-institutional associations and cooperation intensity.

A centrality score exceeding 0.1 implies that these institutions wield significant visibility and direct influence within the network. Among these institutions, the Agency for Science, Technology and Research (Agcy Sci Technol & Res) boasted the highest centrality, standing at 0.17. Next in line were Washington University (0.13), the University of Manchester (0.09), Shanghai Jiao Tong University (0.08), and the University of Edinburgh (0.08). In the cooperation relationship graph among institutions (Figure 5), the link clusters represented author collaborations. The largest connected components of institutions co-occurrence contained 340 nodes and 483 connections with a map density of 0.0084. During the period from 2024 to 2025, Shanghai Jiao Tong University, Fudan University, Baylor Coll Med, and the University of Oxford published relatively frequently. Washington University exhibited the highest centrality and was most closely related to other institutions, and its node was marked with a purple circle.

### Top 10 journals in publication frequency

Among the journals with the largest number of published articles, most of them focused on research fields such as immunology, molecular biology, cancer, cell biology, and medicine (Table 3). In terms of the number of publications of journals, *Frontiers in Immunology* had the most publications, accounting for 6.80% of all publications, which is much higher than that of other magazines. Next is the *International Journal of Molecular Sciences*, with 22 publications, accounting for 2.38% of all publications.

**Table 3.** The top 10 most productive journals.

Rank	Journal	N	Country	IF	JCR-c
1	Frontiers in immunology	68	Switzerland	5.7	Q1
2	International Journal of Molecular Sciences	22	USA	4.9	Q1
3	Cancers	15	Switzerland	4.5	Q1
4	Cell Reports	14	USA	7.5	Q1
5	Immunity	14	USA	25.5	Q1
6	Journal of Immunology	13	USA	3.6	Q2
7	Frontiers in Cell and Developmental Biology	12	Switzerland	4.6	Q1
8	The Journal of Experimental Medicine	12	USA	12.6	Q1
9	Cells	11	Switzerland	5.1	Q2
10	PLOS ONE	11	USA	2.9	Q1

### Publication status of authors and co-authorship

Based on the number of citations, the top 10 authors are listed in Table 4. These authors had contributed significantly to the development of the field. Among them, DeNardo, DG had published only 6 papers, but the number of citation times was as high as 163, proving higher reference value and greater influence.

**Table 4.** The top 10 most productive authors.

Rank	Author	N	Co-citations	Average citations
1	DeNardo, DG	6	163	27.17
2	Randolph, GJ	8	148	18.5
3	Klemm, F	6	115	19.17
4	Joyce, JA	5	106	21.2
5	Hume, DA	9	79	8.78
6	Tacke, F	11	65	5.91
7	Geissmann, F	6	61	10.17
8	Murray, PJ	5	53	10.6
9	Pollard, J	5	52	10.4
10	Merad, M	5	44	8.8

**Figure 6.**
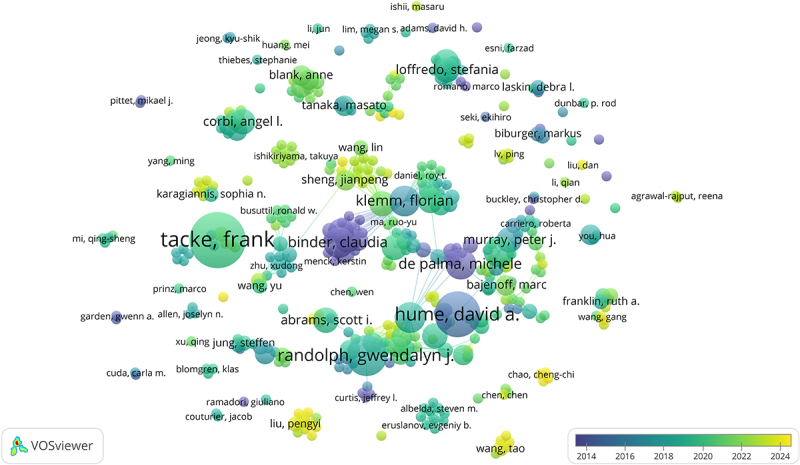
The authors’ cooperation. Author cooperation network map in TRMs and cancer research, with nodes representing authors and connections indicating collaborations.

Figure 6 depicts the co – authorship relationships. Node size indicates publication volume, and links between nodes represent author collaborations. Node and line colors denote publication time. Authors with high publication counts, like Tacke Frank, Hume DA, and Randolph GJ, had close collaborations. Hume DA closely collaborated with De Palma Michele, and Binder Claudia with Klemm Florian, highlighting the significance of teamwork in research. Conversely, some low – volume authors had no collaborative ties.

**Figure 7.**
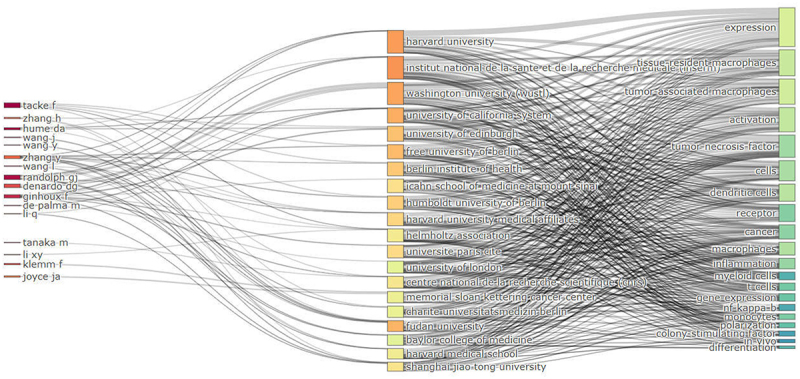
The author river map. River map showing research output and influence trends of core authors, institutions, and key terms in TRMs and cancer field.

The author river map delineated the intricate associations among authors, institutions, and research field keywords (Figure 7). The length of each node corresponds to the quantity of published works, while the lines signify the interconnections among authors, institutions, and research keywords. It showed the institutions that engage in the highest number of collaborative endeavors and the keywords that had been the subject of long – standing research. Authors like Tacke F, Zang Y, Randolph GJ, Denardo DG, and Ginhoux F exhibited a remarkable frequency of collaboration with a diverse array of institutions. Specifically, authors affiliated with Harvard University, Institut national de la santé et de la recherche médicale, and Washington University forged closer collaborative bonds with their peers. In the context of inter – institutional collaborations, the most prevalently investigated keywords encompass ‘expression,’ ‘tissue – resident macrophages,’ and ‘tumor – associated macrophages,’ among others.

### The top 10 most – cited publications

The citation frequency serves as a crucial metric for gauging the influence of a scholarly article. The top 10 most – cited publications is shown in Table 5. In the context of citation analysis, the article authored by Murray, PJ in 2017 stood out with the highest citation count, reaching 2006 citations. This article was published in *Annual Review of Physiology*, which boasted an impact factor (IF) of 19.1. Among the top 10 most cited publications, reviews constituted the predominant type (accounting for 70%), followed by experimental studies. Articles of the research type predominantly adopted single-cell sequencing and transcriptome sequencing as their primary research methodologies. The scope of the content encompassed a wide range of topics, including macrophage polarization, single – cell RNA – seq, Kupffer cells and tissue macrophage biology. The spectrum of diseases addressed in these articles included breast cancer, metastatic lung adenocarcinoma, liver cancer, hepatitis, and fibrosis.

**Table 5.** The top 10 most – cited publications.

Rank	Title	Article type	First author	Journal	IF	Year	Citation times
1	Macrophage Polarization	Review	Murray, PJ	Annual Review of Physiology	19.1	2017	2,006
2	Targeting macrophages: therapeutic approaches in cancer	Review	Cassetta, L	Nature Reviews Drug Discovery	101.8	2018	1553
3	Macrophages as regulators of tumor immunity and immunotherapy	Review	DeNardo, DG	Nature Reviews Immunology	60.9	2019	1514
4	From monocytes to M1/M2 macrophages: phenotypical vs. functional differentiation	Review	Italiani, P	Frontiers in Immunology	5.9	2014	1,462
5	Single-cell RNA sequencing demonstrates the molecular and cellular reprogramming of metastatic lung adenocarcinoma	Article	Kim, N	Nature Communications	15.7	2020	1,258
6	The cellular and molecular origin of tumor-associated macrophages	Article	Franklin, RA	Science	45.8	2014	1,098
7	Liver macrophages in tissue homeostasis and disease	Review	Krenkel, O	Nature Reviews Immunology	60.9	2017	959
8	Decoding cell death signals in liver inflammation	Review	Brenner, C	Journal of Hepatology	33	2013	763
9	Targeting hepatic macrophages to treat liver diseases	Review	Tacke, F	Journal of Hepatology	33	2017	708
10	Human Tumor-Associated Macrophage and Monocyte Transcriptional Landscapes Reveal Cancer-Specific Reprogramming, Biomarkers, and Therapeutic Targets	Article	Cassetta, L	Cancer Cell	44.5	2019	706

### Keyword co-occurrence, clusters, and burst

A keyword co-occurrence map was constructed using CiteSpace. A total of 454 keywords appeared. Among them, 10 keywords had an occurrence frequency greater than 100, and 21 keywords had an occurrence frequency of 50 or more. To conduct a classified discussion of the keywords, we grouped them according to ‘cell,’ ‘system and tissue,’ ‘bioactive factor,’ ‘biological process,’ and ‘disease.’ Table 6 summarizes the keywords with an occurrence frequency greater than 30. ‘Inflammation,’ ‘expression,’ ‘tumor associated macrophage,’ and ‘activation’ were the core content of the research on TRMs in the field of cancer research. ‘Tumor necrosis factor’ (0.11) and ‘Kupffer cell’ (0.1) exhibited a ‘bridge’ effect in the keyword co-occurrence map.

**Table 6.** Frequency and centrality of key words.

Types	Key words	Count	Centrality	Year
Cell	macrophage	297	0.05	2004
	tumor associated macrophage	134	0.04	2009
	dendritic cell	115	0.03	2008
	tissue resident macrophage	115	0.02	2014
	monocyte	109	0.03	2004
	T cell	82	0.05	2006
	microglia	77	0.04	2006
	myeloid cell	58	0.03	2010
	Kupffer cell	50	0.1	2004
	regulatory T cell	39	0.03	2010
	suppressor cell	37	0.01	2018
	tumor-associated macrophage	36	0.01	2016
System and tissue	central nervous system	36	0.07	2006
	bone marrow	35	0.03	2004
	adipose tissue	34	0.04	2009
Bioactive factor	tumor necrosis factor	98	0.11	2006
	cytokine	35	0.09	2004
	NF-κB	45	0.09	2005
	colony stimulating factor	41	0.05	2008
	TNF alpha	30	0.05	2006
Biological process	inflammation	178	0.07	2004
	expression	159	0.05	2004
	activation	123	0.03	2004
	polarization	74	0.02	2008
	gene expression	70	0.07	2005
	differentiation	56	0.02	2004
Disease	cancer	128	0.02	2005
	breast cancer	49	0.04	2012
	hepatocellular carcinoma	37	0.03	2014
	obesity	36	0.04	2008
Other key words	receptor	74	0.03	2009
	tumor microenvironment	73	0.01	2016
	immunotherapy	59	0.05	2012

### Keywords co-occurrence network

**Figure 8.**
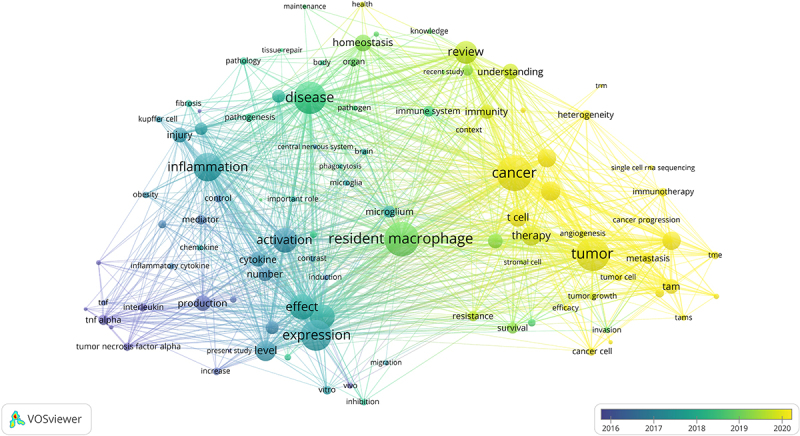
Keyword co-occurrence network. Keyword co-occurrence network map in TRMs and cancer research, with nodes representing keywords, reflecting core research topics and correlation.

Keyword co-occurrence network reflects the relationships between keywords over different periods and the evolution trends of keywords (Figure 8). In 2016, the research directions mainly revolved around the study of cytokines such as tumor necrosis factor alpha (TNFα) and interleukin. From 2017 to 2018, it gradually transitioned to research focusing on functions, with keywords like inflammation, activation, effect, disease, and important role. In the realm of inflammation – related research, terms like ‘injury,’ ‘activation,’ ‘effect,’ and ‘expression’ commonly appeared in conjunction. The investigations predominantly centered on cytokines, mediators, chemokines, and specifically, TNFα. Expression – focused research typically extended to aspects such as ‘level,’ ‘effect,’ ‘in vitro,’ ‘in vivo,’ and ‘inhibition.’ The primary emphasis is on exploring the levels and functions of gene expression, as well as vivo and vitro experiments. From 2017 to 2018, there was an increasing amount of research on the research directions related to microglia, the central nervous system, and the brain. By 2019, the research on resident macrophages had become a key focus, mainly based on immunity, and there was a further increase in the number of review-type articles. In 2020, the main research focused on studies related to treatment, as well as cancer progression and metastasis, and single cell RNA sequencing became a hot research direction. T cells, immunotherapy, heterogeneity, and tumor-associated macrophages (TAMs) were the research hotspots.

The keyword burst generated by CiteSpace takes the temporal distribution of keywords as the research object. This reflects the evolution of research hotspots over time, highlights emerging topics, and facilitates in – depth exploration of the research frontiers and development trends in this discipline. Table 7 shows the top 40 keywords with the strongest citation frequencies. Among them, the top five keywords ranked by burst intensity were tumor necrosis factor (10.52), tumor-microenvironment (7.92), cytokine (6.25), tumor microenvironment (6.23), and suppressor cell (6.01). The most recent peaks of keywords occurred in 2020 and 2022. Tumor microenvironment, landscape, immunotherapy, resident macrophage, response, tumor, tissue-resident macrophage, pathway, promote, and single cell were still showing a continuous burst trend, and this might herald future research hotspots.

**Table 7.** Key words with the strongest citation bursts.

Rank	Keywords	Weight	Begin	End	2004–2025
1	tumor necrosis factor	10.52	2004	2015	▃▃▃▃▃▃▃▃▃▃▃▃▂▂▂▂▂▂▂▂▂▂
2	cytokine	6.25	2004	2013	▃▃▃▃▃▃▃▃▃▃▂▂▂▂▂▂▂▂▂▂▂▂
3	NF-κB	4.9	2004	2013	▃▃▃▃▃▃▃▃▃▃▂▂▂▂▂▂▂▂▂▂▂▂
4	nitric oxide synthase	4.1	2004	2015	▃▃▃▃▃▃▃▃▃▃▃▃▂▂▂▂▂▂▂▂▂▂
5	lipopolysaccharide	3.41	2004	2009	▃▃▃▃▃▃▂▂▂▂▂▂▂▂▂▂▂▂▂▂▂▂
6	nitric oxide	4.91	2006	2013	▂▂▃▃▃▃▃▃▃▃▂▂▂▂▂▂▂▂▂▂▂▂
7	necrosis factor alpha	4.69	2006	2013	▂▂▃▃▃▃▃▃▃▃▂▂▂▂▂▂▂▂▂▂▂▂
8	central nervous system	3.66	2006	2015	▂▂▃▃▃▃▃▃▃▃▃▃▂▂▂▂▂▂▂▂▂▂
9	inflammation	3.45	2006	2009	▂▂▃▃▃▃▂▂▂▂▂▂▂▂▂▂▂▂▂▂▂▂
10	diet induced obesity	4.12	2008	2017	▂▂▂▂▃▃▃▃▃▃▃▃▃▃▂▂▂▂▂▂▂▂
11	insulin resistance	4.06	2008	2017	▂▂▂▂▃▃▃▃▃▃▃▃▃▃▂▂▂▂▂▂▂▂
12	mice	3.14	2008	2017	▂▂▂▂▃▃▃▃▃▃▃▃▃▃▂▂▂▂▂▂▂▂
13	induced insulin resistance	3.47	2010	2015	▂▂▂▂▂▂▃▃▃▃▃▃▂▂▂▂▂▂▂▂▂▂
14	adipose tissue	3.3	2010	2015	▂▂▂▂▂▂▃▃▃▃▃▃▂▂▂▂▂▂▂▂▂▂
15	mast cell	3.26	2010	2015	▂▂▂▂▂▂▃▃▃▃▃▃▂▂▂▂▂▂▂▂▂▂
16	innate immunity	3.21	2010	2013	▂▂▂▂▂▂▃▃▃▃▂▂▂▂▂▂▂▂▂▂▂▂
17	apoptosis	3.17	2010	2019	▂▂▂▂▂▂▃▃▃▃▃▃▃▃▃▃▂▂▂▂▂▂
18	bone marrow	3.16	2010	2015	▂▂▂▂▂▂▃▃▃▃▃▃▂▂▂▂▂▂▂▂▂▂
19	in vivo	4.81	2012	2019	▂▂▂▂▂▂▂▂▃▃▃▃▃▃▃▃▂▂▂▂▂▂
20	induction	3.43	2012	2017	▂▂▂▂▂▂▂▂▃▃▃▃▃▃▂▂▂▂▂▂▂▂
21	hematopoietic stem cell	5.39	2016	2019	▂▂▂▂▂▂▂▂▂▂▂▂▃▃▃▃▂▂▂▂▂▂
22	myeloid cell	3.87	2016	2019	▂▂▂▂▂▂▂▂▂▂▂▂▃▃▃▃▂▂▂▂▂▂
23	yolk sac	3.79	2016	2019	▂▂▂▂▂▂▂▂▂▂▂▂▃▃▃▃▂▂▂▂▂▂
24	rheumatoid arthriti	3.49	2016	2017	▂▂▂▂▂▂▂▂▂▂▂▂▃▃▂▂▂▂▂▂▂▂
25	glioma	3.28	2016	2017	▂▂▂▂▂▂▂▂▂▂▂▂▃▃▂▂▂▂▂▂▂▂
26	tumor-microenvironment	7.92	2018	2019	▂▂▂▂▂▂▂▂▂▂▂▂▂▂▃▃▂▂▂▂▂▂
27	suppressor cell	6.01	2018	2023	▂▂▂▂▂▂▂▂▂▂▂▂▂▂▃▃▃▃▃▃▂▂
28	stem cell	4.66	2018	2021	▂▂▂▂▂▂▂▂▂▂▂▂▂▂▃▃▃▃▂▂▂▂
29	endothelial growth factor	3.37	2018	2021	▂▂▂▂▂▂▂▂▂▂▂▂▂▂▃▃▃▃▂▂▂▂
30	tumor microenvironment	6.23	2020	2025	▂▂▂▂▂▂▂▂▂▂▂▂▂▂▂▂▃▃▃▃▃▃
31	heterogeneity	4.59	2020	2023	▂▂▂▂▂▂▂▂▂▂▂▂▂▂▂▂▃▃▃▃▂▂
32	macrophage polarization	3.74	2020	2021	▂▂▂▂▂▂▂▂▂▂▂▂▂▂▂▂▃▃▂▂▂▂
33	immunotherapy	5.36	2022	2025	▂▂▂▂▂▂▂▂▂▂▂▂▂▂▂▂▂▂▃▃▃▃
34	resident macrophage	4.76	2022	2025	▂▂▂▂▂▂▂▂▂▂▂▂▂▂▂▂▂▂▃▃▃▃
35	response	4.02	2022	2025	▂▂▂▂▂▂▂▂▂▂▂▂▂▂▂▂▂▂▃▃▃▃
36	tumor	3.93	2022	2025	▂▂▂▂▂▂▂▂▂▂▂▂▂▂▂▂▂▂▃▃▃▃
37	tissue-resident macrophage	3.76	2022	2025	▂▂▂▂▂▂▂▂▂▂▂▂▂▂▂▂▂▂▃▃▃▃
38	pathway	3.76	2022	2025	▂▂▂▂▂▂▂▂▂▂▂▂▂▂▂▂▂▂▃▃▃▃
39	promote	3.56	2022	2025	▂▂▂▂▂▂▂▂▂▂▂▂▂▂▂▂▂▂▃▃▃▃
40	single cell	3.25	2022	2025	▂▂▂▂▂▂▂▂▂▂▂▂▂▂▂▂▂▂▃▃▃▃

### Timeline view of the keyword co-occurrence

**Figure 9.**
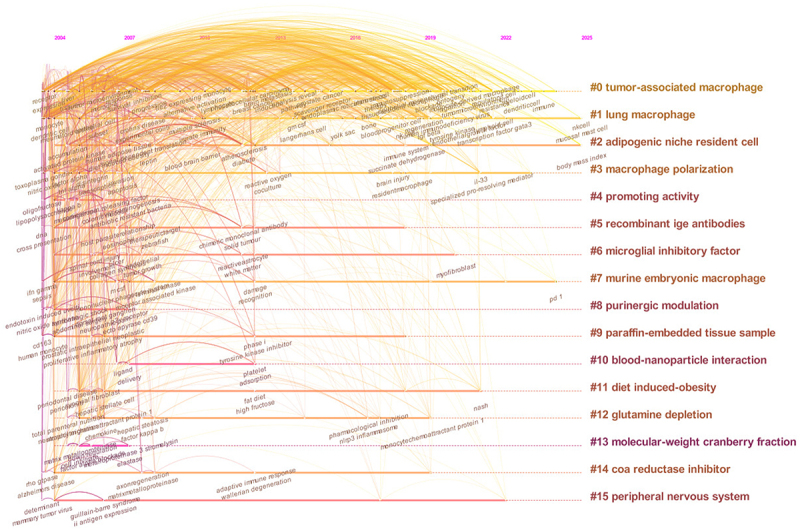
Timeline view of the keyword co-occurrence map. Timeline-based keyword co-occurrence map (2004–2025) for TRMs and cancer research, demonstrating temporal evolution of research hotspots.

Timeline view of the keyword co-occurrence map presented the dynamic evolution path of the research hotspots represented by the keywords (Figure 9). Different colors corresponded to diverse clusters, enabling an intuitive differentiation of the distribution and evolution of assorted research directions over time. The quantity of nodes on the timeline mirrored the level of research activity during a specific period. Cluster #0 (‘tumor-associated macrophage’) and Cluster #1 (‘lung macrophage’) possessed a substantial number of nodes, signifying plentiful research accomplishments and high research enthusiasm. Cluster #0 (‘tumor-associated macrophage’) featured the greatest number of connecting lines, suggesting excellent research continuity and inheritance, vigorous knowledge dissemination, and serving as a pivotal research area and the mainstream orientation. Cluster #1 (‘lung macrophage’) and Cluster #2 (‘adipogenic niche resident cell’) had sustained active research from 2004 to 2025, and the nodes emerging in 2025 might potentially represent novel research directions on the horizon.

## Discussion

### Current status of research

A total of 926 publications were incorporated into our research scope, with reviews accounting for 328 out of the 926. The timeframe from 2004 to 2015 marked the nascent stage of research concerning TRMs and cancer. During this period, the fundamental research efforts laid a robust groundwork for the subsequent advancements in this field. After 2015, there was a notable upward trajectory in the annual publication volume. Specifically, the years 2016–2020 witnessed a rapid expansion phase, followed by a steady growth stage from 2021 to 2024, clearly signifying that this research area had emerged as a prominent hot spot. The USA hitherto boasted the highest cumulative number of publications and exerted the greatest influence. Washington University made remarkable contributions to international integration and academic exchange, effectively serving as a crucial linchpin. Among the journals in which the articles were published, the research scopes predominantly centered around immunology, molecular biology, cancer studies, cell biology, and medicine. The subject matter of the top 10 most impactful publications encompassed macrophage polarization, Kupffer cells, tumor microenvironment and inflammation. Among immunocytes, the research on macrophages, dendritic cells, monocytes, T cells, and TRMs was common. Microglia and Kupffer cells, which belonged to TRMs in different tissues had been the most extensively studied. Single-cell RNA sequencing (scRNA-seq), tumor microenvironment and immunotherapy were key research directions. This phenomenon signaled a gradual transition in TRMs research within the tumor field, evolving from theoretical and fundamental investigations toward the exploration of immune cells within human tumor tissues in clinical settings.

### Research hotspots and important research findings

#### The origin and classification of TRMs

Our research showed that in the initial stage, the origin, differentiation, and distribution of TRMs, as well as their relationships with various cells and cytokines in the immune microenvironment, were the main research directions. However, due to the difficulty in isolating specific macrophages, the research findings based on animal models in the early research stage were difficult to be verified in humans [20]. Encouragingly, scRNA-seq enables researchers to delve deeply into the cellular origins and heterogeneity of TRMs in different tissues by identifying new subpopulations of TRMs with unique biological functions and clinical significance [21,22]. This is crucial for revealing the complex mechanisms by which human TRMs are involved in the tumor microenvironment. TRMs have different names in various tissues, such as microglia in the brain, Kupffer cells in the liver, Langerhans cells in the skin, alveolar macrophages in the lung, fat-associated macrophages in the adipose tissues, red pulp macrophages in the spleen, large peritoneal macrophage in the peritoneum, osteoclasts in the bone and so on. In adult individuals, when the proliferation of TRMs cells of embryonic origin is impaired, monocytes in the bone marrow will be abundantly replenished and differentiate into TRMs cells. Nevertheless, under normal physiological states, bone marrow-derived TRMs cells only account for a relatively minor proportion [5,7–10]. After monocyte-derived macrophages enter the tissue, they will gradually acquire characteristics similar to those of TRMs [23].

Compared with other macrophages, TRMs possess a high degree of tissue adaptability. They are responsible for maintaining tissue-specific homeostasis, local immune defense, facilitating tissue repair and regeneration, establishing immune tolerance, and modulating immune intensity [11]. TRMs in different locations exhibit distinct phenotypic characteristics, and they specifically regulate the tissue microenvironment according to the requirements of the host tissue [11]. For example, lung alveolar macrophages can clear dust particles and excessive surfactant on the alveolar surface, maintaining the normal gas exchange function of the alveoli. Kupffer cells in the liver can remove toxins and senescent red blood cells from the blood, keeping the internal environment of the liver stable. Resident macrophages in the heart can regulate the regeneration of cardiomyocytes and the formation of scar tissue [24].

#### Identification of TRMs

Identification of TRMs and their distinction from monocyte-derived macrophages (MDMs) require the integration of multi-dimensional information, including origin, surface markers, transcriptional profiles, self-renewal capacity, and functional phenotypes (Table 8). Fate-mapping techniques (e.g., Cx3cr1-CreERT2, Runx1-Cre) for labeling embryo-derived cells represent the most precise approach to distinguish them from bone marrow-derived cells. Alternatively, BrdU/EdU labeling assays or parabiosis models can be employed to verify their dependence on bone marrow-derived cell replenishment. The canonical markers for TRMs include CD45^+^, F4/80^+^ (or CD64^+^), and MerTK^+^ [25], with low expression or absence of Ly6C [26]. CD14 and FCN1, which are highly expressed in MDMs, are weakly expressed in TRMs [27], and can also serve as discriminative markers for the two subsets. Additionally, TRMs express tissue-specific signature molecules. Precise identification of TRMs requires the combination of their canonical markers with corresponding ‘tissue-specific markers.’ For instance, the tissue-specific markers of alveolar macrophages are CD11c^+^ and Siglec-F^+^ [28]. The tissue-specific markers of hepatic Kupffer cells are Tim4^+^ [29,30] and CD169^+^ [31]. The tissue-specific markers of cutaneous Langerhans cells are CD207^+^ [32] and CD1a^+^ [33]. The tissue-specific markers of cutaneous dermal macrophages are CD206^+^ [34] and CD11b^+^ [35]. The tissue-specific marker of cardiac interstitial TRMs is MerTK^+^ [36]. TRMs in the renal cortex and medulla highly express CD11b and weakly express MHCII [37]. The tissue-specific markers of microglia are TMEM119^+^, P2RY12^+^ [38], Iba1^+^, and CD11b^+^ [39]. Detecting the expression levels of these surface markers via flow cytometry (FACS) or immunofluorescence represents a practical approach for distinguishing TRMs from MDMs [40].

**Table 8.** Differences between TRMs and MDMs.

Feature/Dimension	Tissue-resident macrophages	Monocyte-derived macrophages
Origin	Yolk Sac and Fetal Liver	Adult Bone Marrow Hematopoietic Stem Cells (HSCs)
Maintenance	Local self-renewal	Continuous replenishment by peripheral monocytes
Markers	General markers: CD64^+^ MerTK^+^ CD14^−^ FCN1^−^ Tissue-specific markers: •Alveolar macrophages: CD11c^+^, Siglec-F^+^ •Kupffer cells: Tim4^+^, CD169^+^ •Skin Langerhans cells: CD207, CD1a • Skin dermal macrophages: CD206^+^, CD11b •Cardiac interstitial TRMs: MerTK •Kidney cortical and medullary TRMs: High CD11b^+^, Low MHCII •Microglia: TMEM119, P2RY12, Iba1, CD11b	CD14^+^ FCN1^+^ S100A8/9^+^
Transcriptional Profile	Tissue-specific (e.g., Alveolar Clec4f, Dermal Lyve1, etc.)	Inflammation-related (e.g., IL1B, TNF)
Functional Phenotype	Tissue homeostasis, repair, anti-inflammatory (M2-like)	Inflammatory response, antigen presentation (M1-like)
During Infection/Inflammation	Maintenance or expansion; functional plasticity	Massive recruitment; dominates acute inflammation
Biological Characteristics	Tissue-specific functions: •Microglia: Synaptic pruning •Alveolar macrophages: Clearance of dust, pathogens, and excess surfactant on the alveolar surface •Kupffer cells: Clearance of bacteria and endotoxins from portal blood; recycling iron from senescent red blood cells, etc.	Pro-inflammatory or reparative functions

#### Role of TRMs in cancer

In the early stage, TRMs may have a certain role in monitoring and suppressing tumor cells. However, during the process of cancer development and progression, TRMs may gradually transform toward the phenotype and function of tumor-associated macrophages (TAMs) under the influence of the tumor microenvironment [41]. At the same time, the presence of TAMs can also shape the tumor microenvironment and affect the function and state of TRMs. Both TAMs and TRMs are involved in tumor initiation, progression, metastasis, and immune evasion. They regulate inflammation, angiogenesis, and immune cell recruitment in the tumor microenvironment by secreting cytokines and chemokines, thus affecting tumor growth and spread [41].

Our study demonstrated that, in the context of diverse cancer types, the investigation into the role of TRMs has been preponderantly centered around their tumor-promoting capabilities. For example, in early-stage non-small cell lung cancer, TRMs could modify the tumor microenvironment. They promoted tumor cell epithelial–mesenchymal transition and triggered effective regulatory T cell responses, creating a niche that favors tumor growth [13]. Senescent alveolar macrophages aggregated early in lung adenocarcinoma and suppressed cytotoxic T cell responses [42]. In pancreatic ductal adenocarcinoma, proliferating tissue-resident macrophages promote fibrosis and suppress immunity. In transgenic mouse models, eliminating these macrophages alleviates fibrosis and immunosuppression, and restores the sensitivity of the cancer to chemotherapy [43]. In the mouse model of triple-negative breast cancer (TNBC), the local depletion of Mammary gland tissue-resident macrophages could significantly reduce the recurrence and distant metastasis of TNBC and improved the outcomes of chemotherapy, which indicated that MGTRMs play a crucial role in the growth and malignant progression of TNBC [44]. In the mouse model of ovarian cancer, CD163+Tim4+ TRMs in the omentum were maintained independently of bone marrow-derived monocytes and promoted the metastasis of ovarian cancer by supporting the acquisition of the epithelial–mesenchymal transition characteristics and stem cell-like phenotypes in ID 8 cells [45].

The tumor-promoting effect of TRMs is related to their secreted cytokines and the functions of their surface marker proteins. TRMs secrete chemokines like CCL2 and CXCL12, recruiting peripheral blood monocytes into the tumor microenvironment, which then differentiate into tumor-promoting TAMs [46]. Inhibitory cytokines like IL-10, IL-4 and VEGF secreted by TRMs can suppress the activity and proliferation of CD8+ T cells, boost the expansion of Tregs, and consume arginine in the microenvironment via Arg-1, inhibiting T cell metabolic activity and weakening the anti-tumor immune response [46]. Pro-angiogenic factors such as VEGF, FGF2 and MMP9 secreted by TRMs can also directly stimulate the proliferation of endothelial cells and the formation of blood vessels [47]. In addition, TRMs highly express PD-L1, which binds to PD-1 on T cells, activating SHP2 phosphatase. This dephosphorylates key TCR signaling molecules like ZAP70 and CD28, inhibiting T cell activation and effector function [48]. Additionally, TRMs participate in tumor microenvironment remodeling and tumor cell metabolic regulation. They directly affect tumor growth and fibrosis, promoting extracellular matrix remodeling by secreting pro – fibrotic factors (e.g., TGF -β, PDGF) to form a tumor – supportive microenvironment. For example, TRMs in pancreatic cancer promote tumor fibrosis by generating collagen and fibronectin [47]. In the lung metastasis microenvironment, PD-L1 on TRMs binds to PD-1 on myeloid cells, inhibiting STAT1 signaling. This indirectly suppresses the IFN-γ-CXCL9 pathway, decreasing CXCL9/CXCL10 secretion and impeding the recruitment of CD8+ T cells to the tumor [49]. Intraperitoneal resident tissue-resident macrophages, such as peritoneal macrophages, bind to phosphatidylserine on the surface of CD8+ T cells through Tim-4, inhibiting their cytotoxicity and impeding the efficacy of anti-PD-1 antibodies [48]. In hypoxic tumor regions, TRMs form tubular structures through self – assembly (vascular mimicry) to assist in the delivery of oxygen and nutrients, thereby supporting tumor growth [50].

#### Frontier researches on different types of TRMs in cancer

##### Microglia

Microglia originate from yolk sac macrophages during the embryonic period. These cells migrate to the central nervous system during embryonic development and become microglia. In adulthood, microglia maintain their numbers through self-renewal. However, under certain pathological conditions, monocytes in the peripheral blood can also enter the central nervous system and differentiate into microglia-like cells [51]. Researchers found that hypoxia induces glioma stem cells to produce high levels of glutamate, which activates local neurons. By upregulating miR-200c-3p in neuronal exosomes, it promoted M2 polarization of microglia and the malignant progression of brain glioma cells [52]. Exosomal miR-374b-3p secreted by glioma stem cells can induce the M2 polarization of macrophages, and then enhance the migration of vascular endothelial cells and the ability of angiogenesis [53]. On 13 May 2024, a research team from Southern Medical University published a paper in *Cell Reports Medicine*, stating that the upregulation of HSP47 in tumor cells drives metastatic colonization and growth in the brain by creating an immunosuppressive microenvironment. Specifically, HSP47-mediated collagen deposition in metastases promotes the polarization of microglia to the M2 phenotype via the α2β1 integrin/nuclear factor κB pathway. And it upregulates anti-inflammatory cytokines and inhibits the anti – tumor response of CD8+ T cells [54]. Microglia can secrete extracellular matrix components and chemokines, which affect the migration and invasion of tumor cells [55]. At the same time, they also provide a supportive growth environment for tumor cells. Microglial cells can also suppress the functions of immune cells such as T cells by expressing immunosuppressive molecules like PD-L1, thus helping tumor cells evade the body’s immune surveillance [56]. However, microglia do not invariably display pro-cancer effects. As Katrina T Evans et al. discovered in their research, in a mouse model of breast cancer brain metastasis, microglia served to promote inflammation and suppressed the brain metastasis of breast cancer. Mice deficient in microglia demonstrated an augmented rate of metastasis, a diminished survival rate, as well as weakened responses of natural killer cells and T cells [57].

In March 2025, the research team led by Gema Moreno – Bueno utilized mouse models and single – cell transcriptome analysis techniques to uncover a biphasic role of microglia in melanoma brain metastasis [58]. In the early stage of melanoma brain metastasis, microglia played an anti – tumor role by phagocytosing tumor cells. But as the tumor grew and colonized the brain, they switched to a tumor – promoting phenotype. The activity of the Rela/NF - κB signaling pathway in related microglia was enhanced. Targeting it could reprogram microglia to be pro – inflammatory, strengthening the anti – tumor immune response, reducing metastatic tumor burden, and improving immunotherapy effectiveness. Moreover, single – cell transcriptome data of patients receiving immunotherapy showed a positive correlation between microglia’s pro – inflammatory phenotype and immunotherapy response, implying potential clinical benefits of targeting NF - κB – triggered reprogramming.

The research team from Sichuan University performed single-cell transcriptome sequencing on CD45+ cells in tumor-bearing mice and discovered that oxidative stress-related genes were enriched in tumor-associated microglia [59]. Under oxidative stress, microglia turn immunosuppressive, with weakened antigen presentation and upregulated CD8 + T cell checkpoint molecules. Further research showed that microglia could promote tumors via the NR4A2/SQLE pathway. Knocking out NR4A2 in microglia restores antigen presentation, boosting CD8 + T cell anti-tumor effects. Combining NR4A2/SQLE inhibitor with PD-1 antibody could activate CD8 + T cells better than PD-1 antibody alone and prolong mouse survival, offering a new glioblastoma treatment strategy.

##### Kupffer cells

During embryonic development, Kupffer cells primarily stem from erythroid-myeloid progenitor cells (EMPs) present in the yolk sac and fetal liver. In adult organisms, whenever the proliferation of embryonically-derived Kupffer cells is compromised, a large number of monocytes from the bone marrow will be recruited to supplement and subsequently differentiate into Kupffer cells. Nevertheless, under normal physiological circumstances, Kupffer cells of bone marrow origin constitute a relatively minor fraction [60]. Kupffer cells are localized adjacent to sinusoids and exert a dual function in liver cancer immunity. Endowed with remarkable clearance and phagocytic capabilities, these cells can directly capture and eradicate circulating cancer cells by means of C-type lectins and Fc receptors, consequently impeding the invasion of liver tumors [61,62]. However, some studies have also found that exosomes derived from liver cancer cells can induce Kupffer cells to transform into tumor-associated macrophages (TAMs). Kupffer cells can be stimulated by tumor cells to secrete immunosuppressive cytokines, facilitating the metastasis of liver cancer via the IL6-JAK1-ACAP4 axis [63]. Under certain circumstances, Kupffer cells can contribute to the development of liver fibrosis, creating conditions for the occurrence of liver cancer [64,65].

Targeting Kupffer cells demonstrated substantial therapeutic potential in the context of liver metastases. Specifically, certain bacteria have been found to trigger significant proliferation and functional reprogramming of Kupffer cells. This reprogramming allows Kupffer cells to engulf cancer cells and transition into pro-inflammatory macrophages. Subsequently, the anti-tumor efficacy of T cells is augmented, thereby effectively suppressing the development of liver metastases in malignant tumors [66]. Enhancing the biogenesis of miR − 206 can drive the M1 polarization of Kupffer cells, promote the recruitment of CD8 + T cells and prevent hepatocellular carcinoma [67]. The team led by Prof. Zutian Zeng found that targeted editing of transcription factors MafB/c-Maf in Kupffer cells can effectively eliminate tumors in mouse models of liver metastases from melanoma, colorectal cancer and lung cancer. It does so mainly by inducing rapid proliferation and pro-inflammatory function changes of tumor-associated Kupffer cells, increasing their infiltration and accumulation in tumors, reshaping the tumor immune microenvironment via macrophage polarization, and triggering anti-tumor T-cell responses [66]. The research on Kupffer cells, obesity and fatty liver is also a hot topic [68]. Eliminating Kupffer cells or blocking TNF can significantly reduce tumor-induced fatty liver generation [69].

### Research directions and challenges

Currently, pexidartinib, a drug targeting macrophage polarization, has received US Food and Drug Administration’s approval for treating tenosynovial giant cell tumor. It directly suppresses tumor cell proliferation and invasion by mitigating pro-tumorigenic activities of TAMs, including angiogenesis and extracellular matrix degradation [70]. The exploration of therapeutic targets and signaling pathways for TRMs in cancer treatment remains an active area of research. Strategies include inducing macrophage depletion, inhibiting recruitment, promoting repolarization, and combining with agents such as immune checkpoint inhibitors [46].

However, studying TRMs in cancer therapy is challenging due to species – specific markers and significant transcriptional and phenotypic heterogeneity across different tissues [71]. Even within a single tumor, macrophage polarization and function go beyond the simple M1/M2 classification. There are multiple intermediate states that change dynamically, depending on the tumor stage, treatment methods, and the changing cues in the tumor microenvironment [72], which complicates the research on TRM cells. Furthermore, TRMs interact intricately with other cells within the tumor microenvironment. When one signaling pathway is regulated, other signals might compensate, heightening the complexity and uncertainty of treatment. Sufficient clinical research data to support the optimal combination treatment regimens are still lacking.

### Limitations

This study presents several limitations. Firstly, the articles incorporated in our research were solely sourced from the Web of Science Core Collection (WOSCC). Consequently, the dataset under analysis lacks comprehensiveness, potentially overlooking valuable insights from other sources. Secondly, research based on CiteSpace generally offers relatively cursory analyses, like simply depicting high-frequency keywords, key authors, research institutes and so on. Although we have delved deeply into the internal relationships, theoretical foundations, and evolutionary mechanisms among the publications by reading a large number of documents, due to space limitations, in – depth analysis of the research still requires a large amount of data support.

## Conclusions

This study comprehensively summarizes the current research status, hotspots and trends of TRMs in the cancer field, providing valuable insights for potential collaborators and institutions. With the continuous advancement of research methodologies, our understanding of TRMs’ heterogeneity across various tissues, as well as their roles and mechanisms in malignant tumor progression, has been enhanced. In cancer treatment, strategies targeting TRMs involve inducing TRMs depletion, suppressing TAMs recruitment, promoting repolarization, among others. The immunotherapy targets related to TRMs are still being explored, and further studies are essential to validate their efficacy and safety in clinical applications.

## Supplementary Material

supplemental document.docx

## Data Availability

The original research findings presented in this study are incorporated in the article or the Supplementary Material. For any further questions or inquiries, please contact the first authors.
