# Supplementary material for: Research on tissue-resident macrophages in the field of cancer research: a bibliometric analysis from 2004 to 2025
Source: Cell Adh Migr. 2026 Feb 2;20(1):2624204. doi: 10.1080/19336918.2026.2624204 (PMC12867409; doi:10.1080/19336918.2026.2624204)
Supplement: supplemental document.docx [file KCAM_A_2624204_SM9681.docx]

FN Clarivate Analytics Web of Science

VR 1.0

PT J

AU Kloosterman, DJ

Akkari, L

AF Kloosterman, Daan J.

Akkari, Leila

TI Macrophages at the interface of the co-evolving cancer ecosystem

SO CELL

LA English

DT Review

ID TUMOR-ASSOCIATED MACROPHAGES; TISSUE-RESIDENT MACROPHAGES; TNF-ALPHA;

MYELOID CELLS; UP-REGULATION; PROGRESSION; MICROENVIRONMENT;

INFLAMMATION; EXPRESSION; EVOLUTION

AB Macrophages are versatile and heterogeneous innate immune cells undertaking central functions in balancing immune responses and tissue repair to maintain homeostasis. This plasticity, once co-opted by malignant outgrowth, orchestrates manifold reciprocal interactions within the tumor microenvironment, fueling the evolution of the cancer ecosystem. Here, we review the multilayered sources of influence that jointly underpin and longitudinally shape tumor-associated macrophage (TAM) phenotypic states in solid neoplasms. We discuss how, in response to these signals, TAMs steer tumor evolution in the context of natural selection, biological dispersion, and treatment resistance. A number of research frontiers to be tackled are laid down in this review to therapeutically exploit the complex roles of TAMs in cancer. Building upon knowledge obtained from currently applied TAM-targeting strategies and using next generation technologies, we propose conceptual advances and novel therapeutic avenues to rewire TAM multifaceted regulation of the co-evolving cancer ecosystem.

C1 [Kloosterman, Daan J.; Akkari, Leila] Netherlands Canc Inst, Oncode Inst, Div Tumor Biol & Immunol, Plesmanlaan 121, NL-1066CX Amsterdam, Netherlands.

C3 Netherlands Cancer Institute

RP Akkari, L (corresponding author), Netherlands Canc Inst, Oncode Inst, Div Tumor Biol & Immunol, Plesmanlaan 121, NL-1066CX Amsterdam, Netherlands.

EM l.akkari@nki.nl

OI Kloosterman, Daan/0000-0003-1082-070X; Akkari, Leila/0000-0001-5479-641X

FU Oncode Institute, NWO [91719355]; Dutch Cancer Society [KWF 14530, KWF

10658]; Brain Tumor Funders Challenge

FX We thank members of the Akkari lab and particularly Daniel Taranto for

insight- ful discussions. This study was supported by Oncode Institute,

NWO Vidi program (91719355 to L.A.) , Dutch Cancer Society (KWF 14530,

KWF 10658) , and the Brain Tumor Funders Challenge (L.A.) .

CR Advani R, 2018, NEW ENGL J MED, V379, P1711, DOI 10.1056/NEJMoa1807315

Aegerter H, 2022, IMMUNITY, V55, P1564, DOI 10.1016/j.immuni.2022.08.010

Akkari L, 2020, SCI TRANSL MED, V12, DOI 10.1126/scitranslmed.aaw7843

Aktipis CA, 2012, CANCER PREV RES, V5, P266, DOI 10.1158/1940-6207.CAPR-11-0004

Aldinucci D, 2020, CANCERS, V12, DOI 10.3390/cancers12071765

Allavena P, 2021, MOL MED, V27, DOI 10.1186/s10020-021-00383-3

Altea-Manzano P, 2020, EMBO REP, V21, DOI 10.15252/embr.202050635

Amirouchene-Angelozzi N, 2017, CANCER DISCOV, V7, P805, DOI 10.1158/2159-8290.CD-17-0343

Anderson ARA, 2006, CELL, V127, P905, DOI 10.1016/j.cell.2006.09.042

Antunes ARP, 2021, NAT NEUROSCI, V24, P595, DOI 10.1038/s41593-020-00789-y

Arias-Alpizar G, 2020, NAT COMMUN, V11, DOI 10.1038/s41467-020-17360-9

Ayasoufi K, 2020, BRAIN, V143, P3629, DOI 10.1093/brain/awaa343

Bader J.E., 2022, PD 1 IS INDUCED TUMO, DOI [10.1101/2022.11. 12.515348, DOI 10.1101/2022.11.12.515348]

Bai RX, 2022, MOL CANCER, V21, DOI 10.1186/s12943-022-01645-2

Ban Y, 2017, CANCER RES, V77, P2857, DOI 10.1158/0008-5472.CAN-16-2913

Bauml J, 2021, J CLIN ONCOL, V39, DOI 10.1200/JCO.2021.39.15_suppl.TPS2660

Bhang HEC, 2015, NAT MED, V21, P440, DOI 10.1038/nm.3841

Bhat KPL, 2013, CANCER CELL, V24, P331, DOI 10.1016/j.ccr.2013.08.001

Bonapace L, 2014, NATURE, V515, DOI 10.1038/nature13862

Borriello L, 2022, NAT COMMUN, V13, DOI 10.1038/s41467-022-28076-3

Boumahdi S, 2020, NAT REV DRUG DISCOV, V19, P39, DOI 10.1038/s41573-019-0044-1

Brempelis KJ, 2020, J IMMUNOTHER CANCER, V8, DOI 10.1136/jitc-2020-001356

Brown KF, 2018, BRIT J CANCER, V118, P1130, DOI 10.1038/s41416-018-0029-6

Broz ML, 2014, CANCER CELL, V26, P638, DOI 10.1016/j.ccell.2014.09.007

Carpenter KJ, 2019, SCI REP-UK, V9, DOI 10.1038/s41598-019-56038-1

Carter JM, 2018, CANCER PREV RES, V11, P113, DOI 10.1158/1940-6207.CAPR-17-0245

Chang CH, 2015, CELL, V162, P1229, DOI 10.1016/j.cell.2015.08.016

Chen AX, 2021, GENOME MED, V13, DOI 10.1186/s13073-021-00906-x

Chen Q, 2011, CANCER CELL, V20, P538, DOI 10.1016/j.ccr.2011.08.025

Chen SY, 2020, CELL MOL IMMUNOL, V17, P36, DOI 10.1038/s41423-019-0315-0

Chow A, 2021, CANCER CELL, V39, P973, DOI 10.1016/j.ccell.2021.05.006

Christofides A, 2022, NAT IMMUNOL, V23, P1148, DOI 10.1038/s41590-022-01267-2

Chu YX, 2010, P NATL ACAD SCI USA, V107, P7975, DOI 10.1073/pnas.0913449107

Comito G, 2014, ONCOGENE, V33, P2423, DOI 10.1038/onc.2013.191

Condeelis J, 2006, CELL, V124, P263, DOI 10.1016/j.cell.2006.01.007

Costa-Silva B, 2015, NAT CELL BIOL, V17, P816, DOI 10.1038/ncb3169

Croci D, 2022, SCI TRANSL MED, V14, DOI 10.1126/scitranslmed.abo2952

de Back DZ, 2014, FRONT PHYSIOL, V5, DOI 10.3389/fphys.2014.00009

de Palma M, 2017, NAT REV CANCER, V17, P457, DOI 10.1038/nrc.2017.51

DeNardo DG, 2019, NAT REV IMMUNOL, V19, P369, DOI 10.1038/s41577-019-0127-6

DeNardo DG, 2011, CANCER DISCOV, V1, P54, DOI 10.1158/2159-8274.CD-10-0028

Desai P, 2018, NAT MED, V24, P1015, DOI 10.1038/s41591-018-0081-z

Dhatchinamoorthy K, 2021, FRONT IMMUNOL, V12, DOI 10.3389/fimmu.2021.636568

Dinarello CA, 2009, ANNU REV IMMUNOL, V27, P519, DOI 10.1146/annurev.immunol.021908.132612

Ding CL, 2023, NAT IMMUNOL, V24, P239, DOI 10.1038/s41590-022-01388-8

Dixit A, 2022, JCI INSIGHT, V7, DOI 10.1172/jci.insight.153242

Duraiswamy J, 2021, CANCER CELL, V39, P1623, DOI 10.1016/j.ccell.2021.10.008

Egeblad M, 2010, CURR OPIN CELL BIOL, V22, P697, DOI 10.1016/j.ceb.2010.08.015

Eisel D, 2019, FRONT IMMUNOL, V10, DOI 10.3389/fimmu.2019.00219

El-Kenawi A, 2021, CANCER RES, V81, P5477, DOI 10.1158/0008-5472.CAN-20-4028

Elliott MR, 2009, NATURE, V461, P282, DOI 10.1038/nature08296

Engelhardt JJ, 2012, CANCER CELL, V21, P402, DOI 10.1016/j.ccr.2012.01.008

Erbani J, 2022, SEMIN CANCER BIOL, V86, P41, DOI 10.1016/j.semcancer.2022.05.003

Fedele M, 2019, INT J MOL SCI, V20, DOI 10.3390/ijms20112746

Fisher G.A., 2020, J CLIN ONCOL

Florey O, 2019, PHILOS T R SOC B, V374, DOI 10.1098/rstb.2018.0154

Franklin RA, 2014, SCIENCE, V344, P921, DOI 10.1126/science.1252510

Friedrich M, 2021, NAT CANCER, V2, P723, DOI 10.1038/s43018-021-00201-z

Fukushi A, 2022, INT J MOL SCI, V23, DOI 10.3390/ijms231710037

Fuller AD, 2008, J NEUROIMMUNE PHARM, V3, P246, DOI 10.1007/s11481-008-9118-2

Gartung A., 2019, P NATL ACAD SCI USA

Gatenby RA, 2020, NAT REV CLIN ONCOL, V17, P675, DOI 10.1038/s41571-020-0411-1

Goossens P, 2019, CELL METAB, V29, P1376, DOI 10.1016/j.cmet.2019.02.016

Gordon SR, 2017, NATURE, V545, P495, DOI 10.1038/nature22396

Greten FR, 2019, IMMUNITY, V51, P27, DOI 10.1016/j.immuni.2019.06.025

Guan W, 2021, GENES-BASEL, V12, DOI 10.3390/genes12050773

Gupta S, 2017, FRONT ONCOL, V7, DOI 10.3389/fonc.2017.00068

Habanjar O, 2022, BIOLOGY-BASEL, V11, DOI 10.3390/biology11020339

Halama N, 2016, CANCER CELL, V29, P587, DOI 10.1016/j.ccell.2016.03.005

Hampton HR, 2019, FRONT IMMUNOL, V10, DOI 10.3389/fimmu.2019.01168

Hanahan D, 2022, CANCER DISCOV, V12, P31, DOI 10.1158/2159-8290.CD-21-1059

Harney AS, 2015, CANCER DISCOV, V5, P932, DOI 10.1158/2159-8290.CD-15-0012

Hernandez C, 2016, ONCOGENE, V35, P5931, DOI 10.1038/onc.2016.104

Hiratsuka S, 2008, NAT CELL BIOL, V10, P1349, DOI 10.1038/ncb1794

Hiratsuka S, 2006, NAT CELL BIOL, V8, P1369, DOI 10.1038/ncb1507

Hudson K, 2020, FRONT IMMUNOL, V11, DOI 10.3389/fimmu.2020.568931

Hughes R, 2015, CANCER RES, V75, P3479, DOI 10.1158/0008-5472.CAN-14-3587

Iwasaki A, 2004, NAT IMMUNOL, V5, P987, DOI 10.1038/ni1112

Jaitin DA, 2019, CELL, V178, P686, DOI 10.1016/j.cell.2019.05.054

Jang JH, 2020, CANCER RES, V80, P1342, DOI 10.1158/0008-5472.CAN-19-2288

Jing YY, 2018, CANCER LETT, V434, P22, DOI 10.1016/j.canlet.2018.07.001

June CH, 2018, NEW ENGL J MED, V379, P64, DOI 10.1056/NEJMra1706169

Karimi E, 2023, NATURE, V614, P555, DOI 10.1038/s41586-022-05680-3

Kaushik NK, 2019, CANCERS, V11, DOI 10.3390/cancers11060842

Kessenbrock K, 2010, CELL, V141, P52, DOI 10.1016/j.cell.2010.03.015

Kielbassa K, 2019, FRONT IMMUNOL, V10, DOI 10.3389/fimmu.2019.02215

Kim JJ, 2010, CELL DEATH DIFFER, V17, P1420, DOI 10.1038/cdd.2010.19

Kim MK, 2020, INTEGR CANCER THER, V19, DOI 10.1177/1534735420949678

Kim SH, 2020, NEOPLASIA, V22, P606, DOI 10.1016/j.neo.2020.08.006

Klichinsky M, 2020, NAT BIOTECHNOL, V38, P947, DOI 10.1038/s41587-020-0462-y

Kobatake K, 2020, CLIN CANCER RES, V26, P2065, DOI 10.1158/1078-0432.CCR-19-2230

Komohara Y, 2012, CANCER SCI, V103, P2165, DOI 10.1111/cas.12015

Korbecki J, 2021, INT J MOL SCI, V22, DOI 10.3390/ijms22020843

Kourtzelis I, 2020, FRONT IMMUNOL, V11, DOI 10.3389/fimmu.2020.00553

Kowal J, 2019, IMMUNOTHERAPY-UK, V11, P677, DOI 10.2217/imt-2018-0156

Lahmar Q, 2016, BBA-REV CANCER, V1865, P23, DOI 10.1016/j.bbcan.2015.06.009

Laoui D, 2014, FRONT IMMUNOL, V5, DOI 10.3389/fimmu.2014.00489

Larionova I, 2020, CANCERS, V12, DOI 10.3390/cancers12061411

Laviron M, 2019, FRONT IMMUNOL, V10, DOI 10.3389/fimmu.2019.01799

Lecoultre M, 2020, J IMMUNOTHER CANCER, V8, DOI 10.1136/jitc-2020-001408

Lee CH, 2010, J IMMUNOTHER, V33, P73, DOI 10.1097/CJI.0b013e3181b7a0a4

Lee D, 2021, J LIPID RES, V62, DOI 10.1016/j.jlr.2021.100117

Lehrman EK, 2018, NEURON, V100, P120, DOI 10.1016/j.neuron.2018.09.017

Leone RD, 2019, SCIENCE, V366, P1013, DOI 10.1126/science.aav2588

Lercher A, 2020, IMMUNITY, V53, P496, DOI 10.1016/j.immuni.2020.08.012

Lérias JR, 2020, FRONT MICROBIOL, V10, DOI 10.3389/fmicb.2019.02924

Lewis CE, 2006, CANCER RES, V66, P605, DOI 10.1158/0008-5472.CAN-05-4005

Li B, 2010, FASEB J, V24, P4767, DOI 10.1096/fj.10-154757

Li CW, 2012, CANCER RES, V72, P1290, DOI 10.1158/0008-5472.CAN-11-3123

Li W, 2012, J CELL PHYSIOL, V227, P1291, DOI 10.1002/jcp.22815

Li YN, 2017, AM J TRANSL RES, V9, P2901

Lin YX, 2019, J HEMATOL ONCOL, V12, DOI 10.1186/s13045-019-0760-3

Linehan D., 2018, J CLIN ONCOL

Liu XJ, 2020, NATURE, V588, P693, DOI 10.1038/s41586-020-2911-7

Marconi GD, 2021, CELLS-BASEL, V10, DOI 10.3390/cells10071587

Marelli G, 2022, J IMMUNOTHER CANCER, V10, DOI 10.1136/jitc-2022-004584

Matsumura H, 2014, INT J ONCOL, V45, P2303, DOI 10.3892/ijo.2014.2662

McDowell SAC, 2021, NAT CANCER, V2, P545, DOI 10.1038/s43018-021-00194-9

McGrail DJ, 2021, ANN ONCOL, V32, P661, DOI 10.1016/j.annonc.2021.02.006

Miranda D, 2016, BIOENG TRANSL MED, V1, P267, DOI 10.1002/btm2.10032

Molgora M, 2020, CELL, V182, P886, DOI 10.1016/j.cell.2020.07.013

Mooney HA, 2001, P NATL ACAD SCI USA, V98, P5446, DOI 10.1073/pnas.091093398

Müller E, 2018, FRONT IMMUNOL, V9, DOI 10.3389/fimmu.2018.02520

Nam AS, 2021, NAT REV GENET, V22, P3, DOI 10.1038/s41576-020-0265-5

Neefjes J, 2011, NAT REV IMMUNOL, V11, P823, DOI 10.1038/nri3084

Netea MG, 2020, NAT REV IMMUNOL, V20, P375, DOI 10.1038/s41577-020-0285-6

Nixon BG, 2022, IMMUNITY, V55, P2044, DOI 10.1016/j.immuni.2022.10.002

Nobs SP, 2021, TRENDS IMMUNOL, V42, P495, DOI 10.1016/j.it.2021.04.007

Olafsson S, 2021, TRENDS GENET, V37, P872, DOI 10.1016/j.tig.2021.06.012

Olson OC, 2015, NAT REV CANCER, V15, P712, DOI 10.1038/nrc4027

Opperman KS, 2019, NEOPLASIA, V21, P777, DOI 10.1016/j.neo.2019.05.006

Paolicelli RC, 2011, SCIENCE, V333, P1456, DOI 10.1126/science.1202529

Park EJ, 2010, CELL, V140, P197, DOI 10.1016/j.cell.2009.12.052

Peranzoni E, 2018, P NATL ACAD SCI USA, V115, pE4041, DOI 10.1073/pnas.1720948115

Perez-Castro L, 2023, FEBS J, V290, P7, DOI 10.1111/febs.16245

Perez-Diez A, 2022, CANCER RES COMMUN, V2, P316, DOI [10.1158/2767-9764.CRC-22-0052, 10.1158/2767-9764.crc-22-0052]

Perillo B, 2020, EXP MOL MED, V52, P192, DOI 10.1038/s12276-020-0384-2

Pittet MJ, 2022, NAT REV CLIN ONCOL, V19, P402, DOI 10.1038/s41571-022-00620-6

Polyak K, 2009, TRENDS GENET, V25, P30, DOI 10.1016/j.tig.2008.10.012

Qian BZ, 2011, NATURE, V475, P222, DOI 10.1038/nature10138

Qian BZ, 2010, CELL, V141, P39, DOI 10.1016/j.cell.2010.03.014

Qian Y, 2017, ACS NANO, V11, P9536, DOI 10.1021/acsnano.7b05465

Quail DF, 2016, SCIENCE, V352, DOI 10.1126/science.aad3018

Quaranta V, 2019, CELLS-BASEL, V8, DOI 10.3390/cells8070747

Raskov H, 2021, FRONT ONCOL, V11, DOI 10.3389/fonc.2021.668731

Reinfeld BI, 2021, NATURE, V593, P282, DOI 10.1038/s41586-021-03442-1

Riabov V, 2014, FRONT PHYSIOL, V5, DOI 10.3389/fphys.2014.00075

Ridker PM, 2017, LANCET, V390, P1833, DOI 10.1016/S0140-6736(17)32247-X

Ries CH, 2015, CURR OPIN PHARMACOL, V23, P45, DOI 10.1016/j.coph.2015.05.008

Ringel AE, 2020, CELL, V183, P1848, DOI 10.1016/j.cell.2020.11.009

Ringelhan M, 2018, NAT IMMUNOL, V19, P222, DOI 10.1038/s41590-018-0044-z

Rogers TL, 2011, J TRANSL MED, V9, DOI 10.1186/1479-5876-9-177

Roh JS, 2018, IMMUNE NETW, V18, DOI 10.4110/in.2018.18.e27

Ross ERA, 2021, FRONT IMMUNOL, V12, DOI 10.3389/fimmu.2021.708186

Ruffell B, 2014, CANCER CELL, V26, P623, DOI 10.1016/j.ccell.2014.09.006

Rybakova Y, 2019, MOL THER, V27, P1415, DOI 10.1016/j.ymthe.2019.05.012

Salmon H, 2016, IMMUNITY, V44, P924, DOI 10.1016/j.immuni.2016.03.012

Salomon R, 2022, NAT CANCER, V3, P287, DOI 10.1038/s43018-022-00329-6

Salvagno C, 2019, NAT CELL BIOL, V21, P511, DOI 10.1038/s41556-019-0298-1

Savagner P, 2010, ANN ONCOL, V21, P89, DOI 10.1093/annonc/mdq292

Seki T, 2022, NATURE, V608, P421, DOI 10.1038/s41586-022-05030-3

Shi JW, 2021, INT J MOL SCI, V22, DOI 10.3390/ijms22062998

Shields CW, 2020, SCI ADV, V6, DOI 10.1126/sciadv.aaz6579

Shree T, 2011, GENE DEV, V25, P2465, DOI 10.1101/gad.180331.111

Silva JM, 2019, J CONTROL RELEASE, V298, P154, DOI 10.1016/j.jconrel.2019.02.006

Sloas C, 2021, FRONT IMMUNOL, V12, DOI 10.3389/fimmu.2021.783305

Stafford JH, 2016, NEURO-ONCOLOGY, V18, P797, DOI 10.1093/neuonc/nov272

Steeg PS, 2016, NAT REV CANCER, V16, P201, DOI 10.1038/nrc.2016.25

Stevens B, 2007, CELL, V131, P1164, DOI 10.1016/j.cell.2007.10.036

Strilic B, 2017, CANCER CELL, V32, P282, DOI 10.1016/j.ccell.2017.07.001

Su PF, 2022, CANCER CELL INT, V22, DOI 10.1186/s12935-022-02717-5

Su SC, 2018, CELL, V175, P442, DOI 10.1016/j.cell.2018.09.007

Sun W, 2017, BRIT J CANCER, V117, P1631, DOI 10.1038/bjc.2017.329

Takahashi K, 2005, J EXP MED, V201, P647, DOI 10.1084/jem.20041611

Tiwari JK, 2022, FRONT ONCOL, V11, DOI 10.3389/fonc.2021.793881

Tonnesen MG, 2000, J INVEST DERM SYMP P, V5, P40, DOI 10.1046/j.1087-0024.2000.00014.x

Torisu H, 2000, INT J CANCER, V85, P182, DOI 10.1002/(SICI)1097-0215(20000115)85:2<182::AID-IJC6>3.0.CO;2-M

Traba J, 2021, FRONT IMMUNOL, V12, DOI 10.3389/fimmu.2021.657293

Tran E, 2016, NEW ENGL J MED, V375, P2255, DOI 10.1056/NEJMoa1609279

Tsai CH, 2023, CELL METAB, V35, P118, DOI 10.1016/j.cmet.2022.12.003

Tu MY, 2021, NAT CANCER, V2, P1185, DOI 10.1038/s43018-021-00258-w

Varga T, 2016, IMMUNITY, V45, P1038, DOI 10.1016/j.immuni.2016.10.016

Vaught David B, 2015, Cancer Cell Microenviron, V2, DOI 10.14800/ccm.666

Venkatesan S, 2017, CSH PERSPECT MED, V7, DOI 10.1101/cshperspect.a026617

Voabil P, 2021, NAT MED, V27, P1250, DOI 10.1038/s41591-021-01398-3

Walens A, 2019, ELIFE, V8, DOI 10.7554/eLife.43653

Wang C, 2019, NATURE, V574, P268, DOI 10.1038/s41586-019-1607-3

Wang TT, 2014, FRONT IMMUNOL, V5, DOI 10.3389/fimmu.2014.00358

Wang Y, 2017, BIOMATERIALS, V112, P153, DOI 10.1016/j.biomaterials.2016.09.034

Weber M, 2021, INT J MOL SCI, V22, DOI 10.3390/ijms22031323

Willyard C, 2016, NATURE, V532, P166, DOI 10.1038/532166a

Xing F, 2018, CANCER RES, V78, P4316, DOI 10.1158/0008-5472.CAN-18-1102

Xun J, 2021, THERANOSTICS, V11, P6847, DOI 10.7150/thno.51864

Yamamoto K, 2020, CANCER SCI, V111, P1103, DOI 10.1111/cas.14328

Yang Q, 2021, FRONT IMMUNOL, V12, DOI 10.3389/fimmu.2021.756722

Yu JL, 2021, NAT MED, V27, P152, DOI 10.1038/s41591-020-1131-x

Zhang D, 2019, NATURE, V574, P575, DOI 10.1038/s41586-019-1678-1

Zhang JY, 2018, HEPATOLOGY, V67, P1872, DOI 10.1002/hep.29681

Zhang Q, 2021, FRONT IMMUNOL, V12, DOI 10.3389/fimmu.2021.702580

Zhang S, 2019, CELL METAB, V29, P443, DOI 10.1016/j.cmet.2018.12.004

Zhang YX, 2022, BMC CANCER, V22, DOI 10.1186/s12885-022-10431-8

Zhao HY, 2022, NAT METAB, V4, P1660, DOI 10.1038/s42255-022-00676-9

Zhao H, 2016, SCI REP-UK, V6, DOI 10.1038/srep29719

Zhou LS, 2022, FRONT IMMUNOL, V13, DOI 10.3389/fimmu.2022.848367

Zhou Y, 2020, IMMUNITY, V52, P357, DOI 10.1016/j.immuni.2020.01.014

Zhou Y, 2018, CANCER MED-US, V7, P4012, DOI 10.1002/cam4.1670

Zhu QY, 2016, ONCOL REP, V36, P3472, DOI 10.3892/or.2016.5136

Zhu WW, 2019, FASEB J, V33, P5208, DOI 10.1096/fj.201801791RR

Zhu Y, 2017, IMMUNITY, V47, P323, DOI 10.1016/j.immuni.2017.07.014

NR 209

TC 128

Z9 135

U1 39

U2 138

PU CELL PRESS

PI CAMBRIDGE

PA 50 HAMPSHIRE ST, FLOOR 5, CAMBRIDGE, MA 02139 USA

SN 0092-8674

EI 1097-4172

J9 CELL

JI Cell

PD APR 13

PY 2023

VL 186

IS 8

BP 1627

EP 1651

DI 10.1016/j.cell.2023.02.020

EA APR 2023

PG 25

WC Biochemistry & Molecular Biology; Cell Biology

WE Science Citation Index Expanded (SCI-EXPANDED)

SC Biochemistry & Molecular Biology; Cell Biology

GA F6DR4

UT WOS:000983234700001

PM 36924769

OA Bronze

HC Y

HP N

DA 2025-02-07

ER

PT J

AU Cotechini, T

Atallah, A

Grossman, A

AF Cotechini, Tiziana

Atallah, Aline

Grossman, Arielle

TI Tissue-Resident and Recruited Macrophages in Primary Tumor and

Metastatic Microenvironments: Potential Targets in Cancer Therapy

SO CELLS

LA English

DT Review

DE tumor-associated macrophages; tissue-resident macrophages; tumor

microenvironment; monocyte; metastasis-associated macrophage; trained

immunity; depletion; recruitment; repolarization; cancer; immune therapy

AB Macrophages within solid tumors and metastatic sites are heterogenous populations with different developmental origins and substantially contribute to tumor progression. A number of tumor-promoting phenotypes associated with both tumor- and metastasis-associated macrophages are similar to innate programs of embryonic-derived tissue-resident macrophages. In contrast to recruited macrophages originating from marrow precursors, tissue-resident macrophages are seeded before birth and function to coordinate tissue remodeling and maintain tissue integrity and homeostasis. Both recruited and tissue-resident macrophage populations contribute to tumor growth and metastasis and are important mediators of resistance to chemotherapy, radiation therapy, and immune checkpoint blockade. Thus, targeting various macrophage populations and their tumor-promoting phenotypes holds therapeutic promise. Here, we discuss various macrophage populations as regulators of tumor progression, immunity, and immunotherapy. We provide an overview of macrophage targeting strategies, including therapeutics designed to induce macrophage depletion, impair recruitment, and induce repolarization. We also provide a perspective on the therapeutic potential for macrophage-specific acquisition of trained immunity as an anti-cancer agent and discuss the therapeutic potential of exploiting macrophages and their traits to reduce tumor burden.

C1 [Cotechini, Tiziana; Atallah, Aline; Grossman, Arielle] Queens Univ, Dept Biomed & Mol Sci, Kingston, ON K7L 3N6, Canada.

C3 Queens University - Canada

RP Cotechini, T (corresponding author), Queens Univ, Dept Biomed & Mol Sci, Kingston, ON K7L 3N6, Canada.

EM tc28@queensu.ca; aline.atallah@queensu.ca; arielle.grossman@queensu.ca

RI Cotechini, Tiziana/JAN-3951-2023

OI Grossman, Arielle/0009-0002-3732-8926; Cotechini,

Tiziana/0000-0001-6762-3649; Atallah, Aline/0000-0001-7256-9632

CR Aegerter H, 2020, NAT IMMUNOL, V21, P145, DOI 10.1038/s41590-019-0568-x

Alderson KL, 2013, CANCER IMMUNOL IMMUN, V62, P665, DOI 10.1007/s00262-012-1372-8

Allavena P, 2005, CANCER RES, V65, P2964, DOI 10.1158/0008-5472.CAN-04-4037

Anderson NM, 2020, CURR BIOL, V30, pR921, DOI 10.1016/j.cub.2020.06.081

Andon FT, 2017, SEMIN IMMUNOL, V34, P103, DOI 10.1016/j.smim.2017.09.004

Andreu P, 2010, CANCER CELL, V17, P121, DOI 10.1016/j.ccr.2009.12.019

Anfray C, 2020, CELLS-BASEL, V9, DOI 10.3390/cells9010046

Angell HK, 2020, CLIN CANCER RES, V26, P332, DOI 10.1158/1078-0432.CCR-18-1851

Antunes ARP, 2021, NAT NEUROSCI, V24, P595, DOI 10.1038/s41593-020-00789-y

Anwar MA, 2019, MED RES REV, V39, P1053, DOI 10.1002/med.21553

Ao JY, 2017, MOL CANCER THER, V16, P1544, DOI 10.1158/1535-7163.MCT-16-0866

Aran D, 2017, GENOME BIOL, V18, DOI 10.1186/s13059-017-1349-1

Argyle D, 2018, FRONT IMMUNOL, V9, DOI 10.3389/fimmu.2018.02629

Arlauckas SP, 2017, SCI TRANSL MED, V9, DOI 10.1126/scitranslmed.aal3604

Bain CC, 2014, NAT IMMUNOL, V15, P929, DOI 10.1038/ni.2967

Balhara J, 2012, MUCOSAL IMMUNOL, V5, P605, DOI 10.1038/mi.2012.74

Balkwill FR, 2012, J CELL SCI, V125, P5591, DOI 10.1242/jcs.116392

Balmanno K, 2009, CELL DEATH DIFFER, V16, P368, DOI 10.1038/cdd.2008.148

Banaei-Bouchareb L, 2004, J LEUKOCYTE BIOL, V76, P359, DOI 10.1189/jlb.1103591

Beatty GL, 2011, SCIENCE, V331, P1612, DOI 10.1126/science.1198443

Ben-Mordechai T, 2019, INT J RADIAT ONCOL, V105, pS125, DOI 10.1016/j.ijrobp.2019.06.103

Bertrand JY, 2005, BLOOD, V106, P3004, DOI 10.1182/blood-2005-02-0461

BIEWENGA J, 1995, CELL TISSUE RES, V280, P189, DOI 10.1007/BF00304524

Blok BA, 2015, J LEUKOCYTE BIOL, V98, P347, DOI 10.1189/jlb.5RI0315-096R

Blumenthal RL, 2001, J ALLERGY CLIN IMMUN, V107, P258, DOI 10.1067/mai.2001.112845

Bolli E, 2019, J CONTROL RELEASE, V314, P1, DOI 10.1016/j.jconrel.2019.10.024

Boulakirba S, 2018, SCI REP-UK, V8, DOI 10.1038/s41598-017-18433-4

Buffen K, 2014, PLOS PATHOG, V10, DOI 10.1371/journal.ppat.1004485

Bulle A, 2020, TRANSL ONCOL, V13, DOI 10.1016/j.tranon.2020.01.004

Burgess Matthew, 2019, Immunohorizons, V3, P262, DOI 10.4049/immunohorizons.1900038

Cailhier JF, 2005, J IMMUNOL, V174, P2336, DOI 10.4049/jimmunol.174.4.2336

Cannarile MA, 2017, J IMMUNOTHER CANCER, V5, DOI 10.1186/s40425-017-0257-y

Carron EC, 2017, ONCOTARGET, V8, P50731, DOI 10.18632/oncotarget.14913

Caslini C, 2019, ONCOGENE, V38, P6599, DOI 10.1038/s41388-019-0897-0

Cassetta L, 2019, CANCER CELL, V35, P588, DOI 10.1016/j.ccell.2019.02.009

Cassetta L, 2018, FRONT CELL DEV BIOL, V6, DOI 10.3389/fcell.2018.00038

Castellaro AM, 2019, CANCERS, V11, DOI 10.3390/cancers11020189

Celus W, 2017, CELL REP, V21, P2842, DOI 10.1016/j.celrep.2017.11.034

Chen Q, 2011, CANCER CELL, V20, P538, DOI 10.1016/j.ccr.2011.08.025

Chen X, 2020, INT J RADIAT ONCOL, V108, pE567

Chevrier S, 2017, CELL, V169, P736, DOI 10.1016/j.cell.2017.04.016

Chihara T, 2010, CELL DEATH DIFFER, V17, P1917, DOI 10.1038/cdd.2010.60

Choi J, 2012, BIOMATERIALS, V33, P4195, DOI 10.1016/j.biomaterials.2012.02.022

Choi Mi-Ran, 2012, Cancer Nanotechnol, V3, P47, DOI 10.1007/s12645-012-0029-9

Dai XM, 2002, BLOOD, V99, P111, DOI 10.1182/blood.V99.1.111

Davies LC, 2013, NAT IMMUNOL, V14, P986, DOI 10.1038/ni.2705

De Schepper S, 2018, CELL, V175, P400, DOI 10.1016/j.cell.2018.07.048

DeNardo DG, 2019, NAT REV IMMUNOL, V19, P369, DOI 10.1038/s41577-019-0127-6

DeNardo DG, 2011, CANCER DISCOV, V1, P54, DOI 10.1158/2159-8274.CD-10-0028

DeNardo DG, 2009, CANCER CELL, V16, P91, DOI 10.1016/j.ccr.2009.06.018

Doedens AL, 2010, CANCER RES, V70, P7465, DOI 10.1158/0008-5472.CAN-10-1439

Donadon M, 2020, J EXP MED, V217, DOI 10.1084/jem.20191847

Downey CM, 2014, PLOS ONE, V9, DOI 10.1371/journal.pone.0099988

Emami F, 2021, J CONTROL RELEASE, V329, P645, DOI 10.1016/j.jconrel.2020.10.001

Eng J, 2020, METHODS MOL BIOL, V2055, P521, DOI 10.1007/978-1-4939-9773-2_24

Etzerodt A, 2020, J EXP MED, V217, DOI 10.1084/jem.20191869

Fang WB, 2016, ONCOTARGET, V7, P49349, DOI 10.18632/oncotarget.9885

Feng QY, 2019, CLIN CANCER RES, V25, P3896, DOI 10.1158/1078-0432.CCR-18-2076

Feng YH, 2011, CANCER BIOL THER, V11, P111, DOI 10.4161/cbt.11.1.13965

Fritz JM, 2014, FRONT IMMUNOL, V5, DOI 10.3389/fimmu.2014.00587

Fu JJ, 2015, J CONTROL RELEASE, V204, P11, DOI 10.1016/j.jconrel.2015.01.039

Gadkaree SK, 2017, HEAD NECK-J SCI SPEC, V39, P1086, DOI 10.1002/hed.24704

Galluzzi L, 2017, NAT REV IMMUNOL, V17, P97, DOI 10.1038/nri.2016.107

Galon J, 2020, Q J NUCL MED MOL IM, V64, P152, DOI 10.23736/S1824-4785.20.03249-5

Gast CE, 2018, SCI ADV, V4, DOI 10.1126/sciadv.aat7828

Gautier EL, 2012, NAT IMMUNOL, V13, P1118, DOI 10.1038/ni.2419

Georgoudaki AM, 2016, CELL REP, V15, P2000, DOI 10.1016/j.celrep.2016.04.084

Germano G, 2013, CANCER CELL, V23, P249, DOI 10.1016/j.ccr.2013.01.008

Giesen C, 2014, NAT METHODS, V11, P417, DOI [10.1038/NMETH.2869, 10.1038/nmeth.2869]

Gordon EM, 2016, ADV THER, V33, P1055, DOI 10.1007/s12325-016-0344-3

Gordon S, 2003, NAT REV IMMUNOL, V3, P23, DOI 10.1038/nri978

Goswami S, 2005, CANCER RES, V65, P5278, DOI 10.1158/0008-5472.CAN-04-1853

Gouon-Evans V, 2000, DEVELOPMENT, V127, P2269

Graham CH, 2021, CUAJ-CAN UROL ASSOC, V15, pE412, DOI 10.5489/cuaj.7066

Guan W, 2019, ENDOCR-RELAT CANCER, V26, P131, DOI 10.1530/ERC-18-0284

Guerriero JL, 2017, NATURE, V543, P428, DOI 10.1038/nature21409

Guilliams M, 2021, NAT IMMUNOL, V22, P118, DOI 10.1038/s41590-020-00849-2

Guilliams M, 2013, J EXP MED, V210, P1977, DOI 10.1084/jem.20131199

Gunderson AJ, 2016, CANCER DISCOV, V6, P270, DOI 10.1158/2159-8290.CD-15-0827

Guo F, 2020, DIS MARKERS, V2020, DOI 10.1155/2020/1805764

Hamilton MJ, 2010, J IMMUNOL, V185, P4545, DOI 10.4049/jimmunol.1002045

Hanada T, 2000, INT J UROL, V7, P263, DOI 10.1046/j.1442-2042.2000.00190.x

Harney AS, 2015, CANCER DISCOV, V5, P932, DOI 10.1158/2159-8290.CD-15-0012

Hartley GP, 2018, CANCER IMMUNOL RES, V6, P1260, DOI 10.1158/2326-6066.CIR-17-0537

Hashimoto D, 2013, IMMUNITY, V38, P792, DOI 10.1016/j.immuni.2013.04.004

HOLT PG, 1993, J EXP MED, V177, P397, DOI 10.1084/jem.177.2.397

Hughes R, 2015, CANCER RES, V75, P3479, DOI 10.1158/0008-5472.CAN-14-3587

Italiani P, 2014, FRONT IMMUNOL, V5, DOI 10.3389/fimmu.2014.00514

Jakubzick C, 2006, J IMMUNOL, V176, P3578, DOI 10.4049/jimmunol.176.6.3578

Jakubzick CV, 2017, NAT REV IMMUNOL, V17, P349, DOI 10.1038/nri.2017.28

Jäppinen N, 2019, NAT COMMUN, V10, DOI 10.1038/s41467-018-08065-1

Jayasingam SD, 2020, FRONT ONCOL, V9, DOI 10.3389/fonc.2019.01512

Jenkins SJ, 2011, SCIENCE, V332, P1284, DOI 10.1126/science.1204351

Jones KI, 2018, EMBO MOL MED, V10, DOI 10.15252/emmm.201809342

Kalafati L, 2020, CELL, V183, P771, DOI 10.1016/j.cell.2020.09.058

Kalbasi A, 2017, CLIN CANCER RES, V23, P137, DOI 10.1158/1078-0432.CCR-16-0870

Kamat AM, 2016, EUR UROL, V69, P197, DOI 10.1016/j.eururo.2015.06.023

Kaneda MM, 2016, NATURE, V539, P437, DOI 10.1038/nature19834

Kaneda MM, 2016, CANCER DISCOV, V6, P870, DOI 10.1158/2159-8290.CD-15-1346

Kitamura T, 2018, FRONT IMMUNOL, V8, DOI 10.3389/fimmu.2017.02004

Kitamura T, 2015, J EXP MED, V212, P1043, DOI 10.1084/jem.20141836

Klichinsky M, 2020, NAT BIOTECHNOL, V38, P947, DOI 10.1038/s41587-020-0462-y

Kopf M, 2015, NAT IMMUNOL, V16, P36, DOI 10.1038/ni.3052

Kosaka A, 2014, CANCER IMMUNOL IMMUN, V63, P847, DOI 10.1007/s00262-014-1561-8

Kowal J, 2019, IMMUNOTHERAPY-UK, V11, P677, DOI 10.2217/imt-2018-0156

Kratofil RM, 2017, ARTERIOSCL THROM VAS, V37, P35, DOI 10.1161/ATVBAHA.116.308198

KUNG JT, 1977, J EXP MED, V146, P665, DOI 10.1084/jem.146.3.665

La Fleur L, 2021, CANCER RES, V81, P956, DOI 10.1158/0008-5472.CAN-20-1885

Li J, 2018, ONCOL LETT, V16, P5575, DOI 10.3892/ol.2018.9368

Li P, 2021, CHEM ENG J, V409, DOI 10.1016/j.cej.2020.128217

Lichanska AM, 2000, EXP HEMATOL, V28, P601, DOI 10.1016/S0301-472X(00)00157-0

Lim SY, 2016, ONCOTARGET, V7, P28697, DOI 10.18632/oncotarget.7376

Lin HS, 2008, SCIENCE, V320, P807, DOI 10.1126/science.1154370

Linde N, 2018, NAT COMMUN, V9, DOI 10.1038/s41467-017-02481-5

Liu M, 2018, CELL PHYSIOL BIOCHEM, V51, P1679, DOI 10.1159/000495673

Liu M, 2015, J IMMUNOL, V195, P5055, DOI 10.4049/jimmunol.1501158

Liu W, 2020, CANCER LETT, V472, P59, DOI 10.1016/j.canlet.2019.12.024

Liu ZY, 2019, CELL, V178, P1509, DOI 10.1016/j.cell.2019.08.009

Liudahl SM, 2021, CANCER DISCOV, V11, P2014, DOI 10.1158/2159-8290.CD-20-0841

Lizotte PH, 2014, ONCOIMMUNOLOGY, V3, DOI 10.4161/onci.28926

Lopez JP, 2014, CURR ONCOL REP, V16, DOI 10.1007/s11912-014-0387-z

Loyher PL, 2018, J EXP MED, V215, P2536, DOI 10.1084/jem.20180534

Luke JJ, 2019, CLIN CANCER RES, V25, P3074, DOI 10.1158/1078-0432.CCR-18-1942

Ma RY, 2020, J EXP MED, V217, DOI 10.1084/jem.20191820

Macciò A, 2020, SCI REP-UK, V10, DOI 10.1038/s41598-020-63276-1

MacDonald KPA, 2010, BLOOD, V116, P3955, DOI 10.1182/blood-2010-02-266296

Mansoori B, 2017, ADV PHARM BULL, V7, P339, DOI 10.15171/apb.2017.041

Mantovani A, 2002, TRENDS IMMUNOL, V23, P549, DOI 10.1016/S1471-4906(02)02302-5

Marofi F, 2021, STEM CELL RES THER, V12, DOI 10.1186/s13287-020-02128-1

Martinez F.O., 2014, F1000PRIME REP, V6, P12703, DOI [DOI 10.12703/P6-13, 10.12703/P6-13]

Matlack R, 2006, IMMUNOLOGY, V117, P386, DOI 10.1111/j.1365-2567.2005.02312.x

Meads MB, 2009, NAT REV CANCER, V9, P665, DOI 10.1038/nrc2714

Medler TR, 2018, CANCER CELL, V34, P561, DOI 10.1016/j.ccell.2018.09.003

Meziani L, 2018, ONCOIMMUNOLOGY, V7, DOI 10.1080/2162402X.2018.1494488

Mills CD, 2000, J IMMUNOL, V164, P6166, DOI 10.4049/jimmunol.164.12.6166

Mitchem JB, 2013, CANCER RES, V73, P1128, DOI 10.1158/0008-5472.CAN-12-2731

Molawi K, 2014, J EXP MED, V211, P2151, DOI 10.1084/jem.20140639

MORALES A, 1976, J UROLOGY, V116, P180, DOI 10.1016/S0022-5347(17)58737-6

Movahedi K, 2010, CANCER RES, V70, P5728, DOI 10.1158/0008-5472.CAN-09-4672

Mu XJ, 2021, SHOCK, V55, P14, DOI 10.1097/SHK.0000000000001601

Mullins SR, 2019, J IMMUNOTHER CANCER, V7, DOI 10.1186/s40425-019-0724-8

Munn DH, 2003, CURR PHARM DESIGN, V9, P257, DOI 10.2174/1381612033392026

Munn DH, 1999, J EXP MED, V189, P1363, DOI 10.1084/jem.189.9.1363

Murray PJ, 2014, IMMUNITY, V41, P14, DOI 10.1016/j.immuni.2014.06.008

Nagai T, 2009, CANCER IMMUNOL IMMUN, V58, P1577, DOI 10.1007/s00262-009-0667-x

Netea MG, 2020, NAT REV IMMUNOL, V20, P375, DOI 10.1038/s41577-020-0285-6

Netea MG, 2016, SCIENCE, V352, DOI 10.1126/science.aaf1098

Neubert NJ, 2018, SCI TRANSL MED, V10, DOI 10.1126/scitranslmed.aan3311

Neyen C, 2013, J IMMUNOL, V190, P3798, DOI 10.4049/jimmunol.1203194

Nielsen SR, 2016, NAT CELL BIOL, V18, P549, DOI 10.1038/ncb3340

Noy R, 2014, IMMUNITY, V41, P49, DOI 10.1016/j.immuni.2014.06.010

Nywening TM, 2016, LANCET ONCOL, V17, P651, DOI 10.1016/S1470-2045(16)00078-4

O'Brien SA, 2021, CANCER IMMUNOL IMMUN, V70, P2401, DOI 10.1007/s00262-021-02861-3

Olsson M, 2011, CELL DEATH DIFFER, V18, P1441, DOI 10.1038/cdd.2011.30

Oshi M, 2020, SCI REP-UK, V10, DOI 10.1038/s41598-020-73624-w

Packiam VT, 2017, CANCER-AM CANCER SOC, V123, P390, DOI 10.1002/cncr.30392

PASSLICK B, 1989, BLOOD, V74, P2527, DOI 10.1182/blood.V74.7.2527.2527

Patel S, 2019, FRONT ONCOL, V9, DOI 10.3389/fonc.2019.00196

Pawelek J, 2006, CONTRIB MICROBIOL, V13, P138, DOI 10.1159/000092970

Peranzoni E, 2018, P NATL ACAD SCI USA, V115, pE4041, DOI 10.1073/pnas.1720948115

Perdiguero EG, 2016, NAT IMMUNOL, V17, P2, DOI 10.1038/ni.3341

Perdiguero EG, 2015, NATURE, V518, P547, DOI 10.1038/nature13989

Pienta KJ, 2013, INVEST NEW DRUG, V31, P760, DOI 10.1007/s10637-012-9869-8

Poh AR, 2018, FRONT ONCOL, V8, DOI 10.3389/fonc.2018.00049

Pollard J.W.S., 1996, Adv Develop Bioch, V4, P153

Pollard JW, 2009, NAT REV IMMUNOL, V9, P259, DOI 10.1038/nri2528

POLLARD JW, 1994, P NATL ACAD SCI USA, V91, P9312, DOI 10.1073/pnas.91.20.9312

Priceman SJ, 2010, BLOOD, V115, P1461, DOI 10.1182/blood-2009-08-237412

Priem B, 2020, CELL, V183, P786, DOI 10.1016/j.cell.2020.09.059

Puig-Kröger A, 2009, CANCER RES, V69, P9395, DOI 10.1158/0008-5472.CAN-09-2050

Pulaski HL, 2009, J TRANSL MED, V7, DOI 10.1186/1479-5876-7-49

Pyonteck SM, 2013, NAT MED, V19, P1264, DOI 10.1038/nm.3337

Qi Y, 2021, MATER DESIGN, V198, DOI 10.1016/j.matdes.2020.109388

Qian BZ, 2015, J EXP MED, V212, P1433, DOI 10.1084/jem.20141555

Qian BZ, 2011, NATURE, V475, P222, DOI 10.1038/nature10138

Qian BZ, 2010, CELL, V141, P39, DOI 10.1016/j.cell.2010.03.014

Qian BZ, 2009, PLOS ONE, V4, DOI 10.1371/journal.pone.0006562

Qiang L, 2019, J NANOBIOTECHNOL, V17, DOI 10.1186/s12951-019-0513-z

Qiao JH, 2020, BIOCHEM PHARMACOL, V180, DOI 10.1016/j.bcp.2020.114126

Quail DF, 2016, SCIENCE, V352, DOI 10.1126/science.aad3018

Quaranta V, 2018, CANCER RES, V78, P4253, DOI 10.1158/0008-5472.CAN-17-3876

Ramesh A, 2019, ADV MATER, V31, DOI 10.1002/adma.201904364

Rodell CB, 2018, NAT BIOMED ENG, V2, P578, DOI 10.1038/s41551-018-0236-8

Rogers TL, 2011, J TRANSL MED, V9, DOI 10.1186/1479-5876-9-177

Ruffell B, 2015, CANCER CELL, V27, P462, DOI 10.1016/j.ccell.2015.02.015

Ruffell B, 2014, CANCER CELL, V26, P623, DOI 10.1016/j.ccell.2014.09.006

Ruffell B, 2012, TRENDS IMMUNOL, V33, P119, DOI 10.1016/j.it.2011.12.001

Ruffell B, 2012, P NATL ACAD SCI USA, V109, P2796, DOI 10.1073/pnas.1104303108

Saeed S, 2014, SCIENCE, V345, P1578, DOI 10.1126/science.1251086

Sarode P, 2020, SCI ADV, V6, DOI 10.1126/sciadv.aaz6105

Sawa-Wejksza K, 2018, ARCH IMMUNOL THER EX, V66, P97, DOI 10.1007/s00005-017-0480-8

Schuette V, 2016, P NATL ACAD SCI USA, V113, P10649, DOI 10.1073/pnas.1605885113

Schulz C, 2012, SCIENCE, V336, P86, DOI 10.1126/science.1219179

Scott CL, 2016, NAT COMMUN, V7, DOI 10.1038/ncomms10321

Scott EM, 2019, J IMMUNOTHER CANCER, V7, DOI 10.1186/s40425-019-0807-6

Shan X, 2020, CLIN IMMUNOL, V216, DOI 10.1016/j.clim.2020.108430

Shen JY, 2015, ONCOTARGET, V6, P14700, DOI 10.18632/oncotarget.3739

Sheng JP, 2015, IMMUNITY, V43, P382, DOI 10.1016/j.immuni.2015.07.016

Shi JJ, 2017, NAT REV CANCER, V17, P20, DOI 10.1038/nrc.2016.108

Shiao SL, 2015, CANCER IMMUNOL RES, V3, P518, DOI 10.1158/2326-6066.CIR-14-0232

Sica A, 2012, J CLIN INVEST, V122, P787, DOI 10.1172/JCI59643

Song E, 2017, J IMMUNOL, V198

Soroosh P, 2013, J EXP MED, V210, P775, DOI 10.1084/jem.20121849

STANLEY E, 1994, P NATL ACAD SCI USA, V91, P5592, DOI 10.1073/pnas.91.12.5592

Stanley ER, 2014, CSH PERSPECT BIOL, V6, DOI 10.1101/cshperspect.a021857

Strachan DC, 2013, ONCOIMMUNOLOGY, V2, DOI 10.4161/onci.26968

Su YX, 2015, ACS BIOMATER SCI ENG, V1, P201, DOI 10.1021/ab500179h

Sunderkötter C, 2004, J IMMUNOL, V172, P4410, DOI 10.4049/jimmunol.172.7.4410

Szczerba BM, 2019, NATURE, V566, P553, DOI 10.1038/s41586-019-0915-y

T'Jonck W, 2018, CELL IMMUNOL, V330, P43, DOI 10.1016/j.cellimm.2018.02.005

Takahashi R, 2020, SCI REP-UK, V10, DOI 10.1038/s41598-020-78320-3

Tan SYS, 2016, DEVELOPMENT, V143, P1318, DOI 10.1242/dev.129122

Taylor PR, 2005, ANNU REV IMMUNOL, V23, P901, DOI 10.1146/annurev.immunol.23.021704.115816

Tham M, 2015, ONCOTARGET, V6, P22857, DOI 10.18632/oncotarget.3127

Tsujikawa T, 2017, CELL REP, V19, P203, DOI 10.1016/j.celrep.2017.03.037

Tu MM, 2020, COMMUN BIOL, V3, DOI 10.1038/s42003-020-01441-y

Tuit S, 2019, CELL REP, V29, P1221, DOI 10.1016/j.celrep.2019.09.067

Valastyan S, 2011, CELL, V147, P275, DOI 10.1016/j.cell.2011.09.024

Valls AF, 2019, CLIN CANCER RES, V25, P5674, DOI 10.1158/1078-0432.CCR-18-2123

Van Acker HH, 2016, PHARMACOL THERAPEUT, V158, P24, DOI 10.1016/j.pharmthera.2015.11.008

van Dalen FJ, 2019, MOLECULES, V24, DOI 10.3390/molecules24010009

van der Heijden CDCC, 2018, ANTIOXID REDOX SIGN, V29, P1023, DOI 10.1089/ars.2017.7310

Van Hove H, 2019, NAT NEUROSCI, V22, P1021, DOI 10.1038/s41593-019-0393-4

VANTWOUT JW, 1992, SCAND J IMMUNOL, V36, P713

Vidyarthi A, 2018, FRONT IMMUNOL, V9, DOI 10.3389/fimmu.2018.01650

Vonderheide RH, 2013, CANCER IMMUNOL IMMUN, V62, P949, DOI 10.1007/s00262-013-1427-5

Vonderheide RH, 2013, CLIN CANCER RES, V19, P1035, DOI 10.1158/1078-0432.CCR-12-2064

Wagner J, 2019, CELL, V177, P1330, DOI 10.1016/j.cell.2019.03.005

Wang Y, 2017, BIOMATERIALS, V112, P153, DOI 10.1016/j.biomaterials.2016.09.034

Wang Y, 2020, ELIFE, V9, DOI 10.7554/eLife.57438

Weizman N, 2014, ONCOGENE, V33, P3812, DOI 10.1038/onc.2013.357

Wesley JD, 2012, HUM VACC IMMUNOTHER, V8, P520, DOI 10.4161/hv.18769

Williams JW, 2018, J AM COLL CARDIOL, V72, P2166, DOI 10.1016/j.jacc.2018.08.2148

Wu K, 2009, CANCER RES, V69, P8067, DOI 10.1158/0008-5472.CAN-09-0901

Wu T, 2016, ONCOTARGET, V7, P19548, DOI 10.18632/oncotarget.6969

Wyckoff J, 2004, CANCER RES, V64, P7022, DOI 10.1158/0008-5472.CAN-04-1449

Xia AL, 2017, ONCOTARGET, V8, P90521, DOI 10.18632/oncotarget.19361

Xin HB, 2021, HPB, V23, P1061, DOI 10.1016/j.hpb.2020.11.002

Xu GL, 2020, ONCOGENE, V39, P1429, DOI 10.1038/s41388-019-1072-3

Xu XJ, 2019, TOXICOL APPL PHARM, V364, P77, DOI 10.1016/j.taap.2018.12.013

Yan D, 2017, ONCOGENE, V36, P6049, DOI 10.1038/onc.2017.261

Yeung CLA, 2016, NAT COMMUN, V7, DOI 10.1038/ncomms11150

Yin SC, 2017, PLOS ONE, V12, DOI 10.1371/journal.pone.0170042

Zanganeh S, 2016, NAT NANOTECHNOL, V11, P986, DOI [10.1038/NNANO.2016.168, 10.1038/nnano.2016.168]

Zeisberger SM, 2006, BRIT J CANCER, V95, P272, DOI 10.1038/sj.bjc.6603240

Zhan XD, 2014, BIOMATERIALS, V35, P10046, DOI 10.1016/j.biomaterials.2014.09.007

Zhang QW, 2012, PLOS ONE, V7, DOI 10.1371/journal.pone.0050946

Zhang W, 2010, CLIN CANCER RES, V16, P3420, DOI 10.1158/1078-0432.CCR-09-2904

Zhang WL, 2019, BRIT J CANCER, V121, P837, DOI 10.1038/s41416-019-0578-3

Zhang X, 2016, CANCER LETT, V381, P305, DOI 10.1016/j.canlet.2016.08.004

Zheng PM, 2017, J EXP CLIN CANC RES, V36, DOI 10.1186/s13046-017-0528-y

Zhou Y, 2020, ONCOL LETT, V20, DOI 10.3892/ol.2020.12161

Zhu Y, 2017, IMMUNITY, V47, P597, DOI 10.1016/j.immuni.2017.08.018

Zhu Y, 2014, CANCER RES, V74, P5057, DOI 10.1158/0008-5472.CAN-13-3723

Zilionis R, 2019, IMMUNITY, V50, P1317, DOI 10.1016/j.immuni.2019.03.009

Zitvogel L, 2008, NAT REV IMMUNOL, V8, P59, DOI 10.1038/nri2216

Zwar TA, 2006, J IMMUNOL, V176, P1609, DOI 10.4049/jimmunol.176.3.1609

NR 257

TC 37

Z9 42

U1 2

U2 41

PU MDPI

PI BASEL

PA ST ALBAN-ANLAGE 66, CH-4052 BASEL, SWITZERLAND

EI 2073-4409

J9 CELLS-BASEL

JI Cells

PD APR

PY 2021

VL 10

IS 4

AR 960

DI 10.3390/cells10040960

PG 31

WC Cell Biology

WE Science Citation Index Expanded (SCI-EXPANDED)

SC Cell Biology

GA RR1KX

UT WOS:000642866800001

PM 33924237

OA Green Published, gold

DA 2025-02-07

ER

PT J

AU Ammarah, U

Pereira-Nunes, A

Delfini, M

Mazzone, M

AF Ammarah, Ummi

Pereira-Nunes, Andreia

Delfini, Marcello

Mazzone, Massimiliano

TI From monocyte-derived macrophages to resident macrophages-how metabolism

leads their way in cancer

SO MOLECULAR ONCOLOGY

LA English

DT Review

DE cancer metabolism; immunometabolism; monocyte-derived macrophages;

tissue-resident macrophages; tumor microenvironment; tumor-associated

macrophages

ID TUMOR-ASSOCIATED MACROPHAGES; BREAST-CANCER; TISSUE-RESIDENT;

INTERSTITIAL MACROPHAGES; MIXED-POLARIZATION; OVARIAN-CARCINOMA;

NITRIC-OXIDE; CELLS; LUNG; EXPRESSION

AB Macrophages are innate immune cells that play key roles during both homeostasis and disease. Depending on the microenvironmental cues sensed in different tissues, macrophages are known to acquire specific phenotypes and exhibit unique features that, ultimately, orchestrate tissue homeostasis, defense, and repair. Within the tumor microenvironment, macrophages are referred to as tumor-associated macrophages (TAMs) and constitute a heterogeneous population. Like their tissue resident counterpart, TAMs are plastic and can switch function and phenotype according to the niche-derived stimuli sensed. While changes in TAM phenotype are known to be accompanied by adaptive alterations in their cell metabolism, it is reported that metabolic reprogramming of macrophages can dictate their activation state and function. In line with these observations, recent research efforts have been focused on defining the metabolic traits of TAM subsets in different tumor malignancies and understanding their role in cancer progression and metastasis formation. This knowledge will pave the way to novel therapeutic strategies tailored to cancer subtype-specific metabolic landscapes. This review outlines the metabolic characteristics of distinct TAM subsets and their implications in tumorigenesis across multiple cancer types.

Tumor-associated macrophages (TAMs) constitute up to 50% of the tumor mass, representing a heterogeneous population of tissue-resident and monocyte-derived macrophages. TAM phenotype not only involves alterations in cell metabolism but also metabolic reprogramming that can dictate their activation state and function. This review elucidates the diverse roles and metabolic traits of distinct TAM subsets in pancreatic, breast, lung and ovarian malignancies. image

C1 [Ammarah, Ummi; Pereira-Nunes, Andreia; Delfini, Marcello; Mazzone, Massimiliano] VIB, Lab Tumor Inflammat & Angiogenesis, Ctr Canc Biol, Leuven, Belgium.

[Ammarah, Ummi; Pereira-Nunes, Andreia; Delfini, Marcello; Mazzone, Massimiliano] Katholieke Univ Leuven, Ctr Canc Biol, Dept Oncol, Lab Tumor Inflammat & Angiogenesis, Leuven, Belgium.

[Ammarah, Ummi] Univ Torino, Mol Biotechnol Ctr, Dept Mol Biotechnol & Hlth Sci, Turin, Italy.

[Pereira-Nunes, Andreia] Univ Minho, Life & Hlth Sci Res Inst ICVS, Sch Med, Braga, Portugal.

[Pereira-Nunes, Andreia] ICVS 3Bs PT Govt Associate Lab, Braga Guimaraes, Portugal.

[Mazzone, Massimiliano] Univ Leuven VIB, Ctr Canc Biol, Dept Oncol, Lab Tumor Inflammat & Angiogenesis, CampusGasthuisberg,Herestr 49,Box 912, B-3000 Leuven, Belgium.

C3 Flanders Institute for Biotechnology (VIB); KU Leuven; University of

Turin; Universidade do Minho; KU Leuven; Flanders Institute for

Biotechnology (VIB)

RP Mazzone, M (corresponding author), Univ Leuven VIB, Ctr Canc Biol, Dept Oncol, Lab Tumor Inflammat & Angiogenesis, CampusGasthuisberg,Herestr 49,Box 912, B-3000 Leuven, Belgium.

EM massimiliano.mazzone@kuleuven.be

RI Delfini, Marcello/KEH-5721-2024

OI Pereira Nunes, Andreia/0000-0002-7890-9228; Mazzone,

Massimiliano/0000-0001-8824-4015

FU European Research Council consolidator grant; FWO research grant

[G011124N]; Fundaco para a Ciencia e a Tecnologia [SFRH/BD/148476/2019]

FX MM was supported by an European Research Council consolidator grant

(Immunofit), and an FWO research grant (G011124N). AP-N received

fellowship from Fundac & atilde;o para a Ciencia e a Tecnologia (ref.

SFRH/BD/148476/2019). The authors acknowledge the use of Biorender for

creating figures.

CR Adhikary T, 2017, BMC GENOMICS, V18, DOI 10.1186/s12864-017-3630-9

Bailey JD, 2019, CELL REP, V28, P218, DOI 10.1016/j.celrep.2019.06.018

Bedoret D, 2009, J CLIN INVEST, V119, P3723, DOI 10.1172/JCI39717

Bellora F, 2014, EUR J IMMUNOL, V44, P1814, DOI 10.1002/eji.201344130

Bharat A, 2016, AM J RESP CELL MOL, V54, P147, DOI 10.1165/rcmb.2015-0147LE

Bi JL, 2021, J TRANSL MED, V19, DOI 10.1186/s12967-021-03057-0

Binnewies M, 2018, NAT MED, V24, P541, DOI 10.1038/s41591-018-0014-x

Biswas SK, 2015, IMMUNITY, V43, P435, DOI 10.1016/j.immuni.2015.09.001

Bleriot C, 2020, IMMUNITY, V52, P957, DOI 10.1016/j.immuni.2020.05.014

Bonapace L, 2014, NATURE, V515, DOI 10.1038/nature13862

Browning L, 2018, CANCER MANAG RES, V10, P6685, DOI 10.2147/CMAR.S179189

Brummer G, 2018, MOL CANCER RES, V16, P296, DOI 10.1158/1541-7786.MCR-17-0308

Bulle A, 2020, SIGNAL TRANSDUCT TAR, V5, DOI 10.1038/s41392-020-00341-1

Calderon B, 2015, J EXP MED, V212, P1497, DOI 10.1084/jem.20150496

Cannon Andrew, 2018, Genes Cancer, V9, P78, DOI 10.18632/genesandcancer.171

Caputa G, 2019, NAT IMMUNOL, V20, P793, DOI 10.1038/s41590-019-0407-0

Caronni N, 2023, NATURE, V623, P415, DOI 10.1038/s41586-023-06685-2

Casanova-Acebes M, 2021, NATURE, V595, P578, DOI 10.1038/s41586-021-03651-8

Casanova-Acebes M, 2020, NAT COMMUN, V11, DOI 10.1038/s41467-020-15371-0

Cassetta L, 2018, NAT REV DRUG DISCOV, V17, P887, DOI 10.1038/nrd.2018.169

Chang CY, 2022, SCI ADV, V8, DOI 10.1126/sciadv.abq0615

Chen PW, 2017, P NATL ACAD SCI USA, V114, P580, DOI 10.1073/pnas.1614035114

Chen X, 2018, CANCER LETT, V435, P80, DOI 10.1016/j.canlet.2018.08.001

Chen X, 2017, ONCOL REP, V38, P522, DOI 10.3892/or.2017.5697

Chen Yi, 2019, Jishengchong Yu Yixue Kunchong Xuebao, V26, P1, DOI 10.3969/j.issn.1005-0507.2019.01.001

Cheng WY, 2016, ELIFE, V5, DOI 10.7554/eLife.18501

Christofides A, 2022, NAT IMMUNOL, V23, P1148, DOI 10.1038/s41590-022-01267-2

CLEETER MWJ, 1994, FEBS LETT, V345, P50, DOI 10.1016/0014-5793(94)00424-2

Conway EM, 2016, AM J RESP CRIT CARE, V193, P116, DOI 10.1164/rccm.201508-1545CI

Corn KC, 2020, PROG LIPID RES, V80, DOI 10.1016/j.plipres.2020.101055

Cotechini T, 2021, CELLS-BASEL, V10, DOI 10.3390/cells10040960

Cruz AF, 2020, IMMUN INFLAMM DIS, V8, P807, DOI 10.1002/iid3.345

Cui R, 2016, ONCOTARGET, V7, P50735, DOI 10.18632/oncotarget.9383

Davies LC, 2013, NAT IMMUNOL, V14, P986, DOI 10.1038/ni.2705

Dawson CA, 2020, NAT CELL BIOL, V22, P546, DOI 10.1038/s41556-020-0505-0

de Goede KE, 2020, BIOLOGY-BASEL, V9, DOI 10.3390/biology9110380

DeNardo DG, 2019, NAT REV IMMUNOL, V19, P369, DOI 10.1038/s41577-019-0127-6

Dick SA, 2022, SCI IMMUNOL, V7, DOI 10.1126/sciimmunol.abf7777

Dijkgraaf EM, 2013, CANCER RES, V73, P2480, DOI 10.1158/0008-5472.CAN-12-3542

El-Arabey AA, 2020, CELL SIGNAL, V68, DOI 10.1016/j.cellsig.2020.109539

Epelman S, 2014, IMMUNITY, V41, P21, DOI 10.1016/j.immuni.2014.06.013

Etzerodt A, 2020, J EXP MED, V217, DOI 10.1084/jem.20191869

Evren E, 2020, IMMUNOLOGY, V160, P126, DOI 10.1111/imm.13154

Finkernagel F, 2016, ONCOTARGET, V7, P75339, DOI 10.18632/oncotarget.12180

Franklin RA, 2014, SCIENCE, V344, P921, DOI 10.1126/science.1252510

Fukuda A, 2011, CANCER CELL, V19, P441, DOI 10.1016/j.ccr.2011.03.002

Furusawa A, 2018, CARCINOGENESIS, V39, P758, DOI 10.1093/carcin/bgy033

Gan L, 2016, INT J BIOL SCI, V12, P1533, DOI 10.7150/ijbs.15943

Gautier EL, 2012, NAT IMMUNOL, V13, P1118, DOI 10.1038/ni.2419

Geissmann F, 2010, SCIENCE, V327, P656, DOI 10.1126/science.1178331

Gibbings SL, 2017, AM J RESP CELL MOL, V57, P66, DOI 10.1165/rcmb.2016-0361OC

Ginhoux F, 2016, IMMUNITY, V44, P439, DOI 10.1016/j.immuni.2016.02.024

Gómez V, 2020, SCI SIGNAL, V13, DOI 10.1126/scisignal.aax4585

Goossens P, 2019, CELL METAB, V29, P1376, DOI 10.1016/j.cmet.2019.02.016

Guilliams M, 2020, IMMUNITY, V52, P434, DOI 10.1016/j.immuni.2020.02.015

Hagemann T, 2006, J IMMUNOL, V176, P5023, DOI 10.4049/jimmunol.176.8.5023

Hao JQ, 2018, CANCER RES, V78, P2343, DOI 10.1158/0008-5472.CAN-17-2465

He D, 2021, ONCOGENE, V40, P355, DOI 10.1038/s41388-020-01528-0

Hezaveh K, 2022, IMMUNITY, V55, P324, DOI 10.1016/j.immuni.2022.01.006

Hinshaw DC, 2021, CANCER RES, V81, P5425, DOI 10.1158/0008-5472.CAN-20-1723

Hirano R, 2023, COMMUN BIOL, V6, DOI 10.1038/s42003-023-04525-7

Hong L, 2018, PATHOL RES PRACT, V214, P1345, DOI 10.1016/j.prp.2018.07.009

Hou F, 2021, FRONT IMMUNOL, V12, DOI 10.3389/fimmu.2021.753940

Huang L, 2018, J EXP MED, V215, P1135, DOI 10.1084/jem.20172020

Italiani P, 2014, FRONT IMMUNOL, V5, DOI 10.3389/fimmu.2014.00514

Jäppinen N, 2019, NAT COMMUN, V10, DOI 10.1038/s41467-018-08065-1

Jeong H, 2019, CANCER RES, V79, P795, DOI 10.1158/0008-5472.CAN-18-2545

Jha AK, 2015, IMMUNITY, V42, P419, DOI 10.1016/j.immuni.2015.02.005

Jokela H, 2020, EUR J IMMUNOL, V50, P1500, DOI 10.1002/eji.202048531

Karamitopoulou E, 2019, BRIT J CANCER, V121, P5, DOI 10.1038/s41416-019-0479-5

Karger A, 2023, EMBO J, V42, DOI 10.15252/embj.2022111620

Karnevi E, 2014, IMMUNOL CELL BIOL, V92, P543, DOI 10.1038/icb.2014.22

Kawano H, 2016, INT IMMUNOL, V28, P489, DOI 10.1093/intimm/dxw012

Kersten K, 2023, CELL REP, V42, DOI 10.1016/j.celrep.2023.112582

Kim N, 2020, NAT COMMUN, V11, DOI 10.1038/s41467-020-16164-1

Koelwyn GJ, 2018, NAT IMMUNOL, V19, P526, DOI 10.1038/s41590-018-0113-3

Krausgruber T, 2011, NAT IMMUNOL, V12, P231, DOI 10.1038/ni.1990

Kühnemuth B, 2015, ONCOGENE, V34, P177, DOI 10.1038/onc.2013.530

Kulbe H, 2007, CANCER RES, V67, P585, DOI 10.1158/0008-5472.CAN-06-2941

Kumar S, 2022, CANCERS, V14, DOI 10.3390/cancers14215224

Kurahara H, 2011, J SURG RES, V167, pE211, DOI 10.1016/j.jss.2009.05.026

Lahmar Q, 2016, BBA-REV CANCER, V1865, P23, DOI 10.1016/j.bbcan.2015.06.009

Lai JF, 2010, PLOS ONE, V5, DOI 10.1371/journal.pone.0014417

Lane D, 2016, MOL CANCER, V15, DOI 10.1186/s12943-016-0542-2

LaRue MM, 2022, P NATL ACAD SCI USA, V119, DOI 10.1073/pnas.2119168119

Lavin Y, 2017, CELL, V169, P750, DOI 10.1016/j.cell.2017.04.014

Laviron M, 2022, CELL REP, V39, DOI 10.1016/j.celrep.2022.110865

Laviron M, 2019, FRONT IMMUNOL, V10, DOI 10.3389/fimmu.2019.01799

Leone RD, 2020, NAT REV CANCER, V20, P516, DOI 10.1038/s41568-020-0273-y

Li H, 2022, J IMMUNOTHER CANCER, V10, DOI 10.1136/jitc-2021-004029

Li MJ, 2022, CELL BIOSCI, V12, DOI 10.1186/s13578-022-00823-5

Li SS, 2022, CELL REP, V39, DOI 10.1016/j.celrep.2022.110609

Liegeois M, 2018, CELL IMMUNOL, V330, P91, DOI 10.1016/j.cellimm.2018.02.001

Lin SS, 2017, ONCOTARGET, V8, P110426, DOI 10.18632/oncotarget.22786

Liu D, 2017, J PROTEOME RES, V16, P288, DOI 10.1021/acs.jproteome.6b00604

Liu MG, 2019, NAT IMMUNOL, V20, P265, DOI 10.1038/s41590-018-0292-y

Liu PS, 2017, NAT IMMUNOL, V18, P985, DOI 10.1038/ni.3796

Liu Y, 2021, BIOMARK RES, V9, DOI 10.1186/s40364-020-00251-y

Liu Y, 2016, CANCER CELL, V30, P668, DOI 10.1016/j.ccell.2016.09.011

Locati M, 2020, ANNU REV PATHOL-MECH, V15, P123, DOI 10.1146/annurev-pathmechdis-012418-012718

Lopez-Gonzalez JS, 2007, CLIN IMMUNOL, V122, P323, DOI 10.1016/j.clim.2006.11.003

Lopez-Yrigoyen M, 2021, ANN NY ACAD SCI, V1499, P18, DOI 10.1111/nyas.14377

Ma XY, 2016, ONCOL REP, V35, P189, DOI 10.3892/or.2015.4357

Mantovani A, 2022, NAT REV DRUG DISCOV, V21, P799, DOI 10.1038/s41573-022-00520-5

Mantovani A, 2017, NAT REV CLIN ONCOL, V14, P399, DOI 10.1038/nrclinonc.2016.217

Mass E, 2023, NAT REV IMMUNOL, DOI 10.1038/s41577-023-00848-y

Mass E, 2018, INT IMMUNOL, V30, P493, DOI 10.1093/intimm/dxy044

Mehla K, 2019, TRENDS CANCER, V5, P822, DOI 10.1016/j.trecan.2019.10.007

Menga A, 2021, EMBO REP, V22, DOI 10.15252/embr.202051981

Mertens C, 2018, ONCOIMMUNOLOGY, V7, DOI 10.1080/2162402X.2017.1408751

Miller A, 2017, CELL METAB, V26, P788, DOI 10.1016/j.cmet.2017.08.014

Morris JP, 2010, NAT REV CANCER, V10, P683, DOI 10.1038/nrc2899

Morrissey SM, 2021, CELL METAB, V33, P2040, DOI 10.1016/j.cmet.2021.09.002

Mosser DM, 2021, CELL MOL IMMUNOL, V18, P579, DOI 10.1038/s41423-020-00541-3

Motohara T, 2019, ONCOGENE, V38, P2885, DOI 10.1038/s41388-018-0637-x

Mulder WJM, 2019, NAT REV DRUG DISCOV, V18, P553, DOI 10.1038/s41573-019-0025-4

Müller S, 2017, GENOME BIOL, V18, DOI 10.1186/s13059-017-1362-4

Murray PJ, 2014, IMMUNITY, V41, P14, DOI 10.1016/j.immuni.2014.06.008

Murray PJ, 2011, NAT REV IMMUNOL, V11, P723, DOI 10.1038/nri3073

Nagy C, 2015, FRONT IMMUNOL, V6, DOI 10.3389/fimmu.2015.00164

Niu ZY, 2017, NAT COMMUN, V8, DOI 10.1038/s41467-017-00523-6

Nomura M, 2016, NAT IMMUNOL, V17, P216, DOI 10.1038/ni.3366

Nwosu ZC, 2023, NATURE, V618, P151, DOI 10.1038/s41586-023-06073-w

O'Neill LAJ, 2015, IMMUNITY, V42, P393, DOI 10.1016/j.immuni.2015.02.017

Ören B, 2016, J PATHOL, V239, P274, DOI 10.1002/path.4724

Olalekan S, 2021, CELL REP, V35, DOI 10.1016/j.celrep.2021.109165

Ono Y, 2018, SCI REP-UK, V8, DOI 10.1038/s41598-018-25837-3

Panchabhai S, 2016, LEUKEMIA, V30, P951, DOI 10.1038/leu.2015.191

Penny HL, 2021, INT J MOL SCI, V22, DOI 10.3390/ijms22126350

Penny HL, 2016, ONCOIMMUNOLOGY, V5, DOI 10.1080/2162402X.2016.1191731

Puthenveetil A, 2020, ANN TRANSL MED, V8, DOI 10.21037/atm-20-2037

Qian BZ, 2011, NATURE, V475, P222, DOI 10.1038/nature10138

Qiao XH, 2023, LIPIDS HEALTH DIS, V22, DOI 10.1186/s12944-023-01807-1

Qiu SQ, 2018, CANCER TREAT REV, V70, P178, DOI 10.1016/j.ctrv.2018.08.010

Reed JR, 2012, PLOS ONE, V7, DOI 10.1371/journal.pone.0045877

Reinartz S, 2014, INT J CANCER, V134, P32, DOI 10.1002/ijc.28335

Ricketts TD, 2021, FRONT IMMUNOL, V12, DOI 10.3389/fimmu.2021.642285

Saini Y, 2014, BMC GENOMICS, V15, DOI 10.1186/1471-2164-15-726

Salmon H, 2019, NAT REV CANCER, V19, P215, DOI 10.1038/s41568-019-0125-9

Schumann T, 2015, ONCOTARGET, V6, P13416, DOI 10.18632/oncotarget.3826

Schyns J, 2019, NAT COMMUN, V10, DOI 10.1038/s41467-019-11843-0

Schyns J, 2018, J IMMUNOL RES, V2018, DOI 10.1155/2018/5160794

Seim GL, 2022, TRENDS ENDOCRIN MET, V33, P345, DOI 10.1016/j.tem.2022.02.005

Shi SZ, 2019, AM J CANCER RES, V9, P2194

Sica A, 2012, J CLIN INVEST, V122, P787, DOI 10.1172/JCI59643

Sindrilaru A, 2011, J CLIN INVEST, V121, P985, DOI 10.1172/JCI44490

Song QQ, 2019, CANCER MED-US, V8, P3072, DOI 10.1002/cam4.2113

Storz P, 2017, NAT REV GASTRO HEPAT, V14, P296, DOI 10.1038/nrgastro.2017.12

Storz P, 2015, ONCOIMMUNOLOGY, V4, DOI 10.1080/2162402X.2015.1008794

Su SC, 2014, CANCER CELL, V25, P605, DOI 10.1016/j.ccr.2014.03.021

Sumitomo R, 2019, EXP THER MED, V18, P4490, DOI 10.3892/etm.2019.8068

Svedberg FR, 2019, NAT IMMUNOL, V20, P571, DOI 10.1038/s41590-019-0352-y

T'Jonck W, 2018, CELL IMMUNOL, V330, P43, DOI 10.1016/j.cellimm.2018.02.005

Takaishi K, 2010, CANCER SCI, V101, P2128, DOI 10.1111/j.1349-7006.2010.01652.x

Tannahill GM, 2013, NATURE, V496, P238, DOI 10.1038/nature11986

Tao S, 2020, CLIN TRANSL ONCOL, V22, P1938, DOI 10.1007/s12094-020-02348-0

Thibault B, 2014, CANCER METAST REV, V33, P17, DOI 10.1007/s10555-013-9456-2

Ural BB, 2020, SCI IMMUNOL, V5, DOI 10.1126/sciimmunol.aax8756

Van den Bossche J, 2017, TRENDS IMMUNOL, V38, P395, DOI 10.1016/j.it.2017.03.001

Van den Bossche J, 2016, CELL REP, V17, P684, DOI 10.1016/j.celrep.2016.09.008

Van den Bossche J, 2012, J LEUKOCYTE BIOL, V91, P685, DOI 10.1189/jlb.0911453

Vitale I, 2019, CELL METAB, V30, P36, DOI 10.1016/j.cmet.2019.06.001

Wagner J, 2019, CELL, V177, P1330, DOI 10.1016/j.cell.2019.03.005

Wang SY, 2019, CANCER LETT, V452, P14, DOI 10.1016/j.canlet.2019.03.015

Wang S, 2021, SIGNAL TRANSDUCT TAR, V6, DOI 10.1038/s41392-021-00659-4

Wang T, 2023, NAT IMMUNOL, V24, P423, DOI 10.1038/s41590-023-01428-x

Watanabe S, 2019, J CLIN INVEST, V129, P2619, DOI 10.1172/JCI124615

Wculek SK, 2022, CELL MOL IMMUNOL, V19, P384, DOI 10.1038/s41423-021-00791-9

Weiss JM, 2018, J CLIN INVEST, V128, P3794, DOI 10.1172/JCI99169

Weizman N, 2014, ONCOGENE, V33, P3812, DOI 10.1038/onc.2013.357

Wen Z, 2015, ONCOGENE, V34, P1241, DOI 10.1038/onc.2014.85

Wenes M, 2016, CELL METAB, V24, P701, DOI 10.1016/j.cmet.2016.09.008

Worzfeld T, 2017, FRONT ONCOL, V7, DOI 10.3389/fonc.2017.00024

Wyckoff J, 2004, CANCER RES, V64, P7022, DOI 10.1158/0008-5472.CAN-04-1449

Xia HJ, 2020, JCI INSIGHT, V5, DOI 10.1172/jci.insight.141115

Xiang W, 2018, NAT COMMUN, V9, DOI 10.1038/s41467-018-04999-8

Yagi T, 2019, EUR J CANCER, V111, P38, DOI 10.1016/j.ejca.2019.01.018

Ye HL, 2018, CELL DEATH DIS, V9, DOI 10.1038/s41419-018-0486-0

Yeung TL, 2015, AM J PHYSIOL-CELL PH, V309, pC444, DOI 10.1152/ajpcell.00188.2015

Yin MC, 2019, ONCOTARGETS THER, V12, P8687, DOI 10.2147/OTT.S216355

Yin MZ, 2016, J CLIN INVEST, V126, P4157, DOI 10.1172/JCI87252

Ying W, 2019, CELL METAB, V29, P457, DOI 10.1016/j.cmet.2018.12.003

Ying X, 2016, ONCOTARGET, V7, P43076, DOI 10.18632/oncotarget.9246

Yu CY, 2020, CANCER IMMUNOL IMMUN, V69, P115, DOI 10.1007/s00262-019-02445-2

Zaidi NE, 2022, BIOMEDICINE-TAIWAN, V12, P9, DOI 10.37796/2211-8039.1381

Zhang JL, 2023, CELL REP, V42, DOI 10.1016/j.celrep.2023.112620

Zhang MW, 2021, SIGNAL TRANSDUCT TAR, V6, DOI 10.1038/s41392-021-00769-z

Zhang SY, 2023, J IMMUNOTHER CANCER, V11, DOI 10.1136/jitc-2023-007441

Zhang ZJ, 2021, BIOL REPROD, V104, P527, DOI 10.1093/biolre/ioaa219

Zhou HC, 2022, INT REV IMMUNOL, V41, P4, DOI 10.1080/08830185.2021.1955876

Zhu Y, 2017, IMMUNITY, V47, P323, DOI 10.1016/j.immuni.2017.07.014

NR 191

TC 4

Z9 4

U1 6

U2 13

PU WILEY

PI HOBOKEN

PA 111 RIVER ST, HOBOKEN 07030-5774, NJ USA

SN 1574-7891

EI 1878-0261

J9 MOL ONCOL

JI Mol. Oncol.

PD JUL

PY 2024

VL 18

IS 7

BP 1739

EP 1758

DI 10.1002/1878-0261.13618

EA FEB 2024

PG 20

WC Oncology

WE Science Citation Index Expanded (SCI-EXPANDED)

SC Oncology

GA XL2J0

UT WOS:001173522500001

PM 38411356

OA Green Published, gold

DA 2025-02-07

ER

PT J

AU Ham, S

Lima, LG

Lek, E

Möller, A

AF Ham, Sunyoung

Lima, Luize G.

Lek, Erica

Moller, Andreas

TI The Impact of the Cancer Microenvironment on Macrophage Phenotypes

SO FRONTIERS IN IMMUNOLOGY

LA English

DT Review

DE tumor microenvironment; tissue-resident macrophage; monocyte-derived

macrophage; small extracellular vesicles; tumor-derived cytokines

ID TUMOR-ASSOCIATED MACROPHAGES; TISSUE-RESIDENT MACROPHAGES;

GROWTH-FACTOR; BREAST; MONOCYTES; RECEPTOR; BIODISTRIBUTION;

TRANSCRIPTION; POLARIZATION; METASTASIS

AB Within the tumor microenvironment, there is an intricate communication happening between tumor and stromal cells. This information exchange, in the form of cytokines, growth factors, extracellular vesicles, danger molecules, cell debris, and other factors, is capable of modulating the function of immune cells. The triggering of specific responses, including phenotypic alterations, can ultimately result in either immune surveillance or tumor cell survival. Macrophages are a well-studied cell lineage illustrating the different cellular phenotypes possible, depending on the tumor microenvironmental context. While our understanding of macrophage responses is well documentedin vitro, surprisingly, little work has been done to confirm these observations in the cancer microenvironment. In fact, there are examples of opposing reactions of macrophages to cytokines in cell culture andin vivotumor settings. Additionally, it seems that different macrophage lineages, for example tissue-resident and monocyte-derived macrophages, respond differently to cytokines and other cancer-derived signals. In this review article, we will describe and discuss the diverging reports on how cancer cells influence monocyte-derived and tissue-resident macrophage traitsin vivo.

C1 [Ham, Sunyoung; Lima, Luize G.; Lek, Erica; Moller, Andreas] QIMR Berghofer Med Res Inst, Tumor Microenvironm Lab, Herston, Qld, Australia.

[Ham, Sunyoung; Moller, Andreas] Queensland Univ Technol, Sch Biomed Sci, Fac Hlth, Brisbane, Qld, Australia.

C3 QIMR Berghofer Medical Research Institute; Queensland University of

Technology (QUT)

RP Möller, A (corresponding author), QIMR Berghofer Med Res Inst, Tumor Microenvironm Lab, Herston, Qld, Australia.; Möller, A (corresponding author), Queensland Univ Technol, Sch Biomed Sci, Fac Hlth, Brisbane, Qld, Australia.

EM andreas.moller@qimrberghofer.edu.au

RI Möller, Andreas/O-1063-2015

OI G Lima, Luize/0000-0002-4167-6723

FU National Breast Cancer Foundation Australia [IIRS-18-159]; National

Health and Medical Research Council Australia [APP1185907]

FX This work was funded by a grant from the National Breast Cancer

Foundation Australia (IIRS-18-159) and the National Health and Medical

Research Council Australia (APP1185907) to AM.

CR A-Gonzalez N, 2017, J EXP MED, V214, P1281, DOI 10.1084/jem.20161375

ADAMS DO, 1984, ANNU REV IMMUNOL, V2, P283, DOI 10.1146/annurev.immunol.2.1.283

Benner B, 2019, J IMMUNOTHER CANCER, V7, DOI 10.1186/s40425-019-0622-0

Casazza A, 2013, CANCER CELL, V24, P695, DOI 10.1016/j.ccr.2013.11.007

Cassetta L, 2019, CANCER CELL, V35, P588, DOI 10.1016/j.ccell.2019.02.009

Chen WL, 2018, PLOS BIOL, V16, DOI 10.1371/journal.pbio.2005869

Chen XW, 2017, ONCOGENE, V36, P5045, DOI 10.1038/onc.2017.118

Das A, 2018, FRONT IMMUNOL, V9, DOI 10.3389/fimmu.2018.00022

de Vrij J, 2015, INT J CANCER, V137, P1630, DOI 10.1002/ijc.29521

deJonge RR, 1997, ONCOL RES, V9, P89

Dobashi K, 2001, CLIN EXP IMMUNOL, V124, P290, DOI 10.1046/j.1365-2249.2001.01535.x

Domínguez-Soto A, 2011, J IMMUNOL, V186, P2192, DOI 10.4049/jimmunol.1000475

El-Kenawi A, 2019, BRIT J CANCER, V121, P556, DOI 10.1038/s41416-019-0542-2

ERROI A, 1989, INT J CANCER, V44, P795, DOI 10.1002/ijc.2910440508

Finkernagel F, 2016, ONCOTARGET, V7, P75339, DOI 10.18632/oncotarget.12180

Franklin RA, 2014, SCIENCE, V344, P921, DOI 10.1126/science.1252510

Gundra UM, 2014, BLOOD, V123, pE110, DOI 10.1182/blood-2013-08-520619

Guo MM, 2015, PROTEOMICS, V15, P3169, DOI 10.1002/pmic.201400431

Haas L, 2019, FRONT IMMUNOL, V10, DOI 10.3389/fimmu.2019.02746

Haderk F, 2017, SCI IMMUNOL, V2, DOI 10.1126/sciimmunol.aah5509

Ham S, 2018, FRONT IMMUNOL, V9, DOI 10.3389/fimmu.2018.00871

Haque ASMR, 2019, SCI REP-UK, V9, DOI 10.1038/s41598-019-51149-1

Hessvik NP, 2018, CELL MOL LIFE SCI, V75, P193, DOI 10.1007/s00018-017-2595-9

Hsu YL, 2018, MOL THER, V26, P568, DOI 10.1016/j.ymthe.2017.11.016

Irey EA, 2019, P NATL ACAD SCI USA, V116, P12442, DOI 10.1073/pnas.1816410116

Jakobsen KR, 2015, J EXTRACELL VESICLES, V4, DOI 10.3402/jev.v4.26659

Jakubzick C, 2013, IMMUNITY, V39, P599, DOI 10.1016/j.immuni.2013.08.007

Janssen WJ, 2011, AM J RESP CRIT CARE, V184, P547, DOI 10.1164/rccm.201011-1891OC

Jaynes JM, 2020, SCI TRANSL MED, V12, DOI 10.1126/scitranslmed.aax6337

Kano A, 2015, SCI REP-UK, V5, DOI 10.1038/srep08913

Kelly A, 2018, J EXP MED, V215, P2725, DOI 10.1084/jem.20171491

Kersten K, 2017, ONCOIMMUNOLOGY, V6, DOI 10.1080/2162402X.2017.1334744

Kitamura T, 2015, J EXP MED, V212, P1043, DOI 10.1084/jem.20141836

Kosaka N, 2019, EXP MOL MED, V51, DOI 10.1038/s12276-019-0219-1

Lapara NJ, 2010, J INFLAMM-LOND, V7, DOI 10.1186/1476-9255-7-8

LAUDER I, 1977, J CLIN PATHOL, V30, P563, DOI 10.1136/jcp.30.6.563

Liao XD, 2018, P NATL ACAD SCI USA, V115, pE4661, DOI 10.1073/pnas.1720065115

Lücke CD, 2001, CANCER RES, V61, P482

Luo YP, 2006, J CLIN INVEST, V116, P2132, DOI 10.1172/JCI27648

Makita N, 2015, INT IMMUNOL, V27, P131, DOI 10.1093/intimm/dxu090

Martinez F.O., 2014, F1000PRIME REP, V6, P12703, DOI [DOI 10.12703/P6-13, 10.12703/P6-13]

Martínez-Reza I, 2017, J BIOMED SCI, V24, DOI 10.1186/s12929-017-0398-9

Mass E, 2016, SCIENCE, V353, DOI 10.1126/science.aaf4238

Melief SM, 2013, HAEMATOLOGICA, V98, P888, DOI 10.3324/haematol.2012.078055

Merlotti A, 2019, ONCOIMMUNOLOGY, V8, DOI 10.1080/2162402X.2019.1629257

Mills CD, 2000, J IMMUNOL, V164, P6166, DOI 10.4049/jimmunol.164.12.6166

Misharin AV, 2017, J EXP MED, V214, P2387, DOI 10.1084/jem.20162152

Misharin AV, 2013, AM J RESP CELL MOL, V49, P503, DOI 10.1165/rcmb.2013-0086MA

Munro DAD, 2017, FRONT PHYSIOL, V8, DOI 10.3389/fphys.2017.00837

NATHAN CF, 1987, J CLIN INVEST, V79, P319, DOI 10.1172/JCI112815

Ohashi T, 2017, CANCER SCI, V108, P1128, DOI 10.1111/cas.13244

OSULLIVAN C, 1993, LANCET, V342, P148, DOI 10.1016/0140-6736(93)91348-P

PARWARESCH MR, 1984, CELL TISSUE KINET, V17, P25, DOI 10.1111/j.1365-2184.1984.tb00565.x

Perdiguero EG, 2015, NATURE, V518, P547, DOI 10.1038/nature13989

Perentes JY, 2011, CANCER RES, V71, P4527, DOI 10.1158/0008-5472.CAN-10-4376

Plebanek MP, 2017, NAT COMMUN, V8, DOI 10.1038/s41467-017-01433-3

Qian BZ, 2011, NATURE, V475, P222, DOI 10.1038/nature10138

Roca H, 2009, J BIOL CHEM, V284, P34342, DOI 10.1074/jbc.M109.042671

Sanford DE, 2013, CLIN CANCER RES, V19, P3404, DOI 10.1158/1078-0432.CCR-13-0525

Sansone P, 2017, P NATL ACAD SCI USA, V114, pE9066, DOI 10.1073/pnas.1704862114

Schaub FX, 2018, CELL SYST, V6, P282, DOI 10.1016/j.cels.2018.03.003

Sharma SK, 2015, J IMMUNOL, V194, P5529, DOI 10.4049/jimmunol.1403215

Sheng JP, 2015, IMMUNITY, V43, P382, DOI 10.1016/j.immuni.2015.07.016

SHIRAI Y, 1994, CANCER, V73, P2275, DOI 10.1002/1097-0142(19940501)73:9<2275::AID-CNCR2820730907>3.0.CO;2-T

SHIRAYOSHI Y, 1988, P NATL ACAD SCI USA, V85, P5884, DOI 10.1073/pnas.85.16.5884

Svedberg FR, 2019, NAT IMMUNOL, V20, P571, DOI 10.1038/s41590-019-0352-y

TAKEMURA R, 1984, AM J PHYSIOL, V246, pC1, DOI 10.1152/ajpcell.1984.246.1.C1

Théry C, 2018, J EXTRACELL VESICLES, V7, DOI 10.1080/20013078.2018.1535750

Tischer J, 2016, GLIA, V64, P1562, DOI 10.1002/glia.23024

Valadi H, 2007, NAT CELL BIOL, V9, P654, DOI 10.1038/ncb1596

van der Laan LJW, 1999, J IMMUNOL, V162, P939

van der Vos KE, 2016, NEURO-ONCOLOGY, V18, P58, DOI 10.1093/neuonc/nov244

VANFURTH R, 1968, J EXP MED, V128, P415, DOI 10.1084/jem.128.3.415

VIGNAUD JM, 1994, CANCER RES, V54, P5455

Wang QR, 2018, NAT COMMUN, V9, DOI 10.1038/s41467-018-03050-0

Wen SW, 2016, CANCER RES, V76, P6816, DOI 10.1158/0008-5472.CAN-16-0868

Wiklander OPB, 2015, J EXTRACELL VESICLES, V4, DOI 10.3402/jev.v4.26316

Wynn TA, 2016, IMMUNITY, V44, P450, DOI 10.1016/j.immuni.2016.02.015

Yan WJ, 2015, GUT, V64, P1593, DOI 10.1136/gutjnl-2014-307671

Zhu Y, 2017, IMMUNITY, V47, P597, DOI 10.1016/j.immuni.2017.08.018

Zhu Y, 2017, IMMUNITY, V47, P323, DOI 10.1016/j.immuni.2017.07.014

NR 81

TC 18

Z9 20

U1 0

U2 13

PU FRONTIERS MEDIA SA

PI LAUSANNE

PA AVENUE DU TRIBUNAL FEDERAL 34, LAUSANNE, CH-1015, SWITZERLAND

SN 1664-3224

J9 FRONT IMMUNOL

JI Front. Immunol.

PD JUN 23

PY 2020

VL 11

AR 1308

DI 10.3389/fimmu.2020.01308

PG 8

WC Immunology

WE Science Citation Index Expanded (SCI-EXPANDED)

SC Immunology

GA MK3GT

UT WOS:000548672600001

PM 32655574

OA Green Published, gold

DA 2025-02-07

ER

PT J

AU Casanova-Acebes, M

Dalla, E

Leader, AM

LeBerichel, J

Nikolic, J

Morales, BM

Brown, M

Chang, C

Troncoso, L

Chen, ST

Sastre-Perona, A

Park, MD

Tabachnikova, A

Dhainaut, M

Hamon, P

Maier, B

Sawai, CM

Agulló-Pascual, E

Schober, M

Brown, BD

Reizis, B

Marron, T

Kenigsberg, E

Moussion, C

Benaroch, P

Aguirre-Ghiso, JA

Merad, M

AF Casanova-Acebes, Maria

Dalla, Erica

Leader, Andrew M.

LeBerichel, Jessica

Nikolic, Jovan

Morales, Blanca M.

Brown, Markus

Chang, Christie

Troncoso, Leanna

Chen, Steven T.

Sastre-Perona, Ana

Park, Matthew D.

Tabachnikova, Alexandra

Dhainaut, Maxime

Hamon, Pauline

Maier, Barbara

Sawai, Catherine M.

Agullo-Pascual, Esperanza

Schober, Markus

Brown, Brian D.

Reizis, Boris

Marron, Thomas

Kenigsberg, Ephraim

Moussion, Christine

Benaroch, Philippe

Aguirre-Ghiso, Julio A.

Merad, Miriam

TI Tissue-resident macrophages provide a pro-tumorigenic niche to early

NSCLC cells

SO NATURE

LA English

DT Article

ID EPITHELIAL-MESENCHYMAL TRANSITIONS; REGULATORY T-CELLS; STEM-CELLS; EMT;

DIFFERENTIATION; MONOCYTES; ACCUMULATION; PLATFORM; REVEALS; MODELS

AB Macrophages have a key role in shaping the tumour microenvironment (TME), tumour immunity and response to immunotherapy, which makes them an important target for cancer treatment(1,2). However, modulating macrophages has proved extremely difficult, as we still lack a complete understanding of the molecular and functional diversity of the tumour macrophage compartment. Macrophages arise from two distinct lineages. Tissue-resident macrophages self-renew locally, independent of adult haematopoiesis(3-5), whereas short-lived monocyte-derived macrophages arise from adult haematopoietic stem cells, and accumulate mostly in inflamed lesions(1). How these macrophage lineages contribute to the TME and cancer progression remains unclear. To explore the diversity of the macrophage compartment in human non-small cell lung carcinoma (NSCLC) lesions, here we performed single-cell RNA sequencing of tumour-associated leukocytes. We identified distinct populations of macrophages that were enriched in human and mouse lung tumours. Using lineage tracing, we discovered that these macrophage populations differ in origin and have a distinct temporal and spatial distribution in the TME. Tissue-resident macrophages accumulate close to tumour cells early during tumour formation to promote epithelial-mesenchymal transition and invasiveness in tumour cells, and they also induce a potent regulatory T cell response that protects tumour cells from adaptive immunity. Depletion of tissue-resident macrophages reduced the numbers and altered the phenotype of regulatory T cells, promoted the accumulation of CD8(+) T cells and reduced tumour invasiveness and growth. During tumour growth, tissue-resident macrophages became redistributed at the periphery of the TME, which becomes dominated by monocyte-derived macrophages in both mouse and human NSCLC. This study identifies the contribution of tissue-resident macrophages to early lung cancer and establishes them as a target for the prevention and treatment of early lung cancer lesions.

C1 [Casanova-Acebes, Maria; Leader, Andrew M.; LeBerichel, Jessica; Chang, Christie; Troncoso, Leanna; Chen, Steven T.; Park, Matthew D.; Tabachnikova, Alexandra; Hamon, Pauline; Maier, Barbara; Marron, Thomas; Aguirre-Ghiso, Julio A.; Merad, Miriam] Icahn Sch Med Mt Sinai, Dept Oncol Sci, New York, NY 10029 USA.

[Casanova-Acebes, Maria; Leader, Andrew M.; LeBerichel, Jessica; Chang, Christie; Troncoso, Leanna; Chen, Steven T.; Park, Matthew D.; Tabachnikova, Alexandra; Dhainaut, Maxime; Hamon, Pauline; Maier, Barbara; Brown, Brian D.; Marron, Thomas; Kenigsberg, Ephraim; Aguirre-Ghiso, Julio A.; Merad, Miriam] Icahn Sch Med Mt Sinai, Precis Immunol Inst, New York, NY 10029 USA.

[Casanova-Acebes, Maria; Leader, Andrew M.; LeBerichel, Jessica; Chang, Christie; Troncoso, Leanna; Chen, Steven T.; Park, Matthew D.; Tabachnikova, Alexandra; Dhainaut, Maxime; Hamon, Pauline; Maier, Barbara; Brown, Brian D.; Marron, Thomas; Aguirre-Ghiso, Julio A.; Merad, Miriam] Icahn Sch Med Mt Sinai, Tisch Canc Inst, New York, NY 10029 USA.

[Dalla, Erica; Marron, Thomas; Aguirre-Ghiso, Julio A.] Icahn Sch Med Mt Sinai, Div Hematol & Oncol, New York, NY USA.

[Dalla, Erica; Aguirre-Ghiso, Julio A.] Icahn Sch Med Mt Sinai, Dept Med, New York, NY USA.

[Dalla, Erica; Aguirre-Ghiso, Julio A.] Icahn Sch Med Mt Sinai, Black Family Stem Cell Inst, New York, NY USA.

[Dalla, Erica; Aguirre-Ghiso, Julio A.] Icahn Sch Med Mt Sinai, Dept Otolaryngol, New York, NY USA.

[Nikolic, Jovan; Benaroch, Philippe] PSL Res Univ, Inst Curie, INSERM U932, Paris, France.

[Morales, Blanca M.; Brown, Markus; Moussion, Christine] Genentech Inc, Dept Canc Immunol, San Francisco, CA USA.

[Sastre-Perona, Ana; Schober, Markus] NYU, Ronald O Perelman Dept Dermatol, Grossman Sch Med, New York, NY USA.

[Sastre-Perona, Ana] Hosp La Paz, Expt Therapies & Novel Biomarkers Canc, Inst Hlth Res IdiPAZ, Madrid, Spain.

[Dhainaut, Maxime; Brown, Brian D.] Icahn Sch Med Mt Sinai, Dept Genet & Genom Sci, New York, NY 10029 USA.

[Sawai, Catherine M.] Univ Bordeaux, INSERM ACT U1218, Bordeaux, France.

[Agullo-Pascual, Esperanza] Icahn Sch Med Mt Sinai, Microscopy CoRE, Deans CoREs, New York, NY 10029 USA.

[Reizis, Boris] NYU, Dept Pathol, Grossman Sch Med, New York, NY 10016 USA.

[Marron, Thomas] Icahn Sch Med Mt Sinai, Inst Thorac Oncol, New York, NY 10029 USA.

[Brown, Brian D.; Kenigsberg, Ephraim] Icahn Sch Med Mt Sinai, Icahn Genom Inst, New York, NY 10029 USA.

[Merad, Miriam] Icahn Sch Med Mt Sinai, Human Immune Monitoring Ctr, New York, NY 10029 USA.

[Casanova-Acebes, Maria] Spanish Natl Canc Ctr, Canc Immun Lab, Mol Oncol Program, Madrid, Spain.

[Maier, Barbara] Austrian Acad Sci, CeMM, Res Ctr Mol Med, Vienna, Austria.

C3 Icahn School of Medicine at Mount Sinai; Icahn School of Medicine at

Mount Sinai; Icahn School of Medicine at Mount Sinai; Icahn School of

Medicine at Mount Sinai; Icahn School of Medicine at Mount Sinai; Icahn

School of Medicine at Mount Sinai; Icahn School of Medicine at Mount

Sinai; Universite PSL; UNICANCER; Institut Curie; Institut National de

la Sante et de la Recherche Medicale (Inserm); Roche Holding; Genentech;

Roche Holding USA; New York University; Hospital Universitario La Paz;

Icahn School of Medicine at Mount Sinai; Universite de Bordeaux; Icahn

School of Medicine at Mount Sinai; New York University; Icahn School of

Medicine at Mount Sinai; Icahn School of Medicine at Mount Sinai; Icahn

School of Medicine at Mount Sinai; Centro Nacional de Investigaciones

Oncologicas (CNIO); Austrian Academy of Sciences; CeMM Research Center

for Molecular Medicine of the Austrian Academy of Sciences

RP Casanova-Acebes, M; Merad, M (corresponding author), Icahn Sch Med Mt Sinai, Dept Oncol Sci, New York, NY 10029 USA.; Casanova-Acebes, M; Merad, M (corresponding author), Icahn Sch Med Mt Sinai, Precis Immunol Inst, New York, NY 10029 USA.; Casanova-Acebes, M; Merad, M (corresponding author), Icahn Sch Med Mt Sinai, Tisch Canc Inst, New York, NY 10029 USA.; Merad, M (corresponding author), Icahn Sch Med Mt Sinai, Human Immune Monitoring Ctr, New York, NY 10029 USA.; Casanova-Acebes, M (corresponding author), Spanish Natl Canc Ctr, Canc Immun Lab, Mol Oncol Program, Madrid, Spain.

EM mcasanova@cnio.es; miriam.merad@mssm.edu

RI Sawai, Catherine/E-5023-2018; AguirreGhiso, Julio/KXR-3097-2024;

/AAM-5690-2020; Acebes, Maria/AAH-1929-2019; Brown, Brian

D./JOZ-4915-2023; Sastre-Perona, Ana/AAC-1202-2021; Benaroch,

Philippe/KHX-4297-2024; Casanova-Acebes, Maria/HKP-1044-2023

OI Sawai, Catherine/0000-0002-2509-474X; Casanova-Acebes,

Maria/0000-0002-5790-1163; Hamon, Pauline/0000-0002-6776-1635; Troncoso,

Leanna/0000-0003-2529-9267; Reizis, Boris/0000-0003-1140-7853;

Tabachnikova, Alexandra/0000-0003-4695-2480; Nikolic,

Jovan/0000-0003-4119-7009; Chang, Christie/0000-0002-7651-7220; Maier,

Barbara/0000-0002-7359-6576; Sastre-Perona, Ana/0000-0002-4502-3025

FU HFSP postdoctoral fellowship [LT000110/2015-L/1]; AACR-AstraZeneca

Immuno-oncology Research Fellowship [20-40-12-CASA, T32 CA078207, F30

CA243210, ANR-10-IDEX-0001-02 PSL, ANR-11-LABX-0043]; Fondation ARC pour

la recherche sur le cancer; Tisch Cancer Institute at Mount Sinai [P30

CA196521]; National Institutes of Health (NIH)-National Cancer Institute

[CA257195, CA254104, AT011326, AI128949, R56AI137244, CA109182,

CA216248, CA218024]; NCI [T32 CA078207]; NIH [AG049074]; National Cancer

Institute [R01CA218024, R01CA216248] Funding Source: NIH RePORTER

FX This work was supported by an HFSP postdoctoral fellowship

(LT000110/2015-L/1) and an AACR-AstraZeneca Immuno-oncology Research

Fellowship (20-40-12-CASA) to M.C.-A.; T32 CA078207 to A.L.; F30

CA243210 to S.T.C.; and ANR-10-IDEX-0001-02 PSL and ANR-11-LABX-0043 and

Fondation ARC pour la recherche sur le cancer to P.B. This research was

supported in part by the Tisch Cancer Institute at Mount Sinai P30

CA196521-Cancer Center Support Grant. We thank the Human Immune

Monitoring Center for all the single-cell profiling and epigenetic

studies; the Merad laboratory and A. Lujambio for discussions and

reagents; the Flow Cytometry and the Imaging Core at Mount Sinai; and

the Cancer Biorepository at MSSM for sample acquisition. This work was

also supported by National Institutes of Health (NIH)-National Cancer

Institute grants CA257195, CA254104, AT011326, AI128949 and R56AI137244

to M.M., who is also a Samuel Waxman Cancer Research Foundation

Investigator; CA109182, CA216248 and CA218024 to J.A.A.-G., who is also

a Samuel Waxman Cancer Research Foundation Investigator; CA257195,

CA254104, AT011326 and AI128949 to B.D.B.; NCI grant T32 CA078207 to

E.D.; and NIH grant AG049074 to B.R.

CR Nieto MA, 2016, CELL, V166, P21, DOI 10.1016/j.cell.2016.06.028

Bankhead P, 2017, SCI REP-UK, V7, DOI 10.1038/s41598-017-17204-5

Battaglia A, 2009, CANCER IMMUNOL IMMUN, V58, P1363, DOI 10.1007/s00262-008-0646-7

Buenrostro JD, 2013, NAT METHODS, V10, P1213, DOI [10.1038/NMETH.2688, 10.1038/nmeth.2688]

Dongre A, 2019, NAT REV MOL CELL BIO, V20, P69, DOI 10.1038/s41580-018-0080-4

DuPage M, 2009, NAT PROTOC, V4, P1064, DOI 10.1038/nprot.2009.95

Ginhoux F, 2010, SCIENCE, V330, P841, DOI 10.1126/science.1194637

Hashimoto D, 2013, IMMUNITY, V38, P792, DOI 10.1016/j.immuni.2013.04.004

He F, 2012, MOL SYST BIOL, V8, DOI 10.1038/msb.2012.56

Karasawa K, 2015, J AM SOC NEPHROL, V26, P896, DOI 10.1681/ASN.2014020195

Krebs AM, 2017, NAT CELL BIOL, V19, P518, DOI 10.1038/ncb3513

Langmead B, 2009, GENOME BIOL, V10, DOI 10.1186/gb-2009-10-3-r25

Lavin Y, 2017, CELL, V169, P750, DOI 10.1016/j.cell.2017.04.014

Lavin Y, 2015, NAT REV IMMUNOL, V15, P731, DOI 10.1038/nri3920

Leach SM, 2020, CELL REP, V33, DOI 10.1016/j.celrep.2020.108337

Leader A. M., 2020, CITESEQ ANAL NONSMAL, DOI [10.1101/2020.07.16.207605, DOI 10.1101/2020.07.16.207605]

Li H, 2009, BIOINFORMATICS, V25, P2078, DOI 10.1093/bioinformatics/btp352

Lim J, 2012, DEVELOPMENT, V139, P3471, DOI 10.1242/dev.071209

Liu ZY, 2019, CELL, V178, P1509, DOI 10.1016/j.cell.2019.08.009

Love MI, 2014, GENOME BIOL, V15, DOI 10.1186/s13059-014-0550-8

Mantovani A, 2017, NAT REV CLIN ONCOL, V14, P399, DOI 10.1038/nrclinonc.2016.217

Martin JC, 2019, CELL, V178, P1493, DOI 10.1016/j.cell.2019.08.008

McLean CY, 2010, NAT BIOTECHNOL, V28, P495, DOI 10.1038/nbt.1630

Miyake Y, 2007, J CLIN INVEST, V117, P2268, DOI 10.1172/JCI31990

Mizukami Y, 2008, INT J CANCER, V122, P2286, DOI 10.1002/ijc.23392

Mucida D, 2007, SCIENCE, V317, P256, DOI 10.1126/science.1145697

Ramírez F, 2014, NUCLEIC ACIDS RES, V42, pW187, DOI 10.1093/nar/gku365

Remark R, 2016, SCI IMMUNOL, V1, DOI 10.1126/sciimmunol.aaf6925

Rhim AD, 2012, CELL, V148, P349, DOI 10.1016/j.cell.2011.11.025

Robinson JT, 2011, NAT BIOTECHNOL, V29, P24, DOI 10.1038/nbt.1754

Russell DG, 2009, NAT IMMUNOL, V10, P943, DOI 10.1038/ni.1781

Sastre-Perona A, 2019, CELL STEM CELL, V24, P390, DOI 10.1016/j.stem.2019.01.003

Sawai CM, 2016, IMMUNITY, V45, P597, DOI 10.1016/j.immuni.2016.08.007

Schindelin J, 2012, NAT METHODS, V9, P676, DOI [10.1038/NMETH.2019, 10.1038/nmeth.2019]

Schmidt U, 2018, LECT NOTES COMPUT SC, V11071, P265, DOI 10.1007/978-3-030-00934-2_30

Schneider C, 2014, NAT IMMUNOL, V15, P1026, DOI 10.1038/ni.3005

Schulz C, 2012, SCIENCE, V336, P86, DOI 10.1126/science.1219179

Soroosh P, 2013, J EXP MED, V210, P775, DOI 10.1084/jem.20121849

Sutherland KD, 2014, P NATL ACAD SCI USA, V111, P4952, DOI 10.1073/pnas.1319963111

Tada Y, 2018, J IMMUNOTHER CANCER, V6, DOI 10.1186/s40425-018-0403-1

Thiery JP, 2002, NAT REV CANCER, V2, P442, DOI 10.1038/nrc822

Xue W, 2011, CANCER DISCOV, V1, P236, DOI 10.1158/2159-8290.CD-11-0073

Yang J, 2004, CELL, V117, P927, DOI 10.1016/j.cell.2004.06.006

Ye X, 2015, NATURE, V525, P256, DOI 10.1038/nature14897

Yona S, 2013, IMMUNITY, V38, P79, DOI 10.1016/j.immuni.2012.12.001

Zhang Y, 2008, GENOME BIOL, V9, DOI 10.1186/gb-2008-9-9-r137

Zilionis R, 2019, IMMUNITY, V50, P1317, DOI 10.1016/j.immuni.2019.03.009

NR 47

TC 327

Z9 355

U1 18

U2 220

PU NATURE PORTFOLIO

PI BERLIN

PA HEIDELBERGER PLATZ 3, BERLIN, 14197, GERMANY

SN 0028-0836

EI 1476-4687

J9 NATURE

JI Nature

PD JUL 22

PY 2021

VL 595

IS 7868

BP 578

EP +

DI 10.1038/s41586-021-03651-8

EA JUN 2021

PG 22

WC Multidisciplinary Sciences

WE Science Citation Index Expanded (SCI-EXPANDED)

SC Science & Technology - Other Topics

GA TM5GG

UT WOS:000662164200003

PM 34135508

OA Green Accepted

HC Y

HP N

DA 2025-02-07

ER

PT J

AU Loyher, PL

Hamon, P

Laviron, M

Meghraoui-Kheddar, A

Goncalves, E

Deng, ZH

Torstensson, S

Bercovici, N

de Chanville, CB

Combadière, B

Geissmann, F

Savina, A

Combadière, C

Boissonnas, A

AF Loyher, Pierre-Louis

Hamon, Pauline

Laviron, Marie

Meghraoui-Kheddar, Aida

Goncalves, Elena

Deng, Zihou

Torstensson, Sara

Bercovici, Nadege

de Chanville, Camille Baudesson

Combadiere, Behazine

Geissmann, Frederic

Savina, Ariel

Combadiere, Christophe

Boissonnas, Alexandre

TI Macrophages of distinct origins contribute to tumor development in the

lung

SO JOURNAL OF EXPERIMENTAL MEDICINE

LA English

DT Article

ID METASTASIS-ASSOCIATED MACROPHAGES; MONONUCLEAR PHAGOCYTE SYSTEM;

TISSUE-RESIDENT MACROPHAGES; ERYTHRO-MYELOID PROGENITORS; BREAST-CANCER

METASTASIS; GM-CSF; INTERSTITIAL MACROPHAGES; ALVEOLAR MACROPHAGES;

ANTICANCER THERAPIES; FETAL MONOCYTES

AB Tissue-resident macrophages can self-maintain without contribution of adult hematopoiesis. Herein we show that tissue-resident interstitial macrophages (Res-TAMs) in mouse lungs contribute to the pool of tumor-associated macrophages (TAMs) together with CCR2-dependent recruited macrophages (MoD-TAMs). Res-TAMs largely correlated with tumor cell growth in vivo, while MoD-TAMs accumulation was associated with enhanced tumor spreading. Both cell subsets were depleted after chemotherapy, but MoD-TAMs rapidly recovered and performed phagocytosis-mediated tumor clearance. Interestingly, anti-VEGF treatment combined with chemotherapy inhibited both Res and Mod-TAM reconstitution without affecting monocyte infiltration and improved its efficacy. Our results reveal that the developmental origin of TAMs dictates their relative distribution, function, and response to cancer therapies in lung tumors.

C1 [Loyher, Pierre-Louis; Hamon, Pauline; Laviron, Marie; Meghraoui-Kheddar, Aida; Goncalves, Elena; Torstensson, Sara; de Chanville, Camille Baudesson; Combadiere, Behazine; Combadiere, Christophe; Boissonnas, Alexandre] Sorbonne Univ, INSERM, UMR1135, CNRS,ERL8255,CIMI, Paris, France.

[Bercovici, Nadege] Univ Paris 05, Sorbonne Paris Cite, CNRS UMR8104, Inst Cochin,Inserm,U1016, Paris, France.

[Savina, Ariel] Inst Roche, Boulogne 30, France.

[Loyher, Pierre-Louis; Deng, Zihou; Geissmann, Frederic] Mem Sloan Kettering Canc Ctr, Immunol Program, 1275 York Ave, New York, NY 10021 USA.

C3 Sorbonne Universite; Institut National de la Sante et de la Recherche

Medicale (Inserm); Centre National de la Recherche Scientifique (CNRS);

CNRS - National Institute for Biology (INSB); Universite Paris Cite;

Centre National de la Recherche Scientifique (CNRS); CNRS - National

Institute for Biology (INSB); Institut National de la Sante et de la

Recherche Medicale (Inserm); Memorial Sloan Kettering Cancer Center

RP Boissonnas, A (corresponding author), Sorbonne Univ, INSERM, UMR1135, CNRS,ERL8255,CIMI, Paris, France.

EM alexandre.boissonnas@upmc.fr

RI Meghraoui-Kheddar, Aida/O-9631-2017; bercovici, nadege/P-5239-2017;

Combadiere, Behazine/S-8054-2019; Boissonnas, Alexandre/A-2801-2016;

Combadiere, Christophe/I-5639-2013

OI Meghraoui, Aida/0000-0002-7440-5495; Boissonnas,

Alexandre/0000-0002-7770-7210; LOYHER, Pierre-Louis/0000-0003-2315-9711;

Bercovici, Nadege/0000-0002-8449-0590; Hamon,

Pauline/0000-0002-6776-1635; Torstensson, Sara/0000-0003-4389-2662;

Deng, Zihou/0009-0007-2743-668X; Combadiere,

Christophe/0000-0002-1755-4531

FU Fondation ARC pour la Recherche sur le Cancer; Memorial Sloan Kettering

Cancer Center Alan and Sandra Gerry Metastasis and Tumor Ecosystems

Center fellowships; Ligue Contre le Cancer; European Community's Seventh

Framework Program (FP7/2007-2013) [304810]; RAIDs; Institut National de

la Sante et de la Recherche Medicale; Roche; la Ligue Contre le Cancer;

Fondation pour la Recherche Medicale equipe labelisee; National

Institutes of Health (NIH) National Cancer Institute [P30CA008748];

Memorial Sloan Kettering Cancer Center core grant; NIH/National

Institute of Allergy and Infectious Diseases grant [1R01AI13034501];

NIH/National Heart, Lung, and Blood Institute grant [1 R01HL138090-01]

FX P.-L. Loyher is funded by Fondation ARC pour la Recherche sur le Cancer

and Memorial Sloan Kettering Cancer Center Alan and Sandra Gerry

Metastasis and Tumor Ecosystems Center fellowships. P. Hamon is funded

by la Ligue Contre le Cancer. This work was supported by funding from

the European Community's Seventh Framework Program (FP7/2007-2013) No.

304810, RAIDs, Institut National de la Sante et de la Recherche

Medicale, Roche, la Ligue Contre le Cancer, Fondation pour la Recherche

Medicale equipe labelisee, Fondation ARC pour la Recherche sur le Cancer

(to A. Boissonnas), National Institutes of Health (NIH) National Cancer

Institute (grant P30CA008748 to F. Geissmann), Memorial Sloan Kettering

Cancer Center core grant, NIH/National Institute of Allergy and

Infectious Diseases grant 1R01AI13034501 and NIH/National Heart, Lung,

and Blood Institute grant 1 R01HL138090-01 (to F. Geissmann).X

CR Amir ED, 2013, NAT BIOTECHNOL, V31, P545, DOI 10.1038/nbt.2594

Barleon B, 1996, BLOOD, V87, P3336, DOI 10.1182/blood.V87.8.3336.bloodjournal8783336

Boissonnas A, 2013, NEOPLASIA, V15, P85, DOI 10.1593/neo.121572

Bowman RL, 2016, CELL REP, V17, P2445, DOI 10.1016/j.celrep.2016.10.052

Broz ML, 2014, CANCER CELL, V26, P638, DOI 10.1016/j.ccell.2014.09.007

Chen TJ, 2014, CURR TOP MICROBIOL, V377, P127, DOI 10.1007/82_2014_364

Cohen MH, 2007, ONCOLOGIST, V12, P713, DOI 10.1634/theoncologist.12-6-713

Cortez-Retamozo V, 2012, P NATL ACAD SCI USA, V109, P2491, DOI 10.1073/pnas.1113744109

De Palma M, 2013, CANCER CELL, V23, P277, DOI 10.1016/j.ccr.2013.02.013

Dellapasqua S, 2008, J CLIN ONCOL, V26, P4899, DOI 10.1200/JCO.2008.17.4789

Doherty TA, 2011, NAT MED, V17, P596, DOI 10.1038/nm.2356

Engelhardt JJ, 2012, CANCER CELL, V21, P402, DOI 10.1016/j.ccr.2012.01.008

Franklin RA, 2014, SCIENCE, V344, P921, DOI 10.1126/science.1252510

Gambardella L, 2010, SCI SIGNAL, V3, DOI 10.1126/scisignal.2001026

Gibbings SL, 2017, AM J RESP CELL MOL, V57, P66, DOI 10.1165/rcmb.2016-0361OC

Gosselin D, 2014, CELL, V159, P1327, DOI 10.1016/j.cell.2014.11.023

Grunewald M, 2006, CELL, V124, P175, DOI 10.1016/j.cell.2005.10.036

Guilliams M, 2013, J EXP MED, V210, P1977, DOI 10.1084/jem.20131199

Hamon P, 2017, BLOOD, V129, P1296, DOI 10.1182/blood-2016-08-732164

Hawley CA, 2018, J IMMUNOL, V200, P2209, DOI 10.4049/jimmunol.1701488

Hughes R, 2015, CANCER RES, V75, P3479, DOI 10.1158/0008-5472.CAN-14-3587

Jacquelin S, 2013, BLOOD, V122, P674, DOI 10.1182/blood-2013-01-480749

Ji HX, 1998, INT J CANCER, V78, P41, DOI 10.1002/(SICI)1097-0215(19980925)78:1<41::AID-IJC8>3.0.CO;2-X

Jung S, 2000, MOL CELL BIOL, V20, P4106, DOI 10.1128/MCB.20.11.4106-4114.2000

Kaplan RN, 2005, NATURE, V438, P820, DOI 10.1038/nature04186

Kitamura T, 2018, FRONT IMMUNOL, V8, DOI 10.3389/fimmu.2017.02004

Kitamura T, 2015, J EXP MED, V212, P1043, DOI 10.1084/jem.20141836

Lavin Y, 2014, CELL, V159, P1312, DOI 10.1016/j.cell.2014.11.018

Lewis CE, 2016, CANCER CELL, V30, P365, DOI 10.1016/j.ccell.2016.07.009

Lin KY, 1996, CANCER RES, V56, P21

Loyher PL, 2016, CANCER RES, V76, P6483, DOI 10.1158/0008-5472.CAN-16-0984

Maeda K, 2012, NAT MED, V18, P405, DOI 10.1038/nm.2653

Mantovani A, 2015, J EXP MED, V212, P435, DOI 10.1084/jem.20150295

Mass E, 2017, NATURE, V549, P389, DOI 10.1038/nature23672

Mass E, 2016, SCIENCE, V353, DOI 10.1126/science.aaf4238

Misharin AV, 2013, AM J RESP CELL MOL, V49, P503, DOI 10.1165/rcmb.2013-0086MA

Montero A, 2012, CASE REP ONCOL, V5, P687, DOI 10.1159/000346345

Motz GT, 2011, NAT REV IMMUNOL, V11, P702, DOI 10.1038/nri3064

Ovchinnikov DA, 2008, J LEUKOCYTE BIOL, V83, P430, DOI 10.1189/jlb.0807585

Perdiguero EG, 2015, NATURE, V518, P547, DOI 10.1038/nature13989

Pollard JW, 2004, NAT REV CANCER, V4, P71, DOI 10.1038/nrc1256

Qian BZ, 2015, J EXP MED, V212, P1433, DOI 10.1084/jem.20141555

Qian BZ, 2011, NATURE, V475, P222, DOI 10.1038/nature10138

Qian BZ, 2010, CELL, V141, P39, DOI 10.1016/j.cell.2010.03.014

Rodero MP, 2015, ELIFE, V4, DOI 10.7554/eLife.07847

Sabatel C, 2017, IMMUNITY, V46, P457, DOI 10.1016/j.immuni.2017.02.016

Saeed AI, 2003, BIOTECHNIQUES, V34, P374, DOI 10.2144/03342mt01

Saeed AI, 2006, METHOD ENZYMOL, V411, P134, DOI 10.1016/S0076-6879(06)11009-5

Sauter KA, 2014, PLOS ONE, V9, DOI 10.1371/journal.pone.0105429

Schneider C, 2014, NAT IMMUNOL, V15, P1026, DOI 10.1038/ni.3005

Schulz C, 2012, SCIENCE, V336, P86, DOI 10.1126/science.1219179

Tamagnone L, 2012, CANCER CELL, V22, P145, DOI 10.1016/j.ccr.2012.06.031

Tan SYS, 2016, DEVELOPMENT, V143, P1318, DOI 10.1242/dev.129122

Trapnell BC, 2002, ANNU REV PHYSIOL, V64, P775, DOI 10.1146/annurev.physiol.64.090601.113847

van de Laar L, 2016, IMMUNITY, V44, P755, DOI 10.1016/j.immuni.2016.02.017

Wyckoff JB, 2007, CANCER RES, V67, P2649, DOI 10.1158/0008-5472.CAN-06-1823

Zhu Y, 2017, IMMUNITY, V47, P597, DOI 10.1016/j.immuni.2017.08.018

NR 57

TC 194

Z9 222

U1 1

U2 38

PU ROCKEFELLER UNIV PRESS

PI NEW YORK

PA 950 THIRD AVE, 2ND FLR, NEW YORK, NY 10022 USA

SN 0022-1007

EI 1540-9538

J9 J EXP MED

JI J. Exp. Med.

PD OCT

PY 2018

VL 215

IS 10

BP 2536

EP 2553

DI 10.1084/jem.20180534

PG 18

WC Immunology; Medicine, Research & Experimental

WE Science Citation Index Expanded (SCI-EXPANDED)

SC Immunology; Research & Experimental Medicine

GA GX4BZ

UT WOS:000447673200008

PM 30201786

OA Green Submitted, hybrid, Green Published

DA 2025-02-07

ER

PT J

AU Etzerodt, A

Moulin, M

Doktor, TK

Delfini, M

Mossadegh-Keller, N

Bajenoff, M

Sieweke, MH

Moestrup, SK

Auphan-Anezin, N

Lawrence, T

AF Etzerodt, Anders

Moulin, Morgane

Doktor, Thomas Koed

Delfini, Marcello

Mossadegh-Keller, Noushine

Bajenoff, Marc

Sieweke, Michael H.

Moestrup, Soren Kragh

Auphan-Anezin, Nathalie

Lawrence, Toby

TI Tissue-resident macrophages in omentum promote metastatic spread of

ovarian cancer

SO JOURNAL OF EXPERIMENTAL MEDICINE

LA English

DT Article

ID TUMOR-ASSOCIATED MACROPHAGES; STEM-CELLS; MILKY SPOTS; ORGANIZING MAPS;

SELF-RENEWAL; LIPOSOMES; REVEALS; DISSEMINATION; MAINTENANCE; HEMOGLOBIN

AB Experimental and clinical evidence suggests that tumor-associated macrophages (TAMS) play important roles in cancer progression. Here, we have characterized the ontogeny and function of TAM subsets in a mouse model of metastatic ovarian cancer that is representative for visceral peritoneal metastasis. We show that the omentum is a critical premetastatic niche for development of invasive disease in this model and define a unique subset of CD163(+) Tim4(+ )resident omental macrophages responsible for metastatic spread of ovarian cancer cells. Transcriptomic analysis showed that resident CD163(+) Tim4(+) omental macrophages were phenotypically distinct and maintained their resident identity during tumor growth. Selective depletion of CD163(+) Tim4(+) macrophages in omentum using genetic and pharmacological tools prevented tumor progression and metastatic spread of disease. These studies describe a specific role for tissue-resident macrophages in the invasive progression of metastatic ovarian cancer. The molecular pathways of cross-talk between tissue-resident macrophages and disseminated cancer cells may represent new targets to prevent metastasis and disease recurrence.

C1 [Etzerodt, Anders; Moulin, Morgane; Delfini, Marcello; Mossadegh-Keller, Noushine; Bajenoff, Marc; Sieweke, Michael H.; Auphan-Anezin, Nathalie; Lawrence, Toby] Aix Marseille Univ, CIML, INSERM, CNRS, Marseille, France.

[Etzerodt, Anders; Moestrup, Soren Kragh] Univ Aarhus, Dept Biomed, Aarhus, Denmark.

[Moulin, Morgane; Lawrence, Toby] Kings Coll London, Ctr Inflammat Biol & Canc Immunol, Sch Immunol & Microbial Sci, London, England.

[Doktor, Thomas Koed] Univ Southern Denmark, Dept Biochem & Mol Biol, Odense, Denmark.

[Sieweke, Michael H.] Tech Univ Dresden, Ctr Regenerat Therapies, Dresden, Germany.

[Moestrup, Soren Kragh] Univ Southern Denmark, Inst Mol Med, Odense, Denmark.

[Lawrence, Toby] Xinxiang Med Univ, Sch Lab Med, Henan Key Lab Immunol & Targeted Therapy, Xinxiang, Henan, Peoples R China.

C3 Institut National de la Sante et de la Recherche Medicale (Inserm);

Centre National de la Recherche Scientifique (CNRS); Aix-Marseille

Universite; Aarhus University; University of London; King's College

London; University of Southern Denmark; Technische Universitat Dresden;

University of Southern Denmark; Xinxiang Medical University

RP Etzerodt, A; Lawrence, T (corresponding author), Aix Marseille Univ, CIML, INSERM, CNRS, Marseille, France.; Etzerodt, A (corresponding author), Univ Aarhus, Dept Biomed, Aarhus, Denmark.; Lawrence, T (corresponding author), Kings Coll London, Ctr Inflammat Biol & Canc Immunol, Sch Immunol & Microbial Sci, London, England.; Lawrence, T (corresponding author), Xinxiang Med Univ, Sch Lab Med, Henan Key Lab Immunol & Targeted Therapy, Xinxiang, Henan, Peoples R China.

EM ae@biomed.au.dk; toby.lawrence@kcl.ac.uk

RI Delfini, Marcello/KEH-5721-2024; Doktor, Thomas/AAB-2216-2020; Etzerodt,

Anders/O-9860-2019; Sieweke, Michael/AAR-7515-2020; Lawrence,

Toby/GLU-3895-2022; Moestrup, Søren/AAD-1735-2019; Etzerodt,

Anders/E-2839-2013; Lawrence, Toby/F-4461-2015

OI Sieweke, Michael/0000-0002-3228-9537; AUPHAN-ANEZIN,

Nathalie/0000-0002-1967-5206; Mossadegh-Keller,

Noushin/0000-0002-7088-3676; Delfini, Marcello/0000-0003-0342-5445;

Etzerodt, Anders/0000-0002-6757-2068; Lawrence, Toby/0000-0003-0967-6122

FU Novo Nordisk Foundation [NNF14OC0008781]; Agence Nationale de la

Recherche [ANR-09MIEN-029-01, ANR-10-BLAN-1302-01]; FP7 Ideas: European

Research Council 2007-2013 [260753]; Institut national de la sante et de

la recherche medicale; Centre national de la recherche scientifique; Aix

Marseille Universite; France Bio Imaging grant [ANR-10-INBS-04-01];

Agence Nationale de la Recherche (ANR) [ANR-10-BLAN-1302] Funding

Source: Agence Nationale de la Recherche (ANR); European Research

Council (ERC) [260753] Funding Source: European Research Council (ERC)

FX These studies were supported by grants to A. Etzerodt from the Novo

Nordisk Foundation (NNF14OC0008781) and to T. Lawrence from the Agence

Nationale de la Recherche (ANR-09MIEN-029-01 and ANR-10-BLAN-1302-01),

FP7 Ideas: European Research Council 2007-2013 grant agreement number

260753, and institutional funding from Institut national de la sante et

de la recherche medicale, Centre national de la recherche scientifique,

and Aix Marseille Universite. Microscopy facilities are supported by

France Bio Imaging grant ANR-10-INBS-04-01.

CR Abubaker K, 2014, BMC CANCER, V14, DOI 10.1186/1471-2407-14-317

AlHossiny M, 2016, CANCER RES, V76, P3376, DOI 10.1158/0008-5472.CAN-15-2654

Nieto MA, 2016, CELL, V166, P21, DOI 10.1016/j.cell.2016.06.028

Bapat SA, 2005, CANCER RES, V65, P3025, DOI 10.1158/0008-5472.CAN-04-3931

Baratin M, 2017, IMMUNITY, V47, P349, DOI 10.1016/j.immuni.2017.07.019

Bénézech C, 2015, NAT IMMUNOL, V16, P819, DOI 10.1038/ni.3215

Bushnell B, 2014, BBMAP FAST ACCURATE

Chakarov S, 2019, SCIENCE, V363, P1190, DOI 10.1126/science.aau0964

Chakrabarti R, 2018, SCIENCE, V360, P1421, DOI 10.1126/science.aan4153

Charles KA, 2009, J CLIN INVEST, V119, P3011, DOI 10.1172/JCI39065

Chen JF, 2017, STEM CELL REP, V8, P140, DOI 10.1016/j.stemcr.2016.11.008

Chou J, 2010, J CELL PHYSIOL, V222, P42, DOI 10.1002/jcp.21943

Clark R, 2013, AM J PATHOL, V183, P576, DOI 10.1016/j.ajpath.2013.04.023

De Schepper S, 2018, CELL, V175, P400, DOI 10.1016/j.cell.2018.07.048

Dick SA, 2019, NAT IMMUNOL, V20, P29, DOI [10.1038/s41590-018-0272-2, 10.1038/s41590-019-0363-8]

Dobin A, 2013, BIOINFORMATICS, V29, P15, DOI 10.1093/bioinformatics/bts635

Etzerodt A, 2019, J EXP MED, V216, P2394, DOI 10.1084/jem.20182124

Etzerodt A, 2013, ANTIOXID REDOX SIGN, V18, P2352, DOI 10.1089/ars.2012.4834

Etzerodt A, 2013, ANTIOXID REDOX SIGN, V18, P2254, DOI 10.1089/ars.2012.4605

Etzerodt A, 2012, J CONTROL RELEASE, V160, P72, DOI 10.1016/j.jconrel.2012.01.034

Ferlay J., 2020, Cancer Today

Ford CE, 2014, GYNECOL ONCOL, V134, P338, DOI 10.1016/j.ygyno.2014.06.004

Fritze A, 2006, BBA-BIOMEMBRANES, V1758, P1633, DOI 10.1016/j.bbamem.2006.05.028

Garson K, 2015, REPRODUCTION, V149, pR59, DOI 10.1530/REP-14-0234

Ginhoux F, 2016, IMMUNITY, V44, P439, DOI 10.1016/j.immuni.2016.02.024

Ginhoux F, 2010, SCIENCE, V330, P841, DOI 10.1126/science.1194637

Ginhoux F, 2010, IMMUNOL CELL BIOL, V88, P387, DOI 10.1038/icb.2010.38

Goossens P, 2019, CELL METAB, V29, P1376, DOI 10.1016/j.cmet.2019.02.016

Gray EE, 2012, J INNATE IMMUN, V4, P424, DOI 10.1159/000337007

Hafemeister C, 2019, GENOME BIOL, V20, DOI 10.1186/s13059-019-1874-1

Hagemann T, 2008, J EXP MED, V205, P1261, DOI 10.1084/jem.20080108

HAGIWARA A, 1993, CANCER RES, V53, P687

Hansen KD, 2012, BIOSTATISTICS, V13, P204, DOI 10.1093/biostatistics/kxr054

Jäppinen N, 2019, NAT COMMUN, V10, DOI 10.1038/s41467-018-08065-1

Jung Y, 2016, ONCOTARGET, V7, P25698, DOI 10.18632/oncotarget.8365

Kim D, 2018, NAT REV CANCER, V13, P273, DOI [10.1038/nrc3432, DOI 10.1038/NRC3432]

Komohara Y, 2014, CANCER SCI, V105, P1, DOI 10.1111/cas.12314

Kreso A, 2014, CELL STEM CELL, V14, P275, DOI 10.1016/j.stem.2014.02.006

KRIST LFG, 1995, ANAT REC, V241, P163, DOI 10.1002/ar.1092410204

Lahmar Q, 2016, BBA-REV CANCER, V1865, P23, DOI 10.1016/j.bbcan.2015.06.009

Langyel E, 2010, AM J PATHOL, V177, P1053, DOI 10.2353/ajpath.2010.100105

Lee CC, 2018, NAT COMMUN, V9, DOI 10.1038/s41467-018-06268-0

Lee KM, 2012, INT J ONCOL, V40, P665, DOI 10.3892/ijo.2011.1289

Lee W, 2019, J EXP MED, V216, P176, DOI 10.1084/jem.20181170

Levin TG, 2010, GASTROENTEROLOGY, V139, P2072, DOI 10.1053/j.gastro.2010.08.053

Levina VV, 2009, CANCER RES, V69, P5226, DOI 10.1158/0008-5472.CAN-08-4652

Lim HY, 2018, IMMUNITY, V49, P326, DOI [10.1016/j.immuni.2018.06.008, 10.1016/j.immuni.2018.12.009]

Love MI, 2014, GENOME BIOL, V15, DOI 10.1186/s13059-014-0550-8

Loyher PL, 2018, J EXP MED, V215, P2536, DOI 10.1084/jem.20180534

Lupia M, 2017, MOL CANCER, V16, DOI 10.1186/s12943-017-0638-3

Meng E, 2014, PLOS ONE, V9, DOI 10.1371/journal.pone.0107142

Mossadegh-Keller N, 2017, J EXP MED, V214, P2829, DOI 10.1084/jem.20170829

Motz GT, 2014, NAT MED, V20, P607, DOI 10.1038/nm.3541

Noy R, 2014, IMMUNITY, V41, P49, DOI 10.1016/j.immuni.2014.06.010

Ojalvo LS, 2010, J IMMUNOL, V184, P702, DOI 10.4049/jimmunol.0902360

Oktem G, 2014, ONCOL REP, V32, P641, DOI 10.3892/or.2014.3252

Parte SC, 2018, J OVARIAN RES, V11, DOI 10.1186/s13048-018-0439-3

Pattabiraman DR, 2014, NAT REV DRUG DISCOV, V13, P497, DOI 10.1038/nrd4253

Pearce OMT, 2018, CANCER DISCOV, V8, P304, DOI 10.1158/2159-8290.CD-17-0284

Pollard JW, 2009, NAT REV IMMUNOL, V9, P259, DOI 10.1038/nri2528

Raggi C, 2016, ONCOGENE, V35, P671, DOI 10.1038/onc.2015.132

Rangel-Moreno J, 2009, IMMUNITY, V30, P731, DOI 10.1016/j.immuni.2009.03.014

Roby KF, 2000, CARCINOGENESIS, V21, P585, DOI 10.1093/carcin/21.4.585

Rosas M, 2014, SCIENCE, V344, P645, DOI 10.1126/science.1251414

Saygin C, 2017, J EXP MED, V214, P2715, DOI 10.1084/jem.20170438

Schreiber HA, 2013, J EXP MED, V210, P2025, DOI 10.1084/jem.20130903

Schulz C, 2012, SCIENCE, V336, P86, DOI 10.1126/science.1219179

Scott CL, 2016, NAT COMMUN, V7, DOI 10.1038/ncomms10321

Sehouli J, 2009, J SURG ONCOL, V99, P424, DOI 10.1002/jso.21288

Seo EJ, 2016, STEM CELLS, V34, P551, DOI 10.1002/stem.2279

Solar P, 2008, INT J CANCER, V122, P281, DOI 10.1002/ijc.23071

Soucie EL, 2016, SCIENCE, V351, DOI 10.1126/science.aad5510

Steg AD, 2012, CLIN CANCER RES, V18, P869, DOI 10.1158/1078-0432.CCR-11-2188

Torchilin VP, 2001, BBA-BIOMEMBRANES, V1511, P397, DOI 10.1016/S0005-2728(01)00165-7

Wang EF, 2011, PLOS ONE, V6, DOI 10.1371/journal.pone.0017918

Wang Qi-En, 2015, World J Biol Chem, V6, P57, DOI 10.4331/wjbc.v6.i3.57

Wang YN, 2018, JCI INSIGHT, V3, DOI 10.1172/jci.insight.122360

Wehrens R, 2018, J STAT SOFTW, V87, P1, DOI 10.18637/iss.v087.i07

Wehrens R, 2007, J STAT SOFTW, V21, P1

Wei LW, 2017, MEDICINE, V96, DOI 10.1097/MD.0000000000006345

Witt AE, 2017, ONCOGENE, V36, P1707, DOI 10.1038/onc.2016.337

Yan W, 2010, J BIOL CHEM, V285, P14042, DOI 10.1074/jbc.M110.105262

Yang M, 2018, CANCER RES, V78, P5492, DOI 10.1158/0008-5472.CAN-18-1367

Yang Z, 2017, CLIN CANCER RES, V23, P6673, DOI 10.1158/1078-0432.CCR-17-0882

Yasuda K, 2016, BIOCHEM BIOPH RES CO, V472, P643, DOI 10.1016/j.bbrc.2016.03.004

Yates A, 2016, NUCLEIC ACIDS RES, V44, pD710, DOI 10.1093/nar/gkv1157

Yona S, 2013, IMMUNITY, V38, P79, DOI 10.1016/j.immuni.2012.12.001

Yu GC, 2012, OMICS, V16, P284, DOI 10.1089/omi.2011.0118

Zhang J, 2019, ONCOL LETT, V17, P5351, DOI 10.3892/ol.2019.10221

Zhu Y, 2017, IMMUNITY, V47, P323, DOI 10.1016/j.immuni.2017.07.014

NR 90

TC 190

Z9 202

U1 1

U2 31

PU ROCKEFELLER UNIV PRESS

PI NEW YORK

PA 950 THIRD AVE, 2ND FLR, NEW YORK, NY 10022 USA

SN 0022-1007

EI 1540-9538

J9 J EXP MED

JI J. Exp. Med.

PD APR

PY 2020

VL 217

IS 4

AR e20191869

DI 10.1084/jem.20191869

PG 28

WC Immunology; Medicine, Research & Experimental

WE Science Citation Index Expanded (SCI-EXPANDED)

SC Immunology; Research & Experimental Medicine

GA LK7FC

UT WOS:000531027000018

PM 31951251

OA Green Published, hybrid

DA 2025-02-07

ER

PT J

AU Matsui, T

Taniguchi, S

Ishii, M

AF Matsui, Takahiro

Taniguchi, Seiji

Ishii, Masaru

TI Function of alveolar macrophages in lung cancer microenvironment

SO INFLAMMATION AND REGENERATION

LA English

DT Review

DE Alveolar macrophage; Tissue-resident macrophage; Lung cancer; Cancer

microenvironment; INHBA; Activin A

ID TISSUE-RESIDENT MACROPHAGES; ACTIVIN-A; CELLS; PROGRESSION; MONOCYTES;

HEALTH; LIFE

AB Background Cancer tissues contain a wide variety of immune cells that play critical roles in suppressing or promoting tumor progression. Macrophages are one of the most predominant populations in the tumor microenvironment and are composed of two classes: infiltrating macrophages from the bone marrow and tissue-resident macrophages (TRMs). This review aimed to outline the function of TRMs in the tumor microenvironment, focusing on lung cancer. Review Although the functions of infiltrating macrophages and tumor-associated macrophages have been intensively analyzed, a comprehensive understanding of TRM function in cancer is relatively insufficient because it differs depending on the tissue and organ. Alveolar macrophages (AMs), one of the most important TRMs in the lungs, are replenished in situ, independent of hematopoietic stem cells in the bone marrow, and are abundant in lung cancer tissue. Recently, we reported that AMs support cancer cell proliferation and contribute to unfavorable outcomes. Conclusion In this review, we introduce the functions of AMs in lung cancer and their underlying molecular mechanisms. A thorough understanding of the functions of AMs in lung cancer will lead to improved treatment outcomes.

C1 [Matsui, Takahiro; Taniguchi, Seiji; Ishii, Masaru] Osaka Univ, Grad Sch Med, Dept Immunol & Cell Biol, Suita, Osaka, Japan.

[Matsui, Takahiro] Osaka Univ, Grad Sch Med, Dept Pathol, Suita, Osaka, Japan.

[Taniguchi, Seiji] Osaka Univ, Grad Sch Med, Dept Thorac Surg, Suita, Osaka, Japan.

[Taniguchi, Seiji] Osaka Habikino Med Ctr, Dept Thorac Surg, Habikino, Osaka, Japan.

C3 Osaka University; Osaka University; Osaka University

RP Matsui, T (corresponding author), Osaka Univ, Grad Sch Med, Dept Immunol & Cell Biol, Suita, Osaka, Japan.; Matsui, T (corresponding author), Osaka Univ, Grad Sch Med, Dept Pathol, Suita, Osaka, Japan.

EM matsuit@molpath.med.osaka-u.ac.jp

FU Japan Society for the Promotion of Science

FX We would like to thank Dr. Junichi Kikuta for useful discussion and kind

assistance. Figures and illustrations were partially created with

BioRender.com. We would like to thank Editage (www.editage.com) for

English language editing.

CR Aegerter H, 2022, IMMUNITY, V55, P1564, DOI 10.1016/j.immuni.2022.08.010

Bald T, 2020, NAT IMMUNOL, V21, P835, DOI 10.1038/s41590-020-0728-z

Beattie GM, 2005, STEM CELLS, V23, P489, DOI 10.1634/stemcells.2004-0279

Bettinger I, 2002, ACTA NEUROPATHOL, V103, P351, DOI 10.1007/s00401-001-0472-x

Cao MM, 2024, EXP HEMATOL ONCOL, V13, DOI 10.1186/s40164-023-00469-0

Casanova-Acebes M, 2021, NATURE, V595, P578, DOI 10.1038/s41586-021-03651-8

Christofides A, 2022, NAT IMMUNOL, V23, P1148, DOI 10.1038/s41590-022-01267-2

Cohen N, 2023, CANCER RES, V83, P3354, DOI 10.1158/0008-5472.CAN-22-3707

Davies LC, 2013, NAT IMMUNOL, V14, P986, DOI 10.1038/ni.2705

De Simone G, 2021, IMMUNITY, V54, P2089, DOI 10.1016/j.immuni.2021.05.005

Fu YY, 2022, NAT COMMUN, V13, DOI 10.1038/s41467-022-31596-7

Guilliams M, 2013, J EXP MED, V210, P1977, DOI 10.1084/jem.20131199

Hashimoto D, 2013, IMMUNITY, V38, P792, DOI 10.1016/j.immuni.2013.04.004

Hill W, 2023, NATURE, V616, P159, DOI 10.1038/s41586-023-05874-3

Hoda MA, 2016, ONCOTARGET, V7, P13388, DOI 10.18632/oncotarget.7796

Hussell T, 2014, NAT REV IMMUNOL, V14, P81, DOI 10.1038/nri3600

Kagawa Y, 2023, CANCER SCI, V114, P3423, DOI 10.1111/cas.15848

Kuhlmann-Hogan A, 2024, CANCER DISCOV, V14, P524, DOI 10.1158/2159-8290.CD-23-0434

Matsui T, 2020, CANCER RES, V80, P3745, DOI 10.1158/0008-5472.CAN-20-0348

Memmott RM, 2021, J THORAC ONCOL, V16, P1086, DOI 10.1016/j.jtho.2021.03.017

Morrell ED, 2018, JCI INSIGHT, V3, DOI 10.1172/jci.insight.99281

Mowat AM, 2017, NAT MED, V23, P1258, DOI 10.1038/nm.4430

Neupane AS, 2020, CELL, V183, P110, DOI 10.1016/j.cell.2020.08.020

Nobs SP, 2021, TRENDS IMMUNOL, V42, P495, DOI 10.1016/j.it.2021.04.007

PALLERONI AV, 1991, INT J CANCER, V49, P296, DOI 10.1002/ijc.2910490226

Perdiguero EG, 2015, NATURE, V518, P547, DOI 10.1038/nature13989

Rosen ED, 1999, MOL CELL, V4, P611, DOI 10.1016/S1097-2765(00)80211-7

Schenkel JM, 2023, NAT REV IMMUNOL, V23, P807, DOI 10.1038/s41577-023-00884-8

Schmidt A, 2007, J LEUKOCYTE BIOL, V81, P186, DOI 10.1189/jlb.0606377

Taniguchi S, 2023, NAT COMMUN, V14, DOI 10.1038/s41467-022-35701-8

Trapnell BC, 2009, CURR OPIN IMMUNOL, V21, P514, DOI 10.1016/j.coi.2009.09.004

van Rooijen N, 2010, METHODS MOL BIOL, V605, P189, DOI 10.1007/978-1-60327-360-2_13

Wamsley JJ, 2015, CANCER RES, V75, P426, DOI 10.1158/0008-5472.CAN-13-2702

Xue RD, 2022, NATURE, V612, P141, DOI 10.1038/s41586-022-05400-x

Yang YH, 2021, J THORAC DIS, V13, P5826, DOI 10.21037/jtd-21-854

Yona S, 2013, IMMUNITY, V38, P79, DOI 10.1016/j.immuni.2012.12.001

Yoshinaga K, 2004, CLIN CANCER RES, V10, P5702, DOI 10.1158/1078-0432.CCR-03-0262

Zhang FL, 2024, ONCOL REP, V51, DOI 10.3892/or.2023.8688

Zhang PY, 2018, CANCER CELL INT, V18, DOI 10.1186/s12935-018-0664-2

NR 39

TC 0

Z9 0

U1 6

U2 8

PU BMC

PI LONDON

PA CAMPUS, 4 CRINAN ST, LONDON N1 9XW, ENGLAND

EI 1880-8190

J9 INFLAMM REGEN

JI Inflamm. Regen.

PD MAY 8

PY 2024

VL 44

IS 1

AR 23

DI 10.1186/s41232-024-00335-4

PG 6

WC Immunology; Medicine, Research & Experimental

WE Science Citation Index Expanded (SCI-EXPANDED)

SC Immunology; Research & Experimental Medicine

GA PT2F8

UT WOS:001216262600001

PM 38720352

OA gold

DA 2025-02-07

ER

PT J

AU Hirano, R

Okamoto, K

Shinke, M

Sato, M

Watanabe, S

Watanabe, H

Kondoh, G

Kadonosono, T

Kizaka-Kondoh, S

AF Hirano, Ryuichiro

Okamoto, Koki

Shinke, Miyu

Sato, Marika

Watanabe, Shigeaki

Watanabe, Hitomi

Kondoh, Gen

Kadonosono, Tetsuya

Kizaka-Kondoh, Shinae

TI Tissue-resident macrophages are major tumor-associated macrophage

resources, contributing to early TNBC development, recurrence, and

metastases

SO COMMUNICATIONS BIOLOGY

LA English

DT Article

ID ANGIOGENESIS; CLODRONATE; ORIGINATE; PROGNOSIS; LIPOSOMES; DEPLETION;

ROLES; CELLS

AB The role of mammary gland tissue-resident macrophages (MGTRMs) in the development of TNBC breast tumors and recurrence after surgery and chemotherapy is investigated.

Triple-negative breast cancer (TNBC) is an aggressive and highly heterogenous disease with no well-defined therapeutic targets. Treatment options are thus limited and mortality is significantly higher compared with other breast cancer subtypes. Mammary gland tissue-resident macrophages (MGTRMs) are found to be the most abundant stromal cells in early TNBC before angiogenesis. We therefore aimed to explore novel therapeutic approaches for TNBC by focusing on MGTRMs. Local depletion of MGTRMs in mammary gland fat pads the day before TNBC cell transplantation significantly reduced tumor growth and tumor-associated macrophage (TAM) infiltration in mice. Furthermore, local depletion of MGTRMs at the site of TNBC resection markedly reduced recurrence and distant metastases, and improved chemotherapy outcomes. This study demonstrates that MGTRMs are a major TAM resource and play pivotal roles in the growth and malignant progression of TNBC. The results highlight a possible novel anti-cancer approach targeting tissue-resident macrophages.

C1 [Hirano, Ryuichiro; Okamoto, Koki; Shinke, Miyu; Sato, Marika; Watanabe, Shigeaki; Kadonosono, Tetsuya; Kizaka-Kondoh, Shinae] Tokyo Inst Technol, Sch Life Sci & Technol, Yokohama 2268501, Japan.

[Watanabe, Hitomi; Kondoh, Gen] Kyoto Univ, Inst Life & Med Sci, Sakyo Ku, Kyoto 6068507, Japan.

C3 Institute of Science Tokyo; Tokyo Institute of Technology; Kyoto

University

RP Kizaka-Kondoh, S (corresponding author), Tokyo Inst Technol, Sch Life Sci & Technol, Yokohama 2268501, Japan.

EM skondoh@bio.titech.ac.jp

RI Kadonosono, Tetsuya/D-4651-2019; Kondoh, Shinae/AAF-8745-2020; Kondoh,

Shinae/C-6937-2015

OI Kondoh, Shinae/0000-0003-3085-5782

FU Princess Takamatsu Cancer Research Fund; Uehara Memorial Foundation;

NOVARTIS Foundation of Japan; MEXT Quantum Leap Flagship Program (MEXT

QLEAP) [JPMXS0120330644]

FX The authors thank Dr. Yasufumi Asao (luxonus Inc.

https://www.luxonus.jp/) for technical support of photoacoustic imaging,

and the Open Research Facilities for Life Science and Technology, Tokyo

Institute of Technology for supporting immunohistochemical analysis.

This study was supported by the Princess Takamatsu Cancer Research Fund

(S. K.-K.), the Uehara Memorial Foundation (S. K.-K.), the NOVARTIS

Foundation of Japan (S. K.-K), and MEXT Quantum Leap Flagship Program

(MEXT QLEAP) Grant Number JPMXS0120330644 (S. K.-K). We thank Melissa

Crawford, PhD, from Edanz (https://jp.edanz.com/ac) for editing a draft

of this paper.

CR Alberto M., 2017, NAT REV CLIN ONCOL, V14, P399

Arroyo-Crespo JJ, 2019, INT J CANCER, V145, P2267, DOI 10.1002/ijc.32270

Asao Y, 2022, ULTRASONIC IMAGING, V44, P96, DOI 10.1177/01617346221099201

Asao Y, 2016, J BIOMED OPT, V21, DOI 10.1117/1.JBO.21.11.116009

Atabai K, 2007, J MAMMARY GLAND BIOL, V12, P37, DOI 10.1007/s10911-007-9036-6

Bednarczyk Robert B, 2018, Oncotarget, V9, P24272, DOI 10.18632/oncotarget.24917

Belkacemi Y, 2018, FRONT ONCOL, V8, DOI 10.3389/fonc.2018.00112

Betts CB, 2018, J IMMUNOL, V201, P734, DOI 10.4049/jimmunol.1800023

Bowman RL, 2016, CELL REP, V17, P2445, DOI 10.1016/j.celrep.2016.10.052

Casanova-Acebes M, 2021, NATURE, V595, P578, DOI 10.1038/s41586-021-03651-8

Cassetta L, 2018, NAT REV DRUG DISCOV, V17, P887, DOI 10.1038/nrd.2018.169

Chacón RD, 2010, BREAST CANCER RES, V12, DOI 10.1186/bcr2574

Chakrabarti R, 2018, SCIENCE, V360, P1421, DOI 10.1126/science.aan4153

Chakrabarty A, 2021, FRONT ONCOL, V11, DOI 10.3389/fonc.2021.674354

Condeelis J, 2006, CELL, V124, P263, DOI 10.1016/j.cell.2006.01.007

Crane MJ, 2014, PLOS ONE, V9, DOI 10.1371/journal.pone.0086660

Davies LC, 2013, NAT IMMUNOL, V14, P986, DOI 10.1038/ni.2705

Dawson CA, 2020, NAT CELL BIOL, V22, P546, DOI 10.1038/s41556-020-0505-0

De Palma M, 2013, CANCER CELL, V23, P277, DOI 10.1016/j.ccr.2013.02.013

Eiermann W, 2012, ANN ONCOL, V23, P30, DOI 10.1093/annonc/mds192

Etzerodt A, 2020, J EXP MED, V217, DOI 10.1084/jem.20191869

Franklin RA, 2014, SCIENCE, V344, P921, DOI 10.1126/science.1252510

Gyorki DE, 2009, BREAST CANCER RES, V11, DOI 10.1186/bcr2353

Hamilton N, 2009, TRAFFIC, V10, P951, DOI 10.1111/j.1600-0854.2009.00938.x

Hanson SE, 2022, JAMA SURG, V157, DOI 10.1001/jamasurg.2022.0631

He XM, 2017, SCI REP-UK, V7, DOI 10.1038/s41598-017-10729-9

Hiraga T, 2004, CLIN CANCER RES, V10, P4559, DOI 10.1158/1078-0432.CCR-03-0325

Huang Y, 2015, ONCOTARGET, V6, P17462, DOI 10.18632/oncotarget.3958

Ikebuchi R, 2020, J REPROD IMMUNOL, V140, DOI 10.1016/j.jri.2020.103137

Jäppinen N, 2019, NAT COMMUN, V10, DOI 10.1038/s41467-018-08065-1

Jing YY, 2011, CELL BIOSCI, V1, DOI 10.1186/2045-3701-1-29

Johnstone CN, 2015, DIS MODEL MECH, V8, P237, DOI 10.1242/dmm.017830

Kim IS, 2019, NAT CELL BIOL, V21, P1113, DOI 10.1038/s41556-019-0373-7

Kuchimaru T, 2018, NAT COMMUN, V9, DOI 10.1038/s41467-018-05366-3

Kumar P, 2016, ARCH GYNECOL OBSTET, V293, P247, DOI 10.1007/s00404-015-3859-y

Kwon YS, 2019, ONCOL LETT, V17, P2523, DOI 10.3892/ol.2018.9827

Lavin Y, 2014, CELL, V159, P1312, DOI 10.1016/j.cell.2014.11.018

Leek RD, 1996, CANCER RES, V56, P4625

Lehmann BD, 2011, J CLIN INVEST, V121, P2750, DOI 10.1172/JCI45014

Linde N, 2018, NAT COMMUN, V9, DOI 10.1038/s41467-017-02481-5

Liu X, 2013, ONCOIMMUNOLOGY, V2, DOI 10.4161/onci.23972

Loyher PL, 2018, J EXP MED, V215, P2536, DOI 10.1084/jem.20180534

Madajewski B, 2012, CLIN CANCER RES, V18, P5741, DOI 10.1158/1078-0432.CCR-12-1188

Nguyen MTA, 2007, J BIOL CHEM, V282, P35279, DOI 10.1074/jbc.M706762200

Nichele L, 2020, OSA CONTINUUM, V3, P1417, DOI 10.1364/OSAC.393971

Perdiguero EG, 2015, NATURE, V518, P547, DOI 10.1038/nature13989

Pittet MJ, 2022, NAT REV CLIN ONCOL, V19, P402, DOI 10.1038/s41571-022-00620-6

Plesca I, 2020, FRONT IMMUNOL, V11, DOI 10.3389/fimmu.2020.00364

Pogoda K, 2013, MED ONCOL, V30, DOI 10.1007/s12032-012-0388-4

Qiu JD, 2016, J CANCER, V7, P167, DOI 10.7150/jca.10944

Ramos RN, 2022, CELL, V185, P1189, DOI 10.1016/j.cell.2022.02.021

Soncin I, 2018, NAT COMMUN, V9, DOI 10.1038/s41467-018-02834-8

Valkenburg KC, 2018, NAT REV CLIN ONCOL, V15, P366, DOI 10.1038/s41571-018-0007-1

Valkovic T, 2002, VIRCHOWS ARCH, V440, P583, DOI 10.1007/s004280100458

vanRooijen N, 1996, J IMMUNOL METHODS, V193, P93, DOI 10.1016/0022-1759(96)00056-7

VANROOIJEN N, 1994, J IMMUNOL METHODS, V174, P83

Veronesi U, 2002, NEW ENGL J MED, V347, P1227, DOI 10.1056/NEJMoa020989

Wang XY, 2020, P NATL ACAD SCI USA, V117, P20729, DOI 10.1073/pnas.1915950117

Wang Y, 2020, ELIFE, V9, DOI 10.7554/eLife.57438

Ward NL, 2011, BRIT J DERMATOL, V164, P750, DOI 10.1111/j.1365-2133.2010.10129.x

Wei QY, 2020, ONCOGENE, V39, P6139, DOI 10.1038/s41388-020-01432-7

Wu YY, 2021, FRONT CELL DEV BIOL, V8, DOI 10.3389/fcell.2020.617879

Xu MH, 2005, PHYS REV E, V71, DOI 10.1103/PhysRevE.71.016706

Yagata H, 2011, BREAST CANCER-TOKYO, V18, P165, DOI 10.1007/s12282-011-0254-9

Zhang QW, 2012, PLOS ONE, V7, DOI 10.1371/journal.pone.0050946

Zhu Y, 2017, IMMUNITY, V47, P323, DOI 10.1016/j.immuni.2017.07.014

NR 66

TC 19

Z9 20

U1 1

U2 3

PU NATURE PORTFOLIO

PI BERLIN

PA HEIDELBERGER PLATZ 3, BERLIN, 14197, GERMANY

EI 2399-3642

J9 COMMUN BIOL

JI Commun. Biol.

PD FEB 3

PY 2023

VL 6

IS 1

AR 144

DI 10.1038/s42003-023-04525-7

PG 11

WC Biology; Multidisciplinary Sciences

WE Science Citation Index Expanded (SCI-EXPANDED)

SC Life Sciences & Biomedicine - Other Topics; Science & Technology - Other

Topics

GA 9J9IF

UT WOS:000940490500001

PM 36737474

OA gold, Green Published

DA 2025-02-07

ER

PT J

AU Lahmar, Q

Keirsse, J

Laoui, D

Movahedi, K

Van Overmeire, E

Van Ginderachter, JA

AF Lahmar, Qods

Keirsse, Jiri

Laoui, Damya

Movahedi, Kiavash

Van Overmeire, Eva

Van Ginderachter, Jo A.

TI Tissue-resident versus monocyte-derived macrophages in the tumor

microenvironment

SO BIOCHIMICA ET BIOPHYSICA ACTA-REVIEWS ON CANCER

LA English

DT Article

DE Tumor-associated macrophages; Tissue-resident macrophages;

Monocyte-derived macrophages; Kupffer cell; Microglia; Breast cancer;

Lung cancer; Pancreatic adenocarcinoma; Glioma; Hepatocellular carcinoma

ID CELL LUNG-CANCER; MAMMARY-GLAND DEVELOPMENT; STIMULATING FACTOR-I;

CD8(+) T-CELLS; PANCREATIC-CANCER; MYELOID CELLS; BREAST-CANCER;

TIE2-EXPRESSING MONOCYTES; ACTIVATED MONOCYTES; PROMOTE EXPANSION

AB The tumor-promoting role of macrophages has been firmly established in most cancer types. However, macrophage identity has been a matter of debate, since several levels of complexity result in considerable macrophage heterogeneity. Ontogenically, tissue-resident macrophages derive from yolk sac progenitors which either directly or via a fetal liver monocyte intermediate differentiate into distinct macrophage types during embryogenesis and are maintained throughout life, while a disruption of the steady state mobilizes monocytes and instructs the formation of monocyte-derived macrophages. Histologically, the macrophage phenotype is heavily influenced by the tissue microenvironment resulting in molecularly and functionally distinct macrophages in distinct organs. Finally, a change in the tissue microenvironment as a result of infectious or sterile inflammation instructs different modes of macrophage activation. These considerations are relevant in the context of tumors, which can be considered as sites of chronic sterile inflammation encompassing subregions with distinct environmental conditions (for example, hypoxic versus normoxic). Here, we discuss existing evidence on the role of macrophage subpopulations in steady state tissue and primary tumors of the breast lung, pancreas, brain and liver. (C) 2015 Elsevier B.V. All rights reserved.

C1 [Lahmar, Qods; Keirsse, Jiri; Laoui, Damya; Movahedi, Kiavash; Van Overmeire, Eva; Van Ginderachter, Jo A.] VIB, Myeloid Cell Immunol Lab, Brussels, Belgium.

[Lahmar, Qods; Keirsse, Jiri; Laoui, Damya; Movahedi, Kiavash; Van Overmeire, Eva; Van Ginderachter, Jo A.] Vrije Univ Brussel, Lab Cellular & Mol Immunol, Bldg E8,Pleinlaan 2, B-1050 Brussels, Belgium.

C3 Flanders Institute for Biotechnology (VIB); Vrije Universiteit Brussel

RP Van Ginderachter, JA (corresponding author), Vrije Univ Brussel, Lab Cellular & Mol Immunol, Bldg E8,Pleinlaan 2, B-1050 Brussels, Belgium.

EM jvangind@vub.ac.be

RI Laoui, Damya/J-7652-2019; Movahedi, Kiavash/AAQ-4942-2020; Van

Ginderachter, Jo/H-5473-2013

OI Movahedi, Kiavash/0000-0002-0826-4399; Laoui, Damya/0000-0002-3373-1403;

Van Ginderachter, Jo/0000-0002-4442-7474

CR Al-Shibli K, 2009, HISTOPATHOLOGY, V55, P301, DOI 10.1111/j.1365-2559.2009.03379.x

Alliot F, 1999, DEV BRAIN RES, V117, P145, DOI 10.1016/S0165-3806(99)00113-3

[Anonymous], CA CANC J CLIN

Arnold SA, 2010, DIS MODEL MECH, V3, P57, DOI 10.1242/dmm.003228

Bain CC, 2014, NAT IMMUNOL, V15, P929, DOI 10.1038/ni.2967

Banaei-Bouchareb L, 2004, J LEUKOCYTE BIOL, V76, P359, DOI 10.1189/jlb.1103591

Beatty GL, 2013, CLIN CANCER RES, V19, P6286, DOI 10.1158/1078-0432.CCR-13-1320

Beatty GL, 2011, SCIENCE, V331, P1612, DOI 10.1126/science.1198443

Bedoret D, 2009, J CLIN INVEST, V119, P3723, DOI 10.1172/JCI39717

BERTRAM JS, 1980, CANCER LETT, V11, P63, DOI 10.1016/0304-3835(80)90130-5

Bingle L, 2002, J PATHOL, V196, P254, DOI 10.1002/path.1027

Bonapace L, 2014, NATURE, V515, DOI 10.1038/nature13862

Bond-Smith G, 2012, BRIT MED J, V344, DOI 10.1136/bmj.e2476

Budhu A, 2006, CANCER CELL, V10, P99, DOI 10.1016/j.ccr.2006.06.016

Butovsky O, 2014, NAT NEUROSCI, V17, P131, DOI 10.1038/nn.3599

Campbell MJ, 2011, BREAST CANCER RES TR, V128, P703, DOI 10.1007/s10549-010-1154-y

Capece D, 2013, BIOMED RES INT, V2013, DOI 10.1155/2013/187204

Carus A, 2013, LUNG CANCER, V81, P130, DOI 10.1016/j.lungcan.2013.03.003

Casazza A, 2013, CANCER CELL, V24, P695, DOI 10.1016/j.ccr.2013.11.007

Cavel O, 2012, CANCER RES, V72, P5733, DOI 10.1158/0008-5472.CAN-12-0764

Chen JJW, 2005, J CLIN ONCOL, V23, P953, DOI 10.1200/JCO.2005.12.172

Chittezhath M, 2014, IMMUNITY, V41, P815, DOI 10.1016/j.immuni.2014.09.014

Chung FT, 2012, INT J CANCER, V131, pE227, DOI 10.1002/ijc.27403

Clark CE, 2007, CANCER RES, V67, P9518, DOI 10.1158/0008-5472.CAN-07-0175

Condeelis J, 2006, CELL, V124, P263, DOI 10.1016/j.cell.2006.01.007

Cortez-Retamozo V, 2013, IMMUNITY, V38, P296, DOI 10.1016/j.immuni.2012.10.015

Cortez-Retamozo V, 2012, P NATL ACAD SCI USA, V109, P2491, DOI 10.1073/pnas.1113744109

Coussens LM, 2011, CSH PERSPECT BIOL, V3, DOI 10.1101/cshperspect.a003285

Dai FQ, 2010, BMC CANCER, V10, DOI 10.1186/1471-2407-10-220

De Palma M, 2005, CANCER CELL, V8, P211, DOI 10.1016/j.ccr.2005.08.002

De Palma M, 2008, CANCER CELL, V14, P299, DOI 10.1016/j.ccr.2008.09.004

De Palma M, 2013, CANCER CELL, V23, P277, DOI 10.1016/j.ccr.2013.02.013

de Visser KE, 2006, NAT REV CANCER, V6, P24, DOI 10.1038/nrc1782

DeNardo DG, 2007, BREAST CANCER RES, V9, DOI 10.1186/bcr1746

DeNardo DG, 2011, CANCER DISCOV, V1, P54, DOI 10.1158/2159-8274.CD-10-0028

DeSantis C, 2011, CA-CANCER J CLIN, V61, P409, DOI 10.3322/caac.20134

Domagala-Kulawik J, 2003, RESPIRATION, V70, P43, DOI 10.1159/000068414

Durafourt BA, 2012, GLIA, V60, P717, DOI 10.1002/glia.22298

Egeblad M, 2008, DIS MODEL MECH, V1, P155, DOI 10.1242/dmm.000596

Epelman S, 2014, IMMUNITY, V41, P21, DOI 10.1016/j.immuni.2014.06.013

Epelman S, 2014, IMMUNITY, V40, P91, DOI 10.1016/j.immuni.2013.11.019

Forget MA, 2014, PLOS ONE, V9, DOI 10.1371/journal.pone.0098623

Franklin RA, 2014, SCIENCE, V344, P921, DOI 10.1126/science.1252510

Gabrusiewicz K, 2011, PLOS ONE, V6, DOI 10.1371/journal.pone.0023902

Galarneau H, 2007, CANCER RES, V67, P8874, DOI 10.1158/0008-5472.CAN-07-0177

Gautier EL, 2012, NAT IMMUNOL, V13, P1118, DOI 10.1038/ni.2419

Geutskens SB, 2005, J LEUKOCYTE BIOL, V78, P845, DOI 10.1189/jlb.1004624

Ginhoux F, 2010, SCIENCE, V330, P841, DOI 10.1126/science.1194637

Gironella M, 2013, CANCER RES, V73, P5682, DOI 10.1158/0008-5472.CAN-12-3057

Glass R, 2014, ACTA NEUROPATHOL, V128, P347, DOI 10.1007/s00401-014-1274-2

Goldstraw P, 2011, LANCET, V378, P1727, DOI 10.1016/S0140-6736(10)62101-0

Goswami S, 2005, CANCER RES, V65, P5278, DOI 10.1158/0008-5472.CAN-04-1853

Gouon-Evans V, 2002, BREAST CANCER RES, V4, P155, DOI 10.1186/bcr441

Gouon-Evans V, 2000, DEVELOPMENT, V127, P2269

Guilliams M, 2013, J EXP MED, V210, P1977, DOI 10.1084/jem.20131199

Gyorki DE, 2009, BREAST CANCER RES, V11, DOI 10.1186/bcr2353

Ham M, 2013, ARCH PHARM RES, V36, P1419, DOI 10.1007/s12272-013-0271-7

Hanahan D, 2011, CELL, V144, P646, DOI 10.1016/j.cell.2011.02.013

Hashimoto D, 2013, IMMUNITY, V38, P792, DOI 10.1016/j.immuni.2013.04.004

Helm O, 2014, INT J CANCER, V135, P843, DOI 10.1002/ijc.28736

Hermano E, 2014, JNCI-J NATL CANCER I, V106, DOI 10.1093/jnci/dju332

HICKEY WF, 1988, SCIENCE, V239, P290, DOI 10.1126/science.3276004

HICKEY WF, 1992, J NEUROPATH EXP NEUR, V51, P246, DOI 10.1097/00005072-199205000-00002

Hickman SE, 2013, NAT NEUROSCI, V16, P1896, DOI 10.1038/nn.3554

Hirayama S, 2012, J THORAC ONCOL, V7, P1790, DOI 10.1097/JTO.0b013e3182745968

Hoeffel G, 2015, IMMUNITY, V42, P665, DOI 10.1016/j.immuni.2015.03.011

Hoeffel G, 2012, J EXP MED, V209, P1167, DOI 10.1084/jem.20120340

Ingman WV, 2006, DEV DYNAM, V235, P3222, DOI 10.1002/dvdy.20972

Ino Y, 2013, BRIT J CANCER, V108, P914, DOI 10.1038/bjc.2013.32

Jakubzick C, 2013, IMMUNITY, V39, P599, DOI 10.1016/j.immuni.2013.08.007

Jenne CN, 2013, NAT IMMUNOL, V14, P996, DOI 10.1038/ni.2691

Ji JD, 2013, UROLOGY, V82, DOI 10.1016/j.urology.2013.05.026

Kang TW, 2011, NATURE, V479, P547, DOI 10.1038/nature10599

Karnevi E, 2014, IMMUNOL CELL BIOL, V92, P543, DOI 10.1038/icb.2014.22

Kawai O, 2008, CANCER-AM CANCER SOC, V113, P1387, DOI 10.1002/cncr.23712

Kedrin D, 2008, NAT METHODS, V5, P1019, DOI 10.1038/nmeth.1269

Kim DW, 2008, BRIT J CANCER, V98, P1118, DOI 10.1038/sj.bjc.6604256

KLEIHUES P, 1995, GLIA, V15, P211, DOI 10.1002/glia.440150303

Kolios G, 2006, WORLD J GASTROENTERO, V12, P7413, DOI 10.3748/wjg.v12.i46.7413

Komohara Y, 2008, J PATHOL, V216, P15, DOI 10.1002/path.2370

Kuang DM, 2010, J IMMUNOL, V185, P1544, DOI 10.4049/jimmunol.0904094

Kuang DM, 2010, HEPATOLOGY, V51, P154, DOI 10.1002/hep.23291

Kuang DM, 2009, J EXP MED, V206, P1327, DOI 10.1084/jem.20082173

Kurahara H, 2012, ANN SURG ONCOL, V19, P2264, DOI 10.1245/s10434-012-2263-0

Kurahara H, 2011, J SURG RES, V167, pE211, DOI 10.1016/j.jss.2009.05.026

Landis SH, 1999, CA-CANCER J CLIN, V49, P8, DOI 10.3322/canjclin.49.1.8

Landsman L, 2007, J IMMUNOL, V178, P2000, DOI 10.4049/jimmunol.178.4.2000

Laoui D., 2011, TUMOR ASS MACROPHAGE, P861

Laoui D, 2014, CANCER RES, V74, P24, DOI 10.1158/0008-5472.CAN-13-1196

Lewis CE, 2006, CANCER RES, V66, P605, DOI 10.1158/0008-5472.CAN-05-4005

Lin EY, 2007, CANCER RES, V67, P5064, DOI 10.1158/0008-5472.CAN-07-0912

Liou GY, 2015, CANCER DISCOV, V5, P52, DOI 10.1158/2159-8290.CD-14-0474

Liou GY, 2013, J CELL BIOL, V202, P563, DOI 10.1083/jcb.201301001

Liu CY, 2013, LAB INVEST, V93, P844, DOI 10.1038/labinvest.2013.69

Liu L, 2012, PLOS ONE, V7

Ma JL, 2010, BMC CANCER, V10, DOI 10.1186/1471-2407-10-112

Maeda S, 2005, CELL, V121, P977, DOI 10.1016/j.cell.2005.04.014

Mantovani A, 2002, TRENDS IMMUNOL, V23, P549, DOI 10.1016/S1471-4906(02)02302-5

Mantovani A, 2007, BREAST, V16, pS27, DOI 10.1016/j.breast.2007.07.013

Markovic DS, 2009, P NATL ACAD SCI USA, V106, P12530, DOI 10.1073/pnas.0804273106

Matsubara T, 2013, HEPATOLOGY, V57, P1416, DOI 10.1002/hep.25965

Mazzieri R, 2011, CANCER CELL, V19, P512, DOI 10.1016/j.ccr.2011.02.005

Misharin AV, 2013, AM J RESP CELL MOL, V49, P503, DOI 10.1165/rcmb.2013-0086MA

Mitchem JB, 2013, CANCER RES, V73, P1128, DOI 10.1158/0008-5472.CAN-12-2731

Molawi K, 2014, J EXP MED, V211, P2151, DOI 10.1084/jem.20140639

Movahedi K, 2012, CANCER RES, V72, P4165, DOI 10.1158/0008-5472.CAN-11-2994

Movahedi K, 2010, CANCER RES, V70, P5728, DOI 10.1158/0008-5472.CAN-09-4672

Müller A, 2015, INT J CANCER, V137, P278, DOI 10.1002/ijc.29379

Murdoch C, 2008, NAT REV CANCER, V8, P618, DOI 10.1038/nrc2444

Nakasone ES, 2012, CANCER CELL, V21, P488, DOI 10.1016/j.ccr.2012.02.017

Naugler WE, 2007, SCIENCE, V317, P121, DOI 10.1126/science.1140485

Nguyen AV, 2002, DEV BIOL, V247, P11, DOI 10.1006/dbio.2002.0669

Nimmerjahn A, 2005, SCIENCE, V308, P1314, DOI 10.1126/science.1110647

Noy R, 2014, IMMUNITY, V41, P49, DOI 10.1016/j.immuni.2014.06.010

Ohri CM, 2009, EUR RESPIR J, V33, P118, DOI 10.1183/09031936.00065708

Ohtaki Y, 2010, J THORAC ONCOL, V5, P1507, DOI 10.1097/JTO.0b013e3181eba692

Omuro A, 2013, JAMA-J AM MED ASSOC, V310, P1842, DOI 10.1001/jama.2013.280319

Perdiguero EG, 2015, NATURE, V518, P547, DOI 10.1038/nature13989

Perdiguero EG, 2013, GLIA, V61, P112, DOI 10.1002/glia.22393

Pollard JW, 2009, NAT REV IMMUNOL, V9, P259, DOI 10.1038/nri2528

POLLARD JW, 1994, P NATL ACAD SCI USA, V91, P9312, DOI 10.1073/pnas.91.20.9312

Pong WW, 2013, PLOS ONE, V8, DOI 10.1371/journal.pone.0077571

Pouniotis DS, 2006, CLIN EXP IMMUNOL, V143, P363, DOI 10.1111/j.1365-2249.2006.02998.x

Prinz M, 2014, NAT REV NEUROSCI, V15, P300, DOI 10.1038/nrn3722

Prinz M, 2011, NAT NEUROSCI, V14, P1227, DOI 10.1038/nn.2923

Prinz M, 2011, GLIA, V59, P177, DOI 10.1002/glia.21104

Pucci F, 2009, BLOOD, V114, P901, DOI 10.1182/blood-2009-01-200931

Pyonteck SM, 2013, NAT MED, V19, P1264, DOI 10.1038/nm.3337

Qian BZ, 2010, CELL, V141, P39, DOI 10.1016/j.cell.2010.03.014

Remark R, 2015, AM J RESP CRIT CARE, V191, P377, DOI 10.1164/rccm.201409-1671PP

Robinson BD, 2009, CLIN CANCER RES, V15, P2433, DOI 10.1158/1078-0432.CCR-08-2179

Rolny C, 2011, CANCER CELL, V19, P31, DOI 10.1016/j.ccr.2010.11.009

Ruffell B, 2014, CANCER CELL, V26, P623, DOI 10.1016/j.ccell.2014.09.006

Ruffell B, 2012, TRENDS IMMUNOL, V33, P119, DOI 10.1016/j.it.2011.12.001

Ruffell B, 2012, P NATL ACAD SCI USA, V109, P2796, DOI 10.1073/pnas.1104303108

Ryan DP, 2014, NEW ENGL J MED, V371, P1039, DOI 10.1056/NEJMra1404198

Salter MW, 2014, CELL, V158, P15, DOI 10.1016/j.cell.2014.06.008

Sarkar S, 2014, NAT NEUROSCI, V17, P46, DOI 10.1038/nn.3597

Sawanobori Y, 2008, BLOOD, V111, P5457, DOI 10.1182/blood-2008-01-136895

Schneider C, 2012, GUT, V61, P1733, DOI 10.1136/gutjnl-2011-301116

Schulz C, 2012, SCIENCE, V336, P86, DOI 10.1126/science.1219179

Scott CL, 2014, IMMUNOL REV, V262, P9, DOI 10.1111/imr.12220

Shibata Y, 2001, IMMUNITY, V15, P557, DOI 10.1016/S1074-7613(01)00218-7

Sieweke MH, 2013, SCIENCE, V342, P946, DOI 10.1126/science.1242974

Sliwa M, 2007, BRAIN, V130, P476, DOI 10.1093/brain/awl263

Squadrito ML, 2012, CELL REPORTS, V1, P141, DOI 10.1016/j.celrep.2011.12.005

Szulzewsky F, 2015, PLOS ONE, V10, DOI 10.1371/journal.pone.0116644

Tacke F, 2014, J HEPATOL, V60, P1090, DOI 10.1016/j.jhep.2013.12.025

Takanami I, 1999, ONCOLOGY-BASEL, V57, P138, DOI 10.1159/000012021

Toi M, 1999, CLIN CANCER RES, V5, P1131

Toomey D, 2003, INT J CANCER, V103, P408, DOI 10.1002/ijc.10836

Tremblay ME, 2011, J NEUROSCI, V31, P16064, DOI 10.1523/JNEUROSCI.4158-11.2011

Tymoszuk P, 2014, EUR J IMMUNOL, V44, P2247, DOI 10.1002/eji.201344304

Van Gassen N, 2015, STEM CELL TRANSL MED, V4, P555, DOI 10.5966/sctm.2014-0272

Van Gassen N, 2015, EUR J IMMUNOL, V45, P1482, DOI 10.1002/eji.201445013

Van Overmeire E, 2014, EUR J IMMUNOL, V44, P2238, DOI 10.1002/eji.201444870

VANFURTH R, 1968, J EXP MED, V128, P415, DOI 10.1084/jem.128.3.415

Venneri MA, 2007, BLOOD, V109, P5276, DOI 10.1182/blood-2006-10-053504

Villeneuve J, 2005, CANCER RES, V65, P3928, DOI 10.1158/0008-5472.CAN-04-3612

Vucur M, 2010, ONCOTARGET, V1, P373

Wan SS, 2014, GASTROENTEROLOGY, V147, P1393, DOI 10.1053/j.gastro.2014.08.039

Wang YM, 2012, NAT IMMUNOL, V13, P753, DOI 10.1038/ni.2360

Weizman N, 2014, ONCOGENE, V33, P3812, DOI 10.1038/onc.2013.357

Welsh TJ, 2005, J CLIN ONCOL, V23, P8959, DOI 10.1200/JCO.2005.01.4910

WIKTORJEDRZEJCZAK W, 1990, P NATL ACAD SCI USA, V87, P4828, DOI 10.1073/pnas.87.12.4828

Wolfgang CL, 2013, CA-CANCER J CLIN, V63, P318, DOI 10.3322/caac.21190

Wu J, 2012, CANCER RES, V72, P3977, DOI 10.1158/0008-5472.CAN-12-0938

Wu K, 2009, CANCER RES, V69, P8067, DOI 10.1158/0008-5472.CAN-09-0901

Wyckoff JB, 2007, CANCER RES, V67, P2649, DOI 10.1158/0008-5472.CAN-06-1823

Xiao XW, 2014, P NATL ACAD SCI USA, V111, pE1211, DOI 10.1073/pnas.1321347111

Yamasaki R, 2014, J EXP MED, V211, P1533, DOI 10.1084/jem.20132477

Yan W., 2015, GUT

Yang WW, 2012, J BIOL CHEM, V287, P40140, DOI 10.1074/jbc.M112.348763

Yeung OWH, 2015, J HEPATOL, V62, P607, DOI 10.1016/j.jhep.2014.10.029

Yona S, 2013, IMMUNITY, V38, P79, DOI 10.1016/j.immuni.2012.12.001

Yoshikawa K, 2012, CANCER SCI, V103, P2012, DOI 10.1111/j.1349-7006.2012.02411.x

Zeni E, 2007, EUR RESPIR J, V30, P627, DOI 10.1183/09031936.00129306

Zhai HY, 2011, GLIA, V59, P472, DOI 10.1002/glia.21117

Zhang BC, 2011, MED ONCOL, V28, P1447, DOI 10.1007/s12032-010-9638-5

Zhang BC, 2011, CLINICS, V66, P1879, DOI 10.1590/S1807-59322011001100006

Zhang J, 2014, ONCOTARGET, V5, P9664, DOI 10.18632/oncotarget.1856

Zhou J, 2009, INT J CANCER, V125, P1640, DOI 10.1002/ijc.24556

Zhou WC, 2015, NAT CELL BIOL, V17, P170, DOI 10.1038/ncb3090

Zhu Y, 2014, CANCER RES, V74, P5057, DOI 10.1158/0008-5472.CAN-13-3723

NR 184

TC 120

Z9 138

U1 2

U2 54

PU ELSEVIER SCIENCE BV

PI AMSTERDAM

PA PO BOX 211, 1000 AE AMSTERDAM, NETHERLANDS

SN 0304-419X

EI 1879-2561

J9 BBA-REV CANCER

JI Biochim. Biophys. Acta-Rev. Cancer

PD JAN

PY 2016

VL 1865

IS 1

SI SI

BP 23

EP 34

DI 10.1016/j.bbcan.2015.06.009

PG 12

WC Biochemistry & Molecular Biology; Biophysics; Oncology

WE Science Citation Index Expanded (SCI-EXPANDED)

SC Biochemistry & Molecular Biology; Biophysics; Oncology

GA DF4YT

UT WOS:000371359200004

PM 26145884

DA 2025-02-07

ER

PT J

AU Ramos, RN

Missolo-Koussou, Y

Gerber-Ferder, Y

Bromley, CP

Bugatti, M

Núñez, NG

Boari, JT

Richer, W

Menger, L

Denizeau, J

Sedlik, C

Caudana, P

Kotsias, F

Niborski, LL

Viel, S

Bohec, M

Lameiras, S

Baulande, S

Lesage, L

Nicolas, A

Meseure, D

Vincent-Salomon, A

Reyal, F

Dutertre, CA

Ginhoux, F

Vimeux, L

Donnadieu, E

Buttard, B

Galon, J

Zelenay, S

Vermi, W

Guermonprez, P

Piaggio, E

Helft, J

AF Ramos, Rodrigo Nalio

Missolo-Koussou, Yoann

Gerber-Ferder, Yohan

Bromley, Christian P.

Bugatti, Mattia

Nunez, Nicolas Gonzalo

Boari, Jimena Tosello

Richer, Wilfrid

Menger, Laurie

Denizeau, Jordan

Sedlik, Christine

Caudana, Pamela

Kotsias, Fiorella

Niborski, Leticia L.

Viel, Sophie

Bohec, Mylene

Lameiras, Sonia

Baulande, Sylvain

Lesage, Laetitia

Nicolas, Andre

Meseure, Didier

Vincent-Salomon, Anne

Reyal, Fabien

Dutertre, Charles-Antoine

Ginhoux, Florent

Vimeux, Lene

Donnadieu, Emmanuel

Buttard, Benedicte

Galon, Jerome

Zelenay, Santiago

Vermi, William

Guermonprez, Pierre

Piaggio, Eliane

Helft, Julie

TI Tissue-resident FOLR2<SUP>+</SUP> macrophages associate with

CD8<SUP>+</SUP> T cell infiltration in human breast cancer

SO CELL

LA English

DT Article

ID TUMOR-ASSOCIATED MACROPHAGES; GENE-EXPRESSION; DENDRITIC CELLS;

R/BIOCONDUCTOR PACKAGE; INFLAMMATORY MONOCYTES; LYMPH-NODES;

POLARIZATION; CYTOMETRY; ORIGIN; METASTASIS

AB Macrophage infiltration is a hallmark of solid cancers, and overall macrophage infiltration correlates with lower patient survival and resistance to therapy. Tumor-associated macrophages, however, are phenotypically and functionally heterogeneous. Specific subsets of tumor-associated macrophage might be endowed with distinct roles on cancer progression and antitumor immunity. Here, we identify a discrete population of FOLR2(+) tissue-resident macrophages in healthy mammary gland and breast cancer primary tumors. FOLR2(+) macrophages localize in perivascular areas in the tumor stroma, where they interact with CD8(+) T cells. FOLR2(+) macrophages efficiently prime effector CD8(+) T cells ex vivo. The density of FOLR2(+) macrophages in tumors positively correlates with better patient survival. This study highlights specific roles for tumor-associated macrophage subsets and paves the way for subset-targeted therapeutic interventions in macrophages-based cancer therapies.

C1 [Ramos, Rodrigo Nalio; Missolo-Koussou, Yoann; Gerber-Ferder, Yohan; Nunez, Nicolas Gonzalo; Boari, Jimena Tosello; Richer, Wilfrid; Menger, Laurie; Denizeau, Jordan; Sedlik, Christine; Caudana, Pamela; Kotsias, Fiorella; Niborski, Leticia L.; Viel, Sophie; Vermi, William; Piaggio, Eliane; Helft, Julie] PSL Univ, Inst Curie Res Ctr, U932, INSERM, F-75005 Paris, France.

[Ramos, Rodrigo Nalio; Missolo-Koussou, Yoann; Gerber-Ferder, Yohan; Nunez, Nicolas Gonzalo; Boari, Jimena Tosello; Richer, Wilfrid; Denizeau, Jordan; Sedlik, Christine; Caudana, Pamela; Kotsias, Fiorella; Niborski, Leticia L.; Viel, Sophie; Piaggio, Eliane; Helft, Julie] PSL Univ, SiRIC, Translat Immunotherapy Team, F-75005 Paris, France.

[Bromley, Christian P.; Zelenay, Santiago] Univ Manchester, Canc Res UK Manchester Inst, Canc Inflammat & Immun Grp, Alderley Pk, Manchester, Lancs, England.

[Bugatti, Mattia] Univ Brescia, Dept Pathol, I-25123 Brescia, Italy.

[Vermi, William] Washington Univ, Dept Pathol & Immunol, Sch Med, St Louis, MO 63110 USA.

[Bohec, Mylene; Lameiras, Sonia; Baulande, Sylvain] PSL Univ, Inst Curie Res Ctr, Inst Curie Genom Excellence Platform, F-75005 Paris, France.

[Lesage, Laetitia; Nicolas, Andre; Meseure, Didier; Vincent-Salomon, Anne] PSL Univ, Inst Curie Hosp, Dept Pathol, F-75005 Paris, France.

[Reyal, Fabien] PSL Univ, Inst Curie Hosp, Dept Surg, F-75005 Paris, France.

[Dutertre, Charles-Antoine; Ginhoux, Florent] Univ Paris Saclay, Inst Gustave Roussy, INSERM, U1015, Villejuif, France.

[Ginhoux, Florent] Agcy Sci Technol & Res, Singapore Immunol Network, Singapore 138648, Singapore.

[Vimeux, Lene; Donnadieu, Emmanuel] Univ Paris, Inst Cochin, INSERM, CNRS,UMR 8104,U1016, F-75014 Paris, France.

[Buttard, Benedicte; Galon, Jerome] Univ Paris, Sorbonne Univ, Ctr Rech Cordeliers, Lab Integrat Canc Immunol,INSERM, Paris, France.

[Guermonprez, Pierre] Univ Paris, Ctr Inflammat Res, INSERM1149, CNRS,ERL8252, Paris, France.

[Ramos, Rodrigo Nalio] Univ Sao Paulo, Fac Med, Hosp Clin HCFMUSP,Dept Hematol & Cell Therapy, Lab Med Invest Pathogenesis & Directed Therapy On, Sao Paulo, Brazil.

[Ramos, Rodrigo Nalio] Inst DOr Ensino & Pesquisa, Sao Paulo, Brazil.

[Nunez, Nicolas Gonzalo] Univ Zurich, Inst Expt Immunol, Zurich, Switzerland.

C3 Universite PSL; UNICANCER; Institut Curie; Institut National de la Sante

et de la Recherche Medicale (Inserm); Universite PSL; University of

Manchester; Cancer Research UK; University of Brescia; Washington

University (WUSTL); UNICANCER; Universite PSL; Institut Curie;

UNICANCER; Universite PSL; Institut Curie; UNICANCER; Universite PSL;

Institut Curie; Universite Paris Saclay; UNICANCER; Gustave Roussy;

Institut National de la Sante et de la Recherche Medicale (Inserm);

Agency for Science Technology & Research (A*STAR); A*STAR - Singapore

Immunology Network (SIgN); Universite Paris Cite; Centre National de la

Recherche Scientifique (CNRS); CNRS - National Institute for Biology

(INSB); Institut National de la Sante et de la Recherche Medicale

(Inserm); Sorbonne Universite; Institut National de la Sante et de la

Recherche Medicale (Inserm); Universite Paris Cite; Universite Paris

Cite; Centre National de la Recherche Scientifique (CNRS); CNRS -

National Institute for Biology (INSB); Universidade de Sao Paulo;

University of Zurich

RP Helft, J (corresponding author), PSL Univ, Inst Curie Res Ctr, U932, INSERM, F-75005 Paris, France.; Helft, J (corresponding author), PSL Univ, SiRIC, Translat Immunotherapy Team, F-75005 Paris, France.

EM julie.helft@inserm.fr

RI Ginhoux, Florent/AFN-0385-2022; Dutertre, Charles Antoine/JRX-2224-2023;

Vincent-Salomon, Anne/GXV-2625-2022; Piaggio, Eliane/E-5260-2016;

Bugatti, Mattia/LIG-8178-2024; Viel, Sophie/D-1624-2018; Nalio Ramos,

Rodrigo/K-3553-2012

OI Bohec, Mylene/0000-0002-6530-596X; Dutertre,

Charles-Antoine/0000-0001-7950-3652; Bromley,

Christian/0000-0001-6203-6891; Zelenay, Santiago/0000-0002-6865-3978;

Nunez, Nicolas/0000-0003-3837-270X; Viel, Sophie/0000-0003-1354-7374;

Helft, Julie/0000-0002-2733-1972; Gerber-Ferder,

Yohan/0000-0001-9973-2196; Nalio Ramos, Rodrigo/0000-0003-1249-4733;

Missolo-Koussou, Yoann-Lionel/0000-0002-3815-3980; LESAGE,

LAETITIA/0000-0001-8592-2482; Tosello Boari, Jimena/0000-0003-1563-4420

FU SiRIC-Curie Program [INCa-DGOS-12554]; Labex DCBIOL [ANR-10-IDEX-0001-02

PSL, ANR-11-LABX-0043]; Fondation ARC [PA20181207706]; Ligue Nationale

contre le cancer [RS19/75-92]; Center of Clinical Investigation (CIC

IGR-Curie) [1428]; ITMO-CancerAviesan (Plan Cancer III) [ANR-10-EQPX-03,

ANR-10-INBS-09-08]; SiRIC-Curie program (SiRIC Grant) [INCa-DGOS-4654];

NIHR Manchester BRC; Cancer Research UK Manchester Institute Award

[A19258]; IDEX [ANR-18-IDEX-0001]; INCA [PL-BIO-ICR1]; [IG23179];

[ANR-17-CE11-0001-01]; BBSRC [BB/M029735/1] Funding Source: UKRI

FX We thank A.M. Lennon-Dumenil, J. Waterfall, C. Thery, P. Sirven, and F.

Benvenuti for helpful advice and reagents. E.P. team is supported by the

SiRIC-Curie Program (grant INCa-DGOS-12554) , the Labex DCBIOL

(ANR-10-IDEX-0001-02 PSL and ANR-11-LABX-0043) , the Fondation ARC

(PA20181207706) , the Ligue Nationale contre le cancer (RS19/75-92) ,

and the Center of Clinical Investigation (CIC IGR-Curie 1428) .

High-throughput sequencing was performed by the ICGex NGS platform of

the Institut Curie supported by the grants ANR-10-EQPX-03 and

ANR-10-INBS-09-08, by the ITMO-CancerAviesan (Plan Cancer III) , and by

the SiRIC-Curie program (SiRIC Grant INCa-DGOS-4654) . C.P.B. and S.Z.

are supported by the NIHR Man-chester BRC and by a Cancer Research UK

Manchester Institute Award (A19258) . W.V. is supported by the grant

IG23179, A.I.R.C. P.G. is supported by IDEX (ANR-18-IDEX-0001) , INCA

(PL-BIO-ICR1) , and ANR-17-CE11-0001-01.

CR Ali HR, 2014, ANN ONCOL, V25, P1536, DOI 10.1093/annonc/mdu191

Ali HR, 2016, PLOS MED, V13, DOI 10.1371/journal.pmed.1002194

[Anonymous], 2010, GENOME BIOL, DOI DOI 10.1186/gb-2010-11-10-r106

Arwert EN, 2018, CELL REP, V23, P1239, DOI 10.1016/j.celrep.2018.04.007

Azizi E, 2018, CELL, V174, P1293, DOI 10.1016/j.cell.2018.05.060

Becher B, 2014, NAT IMMUNOL, V15, P1181, DOI 10.1038/ni.3006

Becht E, 2016, GENOME BIOL, V17, DOI 10.1186/s13059-016-1070-5

Bindea G, 2009, BIOINFORMATICS, V25, P1091, DOI 10.1093/bioinformatics/btp101

Bonapace L, 2014, NATURE, V515, DOI 10.1038/nature13862

Bourdely P, 2020, IMMUNITY, V53, P335, DOI 10.1016/j.immuni.2020.06.002

Brummelman J, 2019, NAT PROTOC, V14, P1946, DOI 10.1038/s41596-019-0166-2

Butler A, 2018, NAT BIOTECHNOL, V36, P411, DOI 10.1038/nbt.4096

Casanova-Acebes M, 2021, NATURE, V595, P578, DOI 10.1038/s41586-021-03651-8

Cassetta L, 2019, CANCER CELL, V35, P588, DOI 10.1016/j.ccell.2019.02.009

Cassetta L, 2018, NAT REV DRUG DISCOV, V17, P887, DOI 10.1038/nrd.2018.169

Chakarov S, 2019, SCIENCE, V363, P1190, DOI 10.1126/science.aau0964

Chen X, 2012, CANCER INFORM, V11, P147, DOI 10.4137/CIN.S9983

Chow A, 2021, CANCER CELL, V39, P973, DOI 10.1016/j.ccell.2021.05.006

Coffelt SB, 2010, CANCER RES, V70, P5270, DOI 10.1158/0008-5472.CAN-10-0012

Colonna M, 2004, NAT IMMUNOL, V5, P1219, DOI 10.1038/ni1141

Curtis C, 2012, NATURE, V486, P346, DOI 10.1038/nature10983

Davie SA, 2007, TRANSGENIC RES, V16, P193, DOI 10.1007/s11248-006-9056-9

Dawson CA, 2020, NAT CELL BIOL, V22, P546, DOI 10.1038/s41556-020-0505-0

De Palma M, 2005, CANCER CELL, V8, P211, DOI 10.1016/j.ccr.2005.08.002

DeNardo DG, 2011, CANCER DISCOV, V1, P54, DOI 10.1158/2159-8274.CD-10-0028

Derti A, 2012, GENOME RES, V22, P1173, DOI 10.1101/gr.132563.111

Dutertre CA, 2019, IMMUNITY, V51, P573, DOI 10.1016/j.immuni.2019.08.008

Engblom C, 2016, NAT REV CANCER, V16, P447, DOI 10.1038/nrc.2016.54

Etzerodt A, 2020, J EXP MED, V217, DOI 10.1084/jem.20191869

Finak G, 2008, NAT MED, V14, P518, DOI 10.1038/nm1764

Finck R, 2013, CYTOM PART A, V83A, P483, DOI 10.1002/cyto.a.22271

Franklin RA, 2014, SCIENCE, V344, P921, DOI 10.1126/science.1252510

Gallina G, 2006, J CLIN INVEST, V116, P2777, DOI 10.1172/JCI28828

Gendoo DMA, 2016, BIOINFORMATICS, V32, P1097, DOI 10.1093/bioinformatics/btv693

Goldmann T, 2016, NAT IMMUNOL, V17, P797, DOI 10.1038/ni.3423

Gu ZG, 2016, BIOINFORMATICS, V32, P2847, DOI 10.1093/bioinformatics/btw313

Guilliams M, 2017, NAT REV IMMUNOL, V17, P451, DOI 10.1038/nri.2017.42

Han XP, 2018, CELL, V172, P1091, DOI 10.1016/j.cell.2018.02.001

Hanna RN, 2015, SCIENCE, V350, P985, DOI 10.1126/science.aac9407

Hugues S, 2004, NAT IMMUNOL, V5, P1235, DOI 10.1038/ni1134

Jäppinen N, 2019, NAT COMMUN, V10, DOI 10.1038/s41467-018-08065-1

Katzenelenbogen Y, 2020, CELL, V182, P872, DOI 10.1016/j.cell.2020.06.032

Leek RD, 1996, CANCER RES, V56, P4625

Lewis CE, 2006, CANCER RES, V66, P605, DOI 10.1158/0008-5472.CAN-05-4005

Lewis CE, 2016, CANCER CELL, V30, P18, DOI 10.1016/j.ccell.2016.05.017

Li TW, 2017, CANCER RES, V77, pE108, DOI 10.1158/0008-5472.CAN-17-0307

Lim HY, 2018, IMMUNITY, V49, P326, DOI [10.1016/j.immuni.2018.06.008, 10.1016/j.immuni.2018.12.009]

Lin EY, 2006, CANCER RES, V66, P11238, DOI 10.1158/0008-5472.CAN-06-1278

Lin EY, 2003, AM J PATHOL, V163, P2113, DOI 10.1016/S0002-9440(10)63568-7

Linde N, 2018, NAT COMMUN, V9, DOI 10.1038/s41467-017-02481-5

Love MI, 2014, GENOME BIOL, V15, DOI 10.1186/s13059-014-0550-8

Loyher PL, 2018, J EXP MED, V215, P2536, DOI 10.1084/jem.20180534

Mantovani A, 2004, TRENDS IMMUNOL, V25, P677, DOI 10.1016/j.it.2004.09.015

Mantovani A, 2002, TRENDS IMMUNOL, V23, P549, DOI 10.1016/S1471-4906(02)02302-5

Mantovani A, 2017, NAT REV CLIN ONCOL, V14, P399, DOI 10.1038/nrclinonc.2016.217

Mebius RE, 2005, NAT REV IMMUNOL, V5, P606, DOI 10.1038/nri1669

Mempel TR, 2004, NATURE, V427, P154, DOI 10.1038/nature02238

Molgora M, 2020, CELL, V182, P886, DOI 10.1016/j.cell.2020.07.013

Murray PJ, 2014, IMMUNITY, V41, P14, DOI 10.1016/j.immuni.2014.06.008

Newell EW, 2012, IMMUNITY, V36, P142, DOI 10.1016/j.immuni.2012.01.002

Núñez NG, 2020, NAT COMMUN, V11, DOI 10.1038/s41467-020-17046-2

Pagès F, 2018, LANCET, V391, P2128, DOI 10.1016/S0140-6736(18)30789-X

Peranzoni E, 2018, P NATL ACAD SCI USA, V115, pE4041, DOI 10.1073/pnas.1720948115

Qian BZ, 2011, NATURE, V475, P222, DOI 10.1038/nature10138

Ramos RN, 2020, CLIN TRANSL IMMUNOL, V9, DOI 10.1002/cti2.1108

Ries CH, 2014, CANCER CELL, V25, P846, DOI 10.1016/j.ccr.2014.05.016

Ruffell B, 2015, CANCER CELL, V27, P462, DOI 10.1016/j.ccell.2015.02.015

Ruffell B, 2014, CANCER CELL, V26, P623, DOI 10.1016/j.ccell.2014.09.006

Ruffell B, 2012, P NATL ACAD SCI USA, V109, P2796, DOI 10.1073/pnas.1104303108

Samaniego R, 2014, J LEUKOCYTE BIOL, V95, P797, DOI 10.1189/jlb.0613345

Sharma A, 2020, CELL, V183, P377, DOI 10.1016/j.cell.2020.08.040

Stuart T, 2019, CELL, V177, P1888, DOI 10.1016/j.cell.2019.05.031

Thomas JR, 2021, J EXP MED, V218, DOI 10.1084/jem.20200891

Thorsson V, 2019, IMMUNITY, V51, P411, DOI [10.1016/j.immuni.2019.08.004, 10.1016/j.immuni.2018.03.023]

Utz SG, 2020, CELL, V181, P557, DOI 10.1016/j.cell.2020.03.021

Van Gassen S, 2015, CYTOM PART A, V87A, P636, DOI 10.1002/cyto.a.22625

Villani AC, 2017, SCIENCE, V356, DOI 10.1126/science.aah4573

Wang Y, 2020, ELIFE, V9, DOI 10.7554/eLife.57438

Wang YX, 2005, LANCET, V365, P671, DOI 10.1016/S0140-6736(05)17947-1

Yu GC, 2016, MOL BIOSYST, V12, P477, DOI 10.1039/c5mb00663e

Yuan Z. -Y., 2014, HIGH INFILTRATION TU

Zhao XX, 2017, ONCOTARGET, V8, P30576, DOI 10.18632/oncotarget.15736

Zhu Y, 2017, IMMUNITY, V47, P323, DOI 10.1016/j.immuni.2017.07.014

NR 83

TC 245

Z9 250

U1 33

U2 206

PU CELL PRESS

PI CAMBRIDGE

PA 50 HAMPSHIRE ST, FLOOR 5, CAMBRIDGE, MA 02139 USA

SN 0092-8674

EI 1097-4172

J9 CELL

JI Cell

PD MAR 31

PY 2022

VL 185

IS 7

BP 1189

EP +

DI 10.1016/j.cell.2022.02.021

EA MAR 2022

PG 45

WC Biochemistry & Molecular Biology; Cell Biology

WE Science Citation Index Expanded (SCI-EXPANDED)

SC Biochemistry & Molecular Biology; Cell Biology

GA 0V1VT

UT WOS:000788133700001

PM 35325594

OA Bronze

HC Y

HP N

DA 2025-02-07

ER

PT J

AU Mukaida, N

Nosaka, T

Nakamoto, Y

Baba, T

AF Mukaida, Naofumi

Nosaka, Takuto

Nakamoto, Yasunari

Baba, Tomohisa

TI Lung Macrophages: Multifunctional Regulator Cells for Metastatic Cells

SO INTERNATIONAL JOURNAL OF MOLECULAR SCIENCES

LA English

DT Review

DE alveolar macrophage; classical monocyte; interstitial macrophage;

metastasis-associated macrophage; patrolling monocyte; perivascular

macrophage

ID TUMOR-ASSOCIATED MACROPHAGES; TISSUE-RESIDENT MACROPHAGES; BREAST-CANCER

METASTASIS; ALVEOLAR MACROPHAGES; MYELOID CELLS; YOLK-SAC; MONOCYTES;

DIFFERENTIATION; THERAPY; ACTIVATION

AB Metastasis is responsible for most of the cancer-associated deaths and proceeds through multiple steps. Several lines of evidence have established an indispensable involvement of macrophages present at the primary tumor sites in various steps of metastasis, from primary tumor growth to its intravasation into circulation. The lungs encompass a large, dense vascular area and, therefore, are vulnerable to metastasis, particularly, hematogenous ones arising from various types of neoplasms. Lung tissues constitutively contain several types of tissue-resident macrophages and circulating monocytes to counteract potentially harmful exogenous materials, which directly reach through the airway. Recent advances have provided an insight into the ontogenetic, phenotypic, and functional heterogeneity of these lung macrophage and monocyte populations, under resting and inflammatory conditions. In this review, we discuss the ontogeny, trafficking dynamics, and functions of these pulmonary macrophages and monocytes and their potential roles in lung metastasis and measures to combat lung metastasis by targeting these populations.

C1 [Mukaida, Naofumi; Nosaka, Takuto; Baba, Tomohisa] Kanazawa Univ, Canc Res Inst, Div Mol Bioregulat, Kakuma Machi, Kanazawa, Ishikawa 9201192, Japan.

[Nosaka, Takuto; Nakamoto, Yasunari] Univ Fukui, Fac Med Sci, Dept Internal Med 2, Fukui 9101193, Japan.

C3 Kanazawa University; University of Fukui

RP Mukaida, N (corresponding author), Kanazawa Univ, Canc Res Inst, Div Mol Bioregulat, Kakuma Machi, Kanazawa, Ishikawa 9201192, Japan.

EM mukaida@staff.kanazawa-u.ac.jp; nosat@u-fukui.ac.jp;

nakamoto-med2@med.u-fukui.ac.jp; sergenti@staff.kanazawa-u.ac.jp

RI Nosaka, Takuto/ABB-5695-2021; Mukaida, Naofumi/D-7623-2011

OI Mukaida, Naofumi/0000-0002-4193-1851; Nosaka, Takuto/0000-0002-1929-7305

FU Research Program on the Innovative Development and Application for New

Drugs for Hepatitis B from the Japan Agency for Medical Research and

Development (AMED) [17fk0310116h0001]; Extramural Collaborative Research

Grant of Cancer Research Institute, Kanazawa University

FX This work is supported partially by Research Program on the Innovative

Development and Application for New Drugs for Hepatitis B

(17fk0310116h0001) from the Japan Agency for Medical Research and

Development (AMED) (N.M., Y.N.) and Extramural Collaborative Research

Grant of Cancer Research Institute, Kanazawa University (N.M., Y.N.).

CR Baker AD, 2010, J LIPID RES, V51, P1325, DOI 10.1194/jlr.M001651

Bedoret D, 2009, J CLIN INVEST, V119, P3723, DOI 10.1172/JCI39717

Ben-Aharon I, 2013, PLOS ONE, V8, DOI 10.1371/journal.pone.0070044

Bonapace L, 2014, NATURE, V515, DOI 10.1038/nature13862

Burkard-Mandel L, 2018, ONCOIMMUNOLOGY, V7, DOI 10.1080/2162402X.2017.1419115

Cannarile MA, 2017, J IMMUNOTHER CANCER, V5, DOI 10.1186/s40425-017-0257-y

Carlin LM, 2013, CELL, V153, P362, DOI 10.1016/j.cell.2013.03.010

Cassetta L, 2018, NAT REV DRUG DISCOV, V17, P887, DOI 10.1038/nrd.2018.169

Chen PW, 2017, P NATL ACAD SCI USA, V114, P580, DOI 10.1073/pnas.1614035114

Cohen M, 2018, CELL, V175, P1031, DOI 10.1016/j.cell.2018.09.009

Connelly L, 2011, BREAST CANCER RES, V13, DOI 10.1186/bcr2935

Dhupkar P, 2018, CANCER MED-US, V7, P2654, DOI 10.1002/cam4.1518

DINNEY CPN, 1991, CANCER RES, V51, P3741

DRANOFF G, 1994, SCIENCE, V264, P713, DOI 10.1126/science.8171324

Ferjancic S, 2013, BLOOD, V121, P3289, DOI 10.1182/blood-2012-08-449819

FIDLER IJ, 1994, CELL, V79, P185, DOI 10.1016/0092-8674(94)90187-2

Geissmann F, 2003, IMMUNITY, V19, P71, DOI 10.1016/S1074-7613(03)00174-2

Gibbings SL, 2015, BLOOD, V126, P1357, DOI 10.1182/blood-2015-01-624809

Gil-Bernabé AM, 2012, BLOOD, V119, P3164, DOI 10.1182/blood-2011-08-376426

Guilliams M, 2013, J EXP MED, V210, P1977, DOI 10.1084/jem.20131199

Hanna RN, 2015, SCIENCE, V350, P985, DOI 10.1126/science.aac9407

Hanna RN, 2011, NAT IMMUNOL, V12, P778, DOI 10.1038/ni.2063

Harper KL, 2016, NATURE, V540, P588, DOI 10.1038/nature20609

Hiratsuka S, 2002, CANCER CELL, V2, P289, DOI 10.1016/S1535-6108(02)00153-8

Hüsemann Y, 2008, CANCER CELL, V13, P58, DOI 10.1016/j.ccr.2007.12.003

Izquierdo HM, 2018, CELL REP, V24, P1738, DOI 10.1016/j.celrep.2018.07.034

Joshi N, 2018, CELL IMMUNOL, V330, P86, DOI 10.1016/j.cellimm.2018.01.005

Jung K, 2017, J CLIN INVEST, V127, P3039, DOI 10.1172/JCI93182

KESSLER R, 1994, AM J RESP CELL MOL, V10, P202, DOI 10.1165/ajrcmb.10.2.8110475

Kitamura T, 2018, FRONT IMMUNOL, V8, DOI 10.3389/fimmu.2017.02004

Kitamura T, 2015, J EXP MED, V212, P1043, DOI 10.1084/jem.20141836

Lambert AW, 2017, CELL, V168, P670, DOI 10.1016/j.cell.2016.11.037

Langley RR, 2011, INT J CANCER, V128, P2527, DOI 10.1002/ijc.26031

Lewis CE, 2016, CANCER CELL, V30, P18, DOI 10.1016/j.ccell.2016.05.017

Li QS, 2018, J IMMUNOL, V201, P2842, DOI 10.4049/jimmunol.1800141

Liegeois M, 2018, CELL IMMUNOL, V330, P91, DOI 10.1016/j.cellimm.2018.02.001

Loyher PL, 2018, J EXP MED, V215, P2536, DOI 10.1084/jem.20180534

Machiels B, 2017, NAT IMMUNOL, V18, P1310, DOI 10.1038/ni.3857

Mantovani A, 2017, NAT REV CLIN ONCOL, V14, P399, DOI 10.1038/nrclinonc.2016.217

Mantovani A, 2010, CURR OPIN IMMUNOL, V22, P231, DOI 10.1016/j.coi.2010.01.009

Misharin AV, 2017, J EXP MED, V214, P2387, DOI 10.1084/jem.20162152

Nasser MW, 2015, CANCER RES, V75, P974, DOI 10.1158/0008-5472.CAN-14-2161

Ngambenjawong C, 2017, ADV DRUG DELIVER REV, V114, P206, DOI 10.1016/j.addr.2017.04.010

Nosaka T, 2018, J IMMUNOL, V200, P1839, DOI 10.4049/jimmunol.1700544

PAGET S, 1989, CANCER METAST REV, V8, P98

Perdiguero EG, 2015, NATURE, V518, P547, DOI 10.1038/nature13989

Plebanek MP, 2017, NAT COMMUN, V8, DOI 10.1038/s41467-017-01433-3

Qian BZ, 2015, J EXP MED, V212, P1433, DOI 10.1084/jem.20141555

Qian BZ, 2011, NATURE, V475, P222, DOI 10.1038/nature10138

Rhim AD, 2012, CELL, V148, P349, DOI 10.1016/j.cell.2011.11.025

Ruffell B, 2012, TRENDS IMMUNOL, V33, P119, DOI 10.1016/j.it.2011.12.001

Sabatel C, 2017, IMMUNITY, V46, P457, DOI 10.1016/j.immuni.2017.02.016

Said N, 2012, J CLIN INVEST, V122, P1503, DOI 10.1172/JCI61392

Said N, 2011, J CLIN INVEST, V121, P132, DOI 10.1172/JCI42912

Satoh T, 2017, NATURE, V541, P96, DOI 10.1038/nature20611

Serbina NV, 2008, ANNU REV IMMUNOL, V26, P421, DOI 10.1146/annurev.immunol.26.021607.090326

Serbina NV, 2003, IMMUNITY, V19, P59, DOI 10.1016/S1074-7613(03)00171-7

Sfondrini L, 2013, INT J CANCER, V133, P383, DOI 10.1002/ijc.28028

Shapouri-Moghaddam A, 2018, J CELL PHYSIOL, V233, P6425, DOI 10.1002/jcp.26429

Shibata Y, 2001, IMMUNITY, V15, P557, DOI 10.1016/S1074-7613(01)00218-7

Sommariva M, 2017, CELL IMMUNOL, V313, P52, DOI 10.1016/j.cellimm.2017.01.004

Stathopoulos GT, 2008, MOL CANCER RES, V6, P364, DOI 10.1158/1541-7786.MCR-07-0309

TAKAHASHI K, 1989, J LEUKOCYTE BIOL, V45, P87, DOI 10.1002/jlb.45.2.87

Tan SYS, 2016, DEVELOPMENT, V143, P1318, DOI 10.1242/dev.129122

TANGUAY S, 1994, CANCER RES, V54, P5882

Trikha P, 2016, ONCOGENE, V35, P3636, DOI 10.1038/onc.2015.429

Turajlic S, 2016, SCIENCE, V352, P169, DOI 10.1126/science.aaf2784

UTSUGI T, 1991, CANCER IMMUNOL IMMUN, V33, P375, DOI 10.1007/BF01741597

VANFURTH R, 1968, J EXP MED, V128, P415, DOI 10.1084/jem.128.3.415

Vasiljeva O, 2006, CANCER RES, V66, P5242, DOI 10.1158/0008-5472.CAN-05-4463

Weidle UH, 2016, CANCER GENOM PROTEOM, V13, P129

Wu Y, 2008, J IMMUNOL, V181, P6384, DOI 10.4049/jimmunol.181.9.6384

Yu YRA, 2016, AM J RESP CELL MOL, V54, P13, DOI 10.1165/rcmb.2015-0146OC

Zabuawala T, 2010, CANCER RES, V70, P1323, DOI 10.1158/0008-5472.CAN-09-1474

Zhang HF, 2018, CANCER IMMUNOL RES, V6, P1046, DOI 10.1158/2326-6066.CIR-17-0574

Zhang W, 2010, CLIN CANCER RES, V16, P3420, DOI 10.1158/1078-0432.CCR-09-2904

Zhu Y, 2017, IMMUNITY, V47, P323, DOI 10.1016/j.immuni.2017.07.014

Zhuang XQ, 2017, NAT CELL BIOL, V19, P1274, DOI 10.1038/ncb3613

NR 78

TC 21

Z9 26

U1 1

U2 18

PU MDPI

PI BASEL

PA ST ALBAN-ANLAGE 66, CH-4052 BASEL, SWITZERLAND

SN 1422-0067

J9 INT J MOL SCI

JI Int. J. Mol. Sci.

PD JAN 1

PY 2019

VL 20

IS 1

AR 116

DI 10.3390/ijms20010116

PG 15

WC Biochemistry & Molecular Biology; Chemistry, Multidisciplinary

WE Science Citation Index Expanded (SCI-EXPANDED)

SC Biochemistry & Molecular Biology; Chemistry

GA HM8RD

UT WOS:000459747700116

PM 30597969

OA gold, Green Published, Green Submitted

DA 2025-02-07

ER

PT J

AU Davies, LC

Taylor, PR

AF Davies, Luke C.

Taylor, Philip R.

TI Tissue-resident macrophages: then and now

SO IMMUNOLOGY

LA English

DT Review

DE environmental programming; Gata6; tissue-resident macrophages

ID TUMOR-ASSOCIATED MACROPHAGES; DENDRITIC CELLS; FUNCTIONAL

SPECIALIZATION; PERITONEAL-MACROPHAGES; CARDIAC MACROPHAGES; LANGERHANS

CELLS; SPI-C; POLARIZATION; MONOCYTES; DIFFERENTIATION

AB Macrophages have been at the heart of immune research for over a century and are an integral component of innate immunity. Macrophages are often viewed as terminally differentiated monocytic phagocytes. They infiltrate tissues during inflammation, and form polarized populations that perform pro-inflammatory or anti-inflammatory functions. Tissue-resident macrophages were regarded as differentiated monocytes, which seed the tissues to perform immune sentinel and homeostatic functions. However, tissue-resident macrophages are not a homogeneous population, but are in fact a grouping of cells with similar functions and phenotypes. In the last decade, it has been revealed that many of these cells are not terminally differentiated and, in most cases, are not derived from haematopoiesis in the adult. Recent research has highlighted that tissue-resident macrophages cannot be grouped into simple polarized categories, especially in vivo, when they are exposed to complex signalling events. It has now been demonstrated that the tissue environment itself is a major controller of macrophage phenotype, and can influence the expression of many genes regardless of origin. This is consistent with the concept that cells within different tissues have diverse responses in inflammation. There is still a mountain to climb in the field, as it evolves to encompass not only tissue-resident macrophage diversity, but also categorization of specific tissue environments and the plasticity of macrophages themselves. This knowledge provides a new perspective on therapeutic strategies, as macrophage subsets can potentially be manipulated to control the inflammatory environment in a tissue-specific manner.

C1 [Davies, Luke C.] NCI, Canc Inflammat Program, NIH, Frederick, MD 21702 USA.

[Taylor, Philip R.] Cardiff Univ, Sch Med, Inst Infect & Immun, Cardiff CF10 3AX, S Glam, Wales.

C3 National Institutes of Health (NIH) - USA; NIH National Cancer Institute

(NCI); Cardiff University

RP Davies, LC (corresponding author), NCI, Ctr Canc Res, Bldg 560,Room 31-45, Frederick, MD 21702 USA.

EM luke.davies@nih.gov

OI Taylor, Philip/0000-0003-0163-1421; Davies, Luke/0000-0001-7767-4060

FU Medical Research Council [MR/J002151/1, MR/K02003X/1]; Wellcome Trust

Institutional Strategic Support Fund; federal funds from the National

Cancer Institute, National Institutes of Health, Intramural Research

Program USA; Medical Research Council UK; MRC [MR/K02003X/1,

MR/J002151/1] Funding Source: UKRI

FX We would like to acknowledge Dr Anja Bloom for her assistance in

critically reviewing the manuscript. PRT is supported by the Medical

Research Council (grants MR/J002151/1 and MR/K02003X/1), and

additionally supported through a Wellcome Trust Institutional Strategic

Support Fund. LCD is currently a postdoctoral Visiting Fellow with the

LEI, CIP, CCR, NCI. This work has been funded in part with federal funds

from the National Cancer Institute, National Institutes of Health,

Intramural Research Program USA and Medical Research Council UK. The

content of this review does not necessarily reflect the views or

policies of Cardiff University UK or the Department of Health and Human

Services USA, nor does mention of trade names, commercial products, or

organizations imply endorsement by the US Government.

CR A-Gonzalez N, 2013, NAT IMMUNOL, V14, P831, DOI 10.1038/ni.2622

Ajami B, 2007, NAT NEUROSCI, V10, P1538, DOI 10.1038/nn2014

Amano SU, 2014, CELL METAB, V19, P162, DOI 10.1016/j.cmet.2013.11.017

Aschoff L., 1924, Ergb Inn Med. Kinderheilk, V26, P1, DOI 10.1007/978-3-642-90639-8_1

Bain CC, 2014, NAT IMMUNOL, V15, P929, DOI 10.1038/ni.2967

Capo-Chichi CD, 2009, MOL CELL BIOL, V29, P4766, DOI 10.1128/MCB.00087-09

Chang JT, 2014, NAT IMMUNOL, V15, P1104, DOI 10.1038/ni.3031

Chorro L, 2009, J EXP MED, V206, P3089, DOI 10.1084/jem.20091586

Colegio OR, 2014, NATURE, V513, P559, DOI 10.1038/nature13490

CZERNIELEWSKI JM, 1987, J INVEST DERMATOL, V88, P17, DOI 10.1111/1523-1747.ep12464659

Davies LC, 2013, NAT IMMUNOL, V14, P986, DOI 10.1038/ni.2705

Davies LC, 2013, NAT COMMUN, V4, DOI 10.1038/ncomms2877

Davies LC, 2011, EUR J IMMUNOL, V41, P2155, DOI 10.1002/eji.201141817

Dominical VM, EXP HEMATOL IN PRESS

Duffy SS, 2014, MULT SCLER INT, V2014, DOI 10.1155/2014/285245

Epelman S, 2014, IMMUNITY, V41, P21, DOI 10.1016/j.immuni.2014.06.013

Epelman S, 2014, IMMUNITY, V40, P91, DOI 10.1016/j.immuni.2013.11.019

Fagarasan S, 2001, NATURE, V413, P639, DOI 10.1038/35098100

Franklin RA, 2014, SCIENCE, V344, P921, DOI 10.1126/science.1252510

Ganeshan K, 2014, ANNU REV IMMUNOL, V32, P609, DOI 10.1146/annurev-immunol-032713-120236

Gautier EL, 2014, J EXP MED, V211, P1525, DOI 10.1084/jem.20140570

Gautier EL, 2013, BLOOD, V122, P2714, DOI 10.1182/blood-2013-01-478206

Gautier EL, 2012, NAT IMMUNOL, V13, P1118, DOI 10.1038/ni.2419

Gentek R, 2014, IMMUNOL REV, V262, P56, DOI 10.1111/imr.12224

Ginhoux F, 2006, NAT IMMUNOL, V7, P265, DOI 10.1038/ni1307

Gordon S, 2005, NAT REV IMMUNOL, V5, P953, DOI 10.1038/nri1733

Gordon S, 2014, IMMUNOL REV, V262, P36, DOI 10.1111/imr.12223

Gordon SM, 2008, EUR J IMMUNOL, V38, P3257, DOI 10.1002/eji.200838855

Gosselin D, 2014, CELL, V159, P1327, DOI 10.1016/j.cell.2014.11.023

Gosselin D, 2014, IMMUNOL REV, V262, P96, DOI 10.1111/imr.12213

Guilliams M, 2014, NAT REV IMMUNOL, V14, P571, DOI 10.1038/nri3712

Gundra UM, 2014, BLOOD, V123, pE110, DOI 10.1182/blood-2013-08-520619

Haldar M, 2014, CELL, V156, P1223, DOI 10.1016/j.cell.2014.01.069

Hashimoto D, 2013, IMMUNITY, V38, P792, DOI 10.1016/j.immuni.2013.04.004

Heintzman ND, 2007, NAT GENET, V39, P311, DOI 10.1038/ng1966

Hume DA, 2014, IMMUNOL REV, V262, P74, DOI 10.1111/imr.12211

Jenkins SJ, 2011, SCIENCE, V332, P1284, DOI 10.1126/science.1204351

Kanitakis J, 2004, NEW ENGL J MED, V351, P2661, DOI 10.1056/NEJM200412163512523

Kim JW, 2006, CELL METAB, V3, P177, DOI 10.1016/j.cmet.2006.02.002

Kohyama M, 2009, NATURE, V457, P318, DOI 10.1038/nature07472

Lavin Y, 2014, CELL, V159, P1312, DOI 10.1016/j.cell.2014.11.018

Lewis WH, 1931, B JOHNS HOPKINS HOSP, V49, P17

Lichanska AM, 2000, EXP HEMATOL, V28, P601, DOI 10.1016/S0301-472X(00)00157-0

Malissen B, 2014, NAT REV IMMUNOL, V14, P417, DOI 10.1038/nri3683

Mantovani A, 2005, IMMUNITY, V23, P344, DOI 10.1016/j.immuni.2005.10.001

Martinez F.O., 2014, F1000PRIME REP, V6, P12703, DOI [DOI 10.12703/P6-13, 10.12703/P6-13]

MELNICOFF MJ, 1988, J LEUKOCYTE BIOL, V44, P367, DOI 10.1002/jlb.44.5.367

Metchnikoff E., 1892, Lecons sur la pathologie comparee de l'inflammation, Faites a l'Institut Pasteur en avril et mai 1891

Mills CD, 2000, J IMMUNOL, V164, P6166, DOI 10.4049/jimmunol.164.12.6166

Molawi K, 2014, J EXP MED, V211, P2151, DOI 10.1084/jem.20140639

Murray PJ, 2014, IMMUNITY, V41, P14, DOI 10.1016/j.immuni.2014.06.008

Namboodiri AMA, 2006, MOL CELL ENDOCRINOL, V252, P216, DOI 10.1016/j.mce.2006.03.016

NATHAN CF, 1983, J EXP MED, V158, P670, DOI 10.1084/jem.158.3.670

Newson J, 2014, BLOOD, V124, P1748, DOI 10.1182/blood-2014-03-562710

Okabe Y, 2014, CELL, V157, P832, DOI 10.1016/j.cell.2014.04.016

Ovchinnikov DA, 2008, GENESIS, V46, P447, DOI 10.1002/dvg.20417

Palazon A, 2014, IMMUNITY, V41, P518, DOI 10.1016/j.immuni.2014.09.008

PARWARESCH MR, 1984, CELL TISSUE KINET, V17, P25, DOI 10.1111/j.1365-2184.1984.tb00565.x

Quatromoni JG, 2012, AM J TRANSL RES, V4, P376

Robbins CS, 2013, NAT MED, V19, P1166, DOI 10.1038/nm.3258

Rosas M, 2014, SCIENCE, V344, P645, DOI 10.1126/science.1251414

Sabin F. R., 1925, Contributions to Embryology, V16, P125

SAWYER RT, 1982, LAB INVEST, V46, P165

Schulz C, 2012, SCIENCE, V336, P86, DOI 10.1126/science.1219179

Stables MJ, 2011, BLOOD, V118, pE192, DOI 10.1182/blood-2011-04-345330

STEIN M, 1992, J EXP MED, V176, P287, DOI 10.1084/jem.176.1.287

Suzuki T, 2014, NATURE, V514, P450, DOI 10.1038/nature13807

Tamoutounour S, 2013, IMMUNITY, V39, P925, DOI 10.1016/j.immuni.2013.10.004

VANFURTH R, 1972, B WORLD HEALTH ORGAN, V46, P845

Xue J, 2014, IMMUNITY, V40, P274, DOI 10.1016/j.immuni.2014.01.006

Yona S, 2013, IMMUNITY, V38, P79, DOI 10.1016/j.immuni.2012.12.001

Zigmond E, 2013, TRENDS IMMUNOL, V34, P162, DOI 10.1016/j.it.2013.02.001

NR 72

TC 248

Z9 312

U1 2

U2 58

PU WILEY

PI HOBOKEN

PA 111 RIVER ST, HOBOKEN 07030-5774, NJ USA

SN 0019-2805

EI 1365-2567

J9 IMMUNOLOGY

JI Immunology

PD APR

PY 2015

VL 144

IS 4

BP 541

EP 548

DI 10.1111/imm.12451

PG 8

WC Immunology

WE Science Citation Index Expanded (SCI-EXPANDED)

SC Immunology

GA CD9DR

UT WOS:000351398200001

PM 25684236

OA Green Published, hybrid

DA 2025-02-07

ER

PT J

AU Chi, JH

Gao, QL

Liu, D

AF Chi, Jianhua

Gao, Qinglei

Liu, Dan

TI Tissue-Resident Macrophages in Cancer: Friend or Foe?

SO CANCER MEDICINE

LA English

DT Review

DE cancer immunology; tissue-resident macrophages; tumor microenvironment;

tumor-associated macrophages

ID TUMOR-ASSOCIATED MACROPHAGES; ERYTHRO-MYELOID PROGENITOR; KUPFFER CELLS;

MICROGLIA; IDENTIFICATION; RECEPTOR; REVEALS; PROTEIN; HEALTH; NICHE

AB IntroductionMacrophages are essential in maintaining homeostasis, combating infections, and influencing the process of various diseases, including cancer. Macrophages originate from diverse lineages: Notably, tissue-resident macrophages (TRMs) differ from hematopoietic stem cells and circulating monocyte-derived macrophages based on genetics, development, and function. Therefore, understanding the recruited and TRM populations is crucial for investigating disease processes.MethodsBy searching literature databses, we summarized recent relevant studies. Research has shown that tumor-associated macrophages (TAMs) of distinct origins accumulate in tumor microenvironment (TME), with TRM-derived TAMs closely resembling gene signatures of normal TRMs.ResultsRecent studies have revealed that TRMs play a crucial role in cancer progression. However, organ-specific effects complicate TRM investigations. Nonetheless, the precise involvement of TRMs in tumors is unclear. This review explores the multifaceted roles of TRMs in cancer, presenting insights into their origins, proliferation, the latest research methodologies, their impact across various tumor sites, their potential and strategies as therapeutic targets, interactions with other cells within the TME, and the internal heterogeneity of TRMs.ConclusionsWe believe that a comprehensive understanding of the multifaceted roles of TRMs will pave the way for targeted TRM therapies in the treatment of cancer.

C1 [Chi, Jianhua; Gao, Qinglei; Liu, Dan] Huazhong Univ Sci & Technol, Tongji Hosp, Natl Clin Res Ctr Obstet & Gynecol, Dept Obstet & Gynecol,Tongji Med Coll, Wuhan, Peoples R China.

[Chi, Jianhua; Gao, Qinglei; Liu, Dan] Huazhong Univ Sci & Technol, Tongji Hosp, Tongji Med Coll, Minist Educ,Hubei Key Lab Tumor Invas & Metastasis, Wuhan, Peoples R China.

C3 Huazhong University of Science & Technology; Huazhong University of

Science & Technology

RP Gao, QL; Liu, D (corresponding author), Huazhong Univ Sci & Technol, Tongji Hosp, Natl Clin Res Ctr Obstet & Gynecol, Dept Obstet & Gynecol,Tongji Med Coll, Wuhan, Peoples R China.; Gao, QL; Liu, D (corresponding author), Huazhong Univ Sci & Technol, Tongji Hosp, Tongji Med Coll, Minist Educ,Hubei Key Lab Tumor Invas & Metastasis, Wuhan, Peoples R China.

EM qingleigao@hotmail.com; tj_liudan@tjh.tjmu.edu.cn

OI Chi, Jianhua/0000-0002-3384-4918

FU Hubei Natural Science Foundation Outstanding Young Talents Project

FX Figures were created with . Home for Researchers editorial team ()

provided language editing service.

CR Adhikary T, 2017, BMC GENOMICS, V18, DOI 10.1186/s12864-017-3630-9

Ahlback Anna, 2024, Methods Mol Biol, V2713, P11, DOI 10.1007/978-1-0716-3437-0_2

Ahn JH, 2002, BLOOD, V100, P1742, DOI 10.1182/blood.V100.5.1742.h81702001742_1742_1754

Akhmetzyanova I, 2021, BLOOD ADV, V5, P3592, DOI 10.1182/bloodadvances.2021005327

Aktories P, 2022, CELL REP METHODS, V2, DOI 10.1016/j.crmeth.2022.100260

Alisjahbana A, 2020, BIOCHEM PHARMACOL, V174, DOI 10.1016/j.bcp.2019.113672

Anderson NR, 2021, CANCER RES, V81, P1201, DOI 10.1158/0008-5472.CAN-20-2990

[Anonymous], 2023, CANCER DISCOV, V13, P2119, DOI 10.1158/2159-8290.CD-RW2023-130

Antunes ARP, 2021, NAT NEUROSCI, V24, P595, DOI 10.1038/s41593-020-00789-y

Asano K, 2011, IMMUNITY, V34, P85, DOI 10.1016/j.immuni.2010.12.011

Asano T, 2018, CANCER SCI, V109, P1723, DOI 10.1111/cas.13565

Badie B, 2000, NEUROSURGERY, V46, P957, DOI 10.1097/00006123-200004000-00035

Baer JM, 2023, NAT IMMUNOL, V24, P1443, DOI 10.1038/s41590-023-01579-x

Bain CC, 2016, NAT COMMUN, V7, DOI 10.1038/ncomms11852

Bao YZ, 2024, TRENDS IMMUNOL, V45, P237, DOI 10.1016/j.it.2024.02.007

Bayon LG, 1996, HEPATOLOGY, V23, P1224, DOI 10.1002/hep.510230542

Beatty GL, 2015, GASTROENTEROLOGY, V149, P201, DOI 10.1053/j.gastro.2015.04.010

Bleriot C, 2020, IMMUNITY, V52, P957, DOI 10.1016/j.immuni.2020.05.014

Bourdely P, 2022, FRONT IMMUNOL, V13, DOI 10.3389/fimmu.2022.903069

Bowman RL, 2016, CELL REP, V17, P2445, DOI 10.1016/j.celrep.2016.10.052

Brady NJ, 2016, MEDIAT INFLAMM, V2016, DOI 10.1155/2016/4549676

Brandenburg S, 2021, INT J MOL SCI, V22, DOI 10.3390/ijms22010194

Broquet A, 2024, NAT IMMUNOL, DOI 10.1038/s41590-024-01819-8

Bugatti M, 2022, CANCER IMMUNOL RES, V10, P1340, DOI 10.1158/2326-6066.CIR-22-0271

Cao MM, 2024, EXP HEMATOL ONCOL, V13, DOI 10.1186/s40164-023-00469-0

Casanova-Acebes M, 2021, CANCER DISCOV, V11, P1873, DOI 10.1158/2159-8290.CD-RW2021-089

Casanova-Acebes M, 2021, NATURE, V595, P578, DOI 10.1038/s41586-021-03651-8

Chakarov S, 2019, SCIENCE, V363, P1190, DOI 10.1126/science.aau0964

Chan CYK, 2019, ADV EXP MED BIOL, V1136, P97, DOI 10.1007/978-3-030-12734-3_7

Che G, 2024, DRUG RESIST UPDATE, V74, DOI 10.1016/j.drup.2024.101080

Chen SZ, 2023, SIGNAL TRANSDUCT TAR, V8, DOI 10.1038/s41392-023-01452-1

Chen ZH, 2017, CANCER RES, V77, P2266, DOI 10.1158/0008-5472.CAN-16-2310

Cheng M, 2017, SCI REP-UK, V7, DOI 10.1038/s41598-017-08264-8

Chow A, 2021, CANCER CELL, V39, P973, DOI 10.1016/j.ccell.2021.05.006

Christofides A, 2022, NAT IMMUNOL, V23, P1148, DOI 10.1038/s41590-022-01267-2

Coffelt SB, 2009, BBA-REV CANCER, V1796, P11, DOI 10.1016/j.bbcan.2009.02.004

Conte Enrico, 2022, Pharmacol Ther, V234, P108031, DOI 10.1016/j.pharmthera.2021.108031

Cotechini T, 2021, CELLS-BASEL, V10, DOI 10.3390/cells10040960

Cresswell GM, 2021, CANCER RES, V81, P671, DOI 10.1158/0008-5472.CAN-20-1414

Cutter S, 2023, BIOCHEM SOC T, V51, P387, DOI 10.1042/BST20221008

Davies LC, 2015, IMMUNOLOGY, V144, P541, DOI 10.1111/imm.12451

Davies LC, 2013, NAT IMMUNOL, V14, P986, DOI 10.1038/ni.2705

Davies LC, 2013, NAT COMMUN, V4, DOI 10.1038/ncomms2877

Dawson CA, 2021, J MAMMARY GLAND BIOL, V26, P71, DOI 10.1007/s10911-021-09483-6

Dawson CA, 2020, NAT CELL BIOL, V22, P546, DOI 10.1038/s41556-020-0505-0

Del Prete A, 2022, FRONT CELL DEV BIOL, V10, DOI 10.3389/fcell.2022.1068720

Dick SA, 2022, SCI IMMUNOL, V7, DOI 10.1126/sciimmunol.abf7777

Dong WW, 2023, FRONT IMMUNOL, V14, DOI 10.3389/fimmu.2023.1263633

Duffield JS, 2005, J CLIN INVEST, V115, P56, DOI 10.1172/JCI200522675

Dumas AA, 2020, EMBO J, V39, DOI 10.15252/embj.2019103790

Duzgun Z, 2016, GENE, V575, P187, DOI 10.1016/j.gene.2015.08.060

Elfstrum AK, 2024, CANCER MED-US, V13, DOI 10.1002/cam4.7053

Engblom C, 2016, NAT REV CANCER, V16, P447, DOI 10.1038/nrc.2016.54

Etzerodt A, 2020, J EXP MED, V217, DOI 10.1084/jem.20191869

Filiberti S, 2022, BIOMEDICINES, V10, DOI 10.3390/biomedicines10112709

Ginhoux F, 2010, SCIENCE, V330, P841, DOI 10.1126/science.1194637

Grabowska J, 2018, FRONT IMMUNOL, V9, DOI 10.3389/fimmu.2018.02472

Guilliams M, 2021, NAT IMMUNOL, V22, P118, DOI 10.1038/s41590-020-00849-2

Guilliams M, 2020, IMMUNITY, V52, P434, DOI 10.1016/j.immuni.2020.02.015

Guilliams M, 2017, NAT REV IMMUNOL, V17, P451, DOI 10.1038/nri.2017.42

Guillot A, 2020, METHODS MOL BIOL, V2164, P45, DOI 10.1007/978-1-0716-0704-6_6

Guldner IH, 2020, CELL, V183, P1234, DOI 10.1016/j.cell.2020.09.064

Gunnarsdottir FB, 2023, FRONT IMMUNOL, V14, DOI 10.3389/fimmu.2023.1180209

Gutbier S, 2020, INT J MOL SCI, V21, DOI 10.3390/ijms21134808

Hashimoto D, 2013, IMMUNITY, V38, P792, DOI 10.1016/j.immuni.2013.04.004

Helmy KY, 2006, CELL, V124, P915, DOI 10.1016/j.cell.2005.12.039

Hey J, 2023, INT J CANCER, V152, P1226, DOI 10.1002/ijc.34364

Hiemstra IH, 2014, IMMUNOLOGY, V142, P269, DOI 10.1111/imm.12251

Hirano R, 2023, COMMUN BIOL, V6, DOI 10.1038/s42003-023-04525-7

Hoeffel G, 2015, IMMUNITY, V42, P665, DOI 10.1016/j.immuni.2015.03.011

Hossain M, 2022, NAT COMMUN, V13, DOI 10.1038/s41467-022-32080-y

Hume DA, 2023, BLOOD, V142, P1339, DOI 10.1182/blood.2023020597

Irvine KM, 2016, JCI INSIGHT, V1, DOI 10.1172/jci.insight.86914

Jäppinen N, 2019, NAT COMMUN, V10, DOI 10.1038/s41467-018-08065-1

Jing WQ, 2018, CELL IMMUNOL, V331, P168, DOI 10.1016/j.cellimm.2017.09.006

Joshi S, 2024, CELL REP, V43, DOI 10.1016/j.celrep.2024.114096

Katholnig K, 2019, JCI INSIGHT, V4, DOI 10.1172/jci.insight.124164

Katzenelenbogen Y, 2020, CELL, V182, P872, DOI 10.1016/j.cell.2020.06.032

Kielbassa K, 2019, FRONT IMMUNOL, V10, DOI 10.3389/fimmu.2019.02215

Kim JK, 2005, IMMUNOL LETT, V99, P153, DOI 10.1016/j.imlet.2005.02.012

Kim SW, 2021, GASTRIC CANCER, V24, P327, DOI 10.1007/s10120-020-01120-1

Kimura Y, 2016, P NATL ACAD SCI USA, V113, P14097, DOI 10.1073/pnas.1617903113

Kleefeldt F, 2022, CELL DEATH DIS, V13, DOI 10.1038/s41419-022-04605-2

Kloepper J, 2016, P NATL ACAD SCI USA, V113, P4476, DOI 10.1073/pnas.1525360113

Kohlhepp MS, 2023, FRONT MOL BIOSCI, V10, DOI 10.3389/fmolb.2023.1129831

Kong Wan Ting, 2024, Methods Mol Biol, V2713, P269, DOI 10.1007/978-1-0716-3437-0_18

Kramer ED, 2023, JCI INSIGHT, V8, DOI 10.1172/jci.insight.160978

Krishnan V, 2020, COMMUN BIOL, V3, DOI 10.1038/s42003-020-01246-z

Lam JH, 2019, FRONT IMMUNOL, V10, DOI 10.3389/fimmu.2019.02093

Larionova I, 2020, FRONT ONCOL, V10, DOI 10.3389/fonc.2020.566511

Lavin Y, 2014, CELL, V159, P1312, DOI 10.1016/j.cell.2014.11.018

Lazarov T, 2023, NATURE, V618, P698, DOI 10.1038/s41586-023-06002-x

Li J, 2023, NAT IMMUNOL, V24, P1813, DOI 10.1038/s41590-023-01634-7

Li XY, 2016, INT J MOL MED, V37, P1465, DOI 10.3892/ijmm.2016.2561

Li XC, 2022, THERANOSTICS, V12, P620, DOI 10.7150/thno.60540

Liao YM, 2014, LAB INVEST, V94, P706, DOI 10.1038/labinvest.2014.73

Liao ZY, 2023, INT J CANCER, V152, P2396, DOI 10.1002/ijc.34468

Link VM, 2018, CELL, V173, P1796, DOI 10.1016/j.cell.2018.04.018

Lisi L, 2014, J NEUROINFLAMM, V11, DOI 10.1186/1742-2094-11-125

Liu B, 2023, CANCER LETT, V553, DOI 10.1016/j.canlet.2022.215996

Liu W, 2023, J CLIN INVEST, V133, DOI 10.1172/JCI157937

Liu XY, 2022, FRONT IMMUNOL, V13, DOI 10.3389/fimmu.2022.961350

Liu ZY, 2019, CELL, V178, P1509, DOI 10.1016/j.cell.2019.08.009

Loyher PL, 2018, J EXP MED, V215, P2536, DOI 10.1084/jem.20180534

Ma RY, 2022, TRENDS IMMUNOL, V43, P546, DOI 10.1016/j.it.2022.04.008

MacParland SA, 2018, NAT COMMUN, V9, DOI 10.1038/s41467-018-06318-7

Matusiak M, 2024, CANCER DISCOV, V14, P1418, DOI 10.1158/2159-8290.CD-23-1300

Meng YR, 2010, CANCER RES, V70, P1534, DOI 10.1158/0008-5472.CAN-09-2995

Mills CD, 2000, J IMMUNOL, V164, P6166, DOI 10.4049/jimmunol.164.12.6166

Mills CD, 2012, CRIT REV IMMUNOL, V32, P463, DOI 10.1615/CritRevImmunol.v32.i6.10

Miyamoto T, 2023, FRONT IMMUNOL, V14, DOI 10.3389/fimmu.2023.1104694

Miyazaki K, 2012, J IMMUNOL, V188, P4690, DOI 10.4049/jimmunol.1100605

Molgora M, 2020, CELL, V182, P886, DOI 10.1016/j.cell.2020.07.013

Monkman J, 2024, J TRANSL MED, V22, DOI 10.1186/s12967-024-05035-8

Morganti JM, 2014, PLOS ONE, V9, DOI 10.1371/journal.pone.0093650

Müller A, 2015, INT J CANCER, V137, P278, DOI 10.1002/ijc.29379

Mulder K, 2021, IMMUNITY, V54, P1883, DOI 10.1016/j.immuni.2021.07.007

Nobs SP, 2021, TRENDS IMMUNOL, V42, P495, DOI 10.1016/j.it.2021.04.007

Oh MH, 2017, CELL REP, V20, P2439, DOI 10.1016/j.celrep.2017.08.046

Ohnishi K, 2013, CANCER SCI, V104, P1236, DOI 10.1111/cas.12212

Okabe Y, 2016, NAT IMMUNOL, V17, P9, DOI 10.1038/ni.3320

Park MD, 2022, CELL, V185, P4259, DOI 10.1016/j.cell.2022.10.007

Patterson MT, 2023, CELL REP, V42, DOI 10.1016/j.celrep.2023.112732

Perdiguero EG, 2015, IMMUNITY, V43, P1023, DOI 10.1016/j.immuni.2015.11.022

Perdiguero EG, 2015, NATURE, V518, P547, DOI 10.1038/nature13989

Petit V, 2018, METHODS MOL BIOL, V1784, P93, DOI 10.1007/978-1-4939-7837-3_9

Prieto LI, 2023, CANCER CELL, V41, P1261, DOI 10.1016/j.ccell.2023.05.006

Puig-Kröger A, 2009, CANCER RES, V69, P9395, DOI 10.1158/0008-5472.CAN-09-2050

Pyonteck SM, 2013, NAT MED, V19, P1264, DOI 10.1038/nm.3337

Qian JB, 2020, CELL RES, V30, P745, DOI 10.1038/s41422-020-0355-0

Qie JB, 2022, NAT COMMUN, V13, DOI 10.1038/s41467-022-35095-7

Ramos RN, 2022, CELL, V185, P1189, DOI 10.1016/j.cell.2022.02.021

Revel M, 2022, TRENDS CANCER, V8, P517, DOI 10.1016/j.trecan.2022.02.006

Rietkötter E, 2013, ONCOTARGET, V4, P1449, DOI 10.18632/oncotarget.1201

Robinson MW, 2016, CELL MOL IMMUNOL, V13, P267, DOI 10.1038/cmi.2016.3

Rszer T., 2018, Cells, V7

Ruan CS, 2020, BRAIN BEHAV IMMUN, V83, P180, DOI 10.1016/j.bbi.2019.10.009

Sakai M, 2019, IMMUNITY, V51, P655, DOI 10.1016/j.immuni.2019.09.002

Saylor J, 2018, FRONT IMMUNOL, V9, DOI 10.3389/fimmu.2018.02925

Schultze JL, 2016, CURR OPIN PHARMACOL, V26, P10, DOI 10.1016/j.coph.2015.09.007

Schulz C, 2012, SCIENCE, V336, P86, DOI 10.1126/science.1219179

Schulz M, 2021, FRONT IMMUNOL, V12, DOI 10.3389/fimmu.2021.716504

Sevenich L, 2018, FRONT IMMUNOL, V9, DOI 10.3389/fimmu.2018.00697

Shang C, 2022, CANCER LETT, V537, DOI 10.1016/j.canlet.2022.215667

Sharma A, 2022, NAT REV CANCER, V22, P593, DOI 10.1038/s41568-022-00497-8

Sharma A, 2020, CELL, V183, P377, DOI 10.1016/j.cell.2020.08.040

Sharma SK, 2015, J IMMUNOL, V194, P5529, DOI 10.4049/jimmunol.1403215

Sheng JP, 2022, GUT, V71, P1176, DOI 10.1136/gutjnl-2021-324339

Sheng JP, 2015, IMMUNITY, V43, P382, DOI 10.1016/j.immuni.2015.07.016

Soncin I, 2018, NAT COMMUN, V9, DOI 10.1038/s41467-018-02834-8

Staiano RI, 2016, J LEUKOCYTE BIOL, V99, P531, DOI 10.1189/jlb.3HI1214-584R

Sugimura R, 2022, FRONT CELL DEV BIOL, V10, DOI 10.3389/fcell.2022.803563

Sung CYW, 2024, SCI ADV, V10, DOI 10.1126/sciadv.adk9878

Symchych T. V, 2020, Experimental Oncology, V42, P197, DOI 10.32471/exp-oncology.2312-8852.vol-42-no-3.14928

Tacke F, 2017, J HEPATOL, V66, P1300, DOI 10.1016/j.jhep.2017.02.026

Tao S, 2020, CLIN TRANSL ONCOL, V22, P1938, DOI 10.1007/s12094-020-02348-0

Tapmeier TT, 2022, ONCOGENE, V41, P5032, DOI 10.1038/s41388-022-02488-3

Ulland TK, 2018, NAT REV NEUROL, V14, P667, DOI 10.1038/s41582-018-0072-1

Ural BB, 2020, SCI IMMUNOL, V5, DOI 10.1126/sciimmunol.aax8756

van Elsas MJ, 2023, J IMMUNOTHER CANCER, V11, DOI 10.1136/jitc-2022-006433

Vanderborght B, 2023, INT J CANCER, V152, P2615, DOI 10.1002/ijc.34505

Vogel A, 2023, CURR OPIN BIOTECH, V83, DOI 10.1016/j.copbio.2023.102984

Vogt L, 2006, J CLIN INVEST, V116, P2817, DOI 10.1172/JCI25673

Walker MG, 2002, BBA-GENE STRUCT EXPR, V1574, P387, DOI 10.1016/S0167-4781(01)00358-X

Wang CY, 2016, J NEUROSCI RES, V94, P1460, DOI 10.1002/jnr.23922

Wang HB, 2024, DRUG RESIST UPDATE, V73, DOI 10.1016/j.drup.2023.101041

Wang JF, 2021, J HEPATOL, V74, P627, DOI 10.1016/j.jhep.2020.10.021

Wang T, 2023, NAT IMMUNOL, V24, P423, DOI 10.1038/s41590-023-01428-x

Wang Y, 2022, J HEMATOL ONCOL, V15, DOI 10.1186/s13045-022-01335-y

Weiskopf K, 2016, MICROBIOL SPECTR, V4, DOI 10.1128/microbiolspec.MCHD-0031-2016

Wen JC, 2023, EUR J MED CHEM, V245, DOI 10.1016/j.ejmech.2022.114884

Wen SW, 2013, CANCER BIOL THER, V14, P606, DOI 10.4161/cbt.24593

Wu LC, 2023, P NATL ACAD SCI USA, V120, DOI 10.1073/pnas.2210836120

Xia HJ, 2020, JCI INSIGHT, V5, DOI 10.1172/jci.insight.141115

Xiang C, 2023, CELL DEATH DIS, V14, DOI 10.1038/s41419-023-06021-6

Xu T, 2015, AM J TRANSL RES, V7, P1172

Yadav S, 2023, IMMUNOL RES, V71, P130, DOI [10.1007/s12026-022-09330-8, 10.1109/IECON49645.2022.9969022]

Yang KD, 2023, BMC CANCER, V23, DOI 10.1186/s12885-023-10675-y

Yang YL, 2022, INT IMMUNOPHARMACOL, V110, DOI 10.1016/j.intimp.2022.109047

You H, 2019, FRONT IMMUNOL, V10, DOI 10.3389/fimmu.2019.01941

Yuan XM, 2017, ELIFE, V6, DOI 10.7554/eLife.29540

Zhang JL, 2023, CELL REP, V42, DOI 10.1016/j.celrep.2023.112620

Zhang N, 2021, J EXP MED, V218, DOI 10.1084/jem.20210924

Zhang XM, 2021, CELLS-BASEL, V10, DOI 10.3390/cells10040903

Zhang ZJ, 2021, BIOL REPROD, V104, P527, DOI 10.1093/biolre/ioaa219

Zhao Y, 2023, CELL REP, V42, DOI 10.1016/j.celrep.2023.113364

Zhong L, 2017, J EXP MED, V214, P597, DOI 10.1084/jem.20160844

Zhu Y, 2017, IMMUNITY, V47, P323, DOI 10.1016/j.immuni.2017.07.014

Zilionis R, 2019, IMMUNITY, V50, P1317, DOI 10.1016/j.immuni.2019.03.009

NR 189

TC 0

Z9 0

U1 4

U2 4

PU WILEY

PI HOBOKEN

PA 111 RIVER ST, HOBOKEN 07030-5774, NJ USA

SN 2045-7634

J9 CANCER MED-US

JI Cancer Med.

PD NOV

PY 2024

VL 13

IS 21

AR e70387

DI 10.1002/cam4.70387

PG 16

WC Oncology

WE Science Citation Index Expanded (SCI-EXPANDED)

SC Oncology

GA O0K2L

UT WOS:001368111500001

PM 39494816

OA gold

DA 2025-02-07

ER

PT J

AU Cruz, AF

Rohban, R

Esni, F

AF Cruz, Andrea F.

Rohban, Rokhsareh

Esni, Farzad

TI Macrophages in the pancreas: Villains by circumstances, not necessarily

by actions

SO IMMUNITY INFLAMMATION AND DISEASE

LA English

DT Review

DE diabetes; monocytes; macrophages; pancreatic cancer; pancreatitis;

regeneration

ID NF-KAPPA-B; TUMOR-ASSOCIATED MACROPHAGES; TISSUE-RESIDENT MACROPHAGES;

MESENCHYMAL STEM-CELLS; TO-DUCTAL METAPLASIA; NERVE GROWTH-FACTOR;

ALTERNATIVE ACTIVATION; STEADY-STATE; LIVER-INJURY; IN-VIVO

AB Introduction Mounting evidence suggest that macrophages play crucial roles in disease and tissue regeneration. However, despite much efforts during the past decade, our knowledge about the extent of macrophages' contribution to adult pancreatic regeneration after injury or during pancreatic disease progression is still limited. Nevertheless, it is generally accepted that some macrophage features that normally would contribute to healing and regeneration may be detrimental in pancreatic cancer. Altogether, the current literature contains conflicting reports on whether macrophages act as friends or foe in these conditions. Methods and Results In this review, we briefly review the origins of tissue resident and infiltrating macrophages and the importance of cellular crosstalking between macrophages and other resident cells in tissue regeneration. The primary objective of this review is to summarize our knowledge of the distinct roles of tissue resident and infiltrating macrophages, the impact of M1 and M2 macrophage phenotypes, and emerging evidence on macrophage crosstalking in pancreatic injury, regeneration, and disease. Conclusion Macrophages are involved with various stages of pancreatic cancer development, pancreatitis, and diabetes. Elucidating their role in these conditions will aid the development of targeted therapeutic treatments.

C1 [Cruz, Andrea F.; Esni, Farzad] Univ Pittsburgh, Div Pediat Gen & Thorac Surg, Dept Surg, Childrens Hosp Pittsburgh,Med Ctr, Pittsburgh, PA 15224 USA.

[Rohban, Rokhsareh] Med Univ Graz, Dept Internal Med, Div Endocrinol & Diabetol, Graz, Austria.

[Esni, Farzad] Univ Pittsburgh, Dept Dev Biol, Pittsburgh, PA 15224 USA.

[Esni, Farzad] Univ Pittsburgh, Canc Inst, Pittsburgh, PA 15224 USA.

C3 Pennsylvania Commonwealth System of Higher Education (PCSHE); University

of Pittsburgh; Medical University of Graz; Pennsylvania Commonwealth

System of Higher Education (PCSHE); University of Pittsburgh;

Pennsylvania Commonwealth System of Higher Education (PCSHE); University

of Pittsburgh

RP Esni, F (corresponding author), Univ Pittsburgh, Dept Surg, John G Rangos Res Ctr, One Childrens Hosp Dr,Rangos Floor 6,Room 6123, Pittsburgh, PA 15224 USA.

EM farzad.esni@chp.edu

RI Cruz, Andrea/GWQ-5824-2022; Esni, Farzad/AAX-1136-2021

OI Esni, Farzad/0000-0002-0342-6862; Cruz, Andrea/0000-0001-9353-9942

FU NIH/NIDDK [DK101413, DK103002]; NCI [CA236965]; Children's Hospital of

Pittsburgh of UPMC; RACm

FX This study was supported by NIH/NIDDK grants DK101413 and DK103002, and

NCI grant CA236965, RACm and The Children's Hospital of Pittsburgh of

UPMC.

CR Aamodt KI, 2017, DIABETES OBES METAB, V19, P124, DOI 10.1111/dom.13031

Akashi K, 2000, NATURE, V404, P193, DOI 10.1038/35004599

Amano SU, 2014, CELL METAB, V19, P162, DOI 10.1016/j.cmet.2013.11.017

Ardito CM, 2012, CANCER CELL, V22, P304, DOI 10.1016/j.ccr.2012.07.024

Arnold L, 2007, J EXP MED, V204, P1057, DOI 10.1084/jem.20070075

Aurora AB, 2014, J CLIN INVEST, V124, P1382, DOI 10.1172/JCI72181

Baffy G, 2009, J HEPATOL, V51, P212, DOI 10.1016/j.jhep.2009.03.008

Banaei-Bouchareb L, 2006, J ENDOCRINOL, V188, P467, DOI 10.1677/joe.1.06225

Ben-Mordechai T, 2013, J AM COLL CARDIOL, V62, P1890, DOI 10.1016/j.jacc.2013.07.057

Bertrand JY, 2005, BLOOD, V106, P3004, DOI 10.1182/blood-2005-02-0461

Bird TG, 2013, P NATL ACAD SCI USA, V110, P6542, DOI 10.1073/pnas.1302168110

Bishehsari F, 2018, INT J CANCER, V143, P1994, DOI 10.1002/ijc.31592

Boulter L, 2012, NAT MED, V18, P572, DOI 10.1038/nm.2667

Brissova M, 2014, CELL METAB, V19, P498, DOI 10.1016/j.cmet.2014.02.001

Brown BN, 2012, BIOMATERIALS, V33, P3792, DOI 10.1016/j.biomaterials.2012.02.034

Bryer SC, 2008, J IMMUNOL, V180, P1179, DOI 10.4049/jimmunol.180.2.1179

Butoi E, 2016, BBA-MOL CELL RES, V1863, P1568, DOI 10.1016/j.bbamcr.2016.04.001

Calderon B, 2006, AM J PATHOL, V169, P2137, DOI 10.2353/ajpath.2006.060539

Calderon B, 2015, J EXP MED, V212, P1497, DOI 10.1084/jem.20150496

CAMPBELL IL, 1993, P NATL ACAD SCI USA, V90, P10061, DOI 10.1073/pnas.90.21.10061

Cao XC, 2014, INT J BIOCHEM CELL B, V53, P372, DOI 10.1016/j.biocel.2014.06.003

Carlin LM, 2013, CELL, V153, P362, DOI 10.1016/j.cell.2013.03.010

Chang MK, 2008, J IMMUNOL, V181, P1232, DOI 10.4049/jimmunol.181.2.1232

Chazaud B, 2014, IMMUNOBIOLOGY, V219, P172, DOI 10.1016/j.imbio.2013.09.001

Cioffi M, 2015, CLIN CANCER RES, V21, P2325, DOI 10.1158/1078-0432.CCR-14-1399

Clark CE, 2007, CANCER RES, V67, P9518, DOI 10.1158/0008-5472.CAN-07-0175

Cnop M, 2005, DIABETES, V54, pS97, DOI 10.2337/diabetes.54.suppl_2.S97

Coffelt SB, 2010, CANCER RES, V70, P5270, DOI 10.1158/0008-5472.CAN-10-0012

Collisson EA, 2012, CANCER DISCOV, V2, P685, DOI 10.1158/2159-8290.CD-11-0347

Crisan M, 2008, CELL STEM CELL, V3, P301, DOI 10.1016/j.stem.2008.07.003

Criscimanna A, 2014, GASTROENTEROLOGY, V147, P1106, DOI 10.1053/j.gastro.2014.08.008

Cui R, 2016, ONCOTARGET, V7, P50735, DOI 10.18632/oncotarget.9383

Dal-Secco D, 2015, J EXP MED, V212, P447, DOI 10.1084/jem.20141539

Darville MI, 1998, DIABETOLOGIA, V41, P1101, DOI 10.1007/s001250051036

Dayan V, 2011, BASIC RES CARDIOL, V106, P1299, DOI 10.1007/s00395-011-0221-9

Denroche HC, 2018, DIABETOLOGIA, V61, P39, DOI 10.1007/s00125-017-4443-8

Dort J, 2019, STEM CELLS INT, V2019, DOI 10.1155/2019/4761427

Duffield JS, 2005, J CLIN INVEST, V115, P56, DOI 10.1172/JCI200522675

Eizirik DL, 2001, DIABETOLOGIA, V44, P2115, DOI 10.1007/s001250100021

Eming SA, 2017, SCIENCE, V356, P1026, DOI 10.1126/science.aam7928

Epelman S, 2014, IMMUNITY, V41, P21, DOI 10.1016/j.immuni.2014.06.013

Epelman S, 2014, IMMUNITY, V40, P91, DOI 10.1016/j.immuni.2013.11.019

Fan YF, 2008, J CEREBR BLOOD F MET, V28, P90, DOI 10.1038/sj.jcbfm.9600509

Feng Y, 2018, J AM SOC NEPHROL, V29, P182, DOI 10.1681/ASN.2017040391

Fukuda A, 2011, CANCER CELL, V19, P441, DOI 10.1016/j.ccr.2011.03.002

Gautier EL, 2012, NAT IMMUNOL, V13, P1118, DOI 10.1038/ni.2419

Gea-Sorli Sabrina, 2010, World J Gastrointest Pharmacol Ther, V1, P107, DOI 10.4292/wjgpt.v1.i5.107

Geissmann F, 2003, IMMUNITY, V19, P71, DOI 10.1016/S1074-7613(03)00174-2

Geutskens SB, 2005, J LEUKOCYTE BIOL, V78, P845, DOI 10.1189/jlb.1004624

Ginhoux F, 2016, IMMUNITY, V44, P439, DOI 10.1016/j.immuni.2016.02.024

Ginhoux F, 2010, SCIENCE, V330, P841, DOI 10.1126/science.1194637

Godwin JW, 2013, P NATL ACAD SCI USA, V110, P9415, DOI 10.1073/pnas.1300290110

Gong DP, 2012, BMC IMMUNOL, V13, DOI 10.1186/1471-2172-13-31

Gordon S, 2005, NAT REV IMMUNOL, V5, P953, DOI 10.1038/nri1733

Gordon S, 2010, IMMUNITY, V32, P593, DOI 10.1016/j.immuni.2010.05.007

Guilliams M, 2013, J EXP MED, V210, P1977, DOI 10.1084/jem.20131199

Habtezion A, 2016, CANCER LETT, V381, P211, DOI 10.1016/j.canlet.2015.11.049

Hajmrle C, 2016, JCI INSIGHT, V1, DOI 10.1172/jci.insight.86055

Halbrook CJ, 2017, CELL MOL GASTROENTER, V3, P99, DOI [10.1016/j.jcmgh.2016.08.006, 10.1016/j.jcmgh.2016.09.009]

Hashimoto D, 2013, IMMUNITY, V38, P792, DOI 10.1016/j.immuni.2013.04.004

Helm O, 2014, ONCOIMMUNOLOGY, V3, DOI 10.4161/21624011.2014.946818

Hoeffel G, 2015, FRONT IMMUNOL, V6, DOI 10.3389/fimmu.2015.00486

Hoeffel G, 2012, J EXP MED, V209, P1167, DOI 10.1084/jem.20120340

Homo-Delarche F, 2004, TRENDS IMMUNOL, V25, P222, DOI 10.1016/j.it.2004.02.012

Hordijk PL, 2006, FEBS J, V273, P4408, DOI 10.1111/j.1742-4658.2006.05440.x

Houbracken I, 2011, GASTROENTEROLOGY, V141, P731, DOI 10.1053/j.gastro.2011.04.050

Hu H, 2015, TUMOR BIOL, V36, P9119, DOI 10.1007/s13277-015-4127-2

Huang HJ, 2013, GASTROENTEROLOGY, V144, P202, DOI 10.1053/j.gastro.2012.09.059

Ino Y, 2013, BRIT J CANCER, V108, P914, DOI 10.1038/bjc.2013.32

Jakubzick C, 2013, IMMUNITY, V39, P599, DOI 10.1016/j.immuni.2013.08.007

Jenkins SJ, 2011, SCIENCE, V332, P1284, DOI 10.1126/science.1204351

Jeon SH, 2007, J LEUKOCYTE BIOL, V81, P557, DOI 10.1189/jlb.0806517

Jun HS, 1999, J EXP MED, V189, P347, DOI 10.1084/jem.189.2.347

Kamisawa T, 2016, LANCET, V388, P73, DOI 10.1016/S0140-6736(16)00141-0

Karlmark KR, 2009, HEPATOLOGY, V50, P261, DOI 10.1002/hep.22950

Kendall TJ, 2009, HEPATOLOGY, V49, P901, DOI 10.1002/hep.22701

Kim J, 2010, J IMMUNOL, V185, P1274, DOI 10.4049/jimmunol.1000181

Klöppel G, 2007, MODERN PATHOL, V20, pS113, DOI 10.1038/modpathol.3800690

Kordes C, 2014, J CLIN INVEST, V124, P5503, DOI 10.1172/JCI74119

Kordes C, 2013, CELL PHYSIOL BIOCHEM, V31, P290, DOI 10.1159/000343368

Krafts KP, 2010, ORGANOGENESIS, V6, P225, DOI 10.4161/org.6.4.12555

Kühnemuth B, 2015, ONCOGENE, V34, P177, DOI 10.1038/onc.2013.530

Kurahara H, 2011, J SURG RES, V167, pE211, DOI 10.1016/j.jss.2009.05.026

Lankadasari MB, 2019, MOL CANCER, V18, DOI 10.1186/s12943-019-0966-6

Lech M, 2013, BBA-MOL BASIS DIS, V1832, P989, DOI 10.1016/j.bbadis.2012.12.001

Lesina M, 2011, CANCER CELL, V19, P456, DOI 10.1016/j.ccr.2011.03.009

Li AH, 2003, J IMMUNOL, V170, P3369, DOI 10.4049/jimmunol.170.6.3369

Li XL, 2019, MOL CANCER, V18, DOI 10.1186/s12943-019-1102-3

Lin SL, 2010, P NATL ACAD SCI USA, V107, P4194, DOI 10.1073/pnas.0912228107

Ling JH, 2012, CANCER CELL, V21, P105, DOI 10.1016/j.ccr.2011.12.006

Liou GY, 2017, CELL REP, V19, P1322, DOI 10.1016/j.celrep.2017.04.052

Liou GY, 2013, J CELL BIOL, V202, P563, DOI 10.1083/jcb.201301001

Liu F, 2019, STEM CELL RES THER, V10, DOI 10.1186/s13287-019-1447-y

Liu J, 2016, SCI REP-UK, V6, DOI 10.1038/srep30904

Loke P, 2007, J IMMUNOL, V179, P3926, DOI 10.4049/jimmunol.179.6.3926

Lucas T, 2010, J IMMUNOL, V184, P3964, DOI 10.4049/jimmunol.0903356

Ma XY, 2016, ONCOL REP, V35, P189, DOI 10.3892/or.2015.4357

MacDonald TT, 2011, GASTROENTEROLOGY, V140, P1768, DOI 10.1053/j.gastro.2011.02.047

Maniati E, 2011, J CLIN INVEST, V121, P4685, DOI 10.1172/JCI45797

Martignoni ME, 2009, ONCOL REP, V21, P363, DOI 10.3892/or_00000231

Martignoni ME, 2005, CLIN CANCER RES, V11, P5802, DOI 10.1158/1078-0432.CCR-05-0185

Martin P, 2005, TRENDS CELL BIOL, V15, P599, DOI 10.1016/j.tcb.2005.09.002

Martinez FO, 2009, ANNU REV IMMUNOL, V27, P451, DOI 10.1146/annurev.immunol.021908.132532

Masckauchan TNH, 2006, MOL BIOL CELL, V17, P5163, DOI 10.1091/mbc.E06-04-0320

MCCULLOCH DK, 1991, DIABETES, V40, P673, DOI 10.2337/diabetes.40.6.673

McMillan DC, 1998, NUTR CANCER, V31, P101, DOI 10.1080/01635589809514687

Meirelles LD, 2020, CELLS-BASEL, V9, DOI 10.3390/cells9010188

Meirelles LD, 2016, FRONT BIOSCI-LANDMRK, V21, P130, DOI 10.2741/4380

Meng C, 2015, BIOCHEM BIOPH RES CO, V466, P393, DOI 10.1016/j.bbrc.2015.09.037

Meyers N, 2020, SCI REP-UK, V10, DOI 10.1038/s41598-020-62106-8

Mielgo A, 2013, BMB REP, V46, P131, DOI 10.5483/BMBRep.2013.46.3.036

Minutti CM, 2019, IMMUNITY, V50, P645, DOI 10.1016/j.immuni.2019.01.008

Miron VE, 2013, NAT NEUROSCI, V16, P1211, DOI 10.1038/nn.3469

Mitchem JB, 2013, CANCER RES, V73, P1128, DOI 10.1158/0008-5472.CAN-12-2731

Miyamoto Y, 2003, CANCER CELL, V3, P565, DOI 10.1016/S1535-6108(03)00140-5

MOLDAWER LL, 1992, JPEN-PARENTER ENTER, V16, pS43, DOI 10.1177/014860719201600602

Morris JP, 2010, NAT REV CANCER, V10, P683, DOI 10.1038/nrc2899

Mutgan AC, 2018, MOL CANCER, V17, DOI 10.1186/s12943-018-0806-0

Nahrendorf M, 2007, J EXP MED, V204, P3037, DOI 10.1084/jem.20070885

Newman AC, 2012, VASC CELL, V4, DOI 10.1186/2045-824X-4-13

O'Riordain MG, 1999, INT J ONCOL, V15, P823

Oakley F, 2003, AM J PATHOL, V163, P1849, DOI 10.1016/S0002-9440(10)63544-4

Orkin SH, 2008, CELL, V132, P631, DOI 10.1016/j.cell.2008.01.025

Orth M, 2019, RADIAT ONCOL, V14, DOI 10.1186/s13014-019-1345-6

Padoan A, 2019, INT J MOL SCI, V20, DOI 10.3390/ijms20030676

Paik YH, 2003, HEPATOLOGY, V37, P1043, DOI 10.1053/jhep.2003.50182

Paik YH, 2006, LAB INVEST, V86, P676, DOI 10.1038/labinvest.3700422

Pajarinen J, 2019, BIOMATERIALS, V196, P80, DOI 10.1016/j.biomaterials.2017.12.025

Palis J, 2001, EXP HEMATOL, V29, P927, DOI 10.1016/S0301-472X(01)00669-5

Perdiguero EG, 2015, NATURE, V518, P547, DOI 10.1038/nature13989

Pereira C, 2008, ARTERIOSCL THROM VAS, V28, P504, DOI 10.1161/ATVBAHA.107.157438

Perugorria MJ, 2008, HEPATOLOGY, V48, P1251, DOI 10.1002/hep.22437

Poeta VM, 2019, FRONT IMMUNOL, V10, DOI 10.3389/fimmu.2019.00379

Pull SL, 2005, P NATL ACAD SCI USA, V102, P99, DOI 10.1073/pnas.0405979102

Qin CC, 2017, WORLD J GASTROENTERO, V23, P3043, DOI 10.3748/wjg.v23.i17.3043

Ramachandran P, 2012, P NATL ACAD SCI USA, V109, pE3186, DOI 10.1073/pnas.1119964109

Rosell A, 2009, J CEREBR BLOOD F MET, V29, P933, DOI 10.1038/jcbfm.2009.17

Sainz B, 2016, MEDIAT INFLAMM, V2016, DOI 10.1155/2016/9012369

Sainz B, 2015, GUT, V64, P1921, DOI 10.1136/gutjnl-2014-308935

Sainz B, 2014, CANCER RES, V74, P7309, DOI 10.1158/0008-5472.CAN-14-1354

Sawey ET, 2007, P NATL ACAD SCI USA, V104, P19327, DOI 10.1073/pnas.0705953104

Schulz C, 2012, SCIENCE, V336, P86, DOI 10.1126/science.1219179

Shi C, 2011, NAT REV IMMUNOL, V11, P762, DOI 10.1038/nri3070

Sica A, 2012, J CLIN INVEST, V122, P787, DOI 10.1172/JCI59643

Stark K, 2013, NAT IMMUNOL, V14, P41, DOI 10.1038/ni.2477

Stefanowski J, 2019, FRONT IMMUNOL, V10, DOI 10.3389/fimmu.2019.02588

Stefater JA, 2013, BLOOD, V121, P2574, DOI 10.1182/blood-2012-06-434621

Stefater JA, 2011, TRENDS MOL MED, V17, P743, DOI 10.1016/j.molmed.2011.07.009

Stefater JA, 2011, NATURE, V474, P511, DOI 10.1038/nature10085

Storz P, 2017, NAT REV GASTRO HEPAT, V14, P296, DOI 10.1038/nrgastro.2017.12

Storz P, 2015, ONCOIMMUNOLOGY, V4, DOI 10.1080/2162402X.2015.1008794

Strobel O, 2007, GASTROENTEROLOGY, V133, P1999, DOI 10.1053/j.gastro.2007.09.009

Sunderkötter C, 2004, J IMMUNOL, V172, P4410, DOI 10.4049/jimmunol.172.7.4410

Suzuki T, 2013, ACTA HISTOCHEM CYTOC, V46, P51, DOI 10.1267/ahc.12035

Tan ZM, 2013, J IMMUNOL, V191, P1835, DOI 10.4049/jimmunol.1203013

Tanabe K, 2017, J DIABETES RES, V2017, DOI 10.1155/2017/3605178

Tandon M, 2019, CANCER RES, V79, P5316, DOI 10.1158/0008-5472.CAN-18-3064

Theret M, 2019, DEVELOPMENT, V146, DOI 10.1242/dev.156000

Thomas D, 2019, MOL CANCER, V18, DOI 10.1186/s12943-018-0927-5

Trim N, 2000, AM J PATHOL, V156, P1235, DOI 10.1016/S0002-9440(10)64994-2

Valilou SF, 2018, CYTOKINE GROWTH F R, V39, P46, DOI 10.1016/j.cytogfr.2018.01.007

Van Gassen N, 2015, STEM CELL TRANSL MED, V4, P555, DOI 10.5966/sctm.2014-0272

Van Gassen N, 2015, EUR J IMMUNOL, V45, P1482, DOI 10.1002/eji.201445013

van Rooijen N, 2010, METHODS MOL BIOL, V605, P189, DOI 10.1007/978-1-60327-360-2_13

Varol C, 2007, J EXP MED, V204, P171, DOI 10.1084/jem.20061011

Vert G, 2011, DEV CELL, V21, P985, DOI 10.1016/j.devcel.2011.11.006

Vi L, 2015, J BONE MINER RES, V30, P1090, DOI 10.1002/jbmr.2422

Wang XF, 2018, CANCER RES, V78, P4586, DOI 10.1158/0008-5472.CAN-17-3841

Watchorn TM, 2005, INT J ONCOL, V27, P1105

Waters AM, 2018, CSH PERSPECT MED, V8, DOI 10.1101/cshperspect.a031435

Wicksteed B, 2010, DIABETES, V59, P3090, DOI 10.2337/db10-0624

Willcox A, 2009, CLIN EXP IMMUNOL, V155, P173, DOI 10.1111/j.1365-2249.2008.03860.x

Willenborg S, 2012, BLOOD, V120, P613, DOI 10.1182/blood-2012-01-403386

Wynn TA, 2008, J PATHOL, V214, P199, DOI 10.1002/path.2277

Wynn TA, 2016, IMMUNITY, V44, P450, DOI 10.1016/j.immuni.2016.02.015

Xiao XW, 2014, P NATL ACAD SCI USA, V111, pE1211, DOI 10.1073/pnas.1321347111

Xue JF, 2015, NAT COMMUN, V6, DOI 10.1038/ncomms7156

Yadav D, 2013, GASTROENTEROLOGY, V144, P1252, DOI 10.1053/j.gastro.2013.01.068

Ying W, 2019, CELL METAB, V29, P457, DOI 10.1016/j.cmet.2018.12.003

Yona S, 2013, IMMUNITY, V38, P79, DOI 10.1016/j.immuni.2012.12.001

Yrlid U, 2006, J IMMUNOL, V176, P4155, DOI 10.4049/jimmunol.176.7.4155

Zhan HX, 2017, CANCER LETT, V392, P83, DOI 10.1016/j.canlet.2017.01.041

Zhang F, 2016, ONCOTARGET, V7, P52294, DOI 10.18632/oncotarget.10561

Zhang YQ, 2019, ANNU REV PHYSIOL, V81, P211, DOI 10.1146/annurev-physiol-020518-114515

Zhang YQ, 2017, ELIFE, V6, DOI 10.7554/eLife.27388

Zhang YQ, 2017, GUT, V66, P124, DOI 10.1136/gutjnl-2016-312078

Zhu Y, 2017, IMMUNITY, V47, P323, DOI 10.1016/j.immuni.2017.07.014

Zigmond E, 2014, J IMMUNOL, V193, P344, DOI 10.4049/jimmunol.1400574

NR 188

TC 15

Z9 17

U1 0

U2 3

PU WILEY

PI HOBOKEN

PA 111 RIVER ST, HOBOKEN 07030-5774, NJ USA

EI 2050-4527

J9 IMMUN INFLAMM DIS

JI IMMUN. INFLAMM. DIS.

PD DEC

PY 2020

VL 8

IS 4

BP 807

EP 824

DI 10.1002/iid3.345

EA SEP 2020

PG 18

WC Immunology

WE Science Citation Index Expanded (SCI-EXPANDED)

SC Immunology

GA OP1AQ

UT WOS:000565616800001

PM 32885589

OA gold, Green Published

DA 2025-02-07

ER

PT J

AU Biswas, M

AF Biswas, Manjusha

TI Understanding tissue-resident macrophages unlocks the potential for

novel combinatorial strategies in breast cancer

SO FRONTIERS IN IMMUNOLOGY

LA English

DT Review

DE breast cancer; tissue resident macrophage; macrophage ontogeny; tumor

microenvironment; immunotherapy

ID TUMOR-ASSOCIATED MACROPHAGES; SINGLE-CELL; MONOCYTES; SUPPRESSION;

ACTIVATION; RESISTANCE; ORIGINATE; LANDSCAPE; SIGNATURE; CARCINOMA

AB Tissue-resident macrophages (TRMs) are an integral part of the innate immune system, but their biology is not well understood in the context of cancer. Distinctive resident macrophage populations are identified in different organs in mice using fate mapping studies. They develop from the yolk sac and self-maintain themselves lifelong in specific tissular niches. Similarly, breast-resident macrophages are part of the mammary gland microenvironment. They reside in the breast adipose tissue stroma and close to the ductal epithelium and help in morphogenesis. In breast cancer, TRMs may promote disease progression and metastasis; however, precise mechanisms have not been elucidated. TRMs interact intimately with recruited macrophages, cytotoxic T cells, and other immune cells along with cancer cells, deciding further immunosuppressive or cytotoxic pathways. Moreover, triple-negative breast cancer (TNBC), which is generally associated with poor outcomes, can harbor specific TRM phenotypes. The influence of TRMs on adipose tissue stroma of the mammary gland also contributes to tumor progression. The complex crosstalk between TRMs with T cells, stroma, and breast cancer cells can establish a cascade of downstream events, understanding which can offer new insight for drug discovery and upcoming treatment choices. This review aims to acknowledge the previous research done in this regard while exploring existing research gaps and the future therapeutic potential of TRMs as a combination or single agent in breast cancer.

C1 [Biswas, Manjusha] Univ Bonn, Life & Med Sci LIMES Inst, Dept Mol Biomed, Dev Biol Immune Syst, Bonn, Germany.

[Biswas, Manjusha] Univ Bonn, Univ Hosp, Inst Pharmacol & Toxicol, Bonn, Germany.

C3 University of Bonn; University of Bonn

RP Biswas, M (corresponding author), Univ Bonn, Life & Med Sci LIMES Inst, Dept Mol Biomed, Dev Biol Immune Syst, Bonn, Germany.; Biswas, M (corresponding author), Univ Bonn, Univ Hosp, Inst Pharmacol & Toxicol, Bonn, Germany.

EM manjusha@uni-bonn.de

FU University & State Library of Bonn, University of Bonn, Germany

FX The author(s) declare financial support was received for the research,

authorship, and/or publication of this article. The author(s) declare

financial support was received for the publication (APC) for this

article. The open access fee is supported by the University & State

Library of Bonn, University of Bonn, Germany.

CR Ali HR, 2016, PLOS MED, V13, DOI 10.1371/journal.pmed.1002194

AlSendi M, 2021, INT J CANCER, V149, P1520, DOI 10.1002/ijc.33693

Alshetaiwi H, 2020, SCI IMMUNOL, V5, DOI 10.1126/sciimmunol.aay6017

Arnold M, 2022, BREAST, V66, P15, DOI 10.1016/j.breast.2022.08.010

Azizi E, 2018, CELL, V174, P1293, DOI 10.1016/j.cell.2018.05.060

Barkal AA, 2019, NATURE, V572, P392, DOI 10.1038/s41586-019-1456-0

Beck AH, 2009, CLIN CANCER RES, V15, P778, DOI 10.1158/1078-0432.CCR-08-1283

Gunnarsdottir FB, 2020, ONCOIMMUNOLOGY, V9, DOI 10.1080/2162402X.2020.1848067

Blumenthal RL, 2001, J ALLERGY CLIN IMMUN, V107, P258, DOI 10.1067/mai.2001.112845

Brady NJ, 2016, MEDIAT INFLAMM, V2016, DOI 10.1155/2016/4549676

Cassetta L, 2019, CANCER CELL, V35, P588, DOI 10.1016/j.ccell.2019.02.009

Ch'ng ES, 2013, VIRCHOWS ARCH, V462, P257, DOI 10.1007/s00428-012-1362-4

Chen YZ, 2021, BIOMED PHARMACOTHER, V139, DOI 10.1016/j.biopha.2021.111605

Cheng SJ, 2021, CELL, V184, P792, DOI 10.1016/j.cell.2021.01.010

Coffelt SB, 2009, BBA-REV CANCER, V1796, P11, DOI 10.1016/j.bbcan.2009.02.004

Colegio OR, 2014, NATURE, V513, P559, DOI 10.1038/nature13490

Cotechini T, 2021, CELLS-BASEL, V10, DOI 10.3390/cells10040960

Dawson CA, 2020, NAT CELL BIOL, V22, P546, DOI 10.1038/s41556-020-0505-0

Debien V, 2023, NPJ BREAST CANCER, V9, DOI 10.1038/s41523-023-00508-3

Dees S, 2021, TRENDS CANCER, V7, P162, DOI 10.1016/j.trecan.2020.09.004

Dick SA, 2022, SCI IMMUNOL, V7, DOI 10.1126/sciimmunol.abf7777

Eng LG, 2016, BREAST CANCER RES TR, V160, P145, DOI 10.1007/s10549-016-3974-x

Engblom C, 2016, NAT REV CANCER, V16, P447, DOI 10.1038/nrc.2016.54

Engin AB, 2019, J MOL ENDOCRINOL, V62, pR201, DOI 10.1530/JME-18-0252

Evans KT, 2023, NAT CELL BIOL, V25, DOI 10.1038/s41556-023-01273-y

Fan HJ, 2024, CANCER RES, V84, P449, DOI 10.1158/0008-5472.CAN-23-1443

Franklin RA, 2014, SCIENCE, V344, P921, DOI 10.1126/science.1252510

Ginhoux F, 2016, IMMUNITY, V44, P439, DOI 10.1016/j.immuni.2016.02.024

Ginhoux F, 2010, SCIENCE, V330, P841, DOI 10.1126/science.1194637

Goldmann T, 2016, NAT IMMUNOL, V17, P797, DOI 10.1038/ni.3423

Gouon-Evans V, 2000, DEVELOPMENT, V127, P2269

Güç E, 2021, IMMUNITY, V54, P885, DOI 10.1016/j.immuni.2021.03.022

Guerriero JL, 2017, NATURE, V543, P428, DOI 10.1038/nature21409

Gyorki DE, 2009, BREAST CANCER RES, V11, DOI 10.1186/bcr2353

Hamilton MJ, 2010, J IMMUNOL, V185, P4545, DOI 10.4049/jimmunol.1002045

Hey J, 2023, INT J CANCER, V152, P1226, DOI 10.1002/ijc.34364

Hirano R, 2023, COMMUN BIOL, V6, DOI 10.1038/s42003-023-04525-7

Hoeffel G, 2015, IMMUNITY, V42, P665, DOI 10.1016/j.immuni.2015.03.011

Hüsemann Y, 2008, CANCER CELL, V13, P58, DOI 10.1016/j.ccr.2007.12.003

Ingman WV, 2006, DEV DYNAM, V235, P3222, DOI 10.1002/dvdy.20972

Iwase T, 2016, CANCER MED-US, V5, P41, DOI 10.1002/cam4.571

Janiszewski PM, 2010, OBESITY, V18, P1183, DOI 10.1038/oby.2009.336

Jäppinen N, 2019, NAT COMMUN, V10, DOI 10.1038/s41467-018-08065-1

Johnson RW, 2015, PHARMACOL THERAPEUT, V150, P169, DOI 10.1016/j.pharmthera.2015.02.002

Katzenelenbogen Y, 2020, CELL, V182, P872, DOI 10.1016/j.cell.2020.06.032

Khan F, 2023, J CLIN INVEST, V133, DOI 10.1172/JCI163446

Kim A, 2017, SCI REP-UK, V7, DOI 10.1038/s41598-017-11905-7

Kim OH, 2013, MOL CELLS, V36, P432, DOI 10.1007/s10059-013-0194-7

Kramer ED, 2023, JCI INSIGHT, V8, DOI 10.1172/jci.insight.160978

Kuang DM, 2009, J EXP MED, V206, P1327, DOI 10.1084/jem.20082173

Kurozumi S, 2019, SCI REP-UK, V9, DOI 10.1038/s41598-019-52944-6

Laviron M, 2022, CELL REP, V39, DOI 10.1016/j.celrep.2022.110865

Laviron M, 2019, FRONT IMMUNOL, V10, DOI 10.3389/fimmu.2019.01201

Lee CH, 2010, J IMMUNOTHER, V33, P73, DOI 10.1097/CJI.0b013e3181b7a0a4

Lewis CE, 2006, CANCER RES, V66, P605, DOI 10.1158/0008-5472.CAN-05-4005

Li DB, 2020, CANCER SCI, V111, P47, DOI 10.1111/cas.14230

Li TW, 2020, NUCLEIC ACIDS RES, V48, pW509, DOI 10.1093/nar/gkaa407

Lin H, 2018, J CLIN INVEST, V128, P805, DOI 10.1172/JCI96113

Linde N, 2018, NAT COMMUN, V9, DOI 10.1038/s41467-017-02481-5

Liu Z, 2022, ONCOIMMUNOLOGY, V11, DOI 10.1080/2162402X.2022.2085432

Loyher PL, 2018, J EXP MED, V215, P2536, DOI 10.1084/jem.20180534

Mantovani A, 2004, TRENDS IMMUNOL, V25, P677, DOI 10.1016/j.it.2004.09.015

Mass E, 2023, NAT REV IMMUNOL, DOI 10.1038/s41577-023-00848-y

Mass E, 2018, INT IMMUNOL, V30, P493, DOI 10.1093/intimm/dxy044

Mass E, 2016, SCIENCE, V353, DOI 10.1126/science.aaf4238

Masuda T, 2022, NATURE, V604, P740, DOI 10.1038/s41586-022-04596-2

Matlack R, 2006, IMMUNOLOGY, V117, P386, DOI 10.1111/j.1365-2567.2005.02312.x

Matusiak Magdalena, 2023, Res Sq, DOI 10.21203/rs.3.rs-2393443/v1

Mehraj U, 2021, CELL ONCOL, V44, P1209, DOI 10.1007/s13402-021-00634-9

Mehta AK, 2021, FRONT IMMUNOL, V12, DOI 10.3389/fimmu.2021.643771

Mehta AK, 2021, NAT CANCER, V2, P66, DOI 10.1038/s43018-020-00148-7

Molgora M, 2020, CELL, V182, P886, DOI 10.1016/j.cell.2020.07.013

Morrissey SM, 2021, CELL METAB, V33, P2040, DOI 10.1016/j.cmet.2021.09.002

Mulder K, 2021, IMMUNITY, V54, P1883, DOI 10.1016/j.immuni.2021.07.007

Nguyen AV, 2002, DEV BIOL, V247, P11, DOI 10.1006/dbio.2002.0669

O'Brien J, 2012, DEVELOPMENT, V139, P269, DOI 10.1242/dev.071696

Onkar S, 2023, NAT CANCER, V4, P582, DOI 10.1038/s43018-023-00549-4

Pal B, 2021, EMBO J, V40, DOI 10.15252/embj.2020107333

Perdiguero EG, 2015, NATURE, V518, P547, DOI 10.1038/nature13989

Petty AJ, 2021, JCI INSIGHT, V6, DOI 10.1172/jci.insight.146707

Ramos RN, 2022, CELL, V185, P1189, DOI 10.1016/j.cell.2022.02.021

Romano V, 2022, INT J MOL SCI, V23, DOI 10.3390/ijms23126875

Savas P, 2016, NAT REV CLIN ONCOL, V13, P228, DOI 10.1038/nrclinonc.2015.215

Schepisi G, 2023, CANCERS, V15, DOI 10.3390/cancers15051597

Song SR, 2022, FRONT IMMUNOL, V13, DOI 10.3389/fimmu.2022.978909

Suganami T, 2005, ARTERIOSCL THROM VAS, V25, P2062, DOI 10.1161/01.ATV.0000183883.72263.13

Sullivan AR, 2014, J MAMMARY GLAND BIOL, V19, P149, DOI 10.1007/s10911-014-9320-1

Sun XZ, 2012, BREAST CANCER RES TR, V131, P1003, DOI 10.1007/s10549-011-1789-3

Tan ZY, 2022, FRONT IMMUNOL, V13, DOI 10.3389/fimmu.2022.868813

Teschendorff AE, 2007, GENOME BIOL, V8, DOI 10.1186/gb-2007-8-8-r157

Thomas R, 2021, FRONT ONCOL, V10, DOI 10.3389/fonc.2020.600573

Thu MS, 2023, FRONT ONCOL, V13, DOI 10.3389/fonc.2023.1144021

Tichet M, 2023, IMMUNITY, V56, P162, DOI 10.1016/j.immuni.2022.12.006

Timperi E, 2022, CANCER RES, V82, P3291, DOI 10.1158/0008-5472.CAN-22-1427

Torres-Sanchez A, 2023, FRONT ONCOL, V13, DOI 10.3389/fonc.2023.1152458

Wagner J, 2019, CELL, V177, P1330, DOI 10.1016/j.cell.2019.03.005

WALKER NI, 1989, AM J ANAT, V185, P19, DOI 10.1002/aja.1001850104

Wang N, 2018, CELL DEATH DIS, V9, DOI 10.1038/s41419-018-0876-3

Wang Y, 2022, J HEMATOL ONCOL, V15, DOI 10.1186/s13045-022-01335-y

Wang Y, 2020, ELIFE, V9, DOI 10.7554/eLife.57438

Wu L, 2022, FRONT IMMUNOL, V13, DOI 10.3389/fimmu.2022.973935

Wu SZ, 2021, NAT GENET, V53, P1334, DOI 10.1038/s41588-021-00911-1

Wyckoff JB, 2007, CANCER RES, V67, P2649, DOI 10.1158/0008-5472.CAN-06-1823

Xia ZA, 2022, CANCERS, V14, DOI 10.3390/cancers14225506

Zeng WF, 2023, ONCOGENE, V42, P224, DOI 10.1038/s41388-022-02540-2

Zhang WL, 2019, BRIT J CANCER, V121, P837, DOI 10.1038/s41416-019-0578-3

Zhong JX, 2023, FRONT ONCOL, V13, DOI 10.3389/fonc.2023.1077342

Zhu Y, 2017, IMMUNITY, V47, P323, DOI 10.1016/j.immuni.2017.07.014

NR 108

TC 1

Z9 1

U1 6

U2 6

PU FRONTIERS MEDIA SA

PI LAUSANNE

PA AVENUE DU TRIBUNAL FEDERAL 34, LAUSANNE, CH-1015, SWITZERLAND

SN 1664-3224

J9 FRONT IMMUNOL

JI Front. Immunol.

PD JUL 22

PY 2024

VL 15

AR 1375528

DI 10.3389/fimmu.2024.1375528

PG 9

WC Immunology

WE Science Citation Index Expanded (SCI-EXPANDED)

SC Immunology

GA A6I8F

UT WOS:001283560200001

PM 39104525

OA Green Published, gold

DA 2025-02-07

ER

PT J

AU Vogel, A

Weichhart, T

AF Vogel, Andrea

Weichhart, Thomas

TI Tissue-resident macrophages - early passengers or drivers in the tumor

niche?

SO CURRENT OPINION IN BIOTECHNOLOGY

LA English

DT Review

ID SINGLE-CELL; HEALTH; ATLAS

AB Macrophages within the tumor microenvironment of solid tumors and metastasis are heterogeneous populations, which contribute to diverse steps of tumorigenesis. Tumor-associated macrophages (TAMs) can either derive from circulation-derived monocytes or tissue-resident macrophages (TRMs). In health, TRMs populate the majority of tissues, orchestrating critical homeostatic and reparative functions. While TRM-specific functions in tumor initiation and progression remain unclear, recent studies have revealed that TRMs are a significant source of TAMs in both mouse and human cancers, where they closely resemble gene signatures of their normal, organ-specific TRM counterparts. In this review, we highlight recent advances toward systematically understanding the role of TRMs as an important TAM subset and opportunities how this macrophage population could be exploited for therapeutical targeting strategies.

C1 [Vogel, Andrea; Weichhart, Thomas] Med Univ Vienna, Inst Med Genet, Ctr Pathobiochem & Genet, Vienna, Austria.

C3 Medical University of Vienna

RP Weichhart, T (corresponding author), Med Univ Vienna, Inst Med Genet, Ctr Pathobiochem & Genet, Vienna, Austria.

EM thomas.weichhart@meduniwien.ac.at

RI Weichhart, Thomas/J-3531-2014

OI Vogel, Andrea/0000-0002-2613-140X

FU Austrian Science Fund (FWF) [P34023-B, LS18-058]; Vienna Science and

Technology Fund (WWTF); FWF Sonderforschungsbereich F83; [P34266FW];

Austrian Science Fund (FWF) [P34023] Funding Source: Austrian Science

Fund (FWF)

FX We apologize to colleagues whose work we could not cite due to space

constraints. The graphical abstract and figure were created by using

Biorender. This work was supported by funds from the following sources:

the Austrian Science Fund (FWF) Grants P34266FW, P34023-B, FWF

Sonderforschungsbereich F83, and the Vienna Science and Technology Fund

(WWTF) Grant LS18-058.

CR Aegerter H, 2022, IMMUNITY, V55, P1564, DOI 10.1016/j.immuni.2022.08.010

Antunes ARP, 2021, NAT NEUROSCI, V24, P595, DOI 10.1038/s41593-020-00789-y

Bi K, 2021, CANCER CELL, V39, P649, DOI 10.1016/j.ccell.2021.02.015

Bleriot C, 2020, IMMUNITY, V52, P957, DOI 10.1016/j.immuni.2020.05.014

Casanova-Acebes M, 2021, NATURE, V595, P578, DOI 10.1038/s41586-021-03651-8

Cassetta L, 2023, NAT REV CANCER, V23, P238, DOI 10.1038/s41568-022-00547-1

Cassetta L, 2019, CANCER CELL, V35, P588, DOI 10.1016/j.ccell.2019.02.009

Chakarov S, 2019, SCIENCE, V363, P1190, DOI 10.1126/science.aau0964

Cheng SJ, 2021, CELL, V184, P792, DOI 10.1016/j.cell.2021.01.010

Chow A, 2021, CANCER CELL, V39, P973, DOI 10.1016/j.ccell.2021.05.006

Dick SA, 2022, SCI IMMUNOL, V7, DOI 10.1126/sciimmunol.abf7777

Etzerodt A, 2020, J EXP MED, V217, DOI 10.1084/jem.20191869

Friedrich M, 2021, NAT CANCER, V2, P723, DOI 10.1038/s43018-021-00201-z

Gangoso E, 2021, CELL, V184, P2454, DOI 10.1016/j.cell.2021.03.023

Ginhoux F, 2016, IMMUNITY, V44, P439, DOI 10.1016/j.immuni.2016.02.024

Guilliams M, 2021, NAT IMMUNOL, V22, P118, DOI 10.1038/s41590-020-00849-2

Guilliams M, 2017, NAT REV IMMUNOL, V17, P451, DOI 10.1038/nri.2017.42

Hutter G, 2019, P NATL ACAD SCI USA, V116, P997, DOI 10.1073/pnas.1721434116

Karimi E, 2023, NATURE, V614, P555, DOI 10.1038/s41586-022-05680-3

Lavin Y, 2014, CELL, V159, P1312, DOI 10.1016/j.cell.2014.11.018

Laviron M, 2022, CELL REP, V39, DOI 10.1016/j.celrep.2022.110865

Laviron M, 2019, FRONT IMMUNOL, V10, DOI 10.3389/fimmu.2019.01799

Lehmann B, 2017, SCI IMMUNOL, V2, DOI 10.1126/sciimmunol.aah6413

Linde N, 2018, NAT COMMUN, V9, DOI 10.1038/s41467-017-02481-5

Liu HL, 2021, CELL REP, V36, DOI 10.1016/j.celrep.2021.109718

Loyher PL, 2018, J EXP MED, V215, P2536, DOI 10.1084/jem.20180534

Ma RY, 2022, TRENDS IMMUNOL, V43, P546, DOI 10.1016/j.it.2022.04.008

Mantovani A, 2017, NAT REV CLIN ONCOL, V14, P399, DOI 10.1038/nrclinonc.2016.217

Martinez-Usatorre A, 2021, SCI TRANSL MED, V13, DOI 10.1126/scitranslmed.abd1616

Mass E, 2023, NAT REV IMMUNOL, DOI 10.1038/s41577-023-00848-y

Müller A, 2015, INT J CANCER, V137, P278, DOI 10.1002/ijc.29379

Mulder K, 2021, IMMUNITY, V54, P1883, DOI 10.1016/j.immuni.2021.07.007

Pittet MJ, 2022, NAT REV CLIN ONCOL, V19, P402, DOI 10.1038/s41571-022-00620-6

Ramos RN, 2022, CELL, V185, P1189, DOI 10.1016/j.cell.2022.02.021

Salmon H, 2019, NAT REV CANCER, V19, P215, DOI 10.1038/s41568-019-0125-9

Sharma A, 2020, CELL, V183, P377, DOI 10.1016/j.cell.2020.08.040

Singhal S, 2019, SCI TRANSL MED, V11, DOI 10.1126/scitranslmed.aat1500

Siwicki M, 2021, SCI IMMUNOL, V6, DOI 10.1126/sciimmunol.abi7083

Sorin M, 2023, NATURE, V614, P548, DOI 10.1038/s41586-022-05672-3

van Elsas MJ, 2023, J Immunother Cancer, P11

Wu SZ, 2021, NAT GENET, V53, P1334, DOI 10.1038/s41588-021-00911-1

Zhang L, 2020, CELL, V181, P442, DOI 10.1016/j.cell.2020.03.048

Zhang QW, 2012, PLOS ONE, V7, DOI 10.1371/journal.pone.0050946

Zhu Y, 2017, IMMUNITY, V47, P323, DOI 10.1016/j.immuni.2017.07.014

Zilionis R, 2019, IMMUNITY, V50, P1317, DOI 10.1016/j.immuni.2019.03.009

NR 45

TC 4

Z9 4

U1 0

U2 3

PU ELSEVIER SCI LTD

PI London

PA 125 London Wall, London, ENGLAND

SN 0958-1669

EI 1879-0429

J9 CURR OPIN BIOTECH

JI Curr. Opin. Biotechnol.

PD OCT

PY 2023

VL 83

AR 102984

DI 10.1016/j.copbio.2023.102984

EA AUG 2023

PG 6

WC Biochemical Research Methods; Biotechnology & Applied Microbiology

WE Science Citation Index Expanded (SCI-EXPANDED)

SC Biochemistry & Molecular Biology; Biotechnology & Applied Microbiology

GA Q6NN6

UT WOS:001058673200001

PM 37572419

OA hybrid

DA 2025-02-07

ER

PT J

AU Cao, MM

Wang, ZH

Lan, WY

Xiang, BH

Liao, WJ

Zhou, J

Liu, XM

Wang, YL

Zhang, SC

Lu, S

Lang, JY

Zhao, Y

AF Cao, Minmin

Wang, Zihao

Lan, Wanying

Xiang, Binghua

Liao, Wenjun

Zhou, Jie

Liu, Xiaomeng

Wang, Yiling

Zhang, Shichuan

Lu, Shun

Lang, Jinyi

Zhao, Yue

TI The roles of tissue resident macrophages in health and cancer

SO EXPERIMENTAL HEMATOLOGY & ONCOLOGY

LA English

DT Review

DE Tissue resident macrophages; Bone-marrow derived macrophages; Monocytes;

Homeostasis; Cancer

ID TUMOR-ASSOCIATED MACROPHAGES; COLONY-STIMULATING FACTOR; DENDRITIC CELL

SUBSETS; LANGERHANS CELLS; INTESTINAL MACROPHAGES; GLIOBLASTOMA

INVASION; THERAPEUTIC TARGET; DERMAL MACROPHAGES; BRAIN METASTASIS;

MICROGLIA EMERGE

AB As integral components of the immune microenvironment, tissue resident macrophages (TRMs) represent a self-renewing and long-lived cell population that plays crucial roles in maintaining homeostasis, promoting tissue remodeling after damage, defending against inflammation and even orchestrating cancer progression. However, the exact functions and roles of TRMs in cancer are not yet well understood. TRMs exhibit either pro-tumorigenic or anti-tumorigenic effects by engaging in phagocytosis and secreting diverse cytokines, chemokines, and growth factors to modulate the adaptive immune system. The life-span, turnover kinetics and monocyte replenishment of TRMs vary among different organs, adding to the complexity and controversial findings in TRMs studies. Considering the complexity of tissue associated macrophage origin, macrophages targeting strategy of each ontogeny should be carefully evaluated. Consequently, acquiring a comprehensive understanding of TRMs' origin, function, homeostasis, characteristics, and their roles in cancer for each specific organ holds significant research value. In this review, we aim to provide an outline of homeostasis and characteristics of resident macrophages in the lung, liver, brain, skin and intestinal, as well as their roles in modulating primary and metastatic cancer, which may inform and serve the future design of targeted therapies.

C1 [Cao, Minmin] Chengdu Univ Tradit Chinese Med, Sch Basic Med Sci, Chengdu, Peoples R China.

[Wang, Zihao; Lan, Wanying; Xiang, Binghua; Liao, Wenjun; Zhou, Jie; Liu, Xiaomeng; Wang, Yiling; Zhang, Shichuan; Lu, Shun; Lang, Jinyi; Zhao, Yue] Univ Elect Sci & Technol China, Dept Radiat Oncol,Affiliated Canc Hosp, Radiat Oncol Key Lab Sichuan Prov,Sichuan Canc Ctr, Sichuan Clin Res Ctr Canc,Sichuan Canc Hosp & Inst, Chengdu, Peoples R China.

[Wang, Zihao; Xiang, Binghua] Univ Elect Sci & Technol China, Sch Med, Chengdu, Peoples R China.

[Lan, Wanying] Guixi Community Hlth Ctr Chengdu High Tech Zone, Chengdu, Peoples R China.

C3 Chengdu University of Traditional Chinese Medicine; University of

Electronic Science & Technology of China; University of Electronic

Science & Technology of China

RP Zhao, Y (corresponding author), Univ Elect Sci & Technol China, Dept Radiat Oncol,Affiliated Canc Hosp, Radiat Oncol Key Lab Sichuan Prov,Sichuan Canc Ctr, Sichuan Clin Res Ctr Canc,Sichuan Canc Hosp & Inst, Chengdu, Peoples R China.

EM Zhaoyueyeah@126.com

RI Wang, Yiling/GLT-8762-2022

FU Sichuan Provincial Cadre Health Research Project

FX The figures were created with BioRender.com.

CR Aegerter H, 2022, IMMUNITY, V55, P1564, DOI 10.1016/j.immuni.2022.08.010

Aizarani N, 2019, NATURE, V572, P199, DOI 10.1038/s41586-019-1373-2

Akkari L, 2020, SCI TRANSL MED, V12, DOI 10.1126/scitranslmed.aaw7843

Amici SA, 2017, FRONT IMMUNOL, V8, DOI 10.3389/fimmu.2017.01520

[Anonymous], 2018, J HEPATOL, V69, P1197, DOI 10.1016/j.jhep.2018.02.013

Atmatzidis DH, 2017, J EUR ACAD DERMATOL, V31, P1817, DOI 10.1111/jdv.14522

Baer JM, 2023, NAT IMMUNOL, V24, P1443, DOI 10.1038/s41590-023-01579-x

Bain CC, 2013, MUCOSAL IMMUNOL, V6, P498, DOI 10.1038/mi.2012.89

Bain CC, 2018, FRONT IMMUNOL, V9, DOI 10.3389/fimmu.2018.02733

Bain CC, 2014, NAT IMMUNOL, V15, P929, DOI 10.1038/ni.2967

Barlow JL, 2019, ANNU REV PHYSIOL, V81, P429, DOI 10.1146/annurev-physiol-020518-114630

Becerril-García MA, 2020, FRONT IMMUNOL, V11, DOI 10.3389/fimmu.2020.00744

Bellmann L, 2021, J INVEST DERMATOL, V141, P84, DOI 10.1016/j.jid.2020.05.098

Benbenishty A, 2019, PLOS BIOL, V17, DOI 10.1371/journal.pbio.2006859

Benmamar-Badel A, 2020, FRONT IMMUNOL, V11, DOI 10.3389/fimmu.2020.00430

Bennett H, 2021, FRONT IMMUNOL, V11, DOI 10.3389/fimmu.2020.609618

Bennett ML, 2016, P NATL ACAD SCI USA, V113, pE1738, DOI 10.1073/pnas.1525528113

Bettinger I, 2002, ACTA NEUROPATHOL, V103, P351, DOI 10.1007/s00401-001-0472-x

Bissonnette EY, 2020, FRONT IMMUNOL, V11, DOI 10.3389/fimmu.2020.583042

Blériot C, 2021, IMMUNITY, V54, P2101, DOI 10.1016/j.immuni.2021.08.006

Bleriot C, 2020, IMMUNITY, V52, P957, DOI 10.1016/j.immuni.2020.05.014

Böttcher C, 2019, NAT NEUROSCI, V22, P78, DOI 10.1038/s41593-018-0290-2

Bonnardel J, 2019, IMMUNITY, V51, P638, DOI 10.1016/j.immuni.2019.08.017

Bowman RL, 2016, CELL REP, V17, P2445, DOI 10.1016/j.celrep.2016.10.052

Branchett WJ, 2019, MUCOSAL IMMUNOL, V12, P589, DOI 10.1038/s41385-019-0158-0

Buechler MB, 2019, IMMUNITY, V51, P119, DOI 10.1016/j.immuni.2019.05.010

Bujko A, 2018, J EXP MED, V215, P441, DOI 10.1084/jem.20170057

Butovsky O, 2018, NAT REV NEUROSCI, V19, P622, DOI 10.1038/s41583-018-0057-5

Butovsky O, 2012, J CLIN INVEST, V122, P3063, DOI 10.1172/JCI62636

Buttgereit A, 2016, NAT IMMUNOL, V17, P1397, DOI 10.1038/ni.3585

Capucha T, 2015, IMMUNITY, V43, P369, DOI 10.1016/j.immuni.2015.06.017

da Fonseca ACC, 2014, J NEUROIMMUNOL, V274, P71, DOI 10.1016/j.jneuroim.2014.06.021

Casanova-Acebes M, 2021, CANCER DISCOV, V11, P1873, DOI 10.1158/2159-8290.CD-RW2021-089

Casanova-Acebes M, 2021, NATURE, V595, P578, DOI 10.1038/s41586-021-03651-8

Chakarov S, 2019, SCIENCE, V363, P1190, DOI 10.1126/science.aau0964

Cheng SJ, 2021, CELL, V184, P792, DOI 10.1016/j.cell.2021.01.010

Cherry JD, 2014, J NEUROINFLAMM, V11, DOI 10.1186/1742-2094-11-98

Chopin M, 2013, J EXP MED, V210, P2967, DOI 10.1084/jem.20130930

Chorro L, 2009, J EXP MED, V206, P3089, DOI 10.1084/jem.20091586

Chow A, 2021, CANCER CELL, V39, P973, DOI 10.1016/j.ccell.2021.05.006

Christofides A, 2022, NAT IMMUNOL, V23, P1148, DOI 10.1038/s41590-022-01267-2

Colegio OR, 2014, NATURE, V513, P559, DOI 10.1038/nature13490

Collin M, 2021, IMMUNITY, V54, P2188, DOI 10.1016/j.immuni.2021.09.006

Coniglio SJ, 2012, MOL MED, V18, P519, DOI 10.2119/molmed.2011.00217

Cortez-Retamozo V, 2012, P NATL ACAD SCI USA, V109, P2491, DOI 10.1073/pnas.1113744109

Jordao MJC, 2019, SCIENCE, V363, P365, DOI 10.1126/science.aat7554

Cox N, 2021, ANNU REV IMMUNOL, V39, P313, DOI 10.1146/annurev-immunol-093019-111748

Dal-Secco D, 2015, J EXP MED, V212, P447, DOI 10.1084/jem.20141539

De Schepper S, 2018, CELL, V175, P400, DOI 10.1016/j.cell.2018.07.048

De Simone G, 2021, IMMUNITY, V54, P2089, DOI 10.1016/j.immuni.2021.05.005

Dick SA, 2019, NAT IMMUNOL, V20, P29, DOI [10.1038/s41590-018-0272-2, 10.1038/s41590-019-0363-8]

Dou L, 2020, FRONT IMMUNOL, V10, DOI 10.3389/fimmu.2019.03112

DRANOFF G, 1994, SCIENCE, V264, P713, DOI 10.1126/science.8171324

Edin S, 2013, PLOS ONE, V8, DOI 10.1371/journal.pone.0074982

Elmore MRP, 2014, NEURON, V82, P380, DOI 10.1016/j.neuron.2014.02.040

Engblom C, 2016, NAT REV CANCER, V16, P447, DOI 10.1038/nrc.2016.54

Etzerodt A, 2020, J EXP MED, V217, DOI 10.1084/jem.20191869

Evren E, 2022, J EXP MED, V219, DOI 10.1084/jem.20210987

Farro G, 2016, NEUROGASTROENT MOTIL, V28, P934, DOI 10.1111/nmo.12796

Fehres CM, 2017, CELL MOL IMMUNOL, V14, P360, DOI 10.1038/cmi.2015.87

Feinberg PA, 2022, J NEUROSCI, V42, P6171, DOI 10.1523/JNEUROSCI.0601-22.2022

Feuerstein R, 2017, J LEUKOCYTE BIOL, V101, P99, DOI 10.1189/jlb.3MR0316-097RR

Fidler IJ, 2015, CANCER J, V21, P284, DOI 10.1097/PPO.0000000000000126

Foo SL, 2022, BREAST CANCER RES, V24, DOI 10.1186/s13058-022-01514-2

Frade JM, 1998, NEURON, V20, P35, DOI 10.1016/S0896-6273(00)80432-8

Franklin RA, 2014, SCIENCE, V344, P921, DOI 10.1126/science.1252510

Franz E, 2020, CELL DEATH DISCOV, V6, DOI 10.1038/s41420-020-00350-7

Fritz JM, 2014, FRONT IMMUNOL, V5, DOI 10.3389/fimmu.2014.00587

Froh M, 2002, AM J PHYSIOL-GASTR L, V283, pG856, DOI 10.1152/ajpgi.00503.2001

Fujita H, 2012, J INVEST DERMATOL, V132, P1645, DOI 10.1038/jid.2012.34

Gabanyi I, 2016, CELL, V164, P378, DOI 10.1016/j.cell.2015.12.023

Gammella E, 2014, METALLOMICS, V6, P1336, DOI 10.1039/c4mt00104d

García-Pérez R, 2018, SCI REP-UK, V8, DOI 10.1038/s41598-018-26082-4

Ginhoux F, 2016, IMMUNITY, V44, P439, DOI 10.1016/j.immuni.2016.02.024

Ginhoux F, 2016, NAT IMMUNOL, V17, P34, DOI 10.1038/ni.3324

Ginhoux F, 2014, NAT REV IMMUNOL, V14, P392, DOI 10.1038/nri3671

Ginhoux F, 2013, FRONT CELL NEUROSCI, V7, DOI 10.3389/fncel.2013.00045

Ginhoux F, 2010, SCIENCE, V330, P841, DOI 10.1126/science.1194637

Guilliams M, 2013, J EXP MED, V210, P1977, DOI 10.1084/jem.20131199

Guldner IH, 2020, CELL, V183, P1234, DOI 10.1016/j.cell.2020.09.064

Guo SS, 2022, EXPERT REV MOL MED, V24, DOI 10.1017/erm.2022.8

Haage V, 2020, ACTA NEUROPATHOL COM, V8, DOI 10.1186/s40478-019-0875-3

Hammond TR, 2019, IMMUNITY, V50, P253, DOI 10.1016/j.immuni.2018.11.004

He BP, 2006, MOL MED, V12, P161, DOI 10.2119/2006-00033.He

Heymann F, 2015, HEPATOLOGY, V62, P279, DOI 10.1002/hep.27793

Hiemstra IH, 2014, IMMUNOLOGY, V142, P269, DOI 10.1111/imm.12251

Hine AM, 2019, J IMMUNOL, V203, P593, DOI 10.4049/jimmunol.1900345

Ho WZ, 1997, J IMMUNOL, V159, P5654

Hochgerner M, 2022, J INVEST DERMATOL, V142, P2446, DOI 10.1016/j.jid.2022.02.014

Hoeffel G, 2012, J EXP MED, V209, P1167, DOI 10.1084/jem.20120340

Hohsfield LA, 2021, ELIFE, V10, DOI 10.7554/eLife.66738

Horev Y, 2020, MUCOSAL IMMUNOL, V13, P767, DOI 10.1038/s41385-020-0301-y

Hovav AH, 2018, TRENDS IMMUNOL, V39, P788, DOI 10.1016/j.it.2018.08.007

Howell R, 2023, SCI ADV, V9, DOI 10.1126/sciadv.add1992

Huang LT, 2020, FRONT IMMUNOL, V11, DOI 10.3389/fimmu.2020.00912

Huang YB, 2018, NAT NEUROSCI, V21, P530, DOI 10.1038/s41593-018-0090-8

Hume DA, 2019, TRENDS IMMUNOL, V40, P98, DOI 10.1016/j.it.2018.11.007

Hume PS, 2020, AM J RESP CRIT CARE, V201, P1209, DOI 10.1164/rccm.201911-2105OC

Hussell T, 2014, NAT REV IMMUNOL, V14, P81, DOI 10.1038/nri3600

Hutter G, 2019, P NATL ACAD SCI USA, V116, P997, DOI 10.1073/pnas.1721434116

Jacome-Galarza CE, 2019, NATURE, V568, P541, DOI 10.1038/s41586-019-1105-7

Jin Y, 2022, SIGNAL TRANSDUCT TAR, V7, DOI 10.1038/s41392-022-00872-9

Jurga AM, 2020, FRONT CELL NEUROSCI, V14, DOI 10.3389/fncel.2020.00198

Karimi E, 2023, NATURE, V614, P555, DOI 10.1038/s41586-022-05680-3

Kenkhuis B, 2022, NEUROBIOL DIS, V167, DOI 10.1016/j.nbd.2022.105684

Khan F, 2023, J CLIN INVEST, V133, DOI 10.1172/JCI163446

Kierdorf K, 2013, NAT NEUROSCI, V16, P273, DOI 10.1038/nn.3318

Kim N, 2020, NAT COMMUN, V11, DOI 10.1038/s41467-020-16164-1

Kolter J, 2019, IMMUNITY, V50, P1482, DOI 10.1016/j.immuni.2019.05.009

Kozaka S, 2020, MOL PHARMACEUT, V17, P645, DOI 10.1021/acs.molpharmaceut.9b01104

Kramer ED, 2023, JCI INSIGHT, V8, DOI 10.1172/jci.insight.160978

Krenkel O, 2017, NAT REV IMMUNOL, V17, P306, DOI 10.1038/nri.2017.11

KUHN R, 1993, CELL, V75, P263, DOI 10.1016/0092-8674(93)80068-P

Lalancette-Hébert M, 2007, J NEUROSCI, V27, P2596, DOI 10.1523/JNEUROSCI.5360-06.2007

Lavin Y, 2014, CELL, V159, P1312, DOI 10.1016/j.cell.2014.11.018

Lazarov T, 2023, NATURE, V618, P698, DOI 10.1038/s41586-023-06002-x

Leach SM, 2020, CELL REP, V33, DOI 10.1016/j.celrep.2020.108337

Lee SH, 2018, J EXP MED, V215, P357, DOI 10.1084/jem.20171389

Lefere S, 2019, J HEPATOL, V71, P631, DOI 10.1016/j.jhep.2019.03.016

Lelli A, 2013, GLIA, V61, P1542, DOI 10.1002/glia.22540

Li DF, 2022, FRONT ONCOL, V12, DOI 10.3389/fonc.2022.1000807

Li H, 2012, HEPATOLOGY, V56, P1342, DOI 10.1002/hep.25777

Li LW, 2021, JCI INSIGHT, V6, DOI 10.1172/jci.insight.144394

Li QY, 2019, NEURON, V101, P207, DOI 10.1016/j.neuron.2018.12.006

Lim SY, 2016, ONCOGENE, V35, P5735, DOI 10.1038/onc.2016.107

Liu W, 2023, J CLIN INVEST, V133, DOI 10.1172/JCI157937

Liu XC, 2021, IMMUNITY, V54, P2305, DOI 10.1016/j.immuni.2021.08.012

Lonardi S, 2020, CANCER IMMUNOL RES, V8, P829, DOI 10.1158/2326-6066.CIR-19-0232

Louwe PA, 2021, NAT COMMUN, V12, DOI 10.1038/s41467-021-21778-0

Loyher PL, 2018, J EXP MED, V215, P2536, DOI 10.1084/jem.20180534

Luo Y, 2019, ALLERGY, V74, P1738, DOI 10.1111/all.13813

Ma HY, 2020, J HEPATOL, V72, P946, DOI 10.1016/j.jhep.2019.12.016

Ma RY, 2022, TRENDS IMMUNOL, V43, P546, DOI 10.1016/j.it.2022.04.008

Ma RY, 2020, J EXP MED, V217, DOI 10.1084/jem.20191820

Maarifi G, 2020, CELL MOL IMMUNOL, V17, P547, DOI 10.1038/s41423-019-0302-5

MacParland SA, 2018, NAT COMMUN, V9, DOI 10.1038/s41467-018-06318-7

Maraee A, 2020, ECANCERMEDICALSCIENC, V14, DOI 10.3332/ecancer.2020.1045

Marques-da-Silva C, 2011, IMMUNOBIOLOGY, V216, P1, DOI 10.1016/j.imbio.2010.03.010

Marschall P, 2021, J ALLERGY CLIN IMMUN, V147, P1778, DOI 10.1016/j.jaci.2020.10.006

Mass E, 2023, NAT REV IMMUNOL, DOI 10.1038/s41577-023-00848-y

Mass E, 2016, SCIENCE, V353, DOI 10.1126/science.aaf4238

Masuda T, 2019, NATURE, V568, pE4, DOI [10.1038/s41586-019-0924-x, 10.1038/s41586-019-1045-2]

Matsumura H, 2014, INT J ONCOL, V45, P2303, DOI 10.3892/ijo.2014.2662

Mielcarek M, 2014, TRANSPLANTATION, V98, P563, DOI 10.1097/TP.0000000000000097

Misharin AV, 2013, AM J RESP CELL MOL, V49, P503, DOI 10.1165/rcmb.2013-0086MA

Mizumoto N, 2004, J CLIN INVEST, V113, P658, DOI 10.1172/JCI200421140

Modi BG, 2012, SCIENCE, V335, P104, DOI 10.1126/science.1211600

Moore KJ, 2013, NAT REV IMMUNOL, V13, P709, DOI 10.1038/nri3520

Mossanen JC, 2016, HEPATOLOGY, V64, P1667, DOI 10.1002/hep.28682

Mowat AM, 2017, NAT MED, V23, P1258, DOI 10.1038/nm.4430

Mulder K, 2021, IMMUNITY, V54, P1883, DOI 10.1016/j.immuni.2021.07.007

Muller PA, 2020, CURR OPIN IMMUNOL, V62, P54, DOI 10.1016/j.coi.2019.11.011

Muller PA, 2014, CELL, V158, P300, DOI 10.1016/j.cell.2014.04.050

Nakanishi R, 2022, INTERNAL MED, V61, P123, DOI 10.2169/internalmedicine.7799-21

Nayak D, 2014, ANNU REV IMMUNOL, V32, P367, DOI 10.1146/annurev-immunol-032713-120240

Neagu M, 2022, J PERS MED, V12, DOI 10.3390/jpm12122072

Nemethova A, 2013, PLOS ONE, V8, DOI 10.1371/journal.pone.0079264

Nobs SP, 2021, TRENDS IMMUNOL, V42, P495, DOI 10.1016/j.it.2021.04.007

Ohnishi K, 2013, CANCER SCI, V104, P1236, DOI 10.1111/cas.12212

Okumura K, 2020, ONCOGENE, V39, P4756, DOI 10.1038/s41388-020-1323-3

Pello OM, 2012, BLOOD, V119, P411, DOI 10.1182/blood-2011-02-339911

Perdiguero EG, 2015, IMMUNITY, V43, P1023, DOI 10.1016/j.immuni.2015.11.022

Perdiguero EG, 2015, NATURE, V518, P547, DOI 10.1038/nature13989

Pogorzelska-Dyrbus J, 2020, MEDIAT INFLAMM, V2020, DOI 10.1155/2020/8745863

Pollard JW, 2009, NAT REV IMMUNOL, V9, P259, DOI 10.1038/nri2528

Prieto LI, 2023, CANCER CELL, V41, P1261, DOI 10.1016/j.ccell.2023.05.006

Pyonteck SM, 2013, NAT MED, V19, P1264, DOI 10.1038/nm.3337

Ramachandran P, 2019, NATURE, V575, P512, DOI 10.1038/s41586-019-1631-3

Ramachandran P, 2020, NAT REV GASTRO HEPAT, V17, P457, DOI 10.1038/s41575-020-0304-x

Ramos RN, 2022, CELL, V185, P1189, DOI 10.1016/j.cell.2022.02.021

Robinson A, 2022, CANCERS, V14, DOI 10.3390/cancers14030833

Romani N, 2010, IMMUNOL REV, V234, P120, DOI 10.1111/j.0105-2896.2009.00886.x

Roumier A, 2004, J NEUROSCI, V24, P11421, DOI 10.1523/JNEUROSCI.2251-04.2004

Rozis G, 2008, IMMUNOLOGY, V124, P329, DOI 10.1111/j.1365-2567.2007.02770.x

Saba Y, 2022, P NATL ACAD SCI USA, V119, DOI 10.1073/pnas.2118424119

Saederup N, 2010, PLOS ONE, V5, DOI 10.1371/journal.pone.0013693

Saeidi V, 2023, FRONT IMMUNOL, V14, DOI 10.3389/fimmu.2023.1084873

Sakai M, 2019, IMMUNITY, V51, P655, DOI 10.1016/j.immuni.2019.09.002

Salter MW, 2017, NAT MED, V23, P1018, DOI 10.1038/nm.4397

Sankowski R, 2019, NAT NEUROSCI, V22, P2098, DOI 10.1038/s41593-019-0532-y

Soto MS, 2018, FRONT CELL NEUROSCI, V12, DOI 10.3389/fncel.2018.00414

Satpathy AT, 2012, J EXP MED, V209, P1135, DOI 10.1084/jem.20120030

Schneider C, 2015, IMMUNITY, V42, P597, DOI 10.1016/j.immuni.2015.04.001

Schultze JL, 2016, CURR OPIN PHARMACOL, V26, P10, DOI 10.1016/j.coph.2015.09.007

Scott CL, 2018, IMMUNITY, V49, P312, DOI 10.1016/j.immuni.2018.07.004

Scott CL, 2016, NAT COMMUN, V7, DOI 10.1038/ncomms10321

Sehgal A, 2018, NAT COMMUN, V9, DOI 10.1038/s41467-018-03638-6

Serezani APM, 2022, AM J RESP CELL MOL, V67, P50, DOI 10.1165/rcmb.2021-0418OC

Shang C, 2022, CANCER LETT, V537, DOI 10.1016/j.canlet.2022.215667

Sharma A, 2020, CELL, V183, P377, DOI 10.1016/j.cell.2020.08.040

Shaw TN, 2018, J EXP MED, V215, P1507, DOI 10.1084/jem.20180019

Sheng JP, 2022, GUT, V71, P1176, DOI 10.1136/gutjnl-2021-324339

Sica A, 2008, SEMIN CANCER BIOL, V18, P349, DOI 10.1016/j.semcancer.2008.03.004

Silvin A, 2018, GLIA, V66, P2045, DOI 10.1002/glia.23458

Siwicki M, 2021, SCI IMMUNOL, V6, DOI 10.1126/sciimmunol.abi7083

Soncin I, 2018, NAT COMMUN, V9, DOI 10.1038/s41467-018-02834-8

Song K, 2020, HEPATOLOGY, V72, P72, DOI 10.1002/hep.30990

Soysa R, 2019, J HEPATOL, V71, P553, DOI 10.1016/j.jhep.2019.04.015

Sparber F, 2018, PLOS PATHOG, V14, DOI 10.1371/journal.ppat.1007069

Speth JM, 2019, JCI INSIGHT, V4, DOI 10.1172/jci.insight.131340

STANLEY E, 1994, P NATL ACAD SCI USA, V91, P5592, DOI 10.1073/pnas.91.12.5592

Stanley ER, 2014, CSH PERSPECT BIOL, V6, DOI 10.1101/cshperspect.a021857

Strobl H, 2019, SEMIN CELL DEV BIOL, V86, P36, DOI 10.1016/j.semcdb.2018.02.016

Swinnen N, 2013, GLIA, V61, P150, DOI 10.1002/glia.22421

Tamoutounour S, 2013, IMMUNITY, V39, P925, DOI 10.1016/j.immuni.2013.10.004

Tanaka R, 2022, J INVEST DERMATOL, V142, P3167, DOI 10.1016/j.jid.2022.06.006

Taniguchi S, 2023, NAT COMMUN, V14, DOI 10.1038/s41467-022-35701-8

Tapmeier TT, 2022, ONCOGENE, V41, P5032, DOI 10.1038/s41388-022-02488-3

Theurl I, 2016, NAT MED, V22, P945, DOI 10.1038/nm.4146

Trapnell BC, 2002, ANNU REV PHYSIOL, V64, P775, DOI 10.1146/annurev.physiol.64.090601.113847

Tay TL, 2017, J PHYSIOL-LONDON, V595, P1929, DOI 10.1113/JP272134

Türler A, 2002, AM J PHYSIOL-GASTR L, V282, pG145, DOI 10.1152/ajpgi.00263.2001

Uchida K, 2007, NEW ENGL J MED, V356, P567, DOI 10.1056/NEJMoa062505

Ueno M, 2013, NAT NEUROSCI, V16, P543, DOI 10.1038/nn.3358

Ural BB, 2020, SCI IMMUNOL, V5, DOI 10.1126/sciimmunol.aax8756

van de Laar L, 2016, IMMUNITY, V44, P755, DOI 10.1016/j.immuni.2016.02.017

Veillette A, 2018, TRENDS IMMUNOL, V39, P173, DOI 10.1016/j.it.2017.12.005

Verney C, 2010, J ANAT, V217, P436, DOI 10.1111/j.1469-7580.2010.01245.x

Viola MF, 2021, GUT, V70, P1383, DOI 10.1136/gutjnl-2020-323121

Viola MF, 2020, NEUROGASTROENT MOTIL, V32, DOI 10.1111/nmo.13843

Wakselman S, 2008, J NEUROSCI, V28, P8138, DOI 10.1523/JNEUROSCI.1006-08.2008

Wamhoff EC, 2019, ACS CENTRAL SCI, V5, P808, DOI 10.1021/acscentsci.9b00093

Wang PL, 2020, NAT COMMUN, V11, DOI 10.1038/s41467-020-16355-w

Wehner S, 2005, SURGERY, V137, P436, DOI 10.1016/j.surg.2004.11.003

Wei C, 2019, MOL CANCER, V18, DOI 10.1186/s12943-019-0976-4

Wen SW, 2013, CANCER BIOL THER, V14, P606, DOI 10.4161/cbt.24593

Wen YK, 2021, CELL MOL IMMUNOL, V18, P45, DOI 10.1038/s41423-020-00558-8

Wesolowska A, 2008, ONCOGENE, V27, P918, DOI 10.1038/sj.onc.1210683

Willingham SB, 2012, P NATL ACAD SCI USA, V109, P6662, DOI 10.1073/pnas.1121623109

Wolf SA, 2017, ANNU REV PHYSIOL, V79, P619, DOI 10.1146/annurev-physiol-022516-034406

Wu J, 2018, NEURO-ONCOLOGY, V20, P92, DOI 10.1093/neuonc/nox111

Wu J, 2012, CANCER RES, V72, P3977, DOI 10.1158/0008-5472.CAN-12-0938

Wu K, 2009, CANCER RES, V69, P8067, DOI 10.1158/0008-5472.CAN-09-0901

Wu SY, 2017, FRONT BIOSCI-LANDMRK, V22, P1805

Wu XD, 2016, J EXP MED, V213, P2553, DOI 10.1084/jem.20160600

Xu-Vanpala S, 2020, SCI IMMUNOL, V5, DOI 10.1126/sciimmunol.aba7350

Yan HH, 2022, J INVEST SURG, V35, P1239, DOI 10.1080/08941939.2021.2024306

Yao Y, 2020, NAT COMMUN, V11, DOI 10.1038/s41467-020-17630-6

Yao Y, 2018, J ALLERGY CLIN IMMUN, V142, P976, DOI 10.1016/j.jaci.2018.04.024

Yona S, 2013, IMMUNITY, V38, P79, DOI 10.1016/j.immuni.2012.12.001

You Q, 2008, HEPATOLOGY, V48, P978, DOI 10.1002/hep.22395

Yu XY, 2017, IMMUNITY, V47, P903, DOI 10.1016/j.immuni.2017.10.007

Yuan DT, 2017, CANCER CELL, V31, P771, DOI 10.1016/j.ccell.2017.05.006

Zhang CY, 2009, CANCER RES, V69, P828, DOI 10.1158/0008-5472.CAN-08-2588

Zhang J, 2012, CARCINOGENESIS, V33, P312, DOI 10.1093/carcin/bgr289

Zhang PP, 2021, J NEUROINFLAMM, V18, DOI 10.1186/s12974-021-02230-y

Zhang X, 2016, ALLERGY, V71, P758, DOI 10.1111/all.12871

Zhang XL, 2022, JCI INSIGHT, V7, DOI 10.1172/jci.insight.150223

Zhou N, 2020, J EUR ACAD DERMATOL, V34, pE230, DOI 10.1111/jdv.16172

Zhou N, 2019, PROTEIN CELL, V10, P87, DOI 10.1007/s13238-018-0599-3

Zhu Y, 2017, IMMUNITY, V47, P323, DOI 10.1016/j.immuni.2017.07.014

Zigmond E, 2013, TRENDS IMMUNOL, V34, P162, DOI 10.1016/j.it.2013.02.001

Zilionis R, 2019, IMMUNITY, V50, P1317, DOI 10.1016/j.immuni.2019.03.009

NR 254

TC 8

Z9 8

U1 8

U2 20

PU BMC

PI LONDON

PA CAMPUS, 4 CRINAN ST, LONDON N1 9XW, ENGLAND

EI 2162-3619

J9 EXP HEMATOL ONCOL

JI Exp. Hematol. Oncol.

PD JAN 16

PY 2024

VL 13

IS 1

AR 3

DI 10.1186/s40164-023-00469-0

PG 22

WC Oncology; Hematology

WE Science Citation Index Expanded (SCI-EXPANDED)

SC Oncology; Hematology

GA FD7U3

UT WOS:001143896100001

PM 38229178

OA gold, Green Published

DA 2025-02-07

ER

PT J

AU Elfstrum, AK

Bapat, AS

Schwertfeger, KL

AF Elfstrum, Alexis K.

Bapat, Aditi S.

Schwertfeger, Kathryn L.

TI Defining and targeting macrophage heterogeneity in the mammary gland and

breast cancer

SO CANCER MEDICINE

LA English

DT Review

DE breast cancer; LYVE-1; macrophage; mammary gland; TREM2

ID TUMOR-ASSOCIATED MACROPHAGES; COLONY-STIMULATING FACTOR; TISSUE-RESIDENT

MACROPHAGES; TRANSCRIPTOME ANALYSIS REVEALS; REGULATORY T-CELLS;

SINGLE-CELL; FACTOR-I; THERAPEUTIC RESISTANCE; CIRCULATING MONOCYTES;

PROMOTES PROGRESSION

AB IntroductionMacrophages are innate immune cells that are associated with extensive phenotypic and functional plasticity and contribute to normal development, tissue homeostasis, and diseases such as cancer. In this review, we discuss the heterogeneity of tissue resident macrophages in the normal mammary gland and tumor-associated macrophages in breast cancer. Tissue resident macrophages are required for mammary gland development, where they have been implicated in promoting extracellular matrix remodeling, apoptotic clearance, and cellular crosstalk. In the context of cancer, tumor-associated macrophages are key drivers of growth and metastasis via their ability to promote matrix remodeling, angiogenesis, lymphangiogenesis, and immunosuppression.MethodWe identified and summarized studies in Pubmed that describe the phenotypic and functional heterogeneity of macrophages and the implications of targeting individual subsets, specifically in the context of mammary gland development and breast cancer. We also identified and summarized recent studies using single-cell RNA sequencing to identify and describe macrophage subsets in human breast cancer samples.ResultsAdvances in single-cell RNA sequencing technologies have yielded nuances in macrophage heterogeneity, with numerous macrophage subsets identified in both the normal mammary gland and breast cancer tissue. Macrophage subsets contribute to mammary gland development and breast cancer progression in differing ways, and emerging studies highlight a role for spatial localization in modulating their phenotype and function.ConclusionUnderstanding macrophage heterogeneity and the unique functions of each subset in both normal mammary gland development and breast cancer progression may lead to more promising targets for the treatment of breast cancer.

C1 [Elfstrum, Alexis K.] Univ Minnesota, Microbiol Immunol & Canc Biol Grad Program, Minneapolis, MN 55455 USA.

[Bapat, Aditi S.] Univ Minnesota, Mol Pharmacol & Therapeut Grad Program, Minneapolis, MN 55455 USA.

[Schwertfeger, Kathryn L.] Univ Minnesota, Dept Lab Med & Pathol, 2231 6 St SE, Minneapolis, MN 55455 USA.

[Schwertfeger, Kathryn L.] Univ Minnesota, Masonic Canc Ctr, Minneapolis, MN 55455 USA.

[Schwertfeger, Kathryn L.] Univ Minnesota, Ctr Immunol, Minneapolis, MN 55455 USA.

C3 University of Minnesota System; University of Minnesota Twin Cities;

University of Minnesota System; University of Minnesota Twin Cities;

University of Minnesota System; University of Minnesota Twin Cities;

University of Minnesota System; University of Minnesota Twin Cities;

University of Minnesota System; University of Minnesota Twin Cities

RP Schwertfeger, KL (corresponding author), Univ Minnesota, Dept Lab Med & Pathol, 2231 6 St SE, Minneapolis, MN 55455 USA.

EM schwe251@umn.edu

OI Elfstrum, Alexis/0000-0003-2908-3527

FU National Institute of Child Health and Human Development

FX No Statement Availabler No Statement Available

CR Amanzada A, 2013, INT J CLIN EXP PATHO, V6, P561

An GL, 2019, ONCOL REP, V42, P2499, DOI 10.3892/or.2019.7344

Anstee JE, 2023, DEV CELL, V58, P1548, DOI 10.1016/j.devcel.2023.06.006

Azizi E, 2018, CELL, V174, P1293, DOI 10.1016/j.cell.2018.05.060

Bain CC, 2014, NAT IMMUNOL, V15, P929, DOI 10.1038/ni.2967

Bieniasz-Krzywiec P, 2019, CELL METAB, V30, P917, DOI 10.1016/j.cmet.2019.07.015

Binnewies M, 2021, CELL REP, V37, DOI 10.1016/j.celrep.2021.109844

Bonapace L, 2014, NATURE, V515, DOI 10.1038/nature13862

Boulakirba S, 2018, SCI REP-UK, V8, DOI 10.1038/s41598-017-18433-4

Brady NJ, 2017, DEV BIOL, V428, P232, DOI 10.1016/j.ydbio.2017.06.007

Bugatti M, 2022, CANCER IMMUNOL RES, V10, P1340, DOI 10.1158/2326-6066.CIR-22-0271

Burks J, 2015, ONCOTARGET, V6, P7221, DOI 10.18632/oncotarget.3372

Campbell MJ, 2011, BREAST CANCER RES TR, V128, P703, DOI 10.1007/s10549-010-1154-y

Cansever D, 2023, NAT IMMUNOL, V24, P1098, DOI 10.1038/s41590-023-01530-0

Canton M, 2021, FRONT IMMUNOL, V12, DOI 10.3389/fimmu.2021.734229

Cendrowicz E, 2021, CANCERS, V13, DOI 10.3390/cancers13081946

Chakarov S, 2019, SCIENCE, V363, P1190, DOI 10.1126/science.aau0964

Chamberlin T, 2020, CANCER RES, V80, P4465, DOI 10.1158/0008-5472.CAN-20-0789

Chen JQ, 2011, CANCER CELL, V19, P541, DOI 10.1016/j.ccr.2011.02.006

Chen YB, 2019, J BIOMED SCI, V26, DOI 10.1186/s12929-019-0568-z

Cheng SJ, 2021, CELL, V184, P792, DOI 10.1016/j.cell.2021.01.010

Chitu V, 2006, CURR OPIN IMMUNOL, V18, P39, DOI 10.1016/j.coi.2005.11.006

Chua ACL, 2010, DEVELOPMENT, V137, P4229, DOI 10.1242/dev.059261

Chung W, 2017, NAT COMMUN, V8, DOI 10.1038/ncomms15081

Clancy RM, 2019, J IMMUNOL, V202, P48, DOI 10.4049/jimmunol.1800357

Comi M, 2020, CELL MOL IMMUNOL, V17, P95, DOI 10.1038/s41423-019-0218-0

Davies LC, 2013, NAT IMMUNOL, V14, P986, DOI 10.1038/ni.2705

Dawson CA, 2020, NAT CELL BIOL, V22, P546, DOI 10.1038/s41556-020-0505-0

Deckers J, 2018, FRONT IMMUNOL, V9, DOI 10.3389/fimmu.2018.00093

Deligne C, 2021, FRONT ONCOL, V11, DOI 10.3389/fonc.2021.620773

DeNardo DG, 2011, CANCER DISCOV, V1, P54, DOI 10.1158/2159-8274.CD-10-0028

Dick SA, 2022, SCI IMMUNOL, V7, DOI 10.1126/sciimmunol.abf7777

DRANOFF G, 1994, SCIENCE, V264, P713, DOI 10.1126/science.8171324

Dutta P, 2018, BREAST CANCER RES TR, V170, P477, DOI 10.1007/s10549-018-4760-8

Elder AM, 2020, J MAMMARY GLAND BIOL, V25, P103, DOI 10.1007/s10911-020-09451-6

Elder AM, 2018, CANCER RES, V78, P6473, DOI 10.1158/0008-5472.CAN-18-1642

Elnakat H, 2004, ADV DRUG DELIVER REV, V56, P1067, DOI 10.1016/j.addr.2004.01.001

Epelman S, 2014, IMMUNITY, V41, P21, DOI 10.1016/j.immuni.2014.06.013

Faget J, 2011, CANCER RES, V71, P6143, DOI 10.1158/0008-5472.CAN-11-0573

Fuady JH, 2014, HYPOXIA, V2, P23, DOI 10.2147/HP.S54404

Ginhoux F, 2016, IMMUNITY, V44, P439, DOI 10.1016/j.immuni.2016.02.024

Gobert M, 2009, CANCER RES, V69, P2000, DOI 10.1158/0008-5472.CAN-08-2360

Gosselin D, 2014, CELL, V159, P1327, DOI 10.1016/j.cell.2014.11.023

Gouon-Evans V, 2002, BREAST CANCER RES, V4, P155, DOI 10.1186/bcr441

Gouon-Evans V, 2000, DEVELOPMENT, V127, P2269

Gunderson AJ, 2016, CANCER DISCOV, V6, P270, DOI 10.1158/2159-8290.CD-15-0827

Gwak JM, 2015, PLOS ONE, V10, DOI 10.1371/journal.pone.0125728

Hara Y, 2018, CANCER SCI, V109, P3171, DOI 10.1111/cas.13755

Hashimoto D, 2013, IMMUNITY, V38, P792, DOI 10.1016/j.immuni.2013.04.004

Hoeffel G, 2015, FRONT IMMUNOL, V6, DOI 10.3389/fimmu.2015.00486

Huang XQ, 2022, THORAC CANCER, V13, P269, DOI 10.1111/1759-7714.14268

Huggins DN, 2021, CANCER RES, V81, P5284, DOI 10.1158/0008-5472.CAN-21-0101

Hynes NE, 2010, CSH PERSPECT BIOL, V2, DOI 10.1101/cshperspect.a003186

Ibrahim AM, 2020, FRONT ONCOL, V10, DOI 10.3389/fonc.2020.569985

Ingman WV, 2006, DEV DYNAM, V235, P3222, DOI 10.1002/dvdy.20972

Irey EA, 2019, P NATL ACAD SCI USA, V116, P12442, DOI 10.1073/pnas.1816410116

Jaitin DA, 2019, CELL, V178, P686, DOI 10.1016/j.cell.2019.05.054

Jäppinen N, 2019, NAT COMMUN, V10, DOI 10.1038/s41467-018-08065-1

Jay TR, 2017, MOL NEURODEGENER, V12, DOI 10.1186/s13024-017-0197-5

Jenkins SJ, 2021, EUR J IMMUNOL, V51, P1882, DOI 10.1002/eji.202048881

Jesser EA, 2021, BREAST CANCER RES, V23, DOI 10.1186/s13058-021-01481-0

Jinushi M, 2011, P NATL ACAD SCI USA, V108, P12425, DOI 10.1073/pnas.1106645108

Jones CV, 2013, ORGANOGENESIS, V9, P249, DOI 10.4161/org.25676

Katzenelenbogen Y, 2020, CELL, V182, P872, DOI 10.1016/j.cell.2020.06.032

Kim K, 2022, CURR OPIN LIPIDOL, V33, P283, DOI 10.1097/MOL.0000000000000842

Kim K, 2018, CIRC RES, V123, P1127, DOI 10.1161/CIRCRESAHA.118.312804

Kim M, 2021, SCI REP-UK, V11, DOI 10.1038/s41598-021-97390-5

Klichinsky M, 2020, NAT BIOTECHNOL, V38, P947, DOI 10.1038/s41587-020-0462-y

Kohli K, 2022, CANCER GENE THER, V29, P10, DOI 10.1038/s41417-021-00303-x

Komohara Y, 2023, CANCER SCI, V114, P2220, DOI 10.1111/cas.15751

Kong LL, 2017, MOL CLIN ONCOL, V7, P515, DOI 10.3892/mco.2017.1356

Koru-Sengul T, 2016, BREAST CANCER RES TR, V158, P113, DOI 10.1007/s10549-016-3847-3

Kotwal GJ, 2017, RESULTS PROBL CELL D, V62, P353, DOI 10.1007/978-3-319-54090-0_14

Krzyszczyk P, 2018, FRONT PHYSIOL, V9, DOI 10.3389/fphys.2018.00419

Kumar T, 2023, NATURE, V620, P181, DOI 10.1038/s41586-023-06252-9

Kumari N, 2022, J EXP CLIN CANC RES, V41, DOI 10.1186/s13046-022-02272-x

Kuwada K, 2018, J EXP CLIN CANC RES, V37, DOI 10.1186/s13046-018-0981-2

Kzhyshkowska J, 2006, J CELL MOL MED, V10, P635, DOI 10.1111/j.1582-4934.2006.tb00425.x

Lavin Y, 2014, CELL, V159, P1312, DOI 10.1016/j.cell.2014.11.018

Leek RD, 2002, J MAMMARY GLAND BIOL, V7, P177, DOI 10.1023/A:1020304003704

Li HZ, 2015, ONCOTARGET, V6, P29637, DOI 10.18632/oncotarget.4936

Lin EY, 2006, CANCER RES, V66, P11238, DOI 10.1158/0008-5472.CAN-06-1278

Lin EY, 2001, J EXP MED, V193, P727, DOI 10.1084/jem.193.6.727

Linde N, 2018, NAT COMMUN, V9, DOI 10.1038/s41467-017-02481-5

Liu Z, 2022, ONCOIMMUNOLOGY, V11, DOI 10.1080/2162402X.2022.2085432

Lu X, 2009, J BIOL CHEM, V284, P29087, DOI 10.1074/jbc.M109.035899

Luo Q, 2020, CANCER SCI, V111, P4000, DOI 10.1111/cas.14616

Ma RY, 2022, TRENDS IMMUNOL, V43, P546, DOI 10.1016/j.it.2022.04.008

Macias H, 2012, WIRES DEV BIOL, V1, P533, DOI 10.1002/wdev.35

Mahmoud SMA, 2012, J CLIN PATHOL, V65, P159, DOI 10.1136/jclinpath-2011-200355

Maniecki MB, 2006, IMMUNOBIOLOGY, V211, P407, DOI 10.1016/j.imbio.2006.05.019

Mantovani A, 2022, NAT REV DRUG DISCOV, V21, P799, DOI 10.1038/s41573-022-00520-5

Mazzieri R, 2011, CANCER CELL, V19, P512, DOI 10.1016/j.ccr.2011.02.005

Medrek C, 2012, BMC CANCER, V12, DOI 10.1186/1471-2407-12-306

Mehta AK, 2021, FRONT IMMUNOL, V12, DOI 10.3389/fimmu.2021.643771

Mills CD, 2000, J IMMUNOL, V164, P6166, DOI 10.4049/jimmunol.164.12.6166

Minutti CM, 2017, SEMIN CELL DEV BIOL, V61, P3, DOI 10.1016/j.semcdb.2016.08.006

Miyasato Y, 2017, CANCER SCI, V108, P1693, DOI 10.1111/cas.13287

Molgora M, 2020, CELL, V182, P886, DOI 10.1016/j.cell.2020.07.013

Mukhtar RA, 2012, ANN SURG ONCOL, V19, P3979, DOI 10.1245/s10434-012-2415-2

Mulder K, 2021, IMMUNITY, V54, P1883, DOI 10.1016/j.immuni.2021.07.007

Mutka M, 2022, BREAST CANCER RES TR, V195, P237, DOI 10.1007/s10549-022-06683-4

Nakashima-Matsushita N, 1999, ARTHRITIS RHEUM-US, V42, P1609, DOI 10.1002/1529-0131(199908)42:8<1609::AID-ANR7>3.0.CO;2-L

Nguyen-Lefebvre AT, 2015, J Enzymol Metab, V1

Nielsen SR, 2017, MEDIAT INFLAMM, V2017, DOI 10.1155/2017/9624760

Niida S, 1999, J EXP MED, V190, P293, DOI 10.1084/jem.190.2.293

Noy R, 2014, IMMUNITY, V41, P49, DOI 10.1016/j.immuni.2014.06.010

O'Brien J, 2012, DEVELOPMENT, V139, P269, DOI 10.1242/dev.071696

O'Shannessy DJ, 2015, J OVARIAN RES, V8, DOI 10.1186/s13048-015-0156-0

Okabe Y, 2014, CELL, V157, P832, DOI 10.1016/j.cell.2014.04.016

Onkar S, 2023, NAT CANCER, V4, P516, DOI 10.1038/s43018-023-00527-w

Opzoomer JW, 2021, SCI ADV, V7, DOI 10.1126/sciadv.abg9518

Painter MM, 2015, MOL NEURODEGENER, V10, DOI 10.1186/s13024-015-0040-9

Pal B, 2021, EMBO J, V40, DOI 10.15252/embj.2020107333

Palazón A, 2012, CLIN CANCER RES, V18, P1207, DOI 10.1158/1078-0432.CCR-11-1591

Park MD, 2022, CELL, V185, P4259, DOI 10.1016/j.cell.2022.10.007

Park SY, 2009, J CELL SCI, V122, P3365, DOI 10.1242/jcs.049569

Pathria P, 2019, TRENDS IMMUNOL, V40, P310, DOI 10.1016/j.it.2019.02.003

Peng QS, 2010, SCI SIGNAL, V3, DOI 10.1126/scisignal.2000500

Perdiguero EG, 2015, IMMUNITY, V43, P1023, DOI 10.1016/j.immuni.2015.11.022

Perou CM, 2000, NATURE, V406, P747, DOI 10.1038/35021093

Peyraud F, 2017, CURR ONCOL REP, V19, DOI 10.1007/s11912-017-0634-1

Pionyr Immunotherapeutics Inc, 2023, A phase 1a/1b openlabel study to evaluate the safety, tolerability, pharmacokinetics, and pharmacodynamics of PY314 as a single agent and in combination with pembrolizumab in subjects with advanced solid tumors

Pollard JW, 2004, NAT REV CANCER, V4, P71, DOI 10.1038/nrc1256

POLLARD JW, 1994, P NATL ACAD SCI USA, V91, P9312, DOI 10.1073/pnas.91.20.9312

Qian BZ, 2010, CELL, V141, P39, DOI 10.1016/j.cell.2010.03.014

Qiu SQ, 2018, CANCER TREAT REV, V70, P178, DOI 10.1016/j.ctrv.2018.08.010

Ramos RN, 2022, CELL, V185, P1189, DOI 10.1016/j.cell.2022.02.021

Reed JR, 2010, J MAMMARY GLAND BIOL, V15, P329, DOI 10.1007/s10911-010-9188-7

Riabov V, 2016, ONCOTARGET, V7, P31097, DOI 10.18632/oncotarget.8857

Riese DJ, 2014, SEMIN CELL DEV BIOL, V28, P49, DOI 10.1016/j.semcdb.2014.03.005

Roy AG, 2021, INT J MOL SCI, V22, DOI 10.3390/ijms22115572

Ruffell B, 2015, CANCER CELL, V27, P462, DOI 10.1016/j.ccell.2015.02.015

Ruffell B, 2012, P NATL ACAD SCI USA, V109, P2796, DOI 10.1073/pnas.1104303108

Russell DG, 2009, NAT IMMUNOL, V10, P943, DOI 10.1038/ni.1781

Sanchez LR, 2019, J LEUKOCYTE BIOL, V106, P259, DOI 10.1002/JLB.MR0218-056RR

Sathe A, 2020, CLIN CANCER RES, V26, P2640, DOI 10.1158/1078-0432.CCR-19-3231

Savage NDL, 2008, J IMMUNOL, V181, P2220, DOI 10.4049/jimmunol.181.3.2220

Schledzewski K, 2006, J PATHOL, V209, P67, DOI 10.1002/path.1942

Schneider C, 2014, NAT IMMUNOL, V15, P1026, DOI 10.1038/ni.3005

Schwertfeger KL, 2006, J MAMMARY GLAND BIOL, V11, P229, DOI 10.1007/s10911-006-9028-y

Sheng JP, 2015, IMMUNITY, V43, P382, DOI 10.1016/j.immuni.2015.07.016

Shin SA, 2019, ARCH PHARM RES, V42, P658, DOI 10.1007/s12272-019-01169-2

Shree T, 2011, GENE DEV, V25, P2465, DOI 10.1101/gad.180331.111

Siveen KS, 2009, IMMUNOL LETT, V123, P97, DOI 10.1016/j.imlet.2009.02.011

STANLEY E, 1994, P NATL ACAD SCI USA, V91, P5592, DOI 10.1073/pnas.91.12.5592

Sternlicht MD, 2006, BREAST CANCER RES, V8, DOI 10.1186/bcr1368

Stewart TA, 2019, FRONT CELL DEV BIOL, V7, DOI 10.3389/fcell.2019.00250

Sun X, 2014, J MAMMARY GLAND BIOL, V19, P191, DOI 10.1007/s10911-014-9319-7

Takahashi K, 2005, J EXP MED, V201, P647, DOI 10.1084/jem.20041611

Timperi E, 2022, CANCER RES, V82, P3291, DOI 10.1158/0008-5472.CAN-22-1427

Tkach M, 2022, P NATL ACAD SCI USA, V119, DOI 10.1073/pnas.2107394119

Ueno T, 2000, CLIN CANCER RES, V6, P3282

Vasiljeva O, 2006, CANCER RES, V66, P5242, DOI 10.1158/0008-5472.CAN-05-4463

Wang XL, 2021, FRONT CELL NEUROSCI, V15, DOI 10.3389/fncel.2021.722028

Wang Y, 2020, ELIFE, V9, DOI 10.7554/eLife.57438

Whitsett JA, 2010, ANNU REV MED, V61, P105, DOI 10.1146/annurev.med.60.041807.123500

Wilson GJ, 2022, IMMUNOLOGY, V165, P206, DOI 10.1111/imm.13430

Wilson GJ, 2020, DEVELOPMENT, V147, DOI 10.1242/dev.187815

Witschen PM, 2023, J MAMMARY GLAND BIOL, V28, DOI 10.1007/s10911-023-09528-y

Wu SZ, 2021, NAT GENET, V53, P1334, DOI 10.1038/s41588-021-00911-1

Wyckoff J, 2004, CANCER RES, V64, P7022, DOI 10.1158/0008-5472.CAN-04-1449

Xia ZA, 2022, CANCERS, V14, DOI 10.3390/cancers14225506

Xu LY, 2022, ANTI-CANCER AGENT ME, V22, P294, DOI 10.2174/1871520621666210618100857

Yan D, 2017, ONCOGENE, V36, P6049, DOI 10.1038/onc.2017.261

Yang W, 2023, BREAST CANCER RES, V25, DOI 10.1186/s13058-023-01703-7

Yersal O, 2014, WORLD J CLIN ONCOL, V5, P412, DOI 10.5306/wjco.v5.i3.412

Yona S, 2013, IMMUNITY, V38, P79, DOI 10.1016/j.immuni.2012.12.001

Yonemitsu K, 2022, SCI REP-UK, V12, DOI 10.1038/s41598-022-16080-y

York MR, 2007, ARTHRITIS RHEUM-US, V56, P1010, DOI 10.1002/art.22382

Yuan ZY, 2014, ONCOTARGETS THER, V7, P1, DOI 10.2147/OTT.S61838

Zhang L, 2020, CELL, V181, P442, DOI 10.1016/j.cell.2020.03.048

Zhang WJ, 2018, J SURG RES, V222, P93, DOI 10.1016/j.jss.2017.09.035

Zheng QL, 2015, CELL RES, V25, P1121, DOI 10.1038/cr.2015.108

Zhong L, 2017, J EXP MED, V214, P597, DOI 10.1084/jem.20160844

NR 175

TC 4

Z9 4

U1 4

U2 10

PU WILEY

PI HOBOKEN

PA 111 RIVER ST, HOBOKEN 07030-5774, NJ USA

SN 2045-7634

J9 CANCER MED-US

JI Cancer Med.

PD FEB

PY 2024

VL 13

IS 3

AR e7053

DI 10.1002/cam4.7053

PG 19

WC Oncology

WE Science Citation Index Expanded (SCI-EXPANDED)

SC Oncology

GA JV2U0

UT WOS:001175879800001

PM 38426622

OA Green Published

DA 2025-02-07

ER

PT J

AU Zhu, Y

Herndon, JM

Sojka, DK

Kim, KW

Knolhoff, BL

Zuo, C

Cullinan, DR

Luo, JQ

Bearden, AR

Lavine, KJ

Yokoyama, WM

Hawkins, WG

Fields, RC

Randolph, GJ

DeNardo, DG

AF Zhu, Yu

Herndon, John M.

Sojka, Dorothy K.

Kim, Ki-Wook

Knolhoff, Brett L.

Zuo, Chong

Cullinan, Darren R.

Luo, Jingqin

Bearden, Audrey R.

Lavine, Kory J.

Yokoyama, Wayne M.

Hawkins, William G.

Fields, Ryan C.

Randolph, Gwendalyn J.

DeNardo, David G.

TI Tissue-Resident Macrophages in Pancreatic Ductal Adenocarcinoma

Originate from Embryonic Hematopoiesis and Promote Tumor Progression

SO IMMUNITY

LA English

DT Article

ID BONE-MARROW; CHECKPOINT IMMUNOTHERAPY; INFILTRATING MACROPHAGES;

FUNCTIONALLY DISTINCT; CARDIAC MACROPHAGES; T-CELLS; MONOCYTES; CANCER;

MICE; INFLAMMATION

AB Tumor-associated macrophages (TAMs) are essential components of the cancer microenvironment and play critical roles in the regulation of tumor progression. Optimal therapeutic intervention requires in-depth understanding of the sources that sustain macrophages in malignant tissues. In this study, we investigated the ontogeny of TAMs in murine pancreatic ductal adenocarcinoma (PDAC) models. We identified both inflammatory monocytes and tissue-resident macrophages as sources of TAMs. Unexpectedly, significant portions of pancreas-resident macrophages originated from embryonic development and expanded through in situ proliferation during tumor progression. Whereas monocyte-derived TAMs played more potent roles in antigen presentation, embryonically derived TAMs exhibited a profibrotic transcriptional profile, indicative of their role in producing and remodeling molecules in the extracellular matrix. Collectively, these findings uncover the heterogeneity of TAM origin and functions and could provide therapeutic insight for PDAC-treatment.

C1 [Zhu, Yu; Herndon, John M.; Sojka, Dorothy K.; Kim, Ki-Wook; Knolhoff, Brett L.; Zuo, Chong; Cullinan, Darren R.; Luo, Jingqin; Bearden, Audrey R.; Lavine, Kory J.; Hawkins, William G.; Fields, Ryan C.; Randolph, Gwendalyn J.; DeNardo, David G.] Washington Univ St Louis, Sch Med, St Louis, MO 63110 USA.

C3 Washington University (WUSTL)

RP DeNardo, DG (corresponding author), Washington Univ St Louis, Sch Med, St Louis, MO 63110 USA.

EM ddenardo@wustl.edu

RI Lavine, Kory/ABP-2301-2022; Zuo, Chong/KIL-6592-2024; Randolph,

Gwendalyn/AES-5195-2022

OI Herndon, John/0000-0002-8378-9654; /0000-0002-7207-4389; Zuo,

Chong/0000-0001-6117-6438; DeNardo, David/0000-0002-3655-5783; Zhu,

Yu/0000-0002-3250-6546; luo, jingqin/0000-0003-2759-3072

FU American Association for Cancer Research and Pancreatic Cancer Action

Network; National Cancer Institute (NCI) [R01-CA177670, R01-CA203890,

P50-CA196510, T32CA009621, UL1TR000448, P30-CA91842]; BJC Institute of

Health and Siteman Cancer Center Cancer Frontier Fund; NCI [P30-CA91842

NCRR UL1RR024992]

FX The authors acknowledge support from an award from the American

Association for Cancer Research and Pancreatic Cancer Action Network,

National Cancer Institute (NCI) awards R01-CA177670, R01-CA203890,

P50-CA196510, T32CA009621, UL1TR000448, and P30-CA91842, and the BJC

Institute of Health and Siteman Cancer Center Cancer Frontier Fund.

Microarray analyses were performed by the Genome Technology Access

Center at Washington University and were partially funded by NCI award

P30-CA91842 NCRR UL1RR024992. The authors also acknowledge Grant Gould

and Hans Challen for help on irradiation experiments, Liping Yang for

help on parabiosis, and Daniel C. Link, Jason C. Mills, Boris Calderon,

and Jesse W. Williams for advice.

CR Afik R, 2016, J EXP MED, V213, P2315, DOI 10.1084/jem.20151193

Bain CC, 2016, NAT COMMUN, V7, DOI 10.1038/ncomms11852

Bain CC, 2014, NAT IMMUNOL, V15, P929, DOI 10.1038/ni.2967

Beatty GL, 2015, GASTROENTEROLOGY, V149, P201, DOI 10.1053/j.gastro.2015.04.010

Bleriot C, 2015, IMMUNITY, V42, P145, DOI 10.1016/j.immuni.2014.12.020

Boring L, 1997, J CLIN INVEST, V100, P2552, DOI 10.1172/JCI119798

Bowman RL, 2016, CELL REP, V17, P2445, DOI 10.1016/j.celrep.2016.10.052

Calderon B, 2015, J EXP MED, V212, P1497, DOI 10.1084/jem.20150496

Epelman S, 2014, IMMUNITY, V40, P91, DOI 10.1016/j.immuni.2013.11.019

Franklin RA, 2014, SCIENCE, V344, P921, DOI 10.1126/science.1252510

Gibbings SL, 2015, BLOOD, V126, P1357, DOI 10.1182/blood-2015-01-624809

Ginhoux F, 2016, IMMUNITY, V44, P439, DOI 10.1016/j.immuni.2016.02.024

Ginhoux F, 2010, SCIENCE, V330, P841, DOI 10.1126/science.1194637

Gundra UM, 2014, BLOOD, V123, pE110, DOI 10.1182/blood-2013-08-520619

Hambardzumyan D, 2016, NAT NEUROSCI, V19, P20, DOI 10.1038/nn.4185

Hanna RN, 2011, NAT IMMUNOL, V12, P778, DOI 10.1038/ni.2063

Hingorani SR, 2005, CANCER CELL, V7, P469, DOI 10.1016/j.ccr.2005.04.023

Hoeffel G, 2015, IMMUNITY, V42, P665, DOI 10.1016/j.immuni.2015.03.011

Ino Y, 2013, BRIT J CANCER, V108, P914, DOI 10.1038/bjc.2013.32

Jenkins SJ, 2011, SCIENCE, V332, P1284, DOI 10.1126/science.1204351

Jiang H, 2016, NAT MED, V22, P851, DOI 10.1038/nm.4123

Kim MP, 2009, NAT PROTOC, V4, P1670, DOI 10.1038/nprot.2009.171

Kurahara H, 2011, J SURG RES, V167, pE211, DOI 10.1016/j.jss.2009.05.026

Mitchem JB, 2013, CANCER RES, V73, P1128, DOI 10.1158/0008-5472.CAN-12-2731

Molawi K, 2014, J EXP MED, V211, P2151, DOI 10.1084/jem.20140639

Movahedi K, 2010, CANCER RES, V70, P5728, DOI 10.1158/0008-5472.CAN-09-4672

Peng H, 2013, J CLIN INVEST, V123, P1444, DOI 10.1172/JCI66381

Perdiguero EG, 2015, NATURE, V518, P547, DOI 10.1038/nature13989

Qian BZ, 2011, NATURE, V475, P222, DOI 10.1038/nature10138

Sanford DE, 2013, CLIN CANCER RES, V19, P3404, DOI 10.1158/1078-0432.CCR-13-0525

Schulz C, 2012, SCIENCE, V336, P86, DOI 10.1126/science.1219179

Scott CL, 2016, NAT COMMUN, V7, DOI 10.1038/ncomms10321

Tacke F, 2006, J EXP MED, V203, P583, DOI 10.1084/jem.20052119

van de Laar L, 2016, IMMUNITY, V44, P755, DOI 10.1016/j.immuni.2016.02.017

Wynn TA, 2016, IMMUNITY, V44, P450, DOI 10.1016/j.immuni.2016.02.015

Yona S, 2013, IMMUNITY, V38, P79, DOI 10.1016/j.immuni.2012.12.001

Zhu Y, 2014, CANCER RES, V74, P5057, DOI 10.1158/0008-5472.CAN-13-3723

NR 37

TC 489

Z9 573

U1 7

U2 61

PU CELL PRESS

PI CAMBRIDGE

PA 50 HAMPSHIRE ST, FLOOR 5, CAMBRIDGE, MA 02139 USA

SN 1074-7613

EI 1097-4180

J9 IMMUNITY

JI Immunity

PD AUG 15

PY 2017

VL 47

IS 2

BP 323

EP +

DI 10.1016/j.immuni.2017.07.014

PG 22

WC Immunology

WE Science Citation Index Expanded (SCI-EXPANDED)

SC Immunology

GA FD8QD

UT WOS:000407788300017

PM 28813661

OA Green Accepted

HC Y

HP N

DA 2025-02-07

ER

PT J

AU Friebel, E

Kapolou, K

Unger, S

Núñez, NG

Utz, S

Rushing, EJ

Regli, L

Weller, M

Greter, M

Tugues, S

Neidert, MC

Becher, B

AF Friebel, Ekaterina

Kapolou, Konstantina

Unger, Susanne

Nunez, Nicolas Gonzalo

Utz, Sebastian

Rushing, Elisabeth Jane

Regli, Luca

Weller, Michael

Greter, Melanie

Tugues, Sonia

Neidert, Marian Christoph

Becher, Burkhard

TI Single-Cell Mapping of Human Brain Cancer Reveals Tumor-Specific

Instruction of Tissue-Invading Leukocytes

SO CELL

LA English

DT Article

ID CENTRAL-NERVOUS-SYSTEM; INFILTRATING LYMPHOCYTES; MACROPHAGE ONTOGENY;

MASS CYTOMETRY; IMMUNE; MICROGLIA; GLIOBLASTOMA; VISUALIZATION;

POLARIZATION; EXPRESSION

AB Brain malignancies can either originate from within the CNS (gliomas) or invade from other locations in the body (metastases). A highly immunosuppressive tumor microenvironment (TME) influences brain tumor outgrowth. Whether the TME is predominantly shaped by the CNS micromilieu or by the malignancy itself is unknown, as is the diversity, origin, and function of CNS tumor-associated macrophages (TAMs). Here, we have mapped the leukocyte landscape of brain tumors using high-dimensional single-cell profiling (Cy-TOF). The heterogeneous composition of tissue-resident and invading immune cells within the TME alone permitted a clear distinction between gliomas and brain metastases (BrM). The glioma TME presented predominantly with tissue-resident, reactive microglia, whereas tissue-invading leukocytes accumulated in BrM. Tissue-invading TAMs showed a distinctive signature trajectory, revealing tumor-driven instruction along with contrasting lymphocyte activation and exhaustion. Defining the specific immunological signature of brain tumors can facilitate the rational design of targeted immunotherapy strategies.

C1 [Friebel, Ekaterina; Unger, Susanne; Nunez, Nicolas Gonzalo; Utz, Sebastian; Greter, Melanie; Tugues, Sonia; Becher, Burkhard] Univ Zurich, Inst Expt Immunol, CH-8057 Zurich, Switzerland.

[Kapolou, Konstantina; Weller, Michael; Neidert, Marian Christoph] Univ Hosp Zurich, Clin Neurosci Ctr, Dept Neurol, Lab Mol Neurooncol, CH-8091 Zurich, Switzerland.

[Kapolou, Konstantina; Rushing, Elisabeth Jane; Regli, Luca; Weller, Michael; Neidert, Marian Christoph] Univ Zurich, CH-8091 Zurich, Switzerland.

[Regli, Luca; Neidert, Marian Christoph] Univ Hosp Zurich, Clin Neurosci Ctr, Dept Neurosurg, CH-8091 Zurich, Switzerland.

[Rushing, Elisabeth Jane] Univ Hosp Zurich, Dept Neuropathol, CH-8091 Zurich, Switzerland.

C3 University of Zurich; University of Zurich; University Zurich Hospital;

University of Zurich; University of Zurich; University Zurich Hospital;

University of Zurich; University Zurich Hospital

RP Becher, B (corresponding author), Univ Zurich, Inst Expt Immunol, CH-8057 Zurich, Switzerland.

EM becher@immunology.uzh.ch

RI Utz, Sebastian/E-4885-2018; Weber, Michael/L-9836-2016; Friebel,

Ekaterina/ABF-1308-2021; Becher, Burkhard/ABE-4225-2020

OI Neidert, Marian C./0000-0003-2828-4706; Weller,

Michael/0000-0002-1748-174X; Unger, Susanne/0000-0001-6916-7544; Nunez,

Nicolas/0000-0003-3837-270X; Friebel, Ekaterina/0000-0003-1419-2376;

Becher, Burkhard/0000-0002-1541-7867; Greter,

Melanie/0000-0002-7220-5369

FU Swiss Cancer League [KFS-4431-02-2018]; Swiss National Science

Foundation [733310030_170320, 310030_188450, CRSII5_183478]; European

Union [826121]; Clinical Research Priority Program ImmunoCure;

University Research Priority Program Translational Cancer Research of

the University of Zurich; Swiss National Science Foundation (SNF)

[CRSII5_183478, 310030_188450] Funding Source: Swiss National Science

Foundation (SNF)

FX Sall1CreER mice were kindly provided by R. Nishinakamura (Kumamoto

University). We thank the Mass-and Flow Cytometry Facility (University

of Zurich) for technical assistance, the Department of Neuropathology

(University Hospital Zurich and University of Zurich) for performing

immunohistochemistry staining, and Helen Pickersgill of Life Science

Editors for critical review and editing of the manuscript. This work was

supported by grants from the Swiss Cancer League (KFS-4431-02-2018), the

Swiss National Science Foundation (733310030_170320, 310030_188450, and

CRSII5_183478) to B.B., the European Union H2020 Project iPC (826121 to

B.B.), the Clinical Research Priority Program ImmunoCure (to B.B., M.W.,

and M.C.N.), and the University Research Priority Program Translational

Cancer Research of the University of Zurich (to B.B. and M.W.).

CR Aldape K, 2019, NAT REV CLIN ONCOL, V16, P509, DOI 10.1038/s41571-019-0177-5

Amankulor NM, 2017, GENE DEV, V31, P774, DOI 10.1101/gad.294991.116

Amit M, 2013, CANCER MED-US, V2, P155, DOI 10.1002/cam4.45

An J, 2017, MICRO NANO TECHNOL, P1, DOI 10.1016/B978-0-323-41481-4.00001-0

Bendall SC, 2011, SCIENCE, V332, P687, DOI 10.1126/science.1198704

Berghoff AS, 2017, NEURO-ONCOLOGY, V19, P1460, DOI 10.1093/neuonc/nox054

Berghoff AS, 2015, NEURO-ONCOLOGY, V17, P1064, DOI 10.1093/neuonc/nou307

Bienkowski M, 2015, CURR OPIN NEUROL, V28, P647, DOI 10.1097/WCO.0000000000000251

Binnewies M, 2018, NAT MED, V24, P541, DOI 10.1038/s41591-018-0014-x

Bouffet E, 2016, J CLIN ONCOL, V34, P2206, DOI 10.1200/JCO.2016.66.6552

Bowman RL, 2016, CELL REP, V17, P2445, DOI 10.1016/j.celrep.2016.10.052

Buja A, 2008, J COMPUT GRAPH STAT, V17, P444, DOI 10.1198/106186008X318440

Bunse L, 2018, NAT MED, V24, P1192, DOI 10.1038/s41591-018-0095-6

Butovsky O, 2014, NAT NEUROSCI, V17, P131, DOI 10.1038/nn.3599

Buttgereit A, 2016, NAT IMMUNOL, V17, P1397, DOI 10.1038/ni.3585

Chen ZH, 2017, CANCER RES, V77, P2266, DOI 10.1158/0008-5472.CAN-16-2310

Cheng Y, 2016, J IMMUNOL, V196, P924, DOI 10.4049/jimmunol.1501928

Colaprico A, 2016, NUCLEIC ACIDS RES, V44, DOI 10.1093/nar/gkv1507

Coniglio SJ, 2012, MOL MED, V18, P519, DOI 10.2119/molmed.2011.00217

Croxford AL, 2015, IMMUNITY, V43, P502, DOI 10.1016/j.immuni.2015.08.010

Daniel P, 2019, FRONT ONCOL, V9, DOI 10.3389/fonc.2019.00041

de Boer J, 2003, EUR J IMMUNOL, V33, P314, DOI 10.1002/immu.200310005

Eckel-Passow JE, 2015, NEW ENGL J MED, V372, P2499, DOI 10.1056/NEJMoa1407279

Eisenring M, 2010, NAT IMMUNOL, V11, P1030, DOI 10.1038/ni.1947

Ellis B., 2019, FLOWCORE BASIC STRUC

Field A., 2013, FLOWWORKSPACEDATA DA

Field A. P., 2018, Discovering statistics using SPSS, V5th

Finck R, 2013, CYTOM PART A, V83A, P483, DOI 10.1002/cyto.a.22271

Forrester JV, 2018, NAT REV NEUROSCI, V19, P655, DOI 10.1038/s41583-018-0070-8

Ginhoux F, 2016, IMMUNITY, V44, P439, DOI 10.1016/j.immuni.2016.02.024

Ginhoux F, 2010, SCIENCE, V330, P841, DOI 10.1126/science.1194637

Goldberg SB, 2016, LANCET ONCOL, V17, P976, DOI 10.1016/S1470-2045(16)30053-5

Goldmann T, 2016, NAT IMMUNOL, V17, P797, DOI 10.1038/ni.3423

Good Z, 2019, NAT BIOTECHNOL, V37, P259, DOI 10.1038/s41587-019-0033-2

Gu ZG, 2014, BIOINFORMATICS, V30, P2811, DOI 10.1093/bioinformatics/btu393

Hahne F., 2020, FLOWSTATS STAT METHO

Hambardzumyan D, 2016, NAT NEUROSCI, V19, P20, DOI 10.1038/nn.4185

Harrell F.E., 2018, Hmisc: Harrell Miscellaneous

Harter PN, 2015, ONCOTARGET, V6, P40836, DOI 10.18632/oncotarget.5696

Hartmann FJ, 2016, J EXP MED, V213, P2621, DOI 10.1084/jem.20160897

Haynes SE, 2006, NAT NEUROSCI, V9, P1512, DOI 10.1038/nn1805

Hopperton K, 2018, MOL PSYCHIATR, V23, P177, DOI 10.1038/mp.2017.246

Inoue S, 2010, GENESIS, V48, P207, DOI 10.1002/dvg.20603

Jacob F, 2020, CELL, V180, P188, DOI 10.1016/j.cell.2019.11.036

Jacobs JFM, 2010, J NEUROIMMUNOL, V225, P195, DOI 10.1016/j.jneuroim.2010.05.020

Jacomy M, 2009, Proc Int AAAI Conf Web Soc Med, DOI DOI 10.1609/ICWSM.V3I1.13937

Johanns TM, 2016, CANCER DISCOV, V6, P1230, DOI 10.1158/2159-8290.CD-16-0575

Keenan TE, 2019, NAT MED, V25, P389, DOI 10.1038/s41591-019-0382-x

Keren-Shaul H, 2017, CELL, V169, P1276, DOI 10.1016/j.cell.2017.05.018

Kiss M, 2018, CELL IMMUNOL, V330, P188, DOI 10.1016/j.cellimm.2018.02.008

Kluger HM, 2019, J CLIN ONCOL, V37, P52, DOI 10.1200/JCO.18.00204

Kohanbash G, 2017, J CLIN INVEST, V127, P1425, DOI 10.1172/JCI90644

Kolde, 2019, PHEATMAP PRETTY HEAT, V1, P12, DOI DOI 10.32614/CRAN.PACKAGE.PHEATMAP

KUPPNER MC, 1988, INT J CANCER, V42, P562, DOI 10.1002/ijc.2910420416

Louis DN, 2016, ACTA NEUROPATHOL, V131, P803, DOI 10.1007/s00401-016-1545-1

Mansour AA, 2018, NAT BIOTECHNOL, V36, P432, DOI 10.1038/nbt.4127

Mantovani A, 2017, NAT REV CLIN ONCOL, V14, P399, DOI 10.1038/nrclinonc.2016.217

McInnes L, 2020, Arxiv, DOI [arXiv:1802.03426, 10.21105/joss.00861, DOI 10.48550/ARXIV.1802.03426, 10.48550/arXiv.1802.03426]

Mei HE, 2016, CYTOM PART A, V89A, P292, DOI 10.1002/cyto.a.22778

Miyai M, 2017, J NEURO-ONCOL, V135, P423, DOI 10.1007/s11060-017-2626-2

Mrdjen D, 2018, IMMUNITY, V48, P380, DOI 10.1016/j.immuni.2018.01.011

Mrdjen D, 2017, METHODS MOL BIOL, V1559, P321, DOI 10.1007/978-1-4939-6786-5_22

Müller S, 2017, GENOME BIOL, V18, DOI 10.1186/s13059-017-1362-4

Murray PJ, 2014, IMMUNITY, V41, P14, DOI 10.1016/j.immuni.2014.06.008

Noble WS, 2009, NAT BIOTECHNOL, V27, P1135, DOI 10.1038/nbt1209-1135

Nowicka Malgorzata, 2017, F1000Res, V6, P748, DOI 10.12688/f1000research.11622.1

Okabe Y, 2016, NAT IMMUNOL, V17, P9, DOI 10.1038/ni.3320

Pyonteck SM, 2013, NAT MED, V19, P1264, DOI 10.1038/nm.3337

Quail DF, 2017, CANCER CELL, V31, P326, DOI 10.1016/j.ccell.2017.02.009

Quail DF, 2016, SCIENCE, V352, DOI 10.1126/science.aad3018

Quail DF, 2013, NAT MED, V19, P1423, DOI 10.1038/nm.3394

Ries CH, 2014, CANCER CELL, V25, P846, DOI 10.1016/j.ccr.2014.05.016

Samusik N, 2016, NAT METHODS, V13, P493, DOI [10.1038/NMETH.3863, 10.1038/nmeth.3863]

Sankowski R, 2019, NAT NEUROSCI, V22, P2098, DOI 10.1038/s41593-019-0532-y

Schindelin J, 2012, NAT METHODS, V9, P676, DOI [10.1038/NMETH.2019, 10.1038/nmeth.2019]

Schulz D, 2019, SCI REP-UK, V9, DOI 10.1038/s41598-018-38127-9

Shi Y, 2017, NAT COMMUN, V8, DOI 10.1038/ncomms15080

Simoni Y, 2017, IMMUNITY, V46, P148, DOI 10.1016/j.immuni.2016.11.005

Smolders J, 2018, NAT COMMUN, V9, DOI 10.1038/s41467-018-07053-9

Spitzer MH, 2015, SCIENCE, V349, DOI 10.1126/science.1259425

Street Kelly, 2018, BMC Genomics, V19, P477, DOI 10.1186/s12864-018-4772-0

Takasato M, 2004, MECH DEVELOP, V121, P547, DOI 10.1016/j.mod.2004.04.007

Takenaka MC, 2019, NAT NEUROSCI, V22, P729, DOI 10.1038/s41593-019-0370-y

Tawbi HA, 2018, NEW ENGL J MED, V379, P722, DOI 10.1056/NEJMoa1805453

Team R, 2015, RSTUDIO INT DEV R

Team RC, 2020, R: A language and environment for statistical computing

Thorsson V, 2019, IMMUNITY, V51, P411, DOI [10.1016/j.immuni.2019.08.004, 10.1016/j.immuni.2018.03.023]

Tugues S, 2019, SEMIN IMMUNOL, V41, DOI 10.1016/j.smim.2019.03.002

Uhl M, 2004, CANCER RES, V64, P7954, DOI 10.1158/0008-5472.CAN-04-1013

Unruh D, 2019, SCI REP-UK, V9, DOI 10.1038/s41598-019-45346-1

van der Maaten L, 2008, J MACH LEARN RES, V9, P2579

Van Gassen S, 2015, CYTOM PART A, V87A, P636, DOI 10.1002/cyto.a.22625

Van Hove H, 2019, NAT NEUROSCI, V22, P1021, DOI 10.1038/s41593-019-0393-4

Venteicher AS, 2017, SCIENCE, V355, DOI 10.1126/science.aai8478

Walker DG, 2015, ALZHEIMERS RES THER, V7, DOI 10.1186/s13195-015-0139-9

Warnes G. R., 2019, GPLOTS VARIOUS R PRO

Wickham H, 2021, GGPLOT2 CREATE ELEGA, DOI DOI 10.1007/978-3-319-24277-4

Wickham H, 2018, dplyr: A Grammar of Data Manipulation

Woroniecka Karolina, 2018, Oncotarget, V9, P35287, DOI 10.18632/oncotarget.26228

Xue J, 2014, IMMUNITY, V40, P274, DOI 10.1016/j.immuni.2014.01.006

Zhou WC, 2015, NAT CELL BIOL, V17, P170, DOI 10.1038/ncb3090

Zunder ER, 2015, NAT PROTOC, V10, P316, DOI 10.1038/nprot.2015.020

NR 102

TC 397

Z9 416

U1 15

U2 130

PU CELL PRESS

PI CAMBRIDGE

PA 50 HAMPSHIRE ST, FLOOR 5, CAMBRIDGE, MA 02139 USA

SN 0092-8674

EI 1097-4172

J9 CELL

JI Cell

PD JUN 25

PY 2020

VL 181

IS 7

BP 1626

EP +

DI 10.1016/j.cell.2020.04.055

PG 37

WC Biochemistry & Molecular Biology; Cell Biology

WE Science Citation Index Expanded (SCI-EXPANDED)

SC Biochemistry & Molecular Biology; Cell Biology

GA MD2SA

UT WOS:000543822100018

PM 32470397

OA Bronze

HC Y

HP N

DA 2025-02-07

ER

PT J

AU Aegerter, H

Lambrecht, BN

Jakubzick, C

AF Aegerter, Helena

Lambrecht, Bart N.

Jakubzick, Claudia, V

TI Biology of lung macrophages in health and disease

SO IMMUNITY

LA English

DT Review

ID TISSUE-RESIDENT MACROPHAGES; FLOW-CYTOMETRIC ANALYSIS;

COLONY-STIMULATING FACTOR; ALVEOLAR MACROPHAGES; INTERSTITIAL

MACROPHAGES; MONONUCLEAR PHAGOCYTES; FETAL MONOCYTES; IMMUNE-RESPONSE;

DENDRITIC CELL; MYELOID CELLS

AB Tissue-resident alveolar and interstitial macrophages and recruited macrophages are critical players in innate immunity and maintenance of lung homeostasis. Until recently, assessing the differential functional contributions of tissue-resident versus recruited macrophages has been challenging because they share overlapping cell surface markers, making it difficult to separate them using conventional methods. This review describes how scRNA-seq and spatial transcriptomics can separate these subpopulations and help unravel the complexity of macrophage biology in homeostasis and disease. First, we provide a guide to identifying and distinguishing lung macrophages from other mononuclear phagocytes in humans and mice. Second, we outline emerging concepts related to the development and function of the various lung macrophages in the alveolar, perivascular, and interstitial niches. Finally, we describe how different tissue states profoundly alter their functions, including acute and chronic lung disease, cancer, and aging.

C1 [Aegerter, Helena; Lambrecht, Bart N.] VIB UGent Ctr Inflammat Res, Lab Immunoregulat & Mucosal Immunol, Ghent, Belgium.

[Aegerter, Helena; Lambrecht, Bart N.] Univ Ghent, Dept Internal Med & Pediat, Ghent, Belgium.

[Lambrecht, Bart N.] ErasmusMC, Dept Pulm Med, Rotterdam, Netherlands.

[Jakubzick, Claudia, V] Dartmouth Geisel Sch Med, Dept Microbiol & Immunol, Hanover, NH 03755 USA.

C3 Flanders Institute for Biotechnology (VIB); Ghent University; Ghent

University; Erasmus University Rotterdam; Erasmus MC; Dartmouth College

RP Jakubzick, C (corresponding author), Dartmouth Geisel Sch Med, Dept Microbiol & Immunol, Hanover, NH 03755 USA.

RI Lambrecht, Bart/K-2484-2014

OI Aegerter, Helena Catharine/0000-0002-5755-9126; Jakubzick,

Claudia/0000-0002-3731-0198

FU FWO Methusalem grant; FWO Excellence of Science grant; European Research

Council (ERC) under the European Union [789384]; FWO-VLAIO Fellowship;

National Institutes of Health [R35 HL155458]; European Research Council

(ERC) [789384] Funding Source: European Research Council (ERC)

FX B.N.L. is supported by a FWO Methusalem grant, and by an FWO Excellence

of Science grant. He has received funding from the European Research

Council (ERC) under the European Union's Horizon 2020 research and

innovation programme (grant agreement No 789384). H.A. is supported by

an FWO-VLAIO Fellowship. C.V.J. is supported by National Institutes of

Health grant R35 HL155458.

CR Aegerter H, 2020, NAT IMMUNOL, V21, P145, DOI 10.1038/s41590-019-0568-x

Angelidis I, 2019, NAT COMMUN, V10, DOI 10.1038/s41467-019-08831-9

Antonelli LRV, 2010, J CLIN INVEST, V120, P1674, DOI 10.1172/JCI40817

Arafa EI, 2022, JCI INSIGHT, V7, DOI 10.1172/jci.insight.150239

Aran D, 2019, NAT IMMUNOL, V20, P163, DOI 10.1038/s41590-018-0276-y

Atif SM, 2018, METHODS MOL BIOL, V1784, P69, DOI 10.1007/978-1-4939-7837-3_6

Bain CC, 2022, MUCOSAL IMMUNOL, V15, P223, DOI 10.1038/s41385-021-00480-w

Baker EH, 2018, CHEST, V153, P507, DOI 10.1016/j.chest.2017.05.031

Balbo P, 2001, CLIN EXP ALLERGY, V31, P625, DOI 10.1046/j.1365-2222.2001.01068.x

Barnes Peter J, 2004, COPD, V1, P59, DOI 10.1081/COPD-120028701

Bedoret D, 2009, J CLIN INVEST, V119, P3723, DOI 10.1172/JCI39717

Belchamber KBR, 2019, EUR RESPIR J, V54, DOI 10.1183/13993003.02244-2018

Bharat A, 2016, AM J RESP CELL MOL, V54, P147, DOI 10.1165/rcmb.2015-0147LE

Bhattacharyya A, 2022, AM J PHYSIOL-LUNG C, V322, pL495, DOI 10.1152/ajplung.00458.2021

Bleriot C, 2020, IMMUNITY, V52, P957, DOI 10.1016/j.immuni.2020.05.014

BLUSSEVANOUDALBLAS A, 1979, J EXP MED, V149, P1504, DOI 10.1084/jem.149.6.1504

Bobba CM, 2021, NAT COMMUN, V12, DOI 10.1038/s41467-020-20449-w

Bosteels C, 2020, TRIALS, V21, DOI 10.1186/s13063-020-04451-7

Bosteels C, 2020, IMMUNITY, V52, P1039, DOI 10.1016/j.immuni.2020.04.005

Braga FAV, 2019, NAT MED, V25, P1153, DOI 10.1038/s41591-019-0468-5

Branchett WJ, 2021, J ALLERGY CLIN IMMUN, V147, P1892, DOI 10.1016/j.jaci.2021.01.026

Busch CJL, 2019, BIO-PROTOCOL, V9, DOI 10.21769/BioProtoc.3302

Byrneee AJ, 2020, J EXP MED, V217, DOI 10.1084/jem.20191236

Cao YM, 2022, AM J RESP CELL MOL, V67, P201, DOI 10.1165/rcmb.2021-0504OC

Casanova-Acebes M, 2021, NATURE, V595, P578, DOI 10.1038/s41586-021-03651-8

Chakarov S, 2019, SCIENCE, V363, P1190, DOI 10.1126/science.aau0964

CHANEZ P, 1993, ALLERGY, V48, P576, DOI 10.1111/j.1398-9995.1993.tb00751.x

Chen F, 2022, CELL REP, V38, DOI 10.1016/j.celrep.2021.110215

COGGLE JE, 1984, J LEUKOCYTE BIOL, V35, P317

Cohen SB, 2018, CELL HOST MICROBE, V24, P439, DOI 10.1016/j.chom.2018.08.001

Daniel B, 2020, GENE DEV, V34, P1474, DOI 10.1101/gad.343038.120

De Smet EG, 2020, MUCOSAL IMMUNOL, V13, P423, DOI 10.1038/s41385-019-0241-6

Desch AN, 2016, AM J RESP CRIT CARE, V193, P614, DOI 10.1164/rccm.201507-1376OC

Dick SA, 2022, SCI IMMUNOL, V7, DOI 10.1126/sciimmunol.abf7777

Divangahi M, 2021, NAT IMMUNOL, V22, P2, DOI 10.1038/s41590-020-00845-6

DRANOFF G, 1994, SCIENCE, V264, P713, DOI 10.1126/science.8171324

Dutertre CA, 2019, IMMUNITY, V51, P573, DOI 10.1016/j.immuni.2019.08.008

Eguíluz-Gracia I, 2016, THORAX, V71, P1006, DOI 10.1136/thoraxjnl-2016-208292

Evren E, 2022, J EXP MED, V219, DOI 10.1084/jem.20210987

Fu YY, 2022, NAT COMMUN, V13, DOI 10.1038/s41467-022-31596-7

Fu YY, 2022, SCI IMMUNOL, V7, DOI 10.1126/sciimmunol.abi9768

Fujimori T, 2015, MUCOSAL IMMUNOL, V8, P1021, DOI 10.1038/mi.2014.129

Fukushima K, 2020, IMMUNITY, V52, P542, DOI 10.1016/j.immuni.2020.02.007

Gautier EL, 2012, NAT IMMUNOL, V13, P1118, DOI 10.1038/ni.2419

Gibbings SL, 2018, METHODS MOL BIOL, V1809, P33, DOI 10.1007/978-1-4939-8570-8_3

Gibbings SL, 2018, METHODS MOL BIOL, V1799, P381, DOI 10.1007/978-1-4939-7896-0_28

Gibbings SL, 2017, AM J RESP CELL MOL, V57, P66, DOI 10.1165/rcmb.2016-0361OC

Gibbings SL, 2015, BLOOD, V126, P1357, DOI 10.1182/blood-2015-01-624809

Gibbons MA, 2011, AM J RESP CRIT CARE, V184, P569, DOI 10.1164/rccm.201010-1719OC

Goenka A, 2020, FRONT IMMUNOL, V11, DOI 10.3389/fimmu.2020.00486

Goritzka M, 2015, J EXP MED, V212, P699, DOI 10.1084/jem.20140825

Gorki AD, 2022, AM J RESP CELL MOL, V66, P64, DOI 10.1165/rcmb.2021-0190OC

Grant RA, 2021, NATURE, V590, P635, DOI 10.1038/s41586-020-03148-w

Greiffo FR, 2020, EUR RESPIR J, V55, DOI 10.1183/13993003.00460-2019

Gschwend J, 2021, J EXP MED, V218, DOI 10.1084/jem.20210745

Guilliams M, 2022, CELL, V185, P379, DOI 10.1016/j.cell.2021.12.018

Guilliams M, 2016, IMMUNITY, V45, P669, DOI 10.1016/j.immuni.2016.08.015

Guilliams M, 2013, J EXP MED, V210, P1977, DOI 10.1084/jem.20131199

Guillon A, 2020, JCI INSIGHT, V5, DOI 10.1172/jci.insight.133042

Hashimoto D, 2013, IMMUNITY, V38, P792, DOI 10.1016/j.immuni.2013.04.004

Hegde S, 2021, IMMUNITY, V54, P875, DOI 10.1016/j.immuni.2021.04.004

Herold S, 2011, FRONT IMMUNOL, V2, DOI 10.3389/fimmu.2011.00065

Hoeffel G, 2015, IMMUNITY, V42, P665, DOI 10.1016/j.immuni.2015.03.011

Hu GA, 2019, EBIOMEDICINE, V45, P563, DOI 10.1016/j.ebiom.2019.06.039

Huang L, 2018, J EXP MED, V215, P1135, DOI 10.1084/jem.20172020

Huggins DN, 2021, CANCER RES, V81, P5284, DOI 10.1158/0008-5472.CAN-21-0101

Hume PS, 2020, AM J RESP CRIT CARE, V201, P1209, DOI 10.1164/rccm.201911-2105OC

Hung LY, 2019, MUCOSAL IMMUNOL, V12, P64, DOI 10.1038/s41385-018-0096-2

Ikeda N, 2018, SCI IMMUNOL, V3, DOI 10.1126/sciimmunol.aat0207

Jakkula M, 2000, AM J PHYSIOL-LUNG C, V279, pL600, DOI 10.1152/ajplung.2000.279.3.L600

Jakubzick C, 2003, J IMMUNOL, V171, P2684, DOI 10.4049/jimmunol.171.5.2684

Jakubzick C, 2013, IMMUNITY, V39, P599, DOI 10.1016/j.immuni.2013.08.007

Janssen WJ, 2008, AM J RESP CRIT CARE, V178, P158, DOI 10.1164/rccm.200711-1661OC

Janssen WJ, 2020, G3-GENES GENOM GENET, V10, P555, DOI 10.1534/g3.119.400935

Jardine L, 2019, NAT COMMUN, V10, DOI 10.1038/s41467-019-09913-4

Jenkins SJ, 2011, SCIENCE, V332, P1284, DOI 10.1126/science.1204351

Joshi N, 2020, EUR RESPIR J, V55, DOI 10.1183/13993003.00646-2019

Kawano H, 2016, INT IMMUNOL, V28, P489, DOI 10.1093/intimm/dxw012

Keerthivasan S, 2021, IMMUNITY, V54, P1511, DOI 10.1016/j.immuni.2021.06.012

Kono Y, 2021, SCI REP-UK, V11, DOI 10.1038/s41598-020-79848-0

Koth LL, 2007, AM J RESP CELL MOL, V37, P651, DOI 10.1165/rcmb.2006-0428OC

Kropski JA, 2019, ANNU REV MED, V70, P211, DOI 10.1146/annurev-med-041317-102715

Kurowska-Stolarska M, 2009, J IMMUNOL, V183, P6469, DOI 10.4049/jimmunol.0901575

Lafuse WP, 2019, J IMMUNOL, V203, P2252, DOI 10.4049/jimmunol.1900495

Lam TYW, 2022, P NATL ACAD SCI USA, V119, DOI 10.1073/pnas.2019161119

Larsen SB, 2021, CELL STEM CELL, V28, P1758, DOI 10.1016/j.stem.2021.07.001

Lauzon-Joset JF, 2014, MUCOSAL IMMUNOL, V7, P155, DOI 10.1038/mi.2013.34

Lavin Y, 2017, CELL, V169, P750, DOI 10.1016/j.cell.2017.04.014

Lavrich KS, 2018, AM J PHYSIOL-LUNG C, V315, pL752, DOI 10.1152/ajplung.00208.2018

Leach SM, 2020, CELL REP, V33, DOI 10.1016/j.celrep.2020.108337

Lechner AJ, 2017, CELL STEM CELL, V21, P120, DOI 10.1016/j.stem.2017.03.024

Lee YG, 2015, AM J RESP CELL MOL, V52, P772, DOI 10.1165/rcmb.2014-0255OC

Li X, 2022, LIFE SCI ALLIANCE, V5, DOI 10.26508/lsa.202201458

Liao MF, 2020, NAT MED, V26, P842, DOI 10.1038/s41591-020-0901-9

Lim HY, 2018, IMMUNITY, V49, P326, DOI [10.1016/j.immuni.2018.06.008, 10.1016/j.immuni.2018.12.009]

Liu ZY, 2019, CELL, V178, P1509, DOI 10.1016/j.cell.2019.08.009

Loyher PL, 2018, J EXP MED, V215, P2536, DOI 10.1084/jem.20180534

Ma RY, 2022, TRENDS IMMUNOL, V43, P546, DOI 10.1016/j.it.2022.04.008

Machiels B, 2017, NAT IMMUNOL, V18, P1310, DOI 10.1038/ni.3857

MacLean JA, 1996, AM J PATHOL, V148, P657

Martinez JAB, 1997, AM J PHYSIOL-LUNG C, V273, pL676, DOI 10.1152/ajplung.1997.273.3.L676

Mass E, 2016, SCIENCE, V353, DOI 10.1126/science.aaf4238

Mata E, 2021, SCI IMMUNOL, V6, DOI 10.1126/sciimmunol.abc2934

McCubbrey AL, 2018, AM J RESP CELL MOL, V58, P66, DOI 10.1165/rcmb.2017-0154OC

McCubbrey AL, 2017, FRONT IMMUNOL, V8, DOI 10.3389/fimmu.2017.01618

McQuattie-Pimentel AC, 2021, J CLIN INVEST, V131, DOI 10.1172/JCI140299

Medoff BD, 2009, J IMMUNOL, V182, P623, DOI 10.4049/jimmunol.182.1.623

Meghraoui-Kheddar A, 2020, FRONT IMMUNOL, V11, DOI 10.3389/fimmu.2020.01117

Meliopoulos VA, 2016, PLOS PATHOG, V12, DOI 10.1371/journal.ppat.1005804

Miki H, 2021, J ALLERGY CLIN IMMUN, V147, P1087, DOI 10.1016/j.jaci.2020.10.005

Minutti CM, 2019, IMMUNITY, V50, P645, DOI 10.1016/j.immuni.2019.01.008

Minutti CM, 2017, SCIENCE, V356, P1076, DOI 10.1126/science.aaj2067

Mirchandani AS, 2022, NAT IMMUNOL, V23, P927, DOI 10.1038/s41590-022-01216-z

Misharin AV, 2017, J EXP MED, V214, P2387, DOI 10.1084/jem.20162152

Misharin AV, 2013, AM J RESP CELL MOL, V49, P503, DOI 10.1165/rcmb.2013-0086MA

Mould KJ, 2021, AM J RESP CRIT CARE, V203, P946, DOI 10.1164/rccm.202005-1989OC

Mould KJ, 2019, JCI INSIGHT, V4, DOI 10.1172/jci.insight.126556

Mould KJ, 2017, AM J RESP CELL MOL, V57, P294, DOI 10.1165/rcmb.2017-0061OC

Mulder K, 2021, IMMUNITY, V54, P1883, DOI 10.1016/j.immuni.2021.07.007

Naessens T, 2012, AM J PATHOL, V181, P174, DOI 10.1016/j.ajpath.2012.03.015

Nakano H, 2015, J IMMUNOL, V194, P3808, DOI 10.4049/jimmunol.1402195

Nayak DK, 2016, AM J TRANSPLANT, V16, P2300, DOI 10.1111/ajt.13819

Neupane AS, 2020, CELL, V183, P110, DOI 10.1016/j.cell.2020.08.020

O'Neill LAJ, 2016, NAT REV IMMUNOL, V16, P553, DOI 10.1038/nri.2016.70

Tatsuro O, 2022, BIOCHEM BIOPH RES CO, V599, P113, DOI 10.1016/j.bbrc.2022.02.037

Papp AC, 2018, PLOS ONE, V13, DOI 10.1371/journal.pone.0198221

Perdiguero EG, 2015, NATURE, V518, P547, DOI 10.1038/nature13989

Phelps DS, 2011, PROTEOME SCI, V9, DOI 10.1186/1477-5956-9-67

Pisu D, 2020, CELL REP, V30, P335, DOI 10.1016/j.celrep.2019.12.033

Plantinga M, 2013, IMMUNITY, V38, P322, DOI 10.1016/j.immuni.2012.10.016

Podsiad A, 2016, AM J PHYSIOL-LUNG C, V310, pL465, DOI 10.1152/ajplung.00224.2015

Prasse A, 2006, AM J RESP CRIT CARE, V173, P781, DOI 10.1164/rccm.200509-1518OC

Rauschmeier R, 2019, EMBO J, V38, DOI 10.15252/embj.2018101233

Reyfman PA, 2019, AM J RESP CRIT CARE, V199, P1517, DOI 10.1164/rccm.201712-2410OC

Roberts AW, 2017, IMMUNITY, V47, P913, DOI 10.1016/j.immuni.2017.10.006

Rodero MP, 2015, ELIFE, V4, DOI 10.7554/eLife.07847

Roquilly A, 2020, NAT IMMUNOL, V21, P636, DOI 10.1038/s41590-020-0673-x

Rothchild AC, 2019, SCI IMMUNOL, V4, DOI 10.1126/sciimmunol.aaw6693

Sabatel C, 2017, IMMUNITY, V46, P457, DOI 10.1016/j.immuni.2017.02.016

Sajti E, 2020, NAT IMMUNOL, V21, P221, DOI 10.1038/s41590-019-0582-z

Satoh T, 2017, NATURE, V541, P96, DOI 10.1038/nature20611

Sauler M, 2022, NAT COMMUN, V13, DOI 10.1038/s41467-022-28062-9

SAWYER RT, 1982, LAB INVEST, V46, P165

Schneider C, 2014, NAT IMMUNOL, V15, P1026, DOI 10.1038/ni.3005

Schulz C, 2012, SCIENCE, V336, P86, DOI 10.1126/science.1219179

Schyns J, 2019, NAT COMMUN, V10, DOI 10.1038/s41467-019-11843-0

Serezani APM, 2022, AM J RESP CELL MOL, V67, P50, DOI 10.1165/rcmb.2021-0418OC

Singh A, 2022, P NATL ACAD SCI USA, V119, DOI 10.1073/pnas.2121098119

Snelgrove RJ, 2008, NAT IMMUNOL, V9, P1074, DOI 10.1038/ni.1637

Snyder ME, 2021, AM J RESP CRIT CARE, V203, P1230, DOI 10.1164/rccm.202006-2403OC

Soroosh P, 2013, J EXP MED, V210, P775, DOI 10.1084/jem.20121849

Soucie EL, 2016, SCIENCE, V351, DOI 10.1126/science.aad5510

STRICKLAND DH, 1993, IMMUNOLOGY, V80, P266

Sturrock A, 2014, J BIOL CHEM, V289, P4095, DOI 10.1074/jbc.M113.535922

Subramanian S, 2022, NAT IMMUNOL, V23, P458, DOI 10.1038/s41590-022-01146-w

Suzuki T, 2008, J EXP MED, V205, P2703, DOI 10.1084/jem.20080990

Svedberg FR, 2019, NAT IMMUNOL, V20, P571, DOI 10.1038/s41590-019-0352-y

Tang CB, 1998, AM J RESP CRIT CARE, V157, P1120, DOI 10.1164/ajrccm.157.4.9706118

Tang X.-Z., 2022, BIORXIV, DOI [10.1016/2020.2009.2018.247742, DOI 10.1016/2020.2009.2018.247742]

TARLING JD, 1982, CELL TISSUE KINET, V15, P577, DOI 10.1111/j.1365-2184.1982.tb01064.x

Tazawa R, 2019, NEW ENGL J MED, V381, P923, DOI 10.1056/NEJMoa1816216

Tewari A, 2021, FRONT IMMUNOL, V12, DOI 10.3389/fimmu.2021.763379

THEPEN T, 1991, EUR J IMMUNOL, V21, P2845, DOI 10.1002/eji.1830211128

THEPEN T, 1989, J EXP MED, V170, P499, DOI 10.1084/jem.170.2.499

THEPEN T, 1992, CLIN EXP ALLERGY, V22, P1107, DOI 10.1111/j.1365-2222.1992.tb00137.x

Todd EM, 2016, BLOOD, V128, P2785, DOI 10.1182/blood-2016-03-705962

Ural BB, 2020, SCI IMMUNOL, V5, DOI 10.1126/sciimmunol.aax8756

van de Laar L, 2016, IMMUNITY, V44, P755, DOI 10.1016/j.immuni.2016.02.017

Vermaelen K, 2004, CYTOM PART A, V61A, P170, DOI 10.1002/cyto.a.20064

Viksman MY, 2002, CLIN IMMUNOL, V104, P77, DOI 10.1006/clim.2002.5233

VOLKMAN A, 1983, LAB INVEST, V49, P291

VOLKMAN A, 1965, BRIT J EXP PATHOL, V46, P62

Wang W, 2016, AM J PHYSIOL-LUNG C, V311, pL494, DOI 10.1152/ajplung.00001.2016

Wendisch D, 2021, CELL, V184, P6243, DOI 10.1016/j.cell.2021.11.033

Westphalen K, 2014, NATURE, V506, P503, DOI 10.1038/nature12902

Willart MAM, 2009, J EXP MED, V206, P2823, DOI 10.1084/jem.20082401

Wong CK, 2017, J IMMUNOL, V199, P1060, DOI 10.4049/jimmunol.1700397

Wrench C, 2018, AM J RESP CELL MOL, V58, P271, DOI 10.1165/rcmb.2017-0351LE

Wu KY, 2020, J IMMUNOL, V205, P1084, DOI 10.4049/jimmunol.2000181

Wu K, 2015, J EXP MED, V212, P681, DOI 10.1084/jem.20141732

Xu D, 2022, JCI INSIGHT, V7, DOI 10.1172/jci.insight.151037

Xu-Vanpala S, 2020, SCI IMMUNOL, V5, DOI 10.1126/sciimmunol.aba7350

Yona S, 2013, IMMUNITY, V38, P79, DOI 10.1016/j.immuni.2012.12.001

Yu X., 2017, Immunity

Yu YRA, 2016, AM J RESP CELL MOL, V54, P13, DOI 10.1165/rcmb.2015-0146OC

Zhou BS, 2020, NAT IMMUNOL, V21, P1430, DOI 10.1038/s41590-020-0764-8

NR 186

TC 231

Z9 244

U1 31

U2 126

PU CELL PRESS

PI CAMBRIDGE

PA 50 HAMPSHIRE ST, FLOOR 5, CAMBRIDGE, MA 02139 USA

SN 1074-7613

EI 1097-4180

J9 IMMUNITY

JI Immunity

PD SEP 13

PY 2022

VL 55

IS 9

BP 1564

EP 1580

DI 10.1016/j.immuni.2022.08.010

PG 17

WC Immunology

WE Science Citation Index Expanded (SCI-EXPANDED)

SC Immunology

GA 8F9PC

UT WOS:000919985400002

PM 36103853

OA Green Accepted, Green Published, Bronze

HC Y

HP N

DA 2025-02-07

ER

PT J

AU La, H

Zhu, B

Chen, DG

AF La, Hao

Zhu, Bo

Chen, Degao

TI The heterogeneity of tumor-associated macrophages and strategies to

target it

SO BIOCELL

LA English

DT Article

DE Tumor-associated macrophages; Tissue-resident macrophages;

Heterogeneity; Immune checkpoint therapy

ID TISSUE-RESIDENT MACROPHAGES; PANCREATIC-CANCER; MONOCLONAL-ANTIBODY;

CXCR4 INHIBITION; OPEN-LABEL; GM-CSF; MICROENVIRONMENT; BLOCKADE; CELLS;

CCL2

AB Tumor-associated macrophages (TAMs) are emerging as targets for tumor therapy because of their primary role in promoting tumor progression. Several studies have been conducted to target TAMs by reducing their infiltration, depleting their numbers, and reversing their phenotypes to suppress tumor progression, leading to the development of drugs in preclinical and clinical trials. However, the heterogeneous characteristics of TAMs, including their ontogenetic and functional heterogeneity, limit their targeting. Therefore, in-depth exploration of the heterogeneity of TAMs, combined with immune checkpoint therapy or other therapeutic modalities could improve the efficiency of tumor treatment. This review focuses on the heterogeneous ontogeny and function of TAMs, as well as the current development of tumor therapies targeting TAMs and combination strategies.

C1 [La, Hao; Zhu, Bo; Chen, Degao] Third Mil Med Univ, Xinqiao Hosp, Inst Canc, Chongqing 400037, Peoples R China.

[Zhu, Bo; Chen, Degao] Third Mil Med Univ, Xinqiao Hosp, Chongqing Key Lab Immunotherapy, Chongqing 400037, Peoples R China.

C3 Army Medical University; Army Medical University

RP Chen, DG (corresponding author), Third Mil Med Univ, Xinqiao Hosp, Inst Canc, Chongqing 400037, Peoples R China.; Chen, DG (corresponding author), Third Mil Med Univ, Xinqiao Hosp, Chongqing Key Lab Immunotherapy, Chongqing 400037, Peoples R China.

EM degaochen@tmmu.edu.cn

FU National Natural Science Foundation of China [82003018]

FX Funding Statement: This work was supported by the National Natural

Science Foundation of China (82003018) .

CR Aegerter H, 2022, IMMUNITY, V55, P1564, DOI 10.1016/j.immuni.2022.08.010

Aendekerk JP, 2022, J AUTOIMMUN, V133, DOI 10.1016/j.jaut.2022.102914

Ali HR, 2016, PLOS MED, V13, DOI 10.1371/journal.pmed.1002194

Amorim A, 2022, NAT IMMUNOL, V23, P217, DOI 10.1038/s41590-021-01117-7

Atkins MH, 2021, J EXP MED, V219, DOI 10.1084/jem.20211924

Barone A, 2017, CLIN CANCER RES, V23, P7448, DOI 10.1158/1078-0432.CCR-17-0898

Beatty GL, 2011, SCIENCE, V331, P1612, DOI 10.1126/science.1198443

Becherini C, 2023, STRAHLENTHER ONKOL, V199, P1173, DOI 10.1007/s00066-023-02097-3

Belgiovine C, 2017, BRIT J CANCER, V117, P628, DOI 10.1038/bjc.2017.205

Biasci D, 2020, P NATL ACAD SCI USA, V117, P28960, DOI 10.1073/pnas.2013644117

Bill R, 2023, SCIENCE, V381, P515, DOI 10.1126/science.ade2292

Binnewies M, 2021, CELL REP, V37, DOI 10.1016/j.celrep.2021.109844

Bockorny B, 2021, CLIN CANCER RES, V27, P5020, DOI 10.1158/1078-0432.CCR-21-0929

Bockorny B, 2020, NAT MED, V26, P878, DOI 10.1038/s41591-020-0880-x

Brana I, 2015, TARGET ONCOL, V10, P111, DOI 10.1007/s11523-014-0320-2

Bugatti M, 2022, CANCER IMMUNOL RES, V10, P1340, DOI 10.1158/2326-6066.CIR-22-0271

Butowski N, 2016, NEURO-ONCOLOGY, V18, P557, DOI 10.1093/neuonc/nov245

Casanova-Acebes M, 2021, NATURE, V595, P578, DOI 10.1038/s41586-021-03651-8

Cassetta L, 2019, CANCER CELL, V35, P588, DOI 10.1016/j.ccell.2019.02.009

Cassetta L, 2018, NAT REV DRUG DISCOV, V17, P887, DOI 10.1038/nrd.2018.169

Cassier PA, 2015, LANCET ONCOL, V16, P949, DOI 10.1016/S1470-2045(15)00132-1

Chen C, 2023, MOL PHARMACEUT, V20, P971, DOI 10.1021/acs.molpharmaceut.2c00557

Chen DG, 2021, THERANOSTICS, V11, P1016, DOI 10.7150/thno.51777

Chen DG, 2018, NAT COMMUN, V9, DOI 10.1038/s41467-018-03225-9

Chen SH, 2021, J IMMUNOTHER CANCER, V9, DOI 10.1136/jitc-2021-003464

Chen YC, 2015, HEPATOLOGY, V61, P1591, DOI 10.1002/hep.27665

Chen YC, 2014, HEPATOLOGY, V59, P1435, DOI 10.1002/hep.26790

Chevrier S, 2017, CELL, V169, P736, DOI 10.1016/j.cell.2017.04.016

Chiang Y, 2022, INT J RADIAT ONCOL, V114, P321, DOI 10.1016/j.ijrobp.2022.06.054

Cieslewicz M, 2013, P NATL ACAD SCI USA, V110, P15919, DOI 10.1073/pnas.1312197110

Cortez-Retamozo V, 2013, IMMUNITY, V38, P296, DOI 10.1016/j.immuni.2012.10.015

Coveler AL, 2023, J IMMUNOTHER CANCER, V11, DOI 10.1136/jitc-2022-005584

Dai XM, 2020, THERANOSTICS, V10, P9332, DOI 10.7150/thno.47137

De Henau O, 2016, NATURE, V539, P443, DOI 10.1038/nature20554

DeNardo DG, 2011, CANCER DISCOV, V1, P54, DOI 10.1158/2159-8274.CD-10-0028

El Chartouni C, 2010, IMMUNOBIOLOGY, V215, P466, DOI 10.1016/j.imbio.2009.08.002

Evren E, 2021, IMMUNITY, V54, P259, DOI 10.1016/j.immuni.2020.12.003

Franklin RA, 2016, TRENDS CANCER, V2, P20, DOI 10.1016/j.trecan.2015.11.004

Franklin RA, 2014, SCIENCE, V344, P921, DOI 10.1126/science.1252510

Fuentelsaz-Romero S, 2021, FRONT IMMUNOL, V11, DOI 10.3389/fimmu.2020.613975

Gentles AJ, 2015, NAT MED, V21, P938, DOI 10.1038/nm.3909

Germano G, 2013, CANCER CELL, V23, P249, DOI 10.1016/j.ccr.2013.01.008

Ghobrial IM, 2020, CLIN CANCER RES, V26, P344, DOI 10.1158/1078-0432.CCR-19-0647

Gubin MM, 2018, CELL, V175, P1014, DOI 10.1016/j.cell.2018.09.030

Guillot J, 2022, NAT COMMUN, V13, DOI 10.1038/s41467-022-29659-w

Gunassekaran GR, 2021, BIOMATERIALS, V278, DOI 10.1016/j.biomaterials.2021.121137

Hao XP, 2022, REDOX BIOL, V56, DOI 10.1016/j.redox.2022.102463

He S, 2021, FRONT PHARMACOL, V12, DOI 10.3389/fphar.2021.743837

Heidegger I, 2022, MOL CANCER, V21, DOI 10.1186/s12943-022-01597-7

Hoves S, 2018, J EXP MED, V215, P859, DOI 10.1084/jem.20171440

Huynh C, 2020, PHARMACOL RES, V161, DOI 10.1016/j.phrs.2020.105092

Iwamoto H, 2020, INT J MOL SCI, V21, DOI 10.3390/ijms21239328

Jin L, 2022, PHYTOMEDICINE, V104, DOI 10.1016/j.phymed.2022.154307

Kawaguchi Y, 2023, CANCER SCI, V114, P750, DOI 10.1111/cas.15671

Khalaji A, 2023, HELIYON, V9, DOI 10.1016/j.heliyon.2023.e20507

Khan F, 2023, J CLIN INVEST, V133, DOI 10.1172/JCI163446

Kuo TC, 2020, J HEMATOL ONCOL, V13, DOI 10.1186/s13045-020-00989-w

Kvorning SL, 2020, EUR J HAEMATOL, V104, P409, DOI 10.1111/ejh.13371

Lamb YN, 2019, DRUGS, V79, P1805, DOI 10.1007/s40265-019-01210-0

Lazarov T, 2023, NATURE, V618, P698, DOI 10.1038/s41586-023-06002-x

Li ZT, 2022, NAT COMMUN, V13, DOI 10.1038/s41467-022-29388-0

Li ZQ, 2021, SCI ADV, V7, DOI 10.1126/sciadv.abb6260

Liu XJ, 2018, CELL REP, V24, P2101, DOI 10.1016/j.celrep.2018.07.062

Liu Y, 2020, ACS APPL MATER INTER, V12, P52402, DOI 10.1021/acsami.0c15983

Liu Y, 2014, CELL MOL IMMUNOL, V11, P49, DOI [10.1038/cmi.2014.83, 10.1038/cmi.2013.40]

Loberg RD, 2007, CANCER RES, V67, P9417, DOI 10.1158/0008-5472.CAN-07-1286

Loberg RD, 2007, NEOPLASIA, V9, P556, DOI 10.1593/neo.07307

Lu XF, 2019, IMMUNOBIOLOGY, V224, P355, DOI 10.1016/j.imbio.2019.03.002

Mantovani A, 2017, NAT REV CLIN ONCOL, V14, P399, DOI 10.1038/nrclinonc.2016.217

Meng YM, 2018, ONCOIMMUNOLOGY, V7, DOI 10.1080/2162402X.2017.1408745

Miller JE, 2020, FRONT IMMUNOL, V11, DOI 10.3389/fimmu.2020.00108

Mortezaee K, 2022, CELL ONCOL, V45, P333, DOI 10.1007/s13402-022-00667-8

Mulder K, 2021, IMMUNITY, V54, P1883, DOI 10.1016/j.immuni.2021.07.007

Nakasone ES, 2012, CANCER CELL, V21, P488, DOI 10.1016/j.ccr.2012.02.017

Ngambenjawong C, 2016, J CONTROL RELEASE, V224, P103, DOI 10.1016/j.jconrel.2015.12.057

Nie Y, 2019, CLIN CANCER RES, V25, P3873, DOI 10.1158/1078-0432.CCR-18-3421

Nywening TM, 2016, LANCET ONCOL, V17, P651, DOI 10.1016/S1470-2045(16)00078-4

Palmieri EM, 2017, CELL REP, V20, P1654, DOI 10.1016/j.celrep.2017.07.054

Park DJ, 2021, INT J MOL SCI, V22, DOI 10.3390/ijms22094710

Peranzoni E, 2018, P NATL ACAD SCI USA, V115, pE4041, DOI 10.1073/pnas.1720948115

Perdiguero EG, 2015, NATURE, V518, P547, DOI 10.1038/nature13989

Perry CJ, 2018, J EXP MED, V215, P877, DOI 10.1084/jem.20171435

Pienta KJ, 2013, INVEST NEW DRUG, V31, P760, DOI 10.1007/s10637-012-9869-8

Pittet MJ, 2022, NAT REV CLIN ONCOL, V19, P402, DOI 10.1038/s41571-022-00620-6

Pradel LP, 2016, MOL CANCER THER, V15, P3077, DOI 10.1158/1535-7163.MCT-16-0157

Prieto LI, 2023, CANCER CELL, V41, P1261, DOI 10.1016/j.ccell.2023.05.006

Qiu SK, 2022, J EXP CLIN CANC RES, V41, DOI 10.1186/s13046-022-02499-8

Ramos RN, 2022, CELL, V185, P1189, DOI 10.1016/j.cell.2022.02.021

Rao R, 2022, NEURO-ONCOLOGY, V24, P584, DOI 10.1093/neuonc/noab228

Ries CH, 2014, CANCER CELL, V25, P846, DOI 10.1016/j.ccr.2014.05.016

Rioja-Blanco E, 2022, J EXP CLIN CANC RES, V41, DOI 10.1186/s13046-022-02267-8

Rolny C, 2011, CANCER CELL, V19, P31, DOI 10.1016/j.ccr.2010.11.009

Salmaninejad A, 2019, CELL ONCOL, V42, P591, DOI 10.1007/s13402-019-00453-z

Schürch CM, 2019, THYROID, V29, P979, DOI 10.1089/thy.2018.0555

Seo YD, 2019, CLIN CANCER RES, V25, P3934, DOI 10.1158/1078-0432.CCR-19-0081

Shiao SL, 2015, CANCER IMMUNOL RES, V3, P518, DOI 10.1158/2326-6066.CIR-14-0232

Sockolosky JT, 2016, P NATL ACAD SCI USA, V113, pE2646, DOI 10.1073/pnas.1604268113

Song YD, 2022, SCI TRANSL MED, V14, DOI 10.1126/scitranslmed.abl3649

Sun Y, 2023, MOL PHARMACEUT, V20, P5921, DOI 10.1021/acs.molpharmaceut.3c00769

Tap WD, 2015, NEW ENGL J MED, V373, P428, DOI 10.1056/NEJMoa1411366

Ushach I, 2016, J LEUKOCYTE BIOL, V100, P481, DOI 10.1189/jlb.3RU0316-144R

Vaeteewoottacharn K, 2019, TRANSL ONCOL, V12, P217, DOI 10.1016/j.tranon.2018.10.007

van der Heide D, 2019, FRONT IMMUNOL, V10, DOI 10.3389/fimmu.2019.02852

Varol C, 2015, ANNU REV IMMUNOL, V33, P643, DOI 10.1146/annurev-immunol-032414-112220

Vidyarthi A, 2019, CANCER IMMUNOL IMMUN, V68, P1995, DOI 10.1007/s00262-019-02423-8

Wang JX, 2022, J EXP MED, V219, DOI 10.1084/jem.20211631

Wang YH, 2022, BIOACT MATER, V17, P147, DOI 10.1016/j.bioactmat.2022.01.003

Weiss SA, 2024, CLIN CANCER RES, V30, P74, DOI 10.1158/1078-0432.CCR-23-0475

Weng YS, 2019, MOL CANCER, V18, DOI 10.1186/s12943-019-0988-0

Winning S, 2022, ANTIOXID REDOX SIGN, V37, P956, DOI 10.1089/ars.2022.0004

Wu QC, 2019, HEPATOLOGY, V70, P198, DOI 10.1002/hep.30593

Xu JY, 2013, CANCER RES, V73, P2782, DOI 10.1158/0008-5472.CAN-12-3981

Xu L, 2021, METABOLISM, V125, DOI 10.1016/j.metabol.2021.154914

Yang H, 2020, MOL CANCER, V19, DOI 10.1186/s12943-020-01165-x

Yang XB, 2011, AM J RESP CELL MOL, V45, P127, DOI 10.1165/rcmb.2010-0265OC

Yin HL, 2021, NAT COMMUN, V12, DOI 10.1038/s41467-021-21514-8

Yin YZ, 2023, CLIN TRANSL MED, V13, DOI 10.1002/ctm2.1283

Zeng Z, 2022, GENES DIS, V9, P12, DOI 10.1016/j.gendis.2021.08.004

Zhang N, 2020, J AGR FOOD CHEM, V68, P11182, DOI 10.1021/acs.jafc.0c04041

Zhang W, 2023, FRONT PHARMACOL, V14, DOI 10.3389/fphar.2023.1092767

Zhang XM, 2021, CELLS-BASEL, V10, DOI 10.3390/cells10040903

Zhou CH, 2023, GASTROENTEROLOGY, V164, P1261, DOI 10.1053/j.gastro.2023.02.005

Zhu Y, 2019, GUT, V68, P1653, DOI 10.1136/gutjnl-2019-318419

Zhu Y, 2017, IMMUNITY, V47, P323, DOI 10.1016/j.immuni.2017.07.014

Zhu Y, 2014, CANCER RES, V74, P5057, DOI 10.1158/0008-5472.CAN-13-3723

NR 125

TC 1

Z9 1

U1 2

U2 3

PU TECH SCIENCE PRESS

PI HENDERSON

PA 871 CORONADO CENTER DR, SUTE 200, HENDERSON, NV 89052 USA

SN 0327-9545

EI 1667-5746

J9 BIOCELL

JI Biocell

PY 2024

VL 48

IS 3

BP 363

EP 378

DI 10.32604/biocell.2023.046367

EA JAN 2024

PG 16

WC Biology

WE Science Citation Index Expanded (SCI-EXPANDED)

SC Life Sciences & Biomedicine - Other Topics

GA NU8Y5

UT WOS:001147498700001

OA gold

DA 2025-02-07

ER

PT J

AU Busa, R

Bulati, M

Badami, E

Zito, G

Maresca, DC

Conaldi, PG

Ercolano, G

Lanaro, A

AF Busa, Rosalia

Bulati, Matteo

Badami, Ester

Zito, Giovanni

Maresca, Daniela Claudia

Conaldi, Pier Giulio

Ercolano, Giuseppe

Lanaro, Angela

TI Tissue-Resident Innate Immune Cell-Based Therapy: A Cornerstone of

Immunotherapy Strategies for Cancer Treatment

SO FRONTIERS IN CELL AND DEVELOPMENTAL BIOLOGY

LA English

DT Review

DE innate immune cells; macrophages; innate lymphoid cells (ILC); NK cells;

tissue-resident immune cells; cancer; MDSC (myeloid-derived suppressor

cell)

ID KILLER-T-CELLS; PLASMACYTOID DENDRITIC CELLS; TUMOR-ASSOCIATED

MACROPHAGES; INVARIANT-NKT CELLS; SUPPRESSOR-CELLS; LYMPHOID-CELLS;

BREAST-CANCER; POOR-PROGNOSIS; MYELOID CELLS; STEADY-STATE

AB Cancer immunotherapy has led to impressive advances in cancer treatment. Unfortunately, in a high percentage of patients is difficult to consistently restore immune responses to eradicate established tumors. It is well accepted that adaptive immune cells, such as B lymphocytes, CD4(+) helper T lymphocytes, and CD8(+) cytotoxic T-lymphocytes (CTLs), are the most effective cells able to eliminate tumors. However, it has been recently reported that innate immune cells, including natural killer cells (NK), dendritic cells (DC), macrophages, myeloid-derived suppressor cells (MDSCs), and innate lymphoid cells (ILCs), represent important contributors to modulating the tumor microenvironment and shaping the adaptive tumor response. In fact, their role as a bridge to adaptive immunity, make them an attractive therapeutic target for cancer treatment. Here, we provide a comprehensive overview of the pleiotropic role of tissue-resident innate immune cells in different tumor contexts. In addition, we discuss how current and future therapeutic approaches targeting innate immune cells sustain the adaptive immune system in order to improve the efficacy of current tumor immunotherapies.

C1 [Busa, Rosalia; Bulati, Matteo; Badami, Ester; Zito, Giovanni; Conaldi, Pier Giulio] Mediterranean Inst Transplantat & Adv Specialized, Res Dept, Palermo, Italy.

[Badami, Ester] RiMED Fdn, Palermo, Italy.

[Maresca, Daniela Claudia; Ercolano, Giuseppe; Lanaro, Angela] Univ Naples Federico II, Dept Pharm, Sch Med, Naples, Italy.

C3 IRCCS Istituto Mediterraneo per i Trapianti e Terapie ad Alta

Specializzazione (ISMETT); University of Naples Federico II

RP Ercolano, G (corresponding author), Univ Naples Federico II, Dept Pharm, Sch Med, Naples, Italy.

EM Giuseppe.ercolano@unina.it

RI Busa, Rosalia/AAB-8340-2022; Zito, Giovanni/AAC-3639-2022; Conaldi,

Pier/AAC-1573-2019; Ercolano, Giuseppe/GOJ-9504-2022; Bulati,

Matteo/AAT-1915-2020

OI Busa, Rosalia/0000-0002-7546-7209; Maresca, Daniela

Claudia/0009-0008-4221-6375

FU Associazione Italiana per la Ricerca sul Cancro (AIRC) [26002]; Italian

Government (PRIN 2017) [2017BA9LM5]

FX This work was supported by Associazione Italiana per la Ricerca sul

Cancro (AIRC) (MFAG No. 26002 to GE), and by the Italian Government

grants (PRIN 2017 No. 2017BA9LM5 to AI).

CR Ali Tahir SM, 2001, J IMMUNOL, V167, P4046

Ali TH, 2014, NAT COMMUN, V5, DOI 10.1038/ncomms6639

Allavena P, 2005, CANCER RES, V65, P2964, DOI 10.1158/0008-5472.CAN-04-4037

André P, 2018, CELL, V175, P1731, DOI 10.1016/j.cell.2018.10.014

Andreu P, 2010, CANCER CELL, V17, P121, DOI 10.1016/j.ccr.2009.12.019

Anfray C, 2020, CELLS-BASEL, V9, DOI 10.3390/cells9010046

Ao JY, 2017, MOL CANCER THER, V16, P1544, DOI 10.1158/1535-7163.MCT-16-0866

Bagchi S, 2021, ANNU REV PATHOL-MECH, V16, P223, DOI 10.1146/annurev-pathol-042020-042741

Balachandran VP, 2011, NAT MED, V17, P1094, DOI 10.1038/nm.2438

Baloyan D., 2022, INHIBITION TUFT CELL

Bar-Ephraim YE, 2019, J IMMUNOL, V202, P171, DOI 10.4049/jimmunol.1701153

Baroni S, 2016, CELL DEATH DIS, V7, DOI 10.1038/cddis.2016.224

Barry KC, 2018, NAT MED, V24, P1178, DOI 10.1038/s41591-018-0085-8

Baumann T, 2020, NAT IMMUNOL, V21, P555, DOI 10.1038/s41590-020-0666-9

Belikov AV, 2015, J BIOMED SCI, V22, DOI 10.1186/s12929-015-0194-3

Bentebibel SE, 2019, CANCER DISCOV, V9, P711, DOI 10.1158/2159-8290.CD-18-1495

Bentz BG, 2000, HEAD NECK-J SCI SPEC, V22, P64, DOI 10.1002/(SICI)1097-0347(200001)22:1<64::AID-HED10>3.0.CO;2-J

Berahovich RD, 2006, J IMMUNOL, V177, P7833, DOI 10.4049/jimmunol.177.11.7833

Bingisser RM, 1998, J IMMUNOL, V160, P5729

Binnewies M, 2019, CELL, V177, P556, DOI 10.1016/j.cell.2019.02.005

Blumenthal RL, 2001, J ALLERGY CLIN IMMUN, V107, P258, DOI 10.1067/mai.2001.112845

Bockorny B, 2020, NAT MED, V26, P878, DOI 10.1038/s41591-020-0880-x

Bosurgi L, 2017, SCIENCE, V356, P1072, DOI 10.1126/science.aai8132

Boutilier AJ, 2021, INT J MOL SCI, V22, DOI 10.3390/ijms22136995

Brown S, 2014, EUR J HAEMATOL, V93, P96, DOI 10.1111/ejh.12306

Bruchard M, 2019, FRONT IMMUNOL, V10, DOI 10.3389/fimmu.2019.00656

Carrega P, 2014, J IMMUNOL, V192, P3805, DOI 10.4049/jimmunol.1301889

Chen YW, 2022, J EXP MED, V219, DOI 10.1084/jem.20211805

Cheng JN, 2021, FRONT CELL DEV BIOL, V9, DOI 10.3389/fcell.2021.740827

Cheng ML, 2015, SCI REP-UK, V5, DOI 10.1038/srep10752

Cheng X, 2022, J IMMUNOL RES, V2022, DOI 10.1155/2022/5254911

Chong TW, 2015, J CLIN PATHOL, V68, P200, DOI 10.1136/jclinpath-2014-202735

Chung AW, 2021, SCI TRANSL MED, V13, DOI 10.1126/scitranslmed.abj5070

Cichocki F, 2019, FRONT IMMUNOL, V10, DOI 10.3389/fimmu.2019.02078

Cisse B, 2008, CELL, V135, P37, DOI 10.1016/j.cell.2008.09.016

Close HJ, 2020, CLIN EXP IMMUNOL, V200, P33, DOI 10.1111/cei.13403

Cobbs CS, 2003, CANCER RES, V63, P8670

Collin M, 2013, IMMUNOLOGY, V140, P22, DOI 10.1111/imm.12117

Concha-Benavente F, 2018, CANCER IMMUNOL RES, V6, P1548, DOI 10.1158/2326-6066.CIR-18-0062

Consonni FM, 2019, FRONT IMMUNOL, V10, DOI 10.3389/fimmu.2019.00949

Cooper MA, 2001, BLOOD, V97, P3146, DOI 10.1182/blood.V97.10.3146

Cózar B, 2021, CANCER DISCOV, V11, P34, DOI 10.1158/2159-8290.CD-20-0655

Crinier A, 2021, CELL MOL IMMUNOL, V18, P1290, DOI 10.1038/s41423-020-00574-8

Cursons J, 2019, CANCER IMMUNOL RES, V7, P1162, DOI 10.1158/2326-6066.CIR-18-0500

Curti A, 2007, BLOOD, V109, P2871, DOI 10.1182/blood-2006-07-036863

Dadi S, 2016, CELL, V164, P365, DOI 10.1016/j.cell.2016.01.002

Daussy C, 2014, J EXP MED, V211, P563, DOI 10.1084/jem.20131560

De Cicco P, 2020, FRONT IMMUNOL, V11, DOI 10.3389/fimmu.2020.01680

de Jonge K, 2019, SCI REP-UK, V9, DOI 10.1038/s41598-019-40933-8

DeNardo DG, 2009, CANCER CELL, V16, P91, DOI 10.1016/j.ccr.2009.06.018

Deng Z, 2017, ONCOGENE, V36, P639, DOI 10.1038/onc.2016.229

Dhara V, 2023, DM-DIS MON, V69, DOI 10.1016/j.disamonth.2022.101353

Dhodapkar KM, 2004, INT J CANCER, V109, P893, DOI 10.1002/ijc.20050

Dhodapkar MV, 2003, J EXP MED, V197, P1667, DOI 10.1084/jem.20021650

Di Censo C, 2021, EUR J IMMUNOL, V51, P2568, DOI 10.1002/eji.202149209

Diab A, 2020, CANCER DISCOV, V10, P1158, DOI 10.1158/2159-8290.CD-19-1510

Diamond MS, 2011, J EXP MED, V208, P1989, DOI 10.1084/jem.20101158

Dodagatta-Marri E, 2019, J IMMUNOTHER CANCER, V7, DOI 10.1186/s40425-018-0493-9

Dzionek A, 2000, J IMMUNOL, V165, P6037, DOI 10.4049/jimmunol.165.11.6037

Edwards VDK, 2019, BLOOD, V133, P588, DOI 10.1182/blood-2018-03-838946

Eisenring M, 2010, NAT IMMUNOL, V11, P1030, DOI 10.1038/ni.1947

Elliott JM, 2011, TRENDS IMMUNOL, V32, P364, DOI 10.1016/j.it.2011.06.001

Epelman S, 2014, IMMUNITY, V41, P21, DOI 10.1016/j.immuni.2014.06.013

Epelman S, 2014, IMMUNITY, V40, P91, DOI 10.1016/j.immuni.2013.11.019

Ercolano G, 2023, J INVEST ALLERG CLIN, V33, P129, DOI 10.18176/jiaci.0815

Ercolano G, 2021, NAT COMMUN, V12, DOI 10.1038/s41467-021-22764-2

Ercolano G, 2020, J LEUKOCYTE BIOL, V108, P723, DOI 10.1002/JLB.5MA0120-209R

Ercolano G, 2020, CANCER IMMUNOL RES, V8, P556, DOI 10.1158/2326-6066.CIR-19-0504

Ercolano G, 2019, FRONT IMMUNOL, V10, DOI 10.3389/fimmu.2019.02801

Fallarini S, 2012, BRIT J PHARMACOL, V167, P1533, DOI 10.1111/j.1476-5381.2012.02108.x

Fallarino F, 2006, J IMMUNOL, V176, P6752, DOI 10.4049/jimmunol.176.11.6752

Fan X, 2016, CELL, V164, P1198, DOI 10.1016/j.cell.2016.02.048

Feng S, 2018, P NATL ACAD SCI USA, V115, P10094, DOI 10.1073/pnas.1800695115

Feng YH, 2011, CANCER BIOL THER, V11, P111, DOI 10.4161/cbt.11.1.13965

Fleet JC, 2020, J STEROID BIOCHEM, V198, DOI 10.1016/j.jsbmb.2019.105557

Freud AG, 2017, IMMUNITY, V47, P820, DOI 10.1016/j.immuni.2017.10.008

Fridman WH, 2012, NAT REV CANCER, V12, P298, DOI 10.1038/nrc3245

Friedrich C, 2021, NAT IMMUNOL, V22, P1256, DOI 10.1038/s41590-021-01013-0

Fu S, 2004, AM J TRANSPLANT, V4, P1614, DOI 10.1111/j.1600-6143.2004.00566.x

Fuertes MB, 2011, J EXP MED, V208, P2005, DOI 10.1084/jem.20101159

Fujii S, 2003, BRIT J HAEMATOL, V122, P617, DOI 10.1046/j.1365-2141.2003.04465.x

Gabrilovich DI, 2012, NAT REV IMMUNOL, V12, P253, DOI 10.1038/nri3175

Gabrilovich DI, 2009, NAT REV IMMUNOL, V9, P162, DOI 10.1038/nri2506

Galland S, 2017, CELL REP, V20, P2891, DOI 10.1016/j.celrep.2017.08.089

Galot R, 2021, EUR J CANCER, V158, P17, DOI 10.1016/j.ejca.2021.09.003

Galsky MD, 2014, CLIN CANCER RES, V20, P3581, DOI 10.1158/1078-0432.CCR-13-2686

Ganguly D, 2013, NAT REV IMMUNOL, V13, P566, DOI 10.1038/nri3477

Gao YL, 2017, NAT IMMUNOL, V18, P1004, DOI 10.1038/ni.3800

Gardner A, 2020, FRONT IMMUNOL, V11, DOI 10.3389/fimmu.2020.00924

Geissmann F, 2010, SCIENCE, V327, P656, DOI 10.1126/science.1178331

Gerlini G, 2007, CLIN IMMUNOL, V125, P184, DOI 10.1016/j.clim.2007.07.018

Germano G, 2013, CANCER CELL, V23, P249, DOI 10.1016/j.ccr.2013.01.008

Ghaedi M, 2020, CELL RES, V30, P562, DOI 10.1038/s41422-020-0326-5

Ghinnagow R, 2017, FRONT IMMUNOL, V8, DOI 10.3389/fimmu.2017.00879

Ghiringhelli F, 2005, J EXP MED, V202, P1075, DOI 10.1084/jem.20051511

Ghobrial IM, 2020, CLIN CANCER RES, V26, P344, DOI 10.1158/1078-0432.CCR-19-0647

Giallongo C, 2018, J CELL MOL MED, V22, P1070, DOI 10.1111/jcmm.13326

Gilliet M, 2008, NAT REV IMMUNOL, V8, P594, DOI 10.1038/nri2358

Goff SL, 2021, CLIN BREAST CANCER, V21, pE63, DOI 10.1016/j.clbc.2020.06.011

Gomez-Cadena A, 2020, METHOD ENZYMOL, V631, P328, DOI 10.1016/bs.mie.2019.10.022

Gordon EM, 2016, ADV THER, V33, P1055, DOI 10.1007/s12325-016-0344-3

Gorelik L, 2002, J EXP MED, V195, P1499, DOI 10.1084/jem.20012076

Gray JI, 2022, ANNU REV IMMUNOL, V40, P195, DOI 10.1146/annurev-immunol-093019-112809

Greenhalgh AD, 2018, PLOS BIOL, V16, DOI 10.1371/journal.pbio.2005264

Groth C, 2019, BRIT J CANCER, V120, P16, DOI 10.1038/s41416-018-0333-1

Guan W, 2019, ENDOCR-RELAT CANCER, V26, P131, DOI 10.1530/ERC-18-0284

Guilliams M, 2014, NAT REV IMMUNOL, V14, P94, DOI 10.1038/nri3582

Gulubova M, 2009, APMIS, V117, P870, DOI 10.1111/j.1600-0463.2009.02547.x

Gunassekaran GR, 2021, BIOMATERIALS, V278, DOI 10.1016/j.biomaterials.2021.121137

Hanahan D, 2022, CANCER DISCOV, V12, P31, DOI 10.1158/2159-8290.CD-21-1059

Haniffa M, 2013, ADV IMMUNOL, V120, P1, DOI 10.1016/B978-0-12-417028-5.00001-6

Haniffa M, 2012, IMMUNITY, V37, P60, DOI 10.1016/j.immuni.2012.04.012

Harari O, 2004, CURR PHARM DESIGN, V10, P893, DOI 10.2174/1381612043452893

HARELBELLAN A, 1986, P NATL ACAD SCI USA, V83, P5688, DOI 10.1073/pnas.83.15.5688

Hartmann E, 2003, CANCER RES, V63, P6478

Hashimoto D, 2013, IMMUNITY, V38, P792, DOI 10.1016/j.immuni.2013.04.004

Heath WR, 2013, NAT IMMUNOL, V14, P978, DOI 10.1038/ni.2680

Heo YJ, 2010, IMMUNOL LETT, V127, P150, DOI 10.1016/j.imlet.2009.10.006

Herzog C, 2019, DEVELOPMENT, V146, DOI 10.1242/dev.174698

Hix LM, 2011, PLOS ONE, V6, DOI 10.1371/journal.pone.0020702

Hodi FS, 2010, NEW ENGL J MED, V363, P711, DOI 10.1056/NEJMoa1003466

Hughes R, 2015, CANCER RES, V75, P3479, DOI 10.1158/0008-5472.CAN-14-3587

Iclozan C, 2013, CANCER IMMUNOL IMMUN, V62, P909, DOI 10.1007/s00262-013-1396-8

Islam R, 2021, CELLS-BASEL, V10, DOI 10.3390/cells10051058

Iyoda T, 2018, CLIN IMMUNOL, V187, P76, DOI 10.1016/j.clim.2017.10.007

Izawa S, 2011, CANCER IMMUNOL IMMUN, V60, P1801, DOI 10.1007/s00262-011-1082-7

Jacquelot N, 2021, ONCOIMMUNOLOGY, V10, DOI 10.1080/2162402X.2021.1900508

Jacquelot N, 2022, NAT IMMUNOL, V23, P371, DOI 10.1038/s41590-022-01127-z

Jacquelot N, 2021, NAT IMMUNOL, V22, P851, DOI 10.1038/s41590-021-00943-z

Jennings MR, 2021, J IMMUNOTHER CANCER, V9, DOI 10.1136/jitc-2021-003013

Jiang FJ, 2018, J CELL MOL MED, V22, P2706, DOI 10.1111/jcmm.13554

Jiang HH, 2015, INT J CANCER, V136, P2352, DOI 10.1002/ijc.29297

Johnson DE, 2018, NAT REV CLIN ONCOL, V15, P234, DOI 10.1038/nrclinonc.2018.8

Jordan VA, 2020, LARYNGOSCOPE, V130, P1496, DOI 10.1002/lary.28277

Jovanovic IP, 2014, INT J CANCER, V134, P1669, DOI 10.1002/ijc.28481

Judge SJ, 2020, J IMMUNOTHER CANCER, V8, DOI 10.1136/jitc-2020-001355

Kalbasi A, 2017, CLIN CANCER RES, V23, P137, DOI 10.1158/1078-0432.CCR-16-0870

Kaneda MM, 2016, CANCER DISCOV, V6, P870, DOI 10.1158/2159-8290.CD-15-1346

Kerdidani D, 2019, NAT COMMUN, V10, DOI 10.1038/s41467-019-09370-z

Kim CW, 2021, BMB REP, V54, P31, DOI 10.5483/BMBRep.2021.54.1.224

Klose CSN, 2014, CELL, V157, P340, DOI 10.1016/j.cell.2014.03.030

Koo KC, 2013, PLOS ONE, V8, DOI 10.1371/journal.pone.0078049

Kranz LM, 2016, NATURE, V534, P396, DOI 10.1038/nature18300

Labidi-Galy SI, 2011, CANCER RES, V71, P5423, DOI 10.1158/0008-5472.CAN-11-0367

Lamichhane P, 2017, CANCER RES, V77, P6667, DOI 10.1158/0008-5472.CAN-17-0740

Lande R, 2007, NATURE, V449, P564, DOI 10.1038/nature06116

Le Mercier I, 2013, CANCER RES, V73, P4629, DOI 10.1158/0008-5472.CAN-12-3058

Lechner MG, 2011, J TRANSL MED, V9, DOI 10.1186/1479-5876-9-90

Lechner MG, 2010, J IMMUNOL, V185, P2273, DOI 10.4049/jimmunol.1000901

Lee JC, 2004, J IMMUNOL, V172, P7335, DOI 10.4049/jimmunol.172.12.7335

Lee PT, 2002, J EXP MED, V195, P637, DOI 10.1084/jem.20011908

Lee-Chang C, 2019, CANCER IMMUNOL RES, V7, P1928, DOI 10.1158/2326-6066.CIR-19-0240

Levings MK, 2005, BLOOD, V105, P1162, DOI 10.1182/blood-2004-03-1211

Levring TB, 2015, ONCOTARGET, V6, P21853, DOI 10.18632/oncotarget.5213

Li G, 2015, MOL MED REP, V12, P760, DOI 10.3892/mmr.2015.3374

Li HYS, 2016, SCI SIGNAL, V9, DOI 10.1126/scisignal.aaf3957

Li HQ, 2009, J IMMUNOL, V182, P240, DOI 10.4049/jimmunol.182.1.240

Li K, 2021, SIGNAL TRANSDUCT TAR, V6, DOI 10.1038/s41392-021-00670-9

Li R, 2017, MEDIAT INFLAMM, V2017, DOI 10.1155/2017/4927964

Li YC, 2020, J IMMUNOL RES, V2020, DOI 10.1155/2020/3948928

Lim AI, 2017, CELL, V168, P1086, DOI 10.1016/j.cell.2017.02.021

Lim HX, 2020, INT J MOL SCI, V21, DOI 10.3390/ijms21103599

Lim SY, 2016, ONCOTARGET, V7, P28697, DOI 10.18632/oncotarget.7376

Liu DF, 2013, CLIN IMMUNOL, V149, P55, DOI 10.1016/j.clim.2013.06.005

Liu HY, 2017, CANCER LETT, V411, P182, DOI 10.1016/j.canlet.2017.09.022

Liu X, 2021, ONCOGENE, V40, P4521, DOI 10.1038/s41388-021-01880-9

Liu Y, 2017, ONCOGENE, V36, P6143, DOI 10.1038/onc.2017.209

Liu YJ, 2005, ANNU REV IMMUNOL, V23, P275, DOI 10.1146/annurev.immunol.23.021704.115633

Liu YH, 2019, EBIOMEDICINE, V41, P333, DOI 10.1016/j.ebiom.2019.02.050

Lo ASY, 2010, CLIN CANCER RES, V16, P2769, DOI 10.1158/1078-0432.CCR-10-0043

Locati M, 2020, ANNU REV PATHOL-MECH, V15, P123, DOI 10.1146/annurev-pathmechdis-012418-012718

Lopes AMM, 2017, ONCOL LETT, V13, P1456, DOI 10.3892/ol.2017.5595

Lorenzo-Herrero S, 2019, CANCERS, V11, DOI 10.3390/cancers11010029

Loyher PL, 2018, J EXP MED, V215, P2536, DOI 10.1084/jem.20180534

Ma TM, 2022, CELLS-BASEL, V11, DOI 10.3390/cells11020310

Mäkitie A, 2021, CURR ONCOL REP, V23, DOI 10.1007/s11912-020-00996-7

Malmberg KJ, 2017, SEMIN IMMUNOL, V31, P20, DOI 10.1016/j.smim.2017.08.002

Mamessier E, 2011, J CLIN INVEST, V121, P3609, DOI 10.1172/JCI45816

Mandruzzato S, 2016, CANCER IMMUNOL IMMUN, V65, P161, DOI 10.1007/s00262-015-1782-5

Mantovani A, 2002, TRENDS IMMUNOL, V23, P549, DOI 10.1016/S1471-4906(02)02302-5

Mantovani S, 2019, HEPATOLOGY, V69, P1165, DOI 10.1002/hep.30235

Mariel GC, 2018, TECHNOL CANCER RES T, V17, DOI 10.1177/1533033818764499

Marsh SGE, 2003, IMMUNOGENETICS, V55, P220, DOI 10.1007/s00251-003-0571-z

Matlack R, 2006, IMMUNOLOGY, V117, P386, DOI 10.1111/j.1365-2567.2005.02312.x

McEwen-Smith RM, 2015, CANCER IMMUNOL RES, V3, P425, DOI 10.1158/2326-6066.CIR-15-0062

McKarns SC, 2004, J IMMUNOL, V172, P4275, DOI 10.4049/jimmunol.172.7.4275

Melo-Gonzalez F, 2017, IMMUNOLOGY, V150, P265, DOI 10.1111/imm.12697

Merad M, 2013, ANNU REV IMMUNOL, V31, P563, DOI 10.1146/annurev-immunol-020711-074950

Metelitsa LS, 2004, J EXP MED, V199, P1213, DOI 10.1084/jem.20031462

Metelitsa LS, 2003, LEUKEMIA, V17, P1068, DOI 10.1038/sj.leu.2402943

Mills CD, 2000, J IMMUNOL, V164, P6166, DOI 10.4049/jimmunol.164.12.6166

Min YF, 2017, ONCOTARGET, V8, P50582, DOI 10.18632/oncotarget.16410

Mjösberg J, 2016, J ALLERGY CLIN IMMUN, V138, P1265, DOI 10.1016/j.jaci.2016.09.009

Mlecnik B, 2014, SCI TRANSL MED, V6, DOI 10.1126/scitranslmed.3007240

Molling JW, 2007, J CLIN ONCOL, V25, P862, DOI 10.1200/JCO.2006.08.5787

Molon B, 2011, J EXP MED, V208, P1949, DOI 10.1084/jem.20101956

Moral JA, 2020, NATURE, V579, P130, DOI 10.1038/s41586-020-2015-4

Moretta A, 2008, CELL DEATH DIFFER, V15, P226, DOI 10.1038/sj.cdd.4402170

Munn DH, 2003, CURR PHARM DESIGN, V9, P257, DOI 10.2174/1381612033392026

Nagaraj S, 2007, NAT MED, V13, P828, DOI 10.1038/nm1609

Najafi M, 2019, J CELL BIOCHEM, V120, P2756, DOI 10.1002/jcb.27646

Chuc AEN, 2012, J CANCER RES CLIN, V138, P1427, DOI 10.1007/s00432-012-1251-x

Nakai R, 2010, CANCER SCI, V101, P1326, DOI 10.1111/j.1349-7006.2010.01530.x

Nakamura K, 2020, CELL MOL IMMUNOL, V17, P1, DOI 10.1038/s41423-019-0306-1

Nielsen SR, 2016, NAT CELL BIOL, V18, P549, DOI 10.1038/ncb3340

Nowak M, 2010, PLOS ONE, V5, DOI 10.1371/journal.pone.0011311

Noy R, 2014, IMMUNITY, V41, P49, DOI 10.1016/j.immuni.2014.06.010

O'Brien SA, 2021, CANCER IMMUNOL IMMUN, V70, P2401, DOI 10.1007/s00262-021-02861-3

Ohl K, 2018, FRONT IMMUNOL, V9, DOI 10.3389/fimmu.2018.02499

Orsini G, 2013, INT J MOL SCI, V14, P22022, DOI 10.3390/ijms141122022

Pachynski RK, 2012, J EXP MED, V209, P1427, DOI 10.1084/jem.20112124

Pasha MA, 2019, ALLERGY ASTHMA PROC, V40, P138, DOI 10.2500/aap.2019.40.4217

Patente TA, 2019, FRONT IMMUNOL, V9, DOI 10.3389/fimmu.2018.03176

Peng LS, 2017, CANCER IMMUNOL RES, V5, P248, DOI 10.1158/2326-6066.CIR-16-0152

Pesce S, 2017, J ALLERGY CLIN IMMUN, V139, P335, DOI 10.1016/j.jaci.2016.04.025

Pesce S, 2015, ONCOIMMUNOLOGY, V4, DOI 10.1080/2162402X.2014.1001224

Pienta KJ, 2013, INVEST NEW DRUG, V31, P760, DOI 10.1007/s10637-012-9869-8

Pietra G, 2012, ONCOIMMUNOLOGY, V1, P974, DOI 10.4161/onci.20405

Platonova S, 2011, CANCER RES, V71, P5412, DOI 10.1158/0008-5472.CAN-10-4179

Poh AR, 2018, FRONT ONCOL, V8, DOI 10.3389/fonc.2018.00049

Pulido AD, 2018, CANCER CELL, V33, P60, DOI 10.1016/j.ccell.2017.11.019

Pyonteck SM, 2013, NAT MED, V19, P1264, DOI 10.1038/nm.3337

Qian BZ, 2009, PLOS ONE, V4, DOI 10.1371/journal.pone.0006562

Quail DF, 2016, SCIENCE, V352, DOI 10.1126/science.aad3018

Quaranta V, 2018, CANCER RES, V78, P4253, DOI 10.1158/0008-5472.CAN-17-3876

Quezada SA, 2006, J CLIN INVEST, V116, P1935, DOI 10.1172/JCI27745

Ramos RN, 2012, J LEUKOCYTE BIOL, V92, P673, DOI 10.1189/jlb.0112048

Rashid MH, 2021, ONCOL REP, V45, P1171, DOI 10.3892/or.2021.7936

Redman JM, 2016, BMC MED, V14, DOI 10.1186/s12916-016-0571-0

REES B, 1990, J MOL BIOL, V214, P281, DOI 10.1016/0022-2836(90)90161-E

Remark R, 2013, CLIN CANCER RES, V19, P4079, DOI 10.1158/1078-0432.CCR-12-3847

Reynolds G, 2015, FRONT IMMUNOL, V6, DOI 10.3389/fimmu.2015.00330

Rezaeifard S, 2021, MOL IMMUNOL, V136, P161, DOI 10.1016/j.molimm.2021.03.003

Rezaeifard S, 2019, IRAN J IMMUNOL, V16, P291, DOI [10.22034/iji.2019.80280, 10.22034/IJI.2019.80280]

Richter J, 2013, BLOOD, V121, P423, DOI 10.1182/blood-2012-06-435503

Roan F, 2019, J CLIN INVEST, V129, P1441, DOI 10.1172/JCI124606

Roberts EW, 2016, CANCER CELL, V30, P324, DOI 10.1016/j.ccell.2016.06.003

Rodell CB, 2018, NAT BIOMED ENG, V2, P578, DOI 10.1038/s41551-018-0236-8

Rodriguez PC, 2007, BLOOD, V109, P1568, DOI 10.1182/blood-2006-06-031856

Rodriguez PC, 2002, J BIOL CHEM, V277, P21123, DOI 10.1074/jbc.M110675200

Romagnani C, 2007, J IMMUNOL, V178, P4947, DOI 10.4049/jimmunol.178.8.4947

Rosser EC, 2015, IMMUNITY, V42, P607, DOI 10.1016/j.immuni.2015.04.005

Sade-Feldman M, 2016, CLIN CANCER RES, V22, P5661, DOI 10.1158/1078-0432.CCR-15-3104

Safarzadeh E, 2019, J CELL PHYSIOL, V234, P3515, DOI 10.1002/jcp.26896

Saito T, 2008, CANCER SCI, V99, P2028, DOI 10.1111/j.1349-7006.2008.00907.x

Salimi M, 2018, BMC CANCER, V18, DOI 10.1186/s12885-018-4262-4

Sallusto F., 2018, J EXP MED 1994, V179, P1109

Salmon H, 2016, IMMUNITY, V44, P924, DOI 10.1016/j.immuni.2016.03.012

Salomé B, 2019, BLOOD ADV, V3, P3674, DOI 10.1182/bloodadvances.2018030478

Sambi M, 2019, J ONCOL, V2019, DOI 10.1155/2019/4508794

Sanchez-Correa B, 2012, IMMUNOL CELL BIOL, V90, P109, DOI 10.1038/icb.2011.15

Sato E, 2005, P NATL ACAD SCI USA, V102, P18538, DOI 10.1073/pnas.0509182102

Schlecker E, 2014, CANCER RES, V74, P3429, DOI 10.1158/0008-5472.CAN-13-3017

Schlecker E, 2012, J IMMUNOL, V189, P5602, DOI 10.4049/jimmunol.1201018

Schlegel NC, 2015, EXP DERMATOL, V24, P22, DOI 10.1111/exd.12580

Schleypen JS, 2006, CLIN CANCER RES, V12, P718, DOI 10.1158/1078-0432.CCR-05-0857

Schneiders FL, 2012, J CLIN ONCOL, V30, P567, DOI 10.1200/JCO.2011.38.8819

Schön MP, 2008, ONCOGENE, V27, P190, DOI 10.1038/sj.onc.1210913

Schott AF, 2017, CLIN CANCER RES, V23, P5358, DOI 10.1158/1078-0432.CCR-16-2748

Sconocchia G, 2014, ONCOIMMUNOLOGY, V3, DOI 10.4161/21624011.2014.952197

Sconocchia G, 2012, CANCER RES, V72, P5428, DOI 10.1158/0008-5472.CAN-12-1181

Sechler JM, 2004, CELL IMMUNOL, V230, P99, DOI 10.1016/j.cellimm.2004.10.001

Segura E, 2012, J EXP MED, V209, P653, DOI 10.1084/jem.20111457

Segura E, 2010, MOL IMMUNOL, V47, P1765, DOI 10.1016/j.molimm.2010.02.028

Semeraro M, 2015, SCI TRANSL MED, V7, DOI 10.1126/scitranslmed.aaa2327

Seo H, 2017, NAT COMMUN, V8, DOI 10.1038/ncomms15776

Shaulov A, 2008, EXP HEMATOL, V36, P464, DOI 10.1016/j.exphem.2007.12.010

Shimizu K, 2020, CANCERS, V12, DOI 10.3390/cancers12040817

Shimizu K, 2013, CANCER RES, V73, P62, DOI 10.1158/0008-5472.CAN-12-0759

Shojaei F, 2009, P NATL ACAD SCI USA, V106, P6742, DOI 10.1073/pnas.0902280106

Shortman K, 2002, NAT REV IMMUNOL, V2, P151, DOI 10.1038/nri746

Sica A, 2012, J CLIN INVEST, V122, P787, DOI 10.1172/JCI59643

Sisirak V, 2012, CANCER RES, V72, P5188, DOI 10.1158/0008-5472.CAN-11-3468

Sivori S, 1996, EUR J IMMUNOL, V26, P2487, DOI 10.1002/eji.1830261032

Sojka DK, 2014, SEMIN IMMUNOL, V26, P127, DOI 10.1016/j.smim.2014.01.010

Spanoudakis E, 2009, BLOOD, V113, P2498, DOI 10.1182/blood-2008-06-161281

Spits H, 2013, NAT REV IMMUNOL, V13, P145, DOI 10.1038/nri3365

Srivastava MK, 2010, CANCER RES, V70, P68, DOI 10.1158/0008-5472.CAN-09-2587

Steinman RM., 2006, NOVART FDN SYMP, V279, P101, DOI [DOI 10.1002/9780470035399, DOI 10.1002/9780470035399.CH9, 10.1002/9780470035399.ch9]

Strachan DC, 2013, ONCOIMMUNOLOGY, V2, DOI 10.4161/onci.26968

Sullivan RJ, 2019, CANCER DISCOV, V9, P694, DOI 10.1158/2159-8290.CD-19-0412

Sun C, 2017, ONCOIMMUNOLOGY, V6, DOI 10.1080/2162402X.2016.1264562

Sun HY, 2019, CANCER IMMUNOL RES, V7, P1535, DOI 10.1158/2326-6066.CIR-18-0757

Syn NL, 2017, LANCET ONCOL, V18, pE731, DOI 10.1016/S1470-2045(17)30607-1

Szabo C, 2007, NAT REV DRUG DISCOV, V6, P662, DOI 10.1038/nrd2222

Tachibana T, 2005, CLIN CANCER RES, V11, P7322, DOI 10.1158/1078-0432.CCR-05-0877

Takahashi R, 2020, SCI REP-UK, V10, DOI 10.1038/s41598-020-78320-3

Terabe M, 2008, ADV CANCER RES, V101, P277, DOI 10.1016/S0065-230X(08)00408-9

Terrén I, 2019, FRONT IMMUNOL, V10, DOI 10.3389/fimmu.2019.02278

Tham M, 2015, ONCOTARGET, V6, P22857, DOI 10.18632/oncotarget.3127

Thommen DS, 2015, CANCER IMMUNOL RES, V3, P1344, DOI 10.1158/2326-6066.CIR-15-0097

Thompson TW, 2017, ELIFE, V6, DOI 10.7554/eLife.30881

Thurner B, 1999, J EXP MED, V190, P1669, DOI 10.1084/jem.190.11.1669

Tian T, 2015, CANCER BIOMARK, V15, P425, DOI 10.3233/CBM-150473

Tobin RP, 2018, INT IMMUNOPHARMACOL, V63, P282, DOI 10.1016/j.intimp.2018.08.007

Toniolo PA, 2016, ONCOTARGET, V7, P46301, DOI 10.18632/oncotarget.10093

Trabanelli S, 2022, EMBO J, V41, DOI 10.15252/embj.2021109300

Trabanelli S, 2018, CYTOM PART B-CLIN CY, V94, P392, DOI 10.1002/cyto.b.21614

Trabanelli S, 2017, NAT COMMUN, V8, DOI 10.1038/s41467-017-00678-2

Trabanelli S, 2015, HAEMATOLOGICA, V100, pE257, DOI 10.3324/haematol.2014.119602

Treilleux I, 2004, CLIN CANCER RES, V10, P7466, DOI 10.1158/1078-0432.CCR-04-0684

Tu MM, 2020, COMMUN BIOL, V3, DOI 10.1038/s42003-020-01441-y

Ubil E, 2018, J CLIN INVEST, V128, P2356, DOI 10.1172/JCI97354

Umansky V, 2001, ADV CANCER RES, V82, P107, DOI 10.1016/S0065-230X(01)82004-2

Umansky V, 2016, VACCINES-BASEL, V4, DOI 10.3390/vaccines4040036

Vachhani P, 2016, ONCOTARGETS THER, V9, P5855, DOI 10.2147/OTT.S97746

van Dalen FJ, 2019, MOLECULES, V24, DOI 10.3390/molecules24010009

Vanoni G, 2021, ELIFE, V10, DOI 10.7554/eLife.58838

Veglia F, 2018, NAT IMMUNOL, V19, P108, DOI 10.1038/s41590-017-0022-x

Versluis MAC, 2017, EUR J CANCER, V86, P285, DOI 10.1016/j.ejca.2017.09.008

Vgenopoulou S, 2003, BREAST, V12, P172, DOI 10.1016/S0960-9776(03)00004-3

Vijayan D, 2017, NAT REV CANCER, V17, P709, DOI 10.1038/nrc.2017.86

Villani AC, 2017, SCIENCE, V356, DOI 10.1126/science.aah4573

Vivier E, 2018, CELL, V174, P1054, DOI 10.1016/j.cell.2018.07.017

Vivier E, 2012, NAT REV IMMUNOL, V12, P239, DOI 10.1038/nri3174

Vivier E, 2011, SCIENCE, V331, DOI 10.1126/science.1198687

Vuk-Pavlovic S, 2010, PROSTATE, V70, P443, DOI 10.1002/pros.21078

Wallin JJ, 2016, NAT COMMUN, V7, DOI 10.1038/ncomms12624

Wanderley CW, 2018, CANCER RES, V78, P5891, DOI 10.1158/0008-5472.CAN-17-3480

Wang WX, 2019, FRONT IMMUNOL, V10, DOI 10.3389/fimmu.2019.01804

Wang XY, 2020, P NATL ACAD SCI USA, V117, P20729, DOI 10.1073/pnas.1915950117

Wang ZN, 2019, FRONT IMMUNOL, V10, DOI 10.3389/fimmu.2019.01114

Wculek SK, 2020, NAT REV IMMUNOL, V20, P7, DOI 10.1038/s41577-019-0210-z

Weil S, 2017, FRONT IMMUNOL, V8, DOI 10.3389/fimmu.2017.00387

Wendel M, 2008, CANCER RES, V68, P8437, DOI 10.1158/0008-5472.CAN-08-1440

Wesolowski R, 2019, THER ADV MED ONCOL, V11, DOI 10.1177/1758835919854238

Wu DY, 2003, J EXP MED, V198, P173, DOI 10.1084/jem.20030446

Wu LY, 2011, AM J PATHOL, V179, P2131, DOI 10.1016/j.ajpath.2011.06.028

Wu Y, 2020, FRONT IMMUNOL, V11, DOI 10.3389/fimmu.2020.554880

Xie JY, 2014, CELL MOL IMMUNOL, V11, P495, DOI 10.1038/cmi.2014.30

Xiu B, 2015, BLOOD CANCER J, V5, DOI 10.1038/bcj.2015.56

Yan WJ, 2015, GUT, V64, P1593, DOI 10.1136/gutjnl-2014-307671

Yanagisawa K, 2002, J IMMUNOL, V168, P6494, DOI 10.4049/jimmunol.168.12.6494

Yang L, 2019, INT J CANCER, V145, P1099, DOI 10.1002/ijc.32151

Yao WB, 2017, EBIOMEDICINE, V22, P58, DOI 10.1016/j.ebiom.2017.07.014

Yeung CLA, 2016, NAT COMMUN, V7, DOI 10.1038/ncomms11150

Yona S, 2013, IMMUNITY, V38, P79, DOI 10.1016/j.immuni.2012.12.001

Zecca A, 2021, CELLS-BASEL, V10, DOI 10.3390/cells10030614

Zhang JC, 2020, MOL CELL PROBE, V50, DOI 10.1016/j.mcp.2019.101498

Zhang N, 2012, NAT IMMUNOL, V13, P667, DOI 10.1038/ni.2319

Zhang Q, 2018, NAT IMMUNOL, V19, P723, DOI 10.1038/s41590-018-0132-0

Zhang QF, 2017, CELL MOL IMMUNOL, V14, P819, DOI 10.1038/cmi.2016.28

Zhang W, 2010, CLIN CANCER RES, V16, P3420, DOI 10.1158/1078-0432.CCR-09-2904

Zhang YX, 2017, ADV EXP MED BIOL, V1024, P225, DOI 10.1007/978-981-10-5987-2_11

Zhen ZJ, 2013, MED ONCOL, V30, DOI 10.1007/s12032-013-0482-2

Zheng MJ, 2016, J IMMUNOL, V196, P4122, DOI 10.4049/jimmunol.1500846

Zheng PM, 2017, J EXP CLIN CANC RES, V36, DOI 10.1186/s13046-017-0528-y

Zhu H, 2017, ONCOTARGET, V8, P114554, DOI 10.18632/oncotarget.23020

Zhu J, 2017, NAT COMMUN, V8, DOI 10.1038/s41467-017-00784-1

Zhu Y, 2017, IMMUNITY, V47, P323, DOI 10.1016/j.immuni.2017.07.014

Zhu Y, 2014, CANCER RES, V74, P5057, DOI 10.1158/0008-5472.CAN-13-3723

Zitvogel L, 2008, NAT REV IMMUNOL, V8, P59, DOI 10.1038/nri2216

NR 352

TC 6

Z9 6

U1 0

U2 15

PU FRONTIERS MEDIA SA

PI LAUSANNE

PA AVENUE DU TRIBUNAL FEDERAL 34, LAUSANNE, CH-1015, SWITZERLAND

SN 2296-634X

J9 FRONT CELL DEV BIOL

JI Front. Cell. Dev. Biol.

PD MAY 26

PY 2022

VL 10

AR 907572

DI 10.3389/fcell.2022.907572

PG 24

WC Cell Biology; Developmental Biology

WE Science Citation Index Expanded (SCI-EXPANDED)

SC Cell Biology; Developmental Biology

GA 2I7HM

UT WOS:000815145100001

PM 35757002

OA Green Published, gold

DA 2025-02-07

ER

PT J

AU Filiberti, S

Russo, M

Lonardi, S

Bugatti, M

Vermi, W

Tournier, C

Giurisato, E

AF Filiberti, Serena

Russo, Mariapia

Lonardi, Silvia

Bugatti, Mattia

Vermi, William

Tournier, Cathy

Giurisato, Emanuele

TI Self-Renewal of Macrophages: Tumor-Released Factors and Signaling

Pathways

SO BIOMEDICINES

LA English

DT Review

DE tumor-associated macrophages; self-renewal; metabolic signature;

signaling pathways; proliferation

ID COLONY-STIMULATING FACTOR; TISSUE-RESIDENT MACROPHAGES; BREAST-CANCER

PROGRESSION; MYELOID CELLS; NITRIC-OXIDE; ISLET INFLAMMATION; LANGERHANS

CELLS; LOCAL PROLIFERATION; M-CSF; METABOLISM

AB Macrophages are the most abundant immune cells of the tumor microenvironment (TME) and have multiple important functions in cancer. During tumor growth, both tissue-resident macrophages and newly recruited monocyte-derived macrophages can give rise to tumor-associated macrophages (TAMs), which have been associated with poor prognosis in most cancers. Compelling evidence indicate that the high degree of plasticity of macrophages and their ability to self-renew majorly impact tumor progression and resistance to therapy. In addition, the microenvironmental factors largely affect the metabolism of macrophages and may have a major influence on TAMs proliferation and subsets functions. Thus, understanding the signaling pathways regulating TAMs self-renewal capacity may help to identify promising targets for the development of novel anticancer agents. In this review, we focus on the environmental factors that promote the capacity of macrophages to self-renew and the molecular mechanisms that govern TAMs proliferation. We also highlight the impact of tumor-derived factors on macrophages metabolism and how distinct metabolic pathways affect macrophage self-renewal.

C1 [Filiberti, Serena; Russo, Mariapia; Giurisato, Emanuele] Univ Siena, Dept Biotechnol Chem & Pharm, I-53100 Siena, Italy.

[Lonardi, Silvia; Bugatti, Mattia; Vermi, William] Univ Brescia, Dept Mol & Translat Med, I-25100 Brescia, Italy.

[Vermi, William] Washington Univ, Dept Pathol & Immunol, Sch Med, St Louis, MO 63130 USA.

[Tournier, Cathy; Giurisato, Emanuele] Univ Manchester, Sch Med Sci, Div Canc Sci, Fac Biol, Manchester M13 9PL, England.

C3 University of Siena; University of Brescia; Washington University

(WUSTL); University of Manchester

RP Giurisato, E (corresponding author), Univ Siena, Dept Biotechnol Chem & Pharm, I-53100 Siena, Italy.; Giurisato, E (corresponding author), Univ Manchester, Sch Med Sci, Div Canc Sci, Fac Biol, Manchester M13 9PL, England.

EM giurisato2@unisi.it

RI Bugatti, Mattia/LIG-8178-2024; GIURISATO, EMANUELE/I-4904-2017

OI Russo, Mariapia/0000-0001-6973-5281; GIURISATO,

EMANUELE/0000-0003-0598-6449; Filiberti, Serena/0000-0001-9795-2638;

LONARDI, Silvia/0000-0003-0307-4163; VERMI, WILLIAM/0000-0002-2291-2997;

Tournier, Cathy/0000-0002-4618-2570

CR Amano SU, 2014, CELL METAB, V19, P162, DOI 10.1016/j.cmet.2013.11.017

Ampem G, 2019, CELL TISSUE RES, V378, P81, DOI 10.1007/s00441-019-03019-5

Andrejeva G, 2017, CELL METAB, V26, P49, DOI 10.1016/j.cmet.2017.06.004

Anfray C, 2020, CELLS-BASEL, V9, DOI 10.3390/cells9010046

AYROLDI E, 1992, BIOTHERAPY, V4, P267, DOI 10.1007/BF02172656

Aziz A, 2009, SCIENCE, V326, P867, DOI 10.1126/science.1176056

Bae S, 2021, CELL REP, V35, DOI 10.1016/j.celrep.2021.109264

Baghdadi M, 2018, SCI REP-UK, V8, DOI 10.1038/s41598-017-18796-8

Baghdadi M, 2016, CANCER RES, V76, P6030, DOI 10.1158/0008-5472.CAN-16-1170

Bailey JD, 2019, CELL REP, V28, P218, DOI 10.1016/j.celrep.2019.06.018

Bain CC, 2016, NAT COMMUN, V7, DOI 10.1038/ncomms11852

Bakopoulos D, 2022, FEBS J, V289, P3735, DOI 10.1111/febs.16364

Baud'Huin M, 2010, J PATHOL, V221, P77, DOI 10.1002/path.2684

Belhareth R., 2015, World J Immunol, V5, P131, DOI DOI 10.5411/WJI.V5.I3.131

Biswas S, 2019, J IMMUNOL, V203, P3447, DOI 10.4049/jimmunol.1900692

Bleriot C, 2020, IMMUNITY, V52, P957, DOI 10.1016/j.immuni.2020.05.014

Blondy T, 2020, J IMMUNOTHER CANCER, V8, DOI 10.1136/jitc-2019-000182

BOTTAZZI B, 1990, J IMMUNOL, V144, P2409

Bowman RL, 2016, CELL REP, V17, P2445, DOI 10.1016/j.celrep.2016.10.052

Boyer S, 2022, ELIFE, V11, DOI [10.7554/eLife.73796, 10.7554/eLife.73796.sa0, 10.7554/eLife.73796.sa1, 10.7554/eLife.73796.sa2]

Braune J, 2017, J IMMUNOL, V198, P2927, DOI 10.4049/jimmunol.1600476

Buchrieser J, 2017, STEM CELL REP, V8, P334, DOI 10.1016/j.stemcr.2016.12.020

Bugler-Lamb A, 2020, CELL, V182, P796, DOI 10.1016/j.cell.2020.07.042

Butcher MJ, 2014, DIABETOLOGIA, V57, P491, DOI 10.1007/s00125-013-3116-5

Byles V, 2013, NAT COMMUN, V4, DOI 10.1038/ncomms3834

Campbell MJ, 2011, BREAST CANCER RES TR, V128, P703, DOI 10.1007/s10549-010-1154-y

Cannarile MA, 2017, J IMMUNOTHER CANCER, V5, DOI 10.1186/s40425-017-0257-y

Caorsi R, 2016, PEDIATR RHEUMATOL, V14, DOI 10.1186/s12969-016-0111-7

Cekic C, 2020, CURR OPIN PHARMACOL, V53, P134, DOI 10.1016/j.coph.2020.08.012

Cella M, 2003, J EXP MED, V198, P645, DOI 10.1084/jem.20022220

Coelho I, 2021, J CLIN MED, V10, DOI 10.3390/jcm10061248

Condeelis J, 2006, CELL, V124, P263, DOI 10.1016/j.cell.2006.01.007

Covarrubias AJ, 2016, ELIFE, V5, DOI 10.7554/eLife.11612

Covarrubias AJ, 2015, SEMIN IMMUNOL, V27, P286, DOI 10.1016/j.smim.2015.08.001

Davies LC, 2013, NAT COMMUN, V4, DOI 10.1038/ncomms2877

de Sousa JR, 2019, INFECT DRUG RESIST, V12, P2589, DOI 10.2147/IDR.S208576

del Barrio IM, 2016, J IMMUNOTHER CANCER, V4, DOI 10.1186/s40425-016-0154-9

Deng WH, 2017, J IMMUNOL, V198, P492, DOI 10.4049/jimmunol.1501845

Ding JX, 2016, INT J ONCOL, V49, P2064, DOI 10.3892/ijo.2016.3680

Donath MY, 2008, DIABETES CARE, V31, pS161, DOI 10.2337/dc08-s243

Draijer C, 2019, J IMMUNOL, V202, P2700, DOI 10.4049/jimmunol.1801387

Eguchi K, 2017, J CLIN INVEST, V127, P14, DOI 10.1172/JCI88877

Ensan S, 2016, NAT IMMUNOL, V17, P159, DOI 10.1038/ni.3343

EVANS R, 1984, J LEUKOCYTE BIOL, V35, P561

Fejer G, 2013, P NATL ACAD SCI USA, V110, pE2191, DOI 10.1073/pnas.1302877110

Franken L, 2016, CELL MICROBIOL, V18, P475, DOI 10.1111/cmi.12580

Franklin RA, 2014, SCIENCE, V344, P921, DOI 10.1126/science.1252510

Ginhoux F, 2006, NAT IMMUNOL, V7, P265, DOI 10.1038/ni1307

Ginhoux F, 2014, NAT REV IMMUNOL, V14, P392, DOI 10.1038/nri3671

Giurisato E, 2020, CANCER RES, V80, P3319, DOI 10.1158/0008-5472.CAN-19-2416

Goossens P, 2019, CELL METAB, V29, P1376, DOI 10.1016/j.cmet.2019.02.016

Greter M, 2012, IMMUNITY, V37, P1050, DOI 10.1016/j.immuni.2012.11.001

Gruessner C, 2014, AM J CANCER RES, V4, P61

Guillonneau C, 2017, CELL MOL LIFE SCI, V74, P2569, DOI 10.1007/s00018-017-2482-4

HAMILTON JA, 1991, BLOOD, V77, P616

Hashimoto D, 2013, IMMUNITY, V38, P792, DOI 10.1016/j.immuni.2013.04.004

He D, 2021, ONCOGENE, V40, P355, DOI 10.1038/s41388-020-01528-0

Heaster TM, 2021, FRONT BIOENG BIOTECH, V9, DOI 10.3389/fbioe.2021.644648

Hu QY, 2019, ARTHRITIS RES THER, V21, DOI 10.1186/s13075-018-1800-z

Huang SCC, 2016, IMMUNITY, V45, P817, DOI 10.1016/j.immuni.2016.09.016

Huang SCC, 2014, NAT IMMUNOL, V15, P846, DOI 10.1038/ni.2956

Hume DA, 2006, CURR OPIN IMMUNOL, V18, P49, DOI 10.1016/j.coi.2005.11.008

Hume DA, 2019, TRENDS IMMUNOL, V40, P98, DOI 10.1016/j.it.2018.11.007

Imperatore F, 2017, EMBO J, V36, P2353, DOI 10.15252/embj.201695737

Jarjour NN, 2019, NAT IMMUNOL, V20, P687, DOI 10.1038/s41590-019-0382-5

Jeannin P, 2018, FEBS J, V285, P680, DOI 10.1111/febs.14343

Jenkins SJ, 2011, SCIENCE, V332, P1284, DOI 10.1126/science.1204351

Jha AK, 2015, IMMUNITY, V42, P419, DOI 10.1016/j.immuni.2015.02.005

Katzenelenbogen Y, 2020, CELL, V182, P872, DOI 10.1016/j.cell.2020.06.032

Kelly B, 2015, CELL RES, V25, P771, DOI 10.1038/cr.2015.68

Kemp SB, 2021, LIFE SCI ALLIANCE, V4, DOI 10.26508/lsa.202000935

Kieler M, 2021, FEBS J, V288, P3694, DOI 10.1111/febs.15715

Kim N, 2020, NAT COMMUN, V11, DOI 10.1038/s41467-020-16164-1

Komohara Y, 2014, CANCER SCI, V105, P1, DOI 10.1111/cas.12314

Kuropkat C, 2003, TUMOR BIOL, V24, P236, DOI 10.1159/000076138

Kutryb-Zajac B, 2021, INT J MOL SCI, V22, DOI 10.3390/ijms22073764

Laviron M, 2019, FRONT IMMUNOL, V10, DOI 10.3389/fimmu.2019.01799

Li HW, 2021, J CANCER, V12, P7111, DOI 10.7150/jca.60379

Lin WY, 2019, FRONT IMMUNOL, V10, DOI 10.3389/fimmu.2019.02019

Linde N, 2018, NAT COMMUN, V9, DOI 10.1038/s41467-017-02481-5

Liu LL, 2016, P NATL ACAD SCI USA, V113, P1564, DOI 10.1073/pnas.1518000113

Liu Y, 2021, BIOMARK RES, V9, DOI 10.1186/s40364-020-00251-y

Locati M, 2020, ANNU REV PATHOL-MECH, V15, P123, DOI 10.1146/annurev-pathmechdis-012418-012718

Lopez-Yrigoyen M, 2021, ANN NY ACAD SCI, V1499, P18, DOI 10.1111/nyas.14377

Loyher PL, 2018, J EXP MED, V215, P2536, DOI 10.1084/jem.20180534

Ma RY, 2022, TRENDS IMMUNOL, V43, P546, DOI 10.1016/j.it.2022.04.008

MacMicking J, 1997, ANNU REV IMMUNOL, V15, P323, DOI 10.1146/annurev.immunol.15.1.323

Mantovani A, 2017, NAT REV CLIN ONCOL, V14, P399, DOI 10.1038/nrclinonc.2016.217

Meiser J, 2016, J BIOL CHEM, V291, P3932, DOI 10.1074/jbc.M115.676817

Mills CD, 2012, CRIT REV IMMUNOL, V32, P463, DOI 10.1615/CritRevImmunol.v32.i6.10

MODOLELL M, 1995, EUR J IMMUNOL, V25, P1101, DOI 10.1002/eji.1830250436

Molgora M, 2020, CELL, V182, P886, DOI 10.1016/j.cell.2020.07.013

Morris SM, 2002, ANNU REV NUTR, V22, P87, DOI 10.1146/annurev.nutr.22.110801.140547

Motta JM, 2021, BIOMEDICINES, V9, DOI 10.3390/biomedicines9101387

Motta JM, 2021, CELL BIOL INT, V45, P890, DOI 10.1002/cbin.11514

Movahedi K, 2010, CANCER RES, V70, P5728, DOI 10.1158/0008-5472.CAN-09-4672

Mroczko B, 2004, CLIN CHEM LAB MED, V42, P256, DOI 10.1515/CCLM.2004.047

Mroczko B, 2003, CLIN CHEM LAB MED, V41, P646, DOI 10.1515/CCLM.2003.098

Müller A, 2015, INT J CANCER, V137, P278, DOI 10.1002/ijc.29379

Mulder K, 2021, IMMUNITY, V54, P1883, DOI 10.1016/j.immuni.2021.07.007

Nasser H, 2020, CELL DEATH DISCOV, V6, DOI 10.1038/s41420-020-00300-3

Ning HF, 2020, INT IMMUNOPHARMACOL, V83, DOI 10.1016/j.intimp.2020.106432

Odegaard JI, 2007, J LEUKOCYTE BIOL, V81, P711, DOI 10.1189/jlb.0906590

Odegaard JI, 2011, ANNU REV PATHOL-MECH, V6, P275, DOI 10.1146/annurev-pathol-011110-130138

Otero K, 2009, NAT IMMUNOL, V10, P734, DOI 10.1038/ni.1744

Painter MM, 2015, MOL NEURODEGENER, V10, DOI 10.1186/s13024-015-0040-9

Pang JB, 2021, J IMMUNOL, V206, P621, DOI 10.4049/jimmunol.2000935

Pathria P, 2019, TRENDS IMMUNOL, V40, P310, DOI 10.1016/j.it.2019.02.003

Pepe G, 2017, SCI REP-UK, V7, DOI 10.1038/srep44270

Pereira M, 2019, CELL REP, V28, P498, DOI 10.1016/j.celrep.2019.06.039

Pixley Fiona J, 2012, Int J Cell Biol, V2012, P501962, DOI 10.1155/2012/501962

Preisser L, 2014, HEPATOLOGY, V60, P1879, DOI 10.1002/hep.27328

Puthenveetil A, 2020, ANN TRANSL MED, V8, DOI 10.21037/atm-20-2037

Pyonteck SM, 2013, NAT MED, V19, P1264, DOI 10.1038/nm.3337

Qian BZ, 2010, CELL, V141, P39, DOI 10.1016/j.cell.2010.03.014

Rauschmeier R, 2019, EMBO J, V38, DOI 10.15252/embj.2018101233

Richardsen E, 2008, HISTOPATHOLOGY, V53, P30, DOI 10.1111/j.1365-2559.2008.03058.x

Richardsen E, 2015, ANTICANCER RES, V35, P865

Richardson ET, 2015, PLOS ONE, V10, DOI 10.1371/journal.pone.0140064

Ries CH, 2014, CANCER CELL, V25, P846, DOI 10.1016/j.ccr.2014.05.016

Roberts J., 2020, MACROPHAGE ACTIVATIO, P1, DOI [10.5772/intechopen.86474, DOI 10.5772/INTECHOPEN.86474]

Röszer T, 2018, CELLS-BASEL, V7, DOI 10.3390/cells7080103

Rosas M, 2014, SCIENCE, V344, P645, DOI 10.1126/science.1251414

Rovida E, 2008, J IMMUNOL, V180, P4166, DOI 10.4049/jimmunol.180.6.4166

Sathe A, 2020, CLIN CANCER RES, V26, P2640, DOI 10.1158/1078-0432.CCR-19-3231

Schulz C, 2012, SCIENCE, V336, P86, DOI 10.1126/science.1219179

Scott CL, 2016, NAT COMMUN, V7, DOI 10.1038/ncomms10321

Sica A, 2008, CANCER LETT, V267, P204, DOI 10.1016/j.canlet.2008.03.028

Sieweke MH, 2013, SCIENCE, V342, P946, DOI 10.1126/science.1242974

Soncin I, 2018, NAT COMMUN, V9, DOI 10.1038/s41467-018-02834-8

Soucie EL, 2016, SCIENCE, V351, DOI 10.1126/science.aad5510

Strakhova R, 2020, PURINERG SIGNAL, V16, P289, DOI 10.1007/s11302-020-09701-6

Takeshita S, 2007, J BIOL CHEM, V282, P18980, DOI 10.1074/jbc.M610938200

Tannahill GM, 2013, NATURE, V496, P238, DOI 10.1038/nature11986

Tardelli M, 2016, MOL METAB, V5, P1131, DOI 10.1016/j.molmet.2016.09.003

Thompson TW, 2018, ELIFE, V7, DOI 10.7554/eLife.32919

Tuong ZK, 2021, CELL REP, V37, DOI 10.1016/j.celrep.2021.110132

Tymoszuk P, 2014, EUR J IMMUNOL, V44, P2247, DOI 10.1002/eji.201344304

Ulland TK, 2018, NAT REV NEUROL, V14, P667, DOI 10.1038/s41582-018-0072-1

Vadiveloo PK, 2001, BBA-MOL CELL RES, V1539, P140, DOI 10.1016/S0167-4889(01)00102-1

Valledor AF, 2000, J BIOL CHEM, V275, P7403, DOI 10.1074/jbc.275.10.7403

Vander Heiden MG, 2017, CELL, V168, DOI 10.1016/j.cell.2016.12.039

VANFURTH R, 1968, J EXP MED, V128, P415, DOI 10.1084/jem.128.3.415

Vats D, 2006, CELL METAB, V4, P13, DOI 10.1016/j.cmet.2006.05.011

Viola A, 2019, FRONT IMMUNOL, V10, DOI 10.3389/fimmu.2019.01462

Wang B, 2015, INT J MOL MED, V35, P92, DOI 10.3892/ijmm.2014.2001

Wang FL, 2018, CELL METAB, V28, P463, DOI 10.1016/j.cmet.2018.08.012

Wang HY, 2016, ONCOIMMUNOLOGY, V5, DOI 10.1080/2162402X.2015.1122157

Wang JF, 2021, J HEPATOL, V74, P627, DOI 10.1016/j.jhep.2020.10.021

Wang QQ, 2021, FRONT IMMUNOL, V12, DOI 10.3389/fimmu.2021.629281

Wang RN, 2011, IMMUNITY, V35, P871, DOI 10.1016/j.immuni.2011.09.021

Wang SY, 2019, CANCER LETT, V452, P14, DOI 10.1016/j.canlet.2019.03.015

Wang SH, 2022, MOL THER ONCOLYTICS, V24, P799, DOI 10.1016/j.omto.2022.02.019

Wang T, 2017, MEDIAT INFLAMM, V2017, DOI 10.1155/2017/9029327

Wang YM, 2012, NAT IMMUNOL, V13, P753, DOI 10.1038/ni.2360

Wang Y, 2022, J HEMATOL ONCOL, V15, DOI 10.1186/s13045-022-01335-y

Watanabe S, 2019, J CLIN INVEST, V129, P2619, DOI 10.1172/JCI124615

Wenes M, 2016, CELL METAB, V24, P701, DOI 10.1016/j.cmet.2016.09.008

Wessendarp M, 2022, MITOCHONDRION, V62, P85, DOI 10.1016/j.mito.2021.10.009

Wu K, 2015, J EXP MED, V212, P681, DOI 10.1084/jem.20141732

Yang HL, 2020, AM J REPROD IMMUNOL, V84, DOI 10.1111/aji.13261

Yang YS, 2020, TRENDS PHARMACOL SCI, V41, P701, DOI 10.1016/j.tips.2020.08.003

Ying W, 2020, NAT REV ENDOCRINOL, V16, P81, DOI 10.1038/s41574-019-0286-3

Ying W, 2019, CELL METAB, V29, P457, DOI 10.1016/j.cmet.2018.12.003

Yona S, 2013, IMMUNITY, V38, P79, DOI 10.1016/j.immuni.2012.12.001

Zavialov AV, 2010, J LEUKOCYTE BIOL, V88, P279, DOI 10.1189/jlb.1109764

Zhang HY, 2022, CANCER IMMUNOL IMMUN, V71, P2511, DOI 10.1007/s00262-022-03173-w

Zhang QQ, 2021, NAT COMMUN, V12, DOI 10.1038/s41467-021-27018-9

Zhao HY, 2021, J TRANSL MED, V19, DOI 10.1186/s12967-021-03123-7

Zheng C, 2016, CELL DEATH DIS, V7, DOI 10.1038/cddis.2016.54

Zhong L, 2017, J EXP MED, V214, P597, DOI 10.1084/jem.20160844

Zhou KW, 2020, ONCOL LETT, V20, DOI 10.3892/ol.2020.12097

Zhu BB, 2021, IMMUNITY, V54, P1200, DOI 10.1016/j.immuni.2021.04.001

Zhu Y, 2017, IMMUNITY, V47, P323, DOI 10.1016/j.immuni.2017.07.014

Zhu Y, 2014, CANCER RES, V74, P5057, DOI 10.1158/0008-5472.CAN-13-3723

Zilionis R, 2019, IMMUNITY, V50, P1317, DOI 10.1016/j.immuni.2019.03.009

Zou W, 2008, MOL CELL, V31, P422, DOI 10.1016/j.molcel.2008.06.023

NR 177

TC 12

Z9 13

U1 1

U2 15

PU MDPI

PI BASEL

PA ST ALBAN-ANLAGE 66, CH-4052 BASEL, SWITZERLAND

EI 2227-9059

J9 BIOMEDICINES

JI Biomedicines

PD NOV

PY 2022

VL 10

IS 11

AR 2709

DI 10.3390/biomedicines10112709

PG 20

WC Biochemistry & Molecular Biology; Medicine, Research & Experimental;

Pharmacology & Pharmacy

WE Science Citation Index Expanded (SCI-EXPANDED)

SC Biochemistry & Molecular Biology; Research & Experimental Medicine;

Pharmacology & Pharmacy

GA 6B0EK

UT WOS:000881017300001

PM 36359228

OA Green Published, gold

DA 2025-02-07

ER

PT J

AU Kramer, ED

Tzetzo, SL

Colligan, SH

Hensen, ML

Brackett, CM

Clausen, BE

Taketo, MM

Abrams, SI

AF Kramer, Elliot D.

Tzetzo, Stephanie L.

Colligan, Sean H.

Hensen, Mary L.

Brackett, Craig M.

Clausen, Bjoern E.

Taketo, Makoto M.

Abrams, Scott I.

TI ?-Catenin signaling in alveolar macrophages enhances lung metastasis a

mechanism

SO JCI INSIGHT

LA English

DT Article

ID TUMOR-NECROSIS-FACTOR; CANCER; CELLS; INFLAMMATION; RESISTANCE;

MONOCYTES; ONTOGENY; RENEWAL

AB The main cause of malignancy-related mortality is metastasis. Although metastatic progression is driven by diverse tumor-intrinsic mechanisms, there is a growing appreciation for the contribution of tumor-extrinsic elements of the tumor microenvironment, especially macrophages, which correlate with poor clinical outcomes. Macrophages consist of bone marrow-derived and tissue-resident populations. In contrast to bone marrow-derived macrophages, the transcriptional pathways that govern the pro-metastatic activities of tissue-resident macrophages (TRMs) remain less clear. Alveolar macrophages (AMs) are a TRM population with critical roles in tissue homeostasis and metastasis. Wnt/beta-catenin signaling is a hallmark of cancer and has been identified as a pathologic regulator of AMs in infection. We tested the hypothesis that beta-catenin expression in AMs enhances metastasis in solid tumor models. Using a genetic beta-catenin gain-of-function approach, we demonstrated that (a) enhanced beta-catenin in AMs heightened lung metastasis; (b) beta-catenin activity in AMs drove a dysregulated inflammatory program strongly associated with Tnf expression; and (c) localized TNF-alpha blockade abrogated this metastatic outcome. Last, beta-catenin gene CTNNB1 and TNF expression levels were positively correlated in AMs of patients with lung cancer. Overall, our findings revealed a Wnt/beta-catenin/TNF-alpha pro-metastatic axis in AMs with potential therapeutic implications against tumors refractory to the antineoplastic actions of TNF-alpha.

C1 [Kramer, Elliot D.; Tzetzo, Stephanie L.; Colligan, Sean H.; Hensen, Mary L.; Taketo, Makoto M.; Abrams, Scott I.] Roswell Pk Comprehens Canc Ctr, Dept Immunol, Buffalo, NY USA.

[Brackett, Craig M.] Roswell Pk Comprehens Canc Ctr, Dept Cell Stress Biol, Buffalo, NY USA.

[Clausen, Bjoern E.] Johannes Gutenberg Univ Mainz, Univ Med Ctr Johannes Gutenberg, Inst Mol Med, Paul Klein Ctr Immune Intervent, Mainz, Germany.

[Taketo, Makoto M.] Kyoto Univ, Grad Sch Med, Div Expt Therapeut, Kyoto, Japan.

[Abrams, Scott I.] Roswell Pk Comprehens Canc Ctr, Dept Immunol, Elm & Carlton St, Buffalo, NY 14263 USA.

C3 Roswell Park Comprehensive Cancer Center; Roswell Park Comprehensive

Cancer Center; Johannes Gutenberg University of Mainz; Kyoto University;

Roswell Park Comprehensive Cancer Center

RP Abrams, SI (corresponding author), Roswell Pk Comprehens Canc Ctr, Dept Immunol, Elm & Carlton St, Buffalo, NY 14263 USA.

EM scott.abrams@roswellpark.org

OI Kramer, Elliot/0000-0002-2749-8158

FU Flow and Image Cytometry Shared Resource; NIH National Cancer Institute

Cancer Center [P30CA016056]; National Cancer Institute/NIH; Roswell Park

Alliance Foundation; Sklarow Memorial Trust; NIH Institutional National

Research Service Award Training Grant [F31CA243304]; NIH;

[T32CA085183]; [1S10OD025183]; [F30CA254327]; [F31CA228396];

[R01CA172105]; [R01CA250412]

FX We gratefully thank Vivek Mittal (Weill Medical College at Cornell

University, New York, New York, USA) and Pamela Hershberger (Roswell

Park, Buffalo, New York, USA) for providing the E0771.ML-1 and LLC-luc

cell lines, respectively. We also thank Sharon Evans, Brahm Segal, and

Dominic Smiraglia (Roswell Park, Buffalo, New York, USA) for their

advice and critical review of this work. We thank Justine Jacobi

(Roswell Park, Buffalo, New York, USA) for technical support related to

the CUT&RUN experiments. Model figures were created with BioRender.com.

The results published here are in whole or part based upon data

generated by the TCGA Research Network: https:// www.cancer.gov/tcga.

Services and resources were provided by the Flow and Image Cytometry

Shared Resource, the Translational Imaging Shared Resource, and the

Genomics Shared Resource at Roswell Park. All Roswell Park shared

resources were supported through NIH National Cancer Institute Cancer

Center Support Grant P30CA016056, and the Sony MA900 sorter was funded

through NIH Shared Instrument Grant 1S10OD025183. This work was

supported by R01CA172105 and R01CA250412 from the National Cancer

Institute/NIH (to SIA) ; the Roswell Park Alliance Foundation (to SIA) ;

the Sklarow Memorial Trust (to SIA) ; NIH Institutional National

Research Service Award Training Grant T32CA085183 (to EDK, SLT, and SHC)

; and NIH fellowship grants F30CA254327, F31CA243304, and F31CA228396 to

EDK, SLT, and SHC, respectively.

CR Abram CL, 2014, J IMMUNOL METHODS, V408, P89, DOI 10.1016/j.jim.2014.05.009

Acharyya S, 2012, CELL, V150, P165, DOI 10.1016/j.cell.2012.04.042

Baghba R, 2020, CELL COMMUN SIGNAL, V18, DOI 10.1186/s12964-020-0530-4

Bertrand F, 2017, NAT COMMUN, V8, DOI 10.1038/s41467-017-02358-7

Bertrand F, 2015, CANCER RES, V75, P2619, DOI 10.1158/0008-5472.CAN-14-2524

Bowman RL, 2016, CELL REP, V17, P2445, DOI 10.1016/j.celrep.2016.10.052

Burkard-Mandel L, 2018, ONCOIMMUNOLOGY, V7, DOI 10.1080/2162402X.2017.1419115

Cadigan KM, 1997, GENE DEV, V11, P3286, DOI 10.1101/gad.11.24.3286

CARSWELL EA, 1975, P NATL ACAD SCI USA, V72, P3666, DOI 10.1073/pnas.72.9.3666

Casanova-Acebes M, 2021, NATURE, V595, P578, DOI 10.1038/s41586-021-03651-8

Chen MT, 2017, SCI REP-UK, V7, DOI 10.1038/s41598-017-10166-8

Clausen BE, 1999, TRANSGENIC RES, V8, P265, DOI 10.1023/A:1008942828960

Clevers H, 2006, CELL, V127, P469, DOI 10.1016/j.cell.2006.10.018

Cotechini T, 2021, CELLS-BASEL, V10, DOI 10.3390/cells10040960

DeNardo DG, 2019, NAT REV IMMUNOL, V19, P369, DOI 10.1038/s41577-019-0127-6

Desai TJ, 2014, NATURE, V507, P190, DOI 10.1038/nature12930

Dick SA, 2022, SCI IMMUNOL, V7, DOI 10.1126/sciimmunol.abf7777

Dillekås H, 2019, CANCER MED-US, V8, P5574, DOI 10.1002/cam4.2474

El Rayes T, 2015, P NATL ACAD SCI USA, V112, P16000, DOI 10.1073/pnas.1507294112

Fejer G, 2013, P NATL ACAD SCI USA, V110, pE2191, DOI 10.1073/pnas.1302877110

Feng Y, 2018, KIDNEY DIS-BASEL, V4, P95, DOI 10.1159/000488984

Feng YX, 2018, GENES DIS, V5, P77, DOI 10.1016/j.gendis.2018.05.001

FIDLER IJ, 1976, CANCER RES, V36, P3160

Gentles AJ, 2015, NAT MED, V21, P938, DOI 10.1038/nm.3909

Ginhoux F, 2016, IMMUNITY, V44, P439, DOI 10.1016/j.immuni.2016.02.024

Greten FR, 2019, IMMUNITY, V51, P27, DOI 10.1016/j.immuni.2019.06.025

Guilliams M, 2013, J EXP MED, V210, P1977, DOI 10.1084/jem.20131199

Harada N, 1999, EMBO J, V18, P5931, DOI 10.1093/emboj/18.21.5931

Hume DA, 2011, J LEUKOCYTE BIOL, V89, P525, DOI 10.1189/jlb.0810472

Jho EH, 2002, MOL CELL BIOL, V22, P1172, DOI 10.1128/MCB.22.4.1172-1183.2002

Kodama T, 2020, LAB INVEST, V100, P1140, DOI 10.1038/s41374-020-0441-4

Kohli K, 2022, CANCER GENE THER, V29, P10, DOI 10.1038/s41417-021-00303-x

Kramer ED, 2020, FRONT IMMUNOL, V11, DOI 10.3389/fimmu.2020.01963

Krombach F, 1997, ENVIRON HEALTH PERSP, V105, P1261, DOI 10.2307/3433544

Lambert AW, 2017, CELL, V168, P670, DOI 10.1016/j.cell.2016.11.037

Laviron M, 2019, FRONT IMMUNOL, V10, DOI 10.3389/fimmu.2019.01799

Li B, 2009, CANCER RES, V69, P338, DOI 10.1158/0008-5472.CAN-08-1565

Li PS, 2020, NAT IMMUNOL, V21, P1444, DOI 10.1038/s41590-020-0783-5

Liao SJ, 2019, J CELL COMMUN SIGNAL, V13, P369, DOI 10.1007/s12079-019-00508-8

Loyher PL, 2018, J EXP MED, V215, P2536, DOI 10.1084/jem.20180534

Lu ZH, 2020, NATURE, V579, P284, DOI 10.1038/s41586-020-2054-x

Madisen L, 2010, NAT NEUROSCI, V13, P133, DOI 10.1038/nn.2467

Mantovani A, 2004, TRENDS IMMUNOL, V25, P677, DOI 10.1016/j.it.2004.09.015

Mantovani A, 2002, TRENDS IMMUNOL, V23, P549, DOI 10.1016/S1471-4906(02)02302-5

Mercer PF, 2014, AM J RESP CELL MOL, V50, P144, DOI 10.1165/rcmb.2013-0142OC

Montfort A, 2019, FRONT IMMUNOL, V10, DOI 10.3389/fimmu.2019.01818

Mosimann C, 2009, NAT REV MOL CELL BIO, V10, P276, DOI 10.1038/nrm2654

Paget S., LANCET, V1, P571, DOI [10.1016/s0140-6736(00)49915-0, DOI 10.1016/S0140-6736(00)49915-0]

Qian BZ, 2010, CELL, V141, P39, DOI 10.1016/j.cell.2010.03.014

Roskoski R, 2019, PHARMACOL RES, V144, P19, DOI 10.1016/j.phrs.2019.03.006

Sarode P, 2020, SCI ADV, V6, DOI 10.1126/sciadv.aaz6105

Sarode P, 2017, EUR RESPIR J, V50, DOI 10.1183/1393003.congress-2017.OA4859

Sheng JP, 2015, IMMUNITY, V43, P382, DOI 10.1016/j.immuni.2015.07.016

Sorensen MR, 2014, PLOS ONE, V9, DOI 10.1371/journal.pone.0087831

Spiegel A, 2016, CANCER DISCOV, V6, P630, DOI 10.1158/2159-8290.CD-15-1157

Stathis A, 2018, CANCER DISCOV, V8, P24, DOI 10.1158/2159-8290.CD-17-0605

Timperi E, 2020, J CLIN ONCOL, V38

Travaglini KJ, 2020, NATURE, V587, DOI 10.1038/s41586-020-2922-4

Twum DYF, 2019, JCI INSIGHT, V4, DOI 10.1172/jci.insight.124267

Tzetzo SL, 2021, CANCER CELL, V39, P734, DOI 10.1016/j.ccell.2021.03.002

Valenta T, 2012, EMBO J, V31, P2714, DOI 10.1038/emboj.2012.150

Waarts MR, 2022, J CLIN INVEST, V132, DOI 10.1172/JCI154943

Waight JD, 2013, J CLIN INVEST, V123, P4464, DOI 10.1172/JCI68189

Wang X, 2008, ACTA PHARMACOL SIN, V29, P1275, DOI 10.1111/j.1745-7254.2008.00889.x

Wculek SK, 2015, NATURE, V528, P413, DOI 10.1038/nature16140

Welch DR, 2019, CANCER RES, V79, P3011, DOI 10.1158/0008-5472.CAN-19-0458

White BD, 2012, GASTROENTEROLOGY, V142, P219, DOI 10.1053/j.gastro.2011.12.001

Williams CB, 2016, NPJ BREAST CANCER, V2, DOI 10.1038/npjbcancer.2015.25

Wu S, 2018, NAT COMMUN, V9, DOI 10.1038/s41467-018-05467-z

Yan HYH, 2015, CANCER RES, V75, P5283, DOI 10.1158/0008-5472.CAN-15-2282-T

Yang CC, 2019, INT J ONCOL, V55, P684, DOI 10.3892/ijo.2019.4840

Yang Y, 2018, CELL DEATH DIS, V9, DOI 10.1038/s41419-018-0818-0

Yeo EJ, 2014, CANCER RES, V74, P2962, DOI 10.1158/0008-5472.CAN-13-2421

Yu YRA, 2016, PLOS ONE, V11, DOI 10.1371/journal.pone.0150606

Zbytek Blazej, 2008, Expert Rev Dermatol, V3, P569, DOI 10.1586/17469872.3.5.569

Zhan T, 2017, ONCOGENE, V36, P1461, DOI 10.1038/onc.2016.304

Zhou YY, 2019, NAT COMMUN, V10, DOI 10.1038/s41467-019-09234-6

Zhu BB, 2021, IMMUNITY, V54, P1200, DOI 10.1016/j.immuni.2021.04.001

NR 78

TC 16

Z9 16

U1 0

U2 5

PU AMER SOC CLINICAL INVESTIGATION INC

PI ANN ARBOR

PA 2015 MANCHESTER RD, ANN ARBOR, MI 48104 USA

EI 2379-3708

J9 JCI INSIGHT

JI JCI Insight

PD APR 24

PY 2023

VL 8

IS 8

AR e160978

DI 10.1172/jci.insight.160978

PG 18

WC Medicine, Research & Experimental

WE Science Citation Index Expanded (SCI-EXPANDED)

SC Research & Experimental Medicine

GA E8VU8

UT WOS:000978262600001

PM 37092550

OA Green Published, gold

DA 2025-02-07

ER

PT J

AU Domoto, R

Sekiguchi, F

Tsubota, M

Kawabata, A

AF Domoto, Risa

Sekiguchi, Fumiko

Tsubota, Maho

Kawabata, Atsufumi

TI Macrophage as a Peripheral Pain Regulator

SO CELLS

LA English

DT Review

DE macrophage; neuroimmune crosstalk; neuropathic pain; visceral pain;

inflammatory pain; dorsal root ganglion; primary afferent

ID GROUP BOX 1; TISSUE-RESIDENT MACROPHAGES; SULFIDE-FORMING ENZYME;

DORSAL-ROOT GANGLIA; HYDROGEN-SULFIDE; BLADDER PAIN; CALCIUM-CHANNELS;

SENSORY NEURONS; UP-REGULATION; NEUROPATHIC PAIN

AB A neuroimmune crosstalk is involved in somatic and visceral pathological pain including inflammatory and neuropathic components. Apart from microglia essential for spinal and supraspinal pain processing, the interaction of bone marrow-derived infiltrating macrophages and/or tissue-resident macrophages with the primary afferent neurons regulates pain signals in the peripheral tissue. Recent studies have uncovered previously unknown characteristics of tissue-resident macrophages, such as their origins and association with regulation of pain signals. Peripheral nerve macrophages and intestinal resident macrophages, in addition to adult monocyte-derived infiltrating macrophages, secrete a variety of mediators, such as tumor necrosis factor-alpha, interleukin (IL)-1 beta, IL-6, high mobility group box 1 and bone morphogenic protein 2 (BMP2), that regulate the excitability of the primary afferents. Neuron-derived mediators including neuropeptides, ATP and macrophage-colony stimulating factor regulate the activity or polarization of diverse macrophages. Thus, macrophages have multitasks in homeostatic conditions and participate in somatic and visceral pathological pain by interacting with neurons.

C1 [Domoto, Risa; Sekiguchi, Fumiko; Tsubota, Maho; Kawabata, Atsufumi] Kindai Univ, Fac Pharm, Lab Pharmacol & Pathophysiol, Higashiosaka 5778502, Japan.

C3 Kindai University (Kinki University)

RP Kawabata, A (corresponding author), Kindai Univ, Fac Pharm, Lab Pharmacol & Pathophysiol, Higashiosaka 5778502, Japan.

EM risa.domoto@gmail.com; fumiko@phar.kindai.ac.jp; maho@phar.kindai.ac.jp;

kawabata@phar.kindai.ac.jp

OI Kawabata, Atsufumi/0000-0002-3163-807X

CR Agalave NM, 2014, MOL MED, V20, P569, DOI 10.2119/molmed.2014.00176

Alliot F, 1999, DEV BRAIN RES, V117, P145, DOI 10.1016/S0165-3806(99)00113-3

Silva CEA, 2021, PAIN REP, V6, DOI 10.1097/PR9.0000000000000873

Badiei A, 2013, APPL MICROBIOL BIOT, V97, P7845, DOI 10.1007/s00253-013-5080-x

Baliu-Piqué M, 2014, EUR J IMMUNOL, V44, P3708, DOI 10.1002/eji.201444553

Baral P, 2019, NAT REV IMMUNOL, V19, P433, DOI 10.1038/s41577-019-0147-2

Baum P, 2016, METABOLISM, V65, P391, DOI 10.1016/j.metabol.2015.11.002

Bele T, 2016, J NEUROCHEM, V138, P587, DOI 10.1111/jnc.13680

Binshtok AM, 2008, J NEUROSCI, V28, P14062, DOI 10.1523/JNEUROSCI.3795-08.2008

Chen O, 2020, CURR OPIN NEUROBIOL, V62, P17, DOI 10.1016/j.conb.2019.11.006

Chen PW, 2015, ACTA NEUROPATHOL, V130, P605, DOI 10.1007/s00401-015-1482-4

Choi SC, 2003, CANCER INVEST, V21, P708, DOI 10.1081/CNV-120023764

Cook AD, 2018, TRENDS IMMUNOL, V39, P240, DOI 10.1016/j.it.2017.12.003

Costigan M, 2009, ANNU REV NEUROSCI, V32, P1, DOI 10.1146/annurev.neuro.051508.135531

Dai Y, 2007, J CLIN INVEST, V117, P1979, DOI 10.1172/JCI30951

Davies LC, 2013, NAT IMMUNOL, V14, P986, DOI 10.1038/ni.2705

Di Virgilio F, 2017, IMMUNITY, V47, P15, DOI 10.1016/j.immuni.2017.06.020

Dobberfuhl AD, 2021, INT UROGYNECOL J, V32, P3105, DOI 10.1007/s00192-021-04878-9

Donnelly CR, 2020, NEUROTHERAPEUTICS, V17, P846, DOI 10.1007/s13311-020-00905-7

Ebbinghaus M, 2012, ARTHRITIS RHEUM-US, V64, P3897, DOI 10.1002/art.34675

Ebersberger A, 2018, EUR J PHARMACOL, V835, P19, DOI 10.1016/j.ejphar.2018.07.040

Forster R, 2019, FASEB J, V33, P11210, DOI 10.1096/fj.201900797R

Gaudet AD, 2016, J NEUROSCI, V36, P8516, DOI 10.1523/JNEUROSCI.0735-16.2016

Geraghty T, 2021, PAIN REP, V6, DOI 10.1097/PR9.0000000000000892

Ginhoux F, 2016, IMMUNITY, V44, P439, DOI 10.1016/j.immuni.2016.02.024

Godinho-Silva C, 2019, ANNU REV IMMUNOL, V37, P19, DOI 10.1146/annurev-immunol-042718-041812

Grubisic V, 2020, CELL REP, V32, DOI 10.1016/j.celrep.2020.108100

Gu HB, 2020, INT J CANCER, V146, P2810, DOI 10.1002/ijc.32652

Hiramoto S, 2020, CELLS-BASEL, V9, DOI 10.3390/cells9081748

Hoeffel G, 2018, CELL IMMUNOL, V330, P5, DOI 10.1016/j.cellimm.2018.01.001

Hoeffel G, 2015, IMMUNITY, V42, P665, DOI 10.1016/j.immuni.2015.03.011

Hong HS, 2014, BIOCHEM BIOPH RES CO, V453, P179, DOI 10.1016/j.bbrc.2014.09.090

Huang ZZ, 2014, BRAIN BEHAV IMMUN, V40, P155, DOI 10.1016/j.bbi.2014.03.014

Hylands-White N, 2017, RHEUMATOL INT, V37, P29, DOI 10.1007/s00296-016-3481-8

Inoue K, 2021, BIOCHEM PHARMACOL, V187, DOI 10.1016/j.bcp.2020.114309

Irie Y, 2017, J NEUROIMMUNE PHARM, V12, P693, DOI 10.1007/s11481-017-9757-2

Ji RR, 2014, NAT REV DRUG DISCOV, V13, P533, DOI 10.1038/nrd4334

Jia M, 2017, MOL PAIN, V13, DOI 10.1177/1744806917719804

Jin XC, 2006, J NEUROSCI, V26, P246, DOI 10.1523/JNEUROSCI.3858-05.2006

Junger WG, 2011, NAT REV IMMUNOL, V11, P201, DOI 10.1038/nri2938

Kawabata A, 2007, PAIN, V132, P74, DOI 10.1016/j.pain.2007.01.026

Kawabata A, 2011, BIOL PHARM BULL, V34, P1170, DOI 10.1248/bpb.34.1170

Kerr BJ, 2001, NEUROREPORT, V12, P3077, DOI 10.1097/00001756-200110080-00019

Kiguchi N, 2015, PAIN, V156, P684, DOI 10.1097/j.pain.0000000000000097

Kogan P, 2018, SCI REP-UK, V8, DOI 10.1038/s41598-018-24833-x

Kohno K, 2021, PAIN REP, V6, DOI 10.1097/PR9.0000000000000864

Kolter J, 2020, J IMMUNOL, V204, P271, DOI 10.4049/jimmunol.1901077

Kotaka M, 2020, CANCER CHEMOTH PHARM, V86, P607, DOI 10.1007/s00280-020-04135-8

Kulkarni S, 2018, J NEUROSCI, V38, P9346, DOI 10.1523/JNEUROSCI.1663-18.2018

Kwon MJ, 2015, J NEUROSCI, V35, P15934, DOI 10.1523/JNEUROSCI.1924-15.2015

Kwon MJ, 2013, J NEUROSCI, V33, P15095, DOI 10.1523/JNEUROSCI.0278-13.2013

Labuz D, 2021, J NEUROSCI, V41, P2870, DOI 10.1523/JNEUROSCI.3040-20.2021

Latremoliere A, 2009, J PAIN, V10, P895, DOI 10.1016/j.jpain.2009.06.012

Lee JH, 2017, FRONT PHARMACOL, V8, DOI 10.3389/fphar.2017.00839

Li Y, 2017, PAIN, V158, P417, DOI 10.1097/j.pain.0000000000000774

Lim JE, 2017, SCI REP-UK, V7, DOI 10.1038/s41598-017-09639-7

Liu QY, 2019, EXP NEUROL, V317, P226, DOI 10.1016/j.expneurol.2019.03.005

Lucrezi JD, 2014, INT IMMUNOPHARMACOL, V21, P44, DOI 10.1016/j.intimp.2014.04.007

Luo X, 2019, J NEUROSCI, V39, P6848, DOI 10.1523/JNEUROSCI.3257-18.2019

Ma WY, 2012, EXPERT OPIN THER TAR, V16, P527, DOI 10.1517/14728222.2012.680955

Maeda Y, 2009, PAIN, V142, P127, DOI 10.1016/j.pain.2008.12.021

Malsch P, 2014, J NEUROSCI, V34, P9845, DOI 10.1523/JNEUROSCI.5161-13.2014

Martins JP, 2012, BRIT J PHARMACOL, V165, P183, DOI 10.1111/j.1476-5381.2011.01535.x

Matsui K, 2019, J PHARMACOL SCI, V140, P310, DOI 10.1016/j.jphs.2019.07.010

Matsunami M, 2009, GUT, V58, P751, DOI 10.1136/gut.2007.144543

Matsunami M, 2012, BRIT J PHARMACOL, V167, P917, DOI 10.1111/j.1476-5381.2012.02060.x

Meshkani R, 2016, CLIN CHIM ACTA, V462, P77, DOI 10.1016/j.cca.2016.08.015

Miki T, 2011, PAIN, V152, P1373, DOI 10.1016/j.pain.2011.02.019

Muller PA, 2014, CELL, V158, P300, DOI 10.1016/j.cell.2014.04.050

Nishida T, 2016, TOXICOLOGY, V365, P48, DOI 10.1016/j.tox.2016.07.016

Nishimura S, 2009, GUT, V58, P762, DOI 10.1136/gut.2008.151910

Okubo K, 2011, NEUROSCIENCE, V188, P148, DOI 10.1016/j.neuroscience.2011.05.004

Okubo K, 2012, BRIT J PHARMACOL, V166, P1738, DOI 10.1111/j.1476-5381.2012.01886.x

Oliveira-Fusaro MC, 2020, MOL NEUROBIOL, V57, P1917, DOI 10.1007/s12035-019-01852-x

Ozaki T, 2018, CLIN EXP PHARMACOL P, V45, P355, DOI 10.1111/1440-1681.12875

Ozaki T, 2018, TOXICOLOGY, V393, P102, DOI 10.1016/j.tox.2017.11.012

Palis J, 2016, FEBS LETT, V590, P3965, DOI 10.1002/1873-3468.12459

Panicker JN, 2019, NEUROUROL URODYNAM, V38, pS25, DOI 10.1002/nau.24111

Peters CM, 2007, BRAIN RES, V1168, P46, DOI 10.1016/j.brainres.2007.06.066

Pinho-Ribeiro FA, 2017, TRENDS IMMUNOL, V38, P5, DOI 10.1016/j.it.2016.10.001

Qiao LYY, 2020, AM J PHYSIOL-GASTR L, V319, pG748, DOI 10.1152/ajpgi.00323.2020

Rudjito R, 2021, PAIN, V162, P459, DOI 10.1097/j.pain.0000000000002034

Saika F, 2019, J PHARMACOL EXP THER, V368, P535, DOI 10.1124/jpet.118.252668

Sekiguchi F, 2021, INT J MOL SCI, V22, DOI 10.3390/ijms22010367

Sekiguchi F, 2018, NEUROPHARMACOLOGY, V141, P201, DOI 10.1016/j.neuropharm.2018.08.040

Sekiguchi F, 2018, BIOL PHARM BULL, V41, P1127, DOI 10.1248/bpb.b18-00054

Sekiguchi F, 2013, J PHARMACOL SCI, V122, P244, DOI 10.1254/jphs.13R05CP

Sekiguchi F, 2013, BRIT J PHARMACOL, V168, P734, DOI 10.1111/j.1476-5381.2012.02174.x

Shapouri-Moghaddam A, 2018, J CELL PHYSIOL, V233, P6425, DOI 10.1002/jcp.26429

Shen KF, 2013, EXP NEUROL, V247, P466, DOI 10.1016/j.expneurol.2013.01.018

Shouman K, 2021, CLIN AUTON RES, V31, P477, DOI 10.1007/s10286-021-00787-5

Sica A, 2012, J CLIN INVEST, V122, P787, DOI 10.1172/JCI59643

Silva JR, 2017, J NEUROSCI, V37, P6408, DOI 10.1523/JNEUROSCI.2233-16.2017

Simeoli R, 2017, NAT COMMUN, V8, DOI 10.1038/s41467-017-01841-5

Siouti E, 2019, BIOCHEM PHARMACOL, V165, P152, DOI 10.1016/j.bcp.2019.03.029

Staff NP, 2017, ANN NEUROL, V81, P772, DOI 10.1002/ana.24951

Sun J, 2008, AM J PHYSIOL-CELL PH, V294, pC1586, DOI 10.1152/ajpcell.00129.2008

Sun JJ, 2019, J IMMUNOL RES, V2019, DOI 10.1155/2019/7597382

Takahashi T, 2010, PAIN, V150, P183, DOI 10.1016/j.pain.2010.04.022

Talbot S, 2016, ANNU REV IMMUNOL, V34, P421, DOI 10.1146/annurev-immunol-041015-055340

Tanaka J, 2014, NEUROPHARMACOLOGY, V79, P112, DOI 10.1016/j.neuropharm.2013.11.003

Tanaka J, 2013, BRIT J PHARMACOL, V170, P1233, DOI 10.1111/bph.12396

Tao GJ, 2020, CELL DEATH DISCOV, V6, DOI 10.1038/s41420-020-00333-8

Terada Y, 2015, J NEUROSCI RES, V93, P361, DOI 10.1002/jnr.23490

Tian J, 2021, NEUROCHEM RES, V46, P2276, DOI 10.1007/s11064-021-03365-3

Tominaga M, 2001, P NATL ACAD SCI USA, V98, P6951, DOI 10.1073/pnas.111025298

Tomita Shiori, 2020, Eur J Pharmacol, V888, P173587, DOI 10.1016/j.ejphar.2020.173587

Tsubota M, 2019, J NEUROINFLAMM, V16, DOI 10.1186/s12974-019-1581-6

Tsujita R, 2021, BRIT J PHARMACOL, V178, P798, DOI 10.1111/bph.15091

Udalova IA, 2016, NAT REV RHEUMATOL, V12, P472, DOI 10.1038/nrrheum.2016.91

Ulmann L, 2010, EMBO J, V29, P2290, DOI 10.1038/emboj.2010.126

VANFURTH R, 1972, B WORLD HEALTH ORGAN, V46, P845

Veis N, 1996, J INFLAMM, V46, P106

Viola MF, 2020, NEUROGASTROENT MOTIL, V32, DOI 10.1111/nmo.13843

Wang PL, 2020, NAT COMMUN, V11, DOI 10.1038/s41467-020-16355-w

Windgassen S, 2020, CURR BLADDER DYSFUNC, V15, P9, DOI 10.1007/s11884-019-00571-2

Wu JJ, 2017, J NEUROINFLAMM, V14, DOI 10.1186/s12974-017-0828-3

Wu YY, 2021, FRONT CELL DEV BIOL, V8, DOI 10.3389/fcell.2020.617879

Wynn TA, 2016, IMMUNITY, V44, P450, DOI 10.1016/j.immuni.2016.02.015

Wynn TA, 2013, NATURE, V496, P445, DOI 10.1038/nature12034

Yamasoba D, 2016, J PHARMACOL SCI, V130, P139, DOI 10.1016/j.jphs.2016.01.005

Yang DH, 2015, IMMUNITY, V43, P923, DOI 10.1016/j.immuni.2015.10.009

Ydens E, 2020, NAT NEUROSCI, V23, P676, DOI 10.1038/s41593-020-0618-6

Yu XB, 2020, NAT COMMUN, V11, DOI 10.1038/s41467-019-13839-2

Yuan H, 2019, HEADACHE, V59, P20, DOI 10.1111/head.13583

Zhang HM, 2016, J PAIN, V17, P775, DOI 10.1016/j.jpain.2016.02.011

Zhang XM, 2005, EMBO J, V24, P4211, DOI 10.1038/sj.emboj.7600893

Zhang YH, 2015, J BIOL CHEM, V290, P14647, DOI 10.1074/jbc.M115.650218

Zhu XY, 2010, CELL MOL LIFE SCI, V67, P1119, DOI 10.1007/s00018-009-0250-9

Zigrnond RE, 2019, PROG NEUROBIOL, V173, P102, DOI 10.1016/j.pneurobio.2018.12.001

NR 130

TC 69

Z9 75

U1 5

U2 34

PU MDPI

PI BASEL

PA ST ALBAN-ANLAGE 66, CH-4052 BASEL, SWITZERLAND

EI 2073-4409

J9 CELLS-BASEL

JI Cells

PD AUG

PY 2021

VL 10

IS 8

AR 1881

DI 10.3390/cells10081881

PG 18

WC Cell Biology

WE Science Citation Index Expanded (SCI-EXPANDED)

SC Cell Biology

GA UF8LA

UT WOS:000688818800001

PM 34440650

OA gold, Green Published

DA 2025-02-07

ER

PT J

AU Li, J

Xiao, C

Li, CX

He, J

AF Li, Jia

Xiao, Chu

Li, Chunxiang

He, Jie

TI Tissue-resident immune cells: from defining characteristics to roles in

diseases

SO SIGNAL TRANSDUCTION AND TARGETED THERAPY

LA English

DT Review

ID MEMORY T-CELLS; INNATE LYMPHOID-CELLS; MUCOSAL MAST-CELLS; EXPERIMENTAL

AUTOIMMUNE ENCEPHALOMYELITIS; TUMOR-INFILTRATING LYMPHOCYTES;

NATURAL-KILLER-CELLS; ALVEOLAR MACROPHAGES; STREPTOCOCCUS-PNEUMONIAE; NK

CELLS; TRANSCRIPTIONAL REGULATION

AB Tissue-resident immune cells (TRICs) are a highly heterogeneous and plastic subpopulation of immune cells that reside in lymphoid or peripheral tissues without recirculation. These cells are endowed with notably distinct capabilities, setting them apart from their circulating leukocyte counterparts. Many studies demonstrate their complex roles in both health and disease, involving the regulation of homeostasis, protection, and destruction. The advancement of tissue-resolution technologies, such as single-cell sequencing and spatiotemporal omics, provides deeper insights into the cell morphology, characteristic markers, and dynamic transcriptional profiles of TRICs. Currently, the reported TRIC population includes tissue-resident T cells, tissue-resident memory B (BRM) cells, tissue-resident innate lymphocytes, tissue-resident macrophages, tissue-resident neutrophils (TRNs), and tissue-resident mast cells, but unignorably the existence of TRNs is controversial. Previous studies focus on one of them in specific tissues or diseases, however, the origins, developmental trajectories, and intercellular cross-talks of every TRIC type are not fully summarized. In addition, a systemic overview of TRICs in disease progression and the development of parallel therapeutic strategies is lacking. Here, we describe the development and function characteristics of all TRIC types and their major roles in health and diseases. We shed light on how to harness TRICs to offer new therapeutic targets and present burning questions in this field.

C1 [Li, Jia; Xiao, Chu; Li, Chunxiang; He, Jie] Chinese Acad Med Sci & Peking Union Med Coll, Canc Hosp, Natl Canc Ctr, Dept Thorac Surg,Natl Clin Res Ctr Canc, Beijing, Peoples R China.

C3 Chinese Academy of Medical Sciences - Peking Union Medical College;

Cancer Institute & Hospital - CAMS; Peking Union Medical College

RP Li, CX; He, J (corresponding author), Chinese Acad Med Sci & Peking Union Med Coll, Canc Hosp, Natl Canc Ctr, Dept Thorac Surg,Natl Clin Res Ctr Canc, Beijing, Peoples R China.

EM lichunxiang@cicams.ac.cn; prof.jiehe@gmail.com

FU National Natural Science Foundation of China (National Science

Foundation of China) [2021YFF1201303]; National Key R&D Program of China

[2021-1-I2M-012]; CAMS Innovation Fund for Medical Sciences (CIFMS)

[L248050]; Beijing Natural Science Foundation

FX This work was supported by the National Key R&D Program of China

(2021YFF1201303), the CAMS Innovation Fund for Medical Sciences (CIFMS)

(2021-1-I2M-012), and the Beijing Natural Science Foundation (L248050).

CR Abonia JP, 2006, BLOOD, V108, P1588, DOI 10.1182/blood-2005-12-012781

Abrahamsson SV, 2013, BRAIN, V136, P2888, DOI 10.1093/brain/awt182

Adachi T, 2015, NAT MED, V21, P1272, DOI 10.1038/nm.3962

Adams S, 2019, ANN ONCOL, V30, P397, DOI [10.1093/annonc/mdy517, 10.1093/annonc/mdy518]

Aegerter H, 2020, NAT IMMUNOL, V21, P145, DOI 10.1038/s41590-019-0568-x

Albert-Bayo M, 2019, CELLS-BASEL, V8, DOI 10.3390/cells8020135

Alfonso C, 2006, EUR J IMMUNOL, V36, P149, DOI 10.1002/eji.200535127

Allie SR, 2019, NAT IMMUNOL, V20, P97, DOI 10.1038/s41590-018-0260-6

Anadon CM, 2022, CANCER CELL, V40, P545, DOI 10.1016/j.ccell.2022.03.008

Anderson KG, 2014, NAT PROTOC, V9, P209, DOI 10.1038/nprot.2014.005

Nicolas-Avila JA, 2017, IMMUNITY, V46, P15, DOI 10.1016/j.immuni.2016.12.012

Artis D, 2015, NATURE, V517, P293, DOI 10.1038/nature14189

Asada N, 2022, SEMIN IMMUNOPATHOL, V44, P801, DOI 10.1007/s00281-022-00927-7

Bae GH, 2022, BLOOD, V140, P889, DOI 10.1182/blood.2021014283

Bai L, 2021, SCIENCE, V371, P1332, DOI 10.1126/science.aba4177

Bain CC, 2014, NAT IMMUNOL, V15, P929, DOI 10.1038/ni.2967

Bajpai G, 2018, NAT MED, V24, P1234, DOI 10.1038/s41591-018-0059-x

Baker AD, 2010, J LIPID RES, V51, P1325, DOI 10.1194/jlr.M001651

Ballesteros I, 2020, CELL, V183, P1282, DOI 10.1016/j.cell.2020.10.003

Bando JK, 2015, NAT IMMUNOL, V16, P153, DOI 10.1038/ni.3057

Bar-Ephraim YE, 2017, CELL REP, V21, P823, DOI 10.1016/j.celrep.2017.09.070

Baranek T, 2022, TRENDS IMMUNOL, V43, P503, DOI 10.1016/j.it.2022.04.012

Barker KA, 2021, J CLIN INVEST, V131, DOI 10.1172/JCI141810

Behr FM, 2019, FRONT IMMUNOL, V10, DOI 10.3389/fimmu.2019.00400

Beltrán E, 2019, J CLIN INVEST, V129, P4758, DOI 10.1172/JCI128475

Ben-Sasson SZ, 2009, P NATL ACAD SCI USA, V106, P7119, DOI 10.1073/pnas.0902745106

BENDELAC A, 1995, J EXP MED, V182, P2091, DOI 10.1084/jem.182.6.2091

Benichou G, 2017, FRONT IMMUNOL, V8, DOI 10.3389/fimmu.2017.00170

Bernard JK, 2023, SEMIN LIVER DIS, V43, P226, DOI 10.1055/a-2104-9034

Berzins SP, 2006, J IMMUNOL, V176, P4059, DOI 10.4049/jimmunol.176.7.4059

Bharat A, 2016, AM J RESP CELL MOL, V54, P147, DOI 10.1165/rcmb.2015-0147LE

Bhasin M, 2007, BMC IMMUNOL, V8, DOI 10.1186/1471-2172-8-10

Bian ZL, 2020, NATURE, V582, P571, DOI 10.1038/s41586-020-2316-7

Bigley V, 2011, J EXP MED, V208, P227, DOI 10.1084/jem.20101459

Bischoff SC, 2007, NAT REV IMMUNOL, V7, P93, DOI 10.1038/nri2018

Boniface K, 2018, J INVEST DERMATOL, V138, P355, DOI 10.1016/j.jid.2017.08.038

Böttcher K, 2018, HEPATOLOGY, V68, P172, DOI 10.1002/hep.29782

Boyman O, 2004, J EXP MED, V199, P731, DOI 10.1084/jem.20031482

Bromley SK, 2013, J IMMUNOL, V190, P970, DOI 10.4049/jimmunol.1202805

Brownlie D, 2021, P NATL ACAD SCI USA, V118, DOI 10.1073/pnas.2016580118

Buckley CD, 2019, NATURE, V572, P590, DOI 10.1038/d41586-019-02340-x

Buggert M, 2023, NAT IMMUNOL, V24, P1076, DOI 10.1038/s41590-023-01538-6

Bujko A, 2018, J EXP MED, V215, P441, DOI 10.1084/jem.20170057

Buonocore S, 2010, NATURE, V464, P1371, DOI 10.1038/nature08949

Butovsky O, 2014, NAT NEUROSCI, V17, P131, DOI 10.1038/nn.3599

Buttgereit A, 2016, NAT IMMUNOL, V17, P1397, DOI 10.1038/ni.3585

Buus TB, 2017, NAT COMMUN, V8, DOI 10.1038/s41467-017-01963-w

Califano D, 2018, MUCOSAL IMMUNOL, V11, P209, DOI 10.1038/mi.2017.41

Carbone FR, 2019, SCI IMMUNOL, V4, DOI 10.1126/sciimmunol.aax5595

Carlson CM, 2006, NATURE, V442, P299, DOI 10.1038/nature04882

Casanova-Acebes M, 2018, J EXP MED, V215, P2778, DOI 10.1084/jem.20181468

Casey KA, 2012, J IMMUNOL, V188, P4866, DOI 10.4049/jimmunol.1200402

CEPEK KL, 1994, NATURE, V372, P190, DOI 10.1038/372190a0

Chabot S, 1997, J CLIN INVEST, V100, P604, DOI 10.1172/JCI119571

Chakarov S, 2019, SCIENCE, V363, P1190, DOI 10.1126/science.aau0964

Chang JT, 2014, NAT IMMUNOL, V15, P1104, DOI 10.1038/ni.3031

Chang MH, 2021, CELL REP, V37, DOI 10.1016/j.celrep.2021.109902

Chen Changfeng, 2022, Adv Immunol, V155, P1, DOI 10.1016/bs.ai.2022.08.001

Chen L, 2020, CELL MOL IMMUNOL, V17, P64, DOI 10.1038/s41423-019-0291-4

Chen Y, 2021, CELLS-BASEL, V10, DOI 10.3390/cells10082143

Cheng SJ, 2021, CELL, V184, P792, DOI 10.1016/j.cell.2021.01.010

Cheon IS, 2023, IMMUNOL REV, V316, P63, DOI 10.1111/imr.13201

Cheuk S, 2017, IMMUNITY, V46, P287, DOI 10.1016/j.immuni.2017.01.009

Chorro L, 2009, J EXP MED, V206, P3089, DOI 10.1084/jem.20091586

Cildir G, 2021, TRENDS IMMUNOL, V42, P523, DOI 10.1016/j.it.2021.04.004

Cindik E D, 2000, Technol Health Care, V8, P267

Coles MC, 2000, J IMMUNOL, V164, P2412, DOI 10.4049/jimmunol.164.5.2412

Collins N, 2016, NAT COMMUN, V7, DOI 10.1038/ncomms11514

Collison J, 2019, NAT REV RHEUMATOL, V15, P573, DOI 10.1038/s41584-019-0295-6

Constantinides MG, 2019, SCIENCE, V366, P445, DOI 10.1126/science.aax6624

Constantinides MG, 2014, NATURE, V508, P397, DOI 10.1038/nature13047

Constantinides MG, 2013, CURR OPIN IMMUNOL, V25, P161, DOI 10.1016/j.coi.2013.01.003

Cooper GE, 2018, FRONT IMMUNOL, V9, DOI 10.3389/fimmu.2018.01671

Corsiero E, 2016, FRONT IMMUNOL, V7, DOI 10.3389/fimmu.2016.00485

Cortez VS, 2016, IMMUNITY, V44, P1127, DOI 10.1016/j.immuni.2016.03.007

Cortez VS, 2014, J IMMUNOL, V192, P4487, DOI 10.4049/jimmunol.1303469

Crosby CM, 2018, NAT REV IMMUNOL, V18, P559, DOI 10.1038/s41577-018-0034-2

Crowl JT, 2022, NAT IMMUNOL, V23, P1121, DOI 10.1038/s41590-022-01229-8

Çuburu N, 2018, INT J CANCER, V142, P1467, DOI 10.1002/ijc.31166

Culemann S, 2019, NATURE, V572, P670, DOI 10.1038/s41586-019-1471-1

Cuthbert RJ, 2019, ANN RHEUM DIS, V78, P1559, DOI 10.1136/annrheumdis-2019-215210

Daussy C, 2014, J EXP MED, V211, P563, DOI 10.1084/jem.20131560

Davies LC, 2013, NAT IMMUNOL, V14, P986, DOI 10.1038/ni.2705

de Bree GJ, 2005, J EXP MED, V202, P1433, DOI 10.1084/jem.20051365

de Bree GJ, 2007, J INFECT DIS, V195, P1718, DOI 10.1086/517612

De Schepper S, 2018, CELL, V175, P400, DOI 10.1016/j.cell.2018.07.048

Deng ZH, 2024, NATURE, V626, DOI 10.1038/s41586-023-06950-4

Devi S, 2013, J EXP MED, V210, P2321, DOI 10.1084/jem.20130056

Dick SA, 2022, SCI IMMUNOL, V7, DOI 10.1126/sciimmunol.abf7777

Didierlaurent A, 2008, J EXP MED, V205, P323, DOI 10.1084/jem.20070891

Djenidi F, 2015, J IMMUNOL, V194, P3475, DOI 10.4049/jimmunol.1402711

Dogra P, 2020, CELL, V180, P749, DOI 10.1016/j.cell.2020.01.022

Dong YF, 2019, NAT REV NEUROL, V15, P704, DOI 10.1038/s41582-019-0253-6

Dudeck J, 2021, IMMUNITY, V54, P468, DOI 10.1016/j.immuni.2020.12.017

Dusseaux M, 2011, BLOOD, V117, P1250, DOI 10.1182/blood-2010-08-303339

Dwyer DF, 2016, NAT IMMUNOL, V17, P878, DOI 10.1038/ni.3445

El-Asady R, 2005, J EXP MED, V201, P1647, DOI 10.1084/jem.20041044

Ely KH, 2006, J IMMUNOL, V176, P537, DOI 10.4049/jimmunol.176.1.537

Enamorado M, 2017, NAT COMMUN, V8, DOI 10.1038/ncomms16073

ENERBACK L, 1987, INT ARCH ALLER A IMM, V82, P249, DOI 10.1159/000234199

Epelman S, 2014, IMMUNITY, V41, P21, DOI 10.1016/j.immuni.2014.06.013

Ericsson A, 2004, EUR J IMMUNOL, V34, P2720, DOI 10.1002/eji.200425125

Evrard M, 2023, IMMUNITY, V56, P1664, DOI 10.1016/j.immuni.2023.06.005

Fan X, 2016, CELL, V164, P1198, DOI 10.1016/j.cell.2016.02.048

Fang JL, 2024, J TRANSL MED, V22, DOI 10.1186/s12967-024-04978-2

Fasbender F, 2016, FRONT IMMUNOL, V7, DOI 10.3389/fimmu.2016.00019

Fatahzadeh M, 2007, J AM ACAD DERMATOL, V57, P737, DOI 10.1016/j.jaad.2007.06.027

Fekete R, 2018, ACTA NEUROPATHOL, V136, P461, DOI 10.1007/s00401-018-1885-0

Fert-Bober J, 2020, IMMUNOL REV, V294, P133, DOI 10.1111/imr.12834

Filgueira L, 2021, CELLS-BASEL, V10, DOI 10.3390/cells10071836

FitzPatrick MEB, 2021, CELL REP, V34, DOI 10.1016/j.celrep.2020.108661

Forsythe P, 2019, TRENDS NEUROSCI, V42, P43, DOI 10.1016/j.tins.2018.09.006

Franciszkiewicz K, 2016, IMMUNOL REV, V272, P120, DOI 10.1111/imr.12423

Fransen NL, 2020, BRAIN, V143, P1714, DOI 10.1093/brain/awaa117

Friend DS, 1996, J CELL BIOL, V135, P279, DOI 10.1083/jcb.135.1.279

Frieser D, 2022, SCI TRANSL MED, V14, DOI 10.1126/scitranslmed.abl6157

Frisoli ML, 2020, ANNU REV IMMUNOL, V38, P621, DOI 10.1146/annurev-immunol-100919-023531

Furness JB, 2012, NAT REV GASTRO HEPAT, V9, P286, DOI 10.1038/nrgastro.2012.32

GALLI SJ, 1993, J CLIN INVEST, V91, P148, DOI 10.1172/JCI116164

GALLI SJ, 1990, LAB INVEST, V62, P5

Galli SJ, 2012, NAT MED, V18, P693, DOI 10.1038/nm.2755

Ganesan AP, 2017, NAT IMMUNOL, V18, P940, DOI 10.1038/ni.3775

Gao AQ, 2022, J AUTOIMMUN, V133, DOI 10.1016/j.jaut.2022.102950

Gao CY, 2019, ARTHRITIS RHEUMATOL, V71, P121, DOI 10.1002/art.40676

Gao Z, 2011, NEUROL RES INT, V2011, DOI 10.1155/2011/383087

Gasteiger G, 2015, SCIENCE, V350, P981, DOI 10.1126/science.aac9593

Gattinoni L, 2011, NAT MED, V17, P1290, DOI 10.1038/nm.2446

Geissmann F, 2005, PLOS BIOL, V3, P650, DOI 10.1371/journal.pbio.0030113

Gentek R, 2018, IMMUNITY, V48, P1160, DOI 10.1016/j.immuni.2018.04.025

Gerlach C, 2016, IMMUNITY, V45, P1270, DOI 10.1016/j.immuni.2016.10.018

Ginhoux F, 2016, IMMUNITY, V44, P439, DOI 10.1016/j.immuni.2016.02.024

Ginhoux F, 2010, SCIENCE, V330, P841, DOI 10.1126/science.1194637

Ginsberg P, 2023, FRONT IMMUNOL, V14, DOI 10.3389/fimmu.2023.1111521

Godfrey DI, 2015, NAT IMMUNOL, V16, P1114, DOI 10.1038/ni.3298

González H, 2014, J NEUROIMMUNOL, V274, P1, DOI 10.1016/j.jneuroim.2014.07.012

GOODMAN T, 1989, J EXP MED, V170, P1569, DOI 10.1084/jem.170.5.1569

Goplen NP, 2020, SCI IMMUNOL, V5, DOI 10.1126/sciimmunol.abc4557

Gorski SA, 2013, PLOS PATHOG, V9, DOI 10.1371/journal.ppat.1003615

Graham JB, 2014, J IMMUNOL, V192, P683, DOI 10.4049/jimmunol.1202153

Gray JI, 2022, ANNU REV IMMUNOL, V40, P195, DOI 10.1146/annurev-immunol-093019-112809

Gregoire C, 2022, IMMUNITY, V55, P1216, DOI 10.1016/j.immuni.2022.06.002

Grigg JB, 2021, NATURE, V600, P707, DOI 10.1038/s41586-021-04136-4

Groschwitz KR, 2009, P NATL ACAD SCI USA, V106, P22381, DOI 10.1073/pnas.0906372106

Guia S, 2020, TRENDS IMMUNOL, V41, P436, DOI 10.1016/j.it.2020.03.002

Guilliams M, 2022, CELL, V185, P379, DOI 10.1016/j.cell.2021.12.018

Guilliams M, 2021, NAT IMMUNOL, V22, P118, DOI 10.1038/s41590-020-00849-2

Gurish MF, 2001, J EXP MED, V194, P1243, DOI 10.1084/jem.194.9.1243

Gurish MF, 2012, IMMUNITY, V37, P25, DOI 10.1016/j.immuni.2012.07.003

Haringman JJ, 2005, ANN RHEUM DIS, V64, P834, DOI 10.1136/ard.2004.029751

Harris JE, 2016, J AM ACAD DERMATOL, V74, P370, DOI 10.1016/j.jaad.2015.09.073

Haruwaka K, 2019, NAT COMMUN, V10, DOI 10.1038/s41467-019-13812-z

Hasan MH, 2022, CURR OPIN IMMUNOL, V74, DOI 10.1016/j.coi.2021.10.005

Hashimoto D, 2013, IMMUNITY, V38, P792, DOI 10.1016/j.immuni.2013.04.004

HAYASHI C, 1985, DEV BIOL, V109, P234, DOI 10.1016/0012-1606(85)90363-X

Hedrick CC, 2022, NAT REV IMMUNOL, V22, P173, DOI 10.1038/s41577-021-00571-6

Henderson WR, 1998, J INFECT DIS, V177, P1437, DOI 10.1086/517833

Henz BM, 2001, EXP DERMATOL, V10, P1, DOI 10.1034/j.1600-0625.2001.100101.x

Heppner FL, 2005, NAT MED, V11, P146, DOI 10.1038/nm1177

Heymann F, 2015, HEPATOLOGY, V62, P279, DOI 10.1002/hep.27793

Hidalgo A, 2019, TRENDS IMMUNOL, V40, P584, DOI 10.1016/j.it.2019.04.013

Hildreth AD, 2019, VIRUSES-BASEL, V11, DOI 10.3390/v11030272

Hoeffel G, 2015, FRONT IMMUNOL, V6, DOI 10.3389/fimmu.2015.00486

Hoeffel G, 2015, IMMUNITY, V42, P665, DOI 10.1016/j.immuni.2015.03.011

Hofmann M, 2011, P NATL ACAD SCI USA, V108, P16741, DOI 10.1073/pnas.1107200108

Holtmeier V, 2001, J INVEST DERMATOL, V116, P275, DOI 10.1046/j.1523-1747.2001.01250.x

Hombrink P, 2016, NAT IMMUNOL, V17, P1467, DOI 10.1038/ni.3589

Hondowicz BD, 2016, IMMUNITY, V44, P155, DOI 10.1016/j.immuni.2015.11.004

Hoorweg K, 2012, FRONT IMMUNOL, V3, DOI 10.3389/fimmu.2012.00072

Hu LN, 2023, ISCIENCE, V26, DOI 10.1016/j.isci.2023.107187

Huang H, 2016, SEMIN IMMUNOPATHOL, V38, P539, DOI 10.1007/s00281-016-0562-4

Huang J, 2021, FRONT IMMUNOL, V12, DOI 10.3389/fimmu.2021.686155

Huang QQ, 2021, SCI ADV, V7, DOI 10.1126/sciadv.abd0515

Huang YL, 2011, J NEUROSCI, V31, P15195, DOI 10.1523/JNEUROSCI.2051-11.2011

Hudspeth K, 2016, J AUTOIMMUN, V66, P40, DOI 10.1016/j.jaut.2015.08.011

Hunter S, 2018, J HEPATOL, V69, P654, DOI 10.1016/j.jhep.2018.05.007

Iijima N, 2014, SCIENCE, V346, P93, DOI 10.1126/science.1257530

Inoue Y, 1996, AM J PATHOL, V149, P2037

Ishizuka IE, 2016, ANNU REV IMMUNOL, V34, P299, DOI 10.1146/annurev-immunol-041015-055549

Jacome-Galarza CE, 2019, NATURE, V568, P541, DOI 10.1038/s41586-019-1105-7

Jiang XD, 2012, NATURE, V483, P227, DOI 10.1038/nature10851

Joo HM, 2008, P NATL ACAD SCI USA, V105, P3485, DOI 10.1073/pnas.0800003105

Joshi NS, 2007, IMMUNITY, V27, P281, DOI 10.1016/j.immuni.2007.07.010

Jozwik A, 2015, NAT COMMUN, V6, DOI 10.1038/ncomms10224

Jung JH, 2020, CLIN TRANSL IMMUNOL, V9, DOI 10.1002/cti2.1140

Kalkunte S, 2008, AM J REPROD IMMUNOL, V59, P425, DOI 10.1111/j.1600-0897.2008.00595.x

Kallies A, 2011, BLOOD, V117, P1869, DOI 10.1182/blood-2010-08-303123

Kallies A, 2009, IMMUNITY, V31, P283, DOI 10.1016/j.immuni.2009.06.021

Kang SP, 2022, FASEB J, V36, DOI 10.1096/fj.202101443RR

Kang SP, 2021, FRONT IMMUNOL, V11, DOI 10.3389/fimmu.2020.588227

Karaki S, 2021, J IMMUNOTHER CANCER, V9, DOI 10.1136/jitc-2020-001948

Katzilieris-Petras G, 2022, J VIROL, V96, DOI [10.1128/jvi.01311-21, 10.1128/JVI.01311-21]

Kawasaki T, 2022, CELL REP, V41, DOI 10.1016/j.celrep.2022.111828

Keith YH, 2023, J ALLERGY CLIN IMMUN, V151, P159, DOI 10.1016/j.jaci.2022.09.011

Kemble S, 2021, FRONT IMMUNOL, V12, DOI 10.3389/fimmu.2021.715894

Keskin DB, 2007, P NATL ACAD SCI USA, V104, P3378, DOI 10.1073/pnas.0611098104

KHAN AI, 1993, INT J PARASITOL, V23, P551, DOI 10.1016/0020-7519(93)90159-V

Khan TN, 2016, J EXP MED, V213, P951, DOI 10.1084/jem.20151855

Kirchhammer N, 2022, SCI TRANSL MED, V14, DOI 10.1126/scitranslmed.abm9043

KITAMURA Y, 1978, BLOOD, V52, P447

KITAMURA Y, 1979, BLOOD, V53, P1085

KITAMURA Y, 1977, NATURE, V268, P442, DOI 10.1038/268442a0

KITAMURA Y, 1979, DEV BIOL, V70, P510, DOI 10.1016/0012-1606(79)90042-3

Klonowski KD, 2004, IMMUNITY, V20, P551, DOI 10.1016/S1074-7613(04)00103-7

Klose CSN, 2020, CELL RES, V30, P475, DOI 10.1038/s41422-020-0323-8

Klose CSN, 2014, CELL, V157, P340, DOI 10.1016/j.cell.2014.03.030

Koethe S, 2011, J LEUKOCYTE BIOL, V90, P5, DOI 10.1189/jlb.0710404

Kok L, 2022, NAT REV IMMUNOL, V22, P283, DOI 10.1038/s41577-021-00590-3

Komdeur FL, 2017, ONCOIMMUNOLOGY, V6, DOI 10.1080/2162402X.2017.1338230

Kondo T, 2002, NEUROLOGY, V59, P1105, DOI 10.1212/WNL.59.7.1105

Koutsakos M, 2019, NAT IMMUNOL, V20, P613, DOI 10.1038/s41590-019-0320-6

Kovalovsky D, 2008, NAT IMMUNOL, V9, P1055, DOI 10.1038/ni.1641

Kragten NAM, 2021, EUR J IMMUNOL, V51, P136, DOI 10.1002/eji.202149360

Krebs CF, 2020, SCI IMMUNOL, V5, DOI 10.1126/sciimmunol.aba4163

Krishnan S, 2018, P NATL ACAD SCI USA, V115, P10738, DOI 10.1073/pnas.1802320115

Krovi SH, 2018, FRONT IMMUNOL, V9, DOI 10.3389/fimmu.2018.01393

Kumagai Y, 2007, IMMUNITY, V27, P240, DOI 10.1016/j.immuni.2007.07.013

Kumar BV, 2017, CELL REP, V20, P2921, DOI 10.1016/j.celrep.2017.08.078

Kuric E, 2017, AM J PATHOL, V187, P581, DOI 10.1016/j.ajpath.2016.11.002

Kurihara K, 2019, J DERMATOL SCI, V95, P21, DOI 10.1016/j.jdermsci.2019.06.002

Kurosaki T, 2015, NAT REV IMMUNOL, V15, P149, DOI 10.1038/nri3802

Kuziel WA, 1997, P NATL ACAD SCI USA, V94, P12053, DOI 10.1073/pnas.94.22.12053

Kvestak D, 2024, FRONT CELL NEUROSCI, V18, DOI 10.3389/fncel.2024.1364485

Kwong B, 2017, NAT IMMUNOL, V18, P1117, DOI 10.1038/ni.3816

Lai C, 2021, J IMMUNOTHER CANCER, V9, DOI 10.1136/jitc-2020-001807

Laidlaw BJ, 2014, IMMUNITY, V41, P633, DOI 10.1016/j.immuni.2014.09.007

Lam AJ, 2019, J IMMUNOL, V202, P2195, DOI 10.4049/jimmunol.1801330

Lavin Y, 2015, NAT REV IMMUNOL, V15, P731, DOI 10.1038/nri3920

Lazarov T, 2023, NATURE, V618, P698, DOI 10.1038/s41586-023-06002-x

Lee S, 2024, CURR OPIN VIROL, V65, DOI 10.1016/j.coviro.2024.101397

Lee YJ, 2015, IMMUNITY, V43, P566, DOI 10.1016/j.immuni.2015.06.025

Lehmann GL, 2020, J EXP MED, V217, DOI 10.1084/jem.20190730

Leijten EF, 2021, ARTHRITIS RHEUMATOL, V73, P1220, DOI 10.1002/art.41652

LEVISCHAFFER F, 1990, EXP CELL RES, V188, P42, DOI 10.1016/0014-4827(90)90275-F

Li BWS, 2019, EUR J IMMUNOL, V49, P144, DOI 10.1002/eji.201747421

Li CF, 2019, IMMUNITY, V51, P491, DOI 10.1016/j.immuni.2019.08.013

Li C, 2023, NATURE, V621, P373, DOI 10.1038/s41586-023-06473-y

Li GQ, 2023, FRONT IMMUNOL, V14, DOI 10.3389/fimmu.2023.1206299

Li YK, 2022, FRONT IMMUNOL, V13, DOI 10.3389/fimmu.2022.967055

Li ZQ, 2018, IMMUNITY, V49, P640, DOI 10.1016/j.immuni.2018.09.023

Liechti T, 2019, CYTOM PART A, V95, P946, DOI 10.1002/cyto.a.23850

Liew PX, 2019, PHYSIOL REV, V99, P1223, DOI 10.1152/physrev.00012.2018

Lin YH, 2023, IMMUNITY, V56, P207, DOI 10.1016/j.immuni.2022.12.007

Liu C, 2021, CELL RES, V31, P1106, DOI 10.1038/s41422-021-00529-2

Liu K, 2022, J CELL BIOL, V221, DOI 10.1083/jcb.202108093

Liu LZ, 2010, NAT MED, V16, P224, DOI 10.1038/nm.2078

Lombes A, 2015, J IMMUNOL, V195, P1449, DOI 10.4049/jimmunol.1500375

Lunemann S, 2017, SCI REP-UK, V7, DOI 10.1038/s41598-017-06011-7

Lynch L, 2015, NAT IMMUNOL, V16, P85, DOI 10.1038/ni.3047

Machado-Santos J, 2018, BRAIN, V141, P2066, DOI 10.1093/brain/awy151

Mackay LK, 2017, TRENDS IMMUNOL, V38, P94, DOI 10.1016/j.it.2016.11.004

Mackay LK, 2016, SCIENCE, V352, P459, DOI 10.1126/science.aad2035

Mackay LK, 2015, IMMUNITY, V43, P1101, DOI 10.1016/j.immuni.2015.11.008

Mackay LK, 2013, NAT IMMUNOL, V14, P1294, DOI 10.1038/ni.2744

Mackay LK, 2012, P NATL ACAD SCI USA, V109, P7037, DOI 10.1073/pnas.1202288109

MacLean AJ, 2022, IMMUNITY, V55, P718, DOI 10.1016/j.immuni.2022.03.003

MacParland SA, 2018, NAT COMMUN, V9, DOI 10.1038/s41467-018-06318-7

Mahlakoiv T, 2019, SCI IMMUNOL, V4, DOI 10.1126/sciimmunol.aax0416

Mak RKH, 2009, ACTAS DERMO-SIFILOGR, V100, P2, DOI 10.1016/S0001-7310(09)73372-1

Malaviya R, 1996, NATURE, V381, P77, DOI 10.1038/381077a0

Malaviya R, 1996, J IMMUNOL, V156, P1490

Mami-Chouaib F, 2018, J IMMUNOTHER CANCER, V6, DOI 10.1186/s40425-018-0399-6

Marcocci ME, 2020, TRENDS MICROBIOL, V28, P808, DOI 10.1016/j.tim.2020.03.003

Marquardt N, 2019, NAT COMMUN, V10, DOI 10.1038/s41467-019-11632-9

Marquardt N, 2017, J ALLERGY CLIN IMMUN, V139, P1321, DOI 10.1016/j.jaci.2016.07.043

Martini E, 2019, CIRCULATION, V140, P2089, DOI 10.1161/CIRCULATIONAHA.119.041694

Masopust D, 2006, J IMMUNOL, V176, P2079, DOI 10.4049/jimmunol.176.4.2079

Masopust D, 2019, ANNU REV IMMUNOL, V37, P521, DOI 10.1146/annurev-immunol-042617-053214

Masopust D, 2010, J EXP MED, V207, P553, DOI 10.1084/jem.20090858

Mass E, 2023, NAT REV IMMUNOL, DOI 10.1038/s41577-023-00848-y

Mass E, 2016, SCIENCE, V353, DOI 10.1126/science.aaf4238

Masuda T, 2022, NATURE, V604, P740, DOI 10.1038/s41586-022-04596-2

Mathew NR, 2021, CELL REP, V35, DOI 10.1016/j.celrep.2021.109286

MATSUDA H, 1981, J CELL PHYSIOL, V108, P409, DOI 10.1002/jcp.1041080315

MAURER D, 1995, J INVEST DERMATOL, V104, P707, DOI 10.1111/1523-1747.ep12606958

Maurer M, 1997, LAB INVEST, V77, P319

Maurer M, 2003, EXP DERMATOL, V12, P886, DOI 10.1111/j.0906-6705.2003.0109a.x

Mauro D, 2024, ANN RHEUM DIS, V83, P1345, DOI 10.1136/ard-2023-225069

McCully ML, 2018, J IMMUNOL, V200, P1639, DOI 10.4049/jimmunol.1701377

McGovern N, 2014, IMMUNITY, V41, P465, DOI 10.1016/j.immuni.2014.08.006

McGrath KE, 2015, CELL REP, V11, P1892, DOI 10.1016/j.celrep.2015.05.036

McMillan RE, 2023, EXP NEUROL, V363, DOI 10.1016/j.expneurol.2023.114375

Medvinsky A, 1996, CELL, V86, P897, DOI 10.1016/S0092-8674(00)80165-8

Menon B, 2014, ARTHRITIS RHEUMATOL, V66, P1272, DOI 10.1002/art.38376

Merad M, 2002, NAT IMMUNOL, V3, P1135, DOI 10.1038/ni852

Metcalfe DD, 1997, PHYSIOL REV, V77, P1033, DOI 10.1152/physrev.1997.77.4.1033

Micera A, 2001, P NATL ACAD SCI USA, V98, P6162, DOI 10.1073/pnas.101130898

Milner JJ, 2017, NATURE, V552, P253, DOI 10.1038/nature24993

Miron M, 2021, GENOME MED, V13, DOI 10.1186/s13073-021-00918-7

Miron VE, 2014, J NEUROCHEM, V130, P165, DOI 10.1111/jnc.12705

Mirza R, 2009, AM J PATHOL, V175, P2454, DOI 10.2353/ajpath.2009.090248

Miyabe C, 2019, J CLIN INVEST, V129, P3610, DOI 10.1172/JCI123778

Mohammed J, 2016, NAT IMMUNOL, V17, P414, DOI 10.1038/ni.3396

Molodtsov AK, 2021, IMMUNITY, V54, P2117, DOI 10.1016/j.immuni.2021.08.019

Montaldo E, 2016, IMMUNOL LETT, V179, P2, DOI 10.1016/j.imlet.2016.01.007

Monticelli LA, 2011, NAT IMMUNOL, V12, P1045, DOI [10.1038/ni.2131, 10.1031/ni.2131]

Motohashi S, 2006, CLIN CANCER RES, V12, P6079, DOI 10.1158/1078-0432.CCR-06-0114

Mueller SN, 2013, ANNU REV IMMUNOL, V31, P137, DOI 10.1146/annurev-immunol-032712-095954

Mukai K, 2018, IMMUNOL REV, V282, P121, DOI 10.1111/imr.12634

Mulherin D, 1996, ARTHRITIS RHEUM, V39, P115, DOI 10.1002/art.1780390116

Nadler MJS, 2001, ADV IMMUNOL, V76, P325, DOI 10.1016/S0065-2776(01)76022-1

NAWA Y, 1985, PARASITE IMMUNOL, V7, P429, DOI 10.1111/j.1365-3024.1985.tb00088.x

Neupane AS, 2020, CELL, V183, P110, DOI 10.1016/j.cell.2020.08.020

Nikic I, 2011, NAT MED, V17, P495, DOI 10.1038/nm.2324

Noe A, 2022, FRONT IMMUNOL, V13, DOI 10.3389/fimmu.2022.795463

Norrby K, 2002, APMIS, V110, P355, DOI 10.1034/j.1600-0463.2002.100501.x

Oh JE, 2021, SCI IMMUNOL, V6, DOI 10.1126/sciimmunol.abj5129

Oja AE, 2018, MUCOSAL IMMUNOL, V11, P654, DOI 10.1038/mi.2017.94

Okabe Y, 2014, CELL, V157, P832, DOI 10.1016/j.cell.2014.04.016

Olson JA, 2013, IMMUNITY, V38, P1250, DOI 10.1016/j.immuni.2013.05.009

Onodera T, 2012, P NATL ACAD SCI USA, V109, P2485, DOI 10.1073/pnas.1115369109

Onuora S, 2022, NAT REV RHEUMATOL, V18, P5, DOI 10.1038/s41584-021-00730-y

Osborn JF, 2019, PLOS PATHOG, V15, DOI 10.1371/journal.ppat.1007633

Ostkamp P, 2022, SCI TRANSL MED, V14, DOI 10.1126/scitranslmed.adc9778

Owczarczyk-Saczonek A, 2020, INT J MOL SCI, V21, DOI 10.3390/ijms21020625

Paik DH, 2021, J EXP MED, V218, DOI 10.1084/jem.20200218

Pallett LJ, 2017, J EXP MED, V214, P1567, DOI 10.1084/jem.20162115

Pan YD, 2017, NATURE, V543, P252, DOI 10.1038/nature21379

Panda SK, 2019, FRONT IMMUNOL, V10, DOI 10.3389/fimmu.2019.00861

Parikh SV, 2020, AM J KIDNEY DIS, V76, P265, DOI 10.1053/j.ajkd.2019.10.017

Park DS, 2023, NATURE, V623, DOI 10.1038/s41586-023-06713-1

Park SL, 2018, NAT IMMUNOL, V19, P183, DOI 10.1038/s41590-017-0027-5

Pellicci DG, 2020, NAT REV IMMUNOL, V20, P756, DOI 10.1038/s41577-020-0345-y

Peng H, 2017, SEMIN IMMUNOL, V31, P3, DOI 10.1016/j.smim.2017.07.006

Peng H, 2016, SCI CHINA LIFE SCI, V59, P1218, DOI 10.1007/s11427-016-0334-2

Peng H, 2013, J CLIN INVEST, V123, P1444, DOI 10.1172/JCI66381

Perdiguero EG, 2015, NATURE, V518, P547, DOI 10.1038/nature13989

Pervizaj-Oruqaj L, 2024, NAT COMMUN, V15, DOI 10.1038/s41467-023-44421-6

Petegrosso R, 2020, BRIEF BIOINFORM, V21, P1209, DOI 10.1093/bib/bbz063

Piet B, 2011, J CLIN INVEST, V121, P2254, DOI 10.1172/JCI44675

Pizzolla A, 2018, J CLIN INVEST, V128, P721, DOI 10.1172/JCI96957

Pollard JW, 2009, NAT REV IMMUNOL, V9, P259, DOI 10.1038/nri2528

Ponomarev ED, 2007, J NEUROSCI, V27, P10714, DOI 10.1523/JNEUROSCI.1922-07.2007

Poon MML, 2023, NAT IMMUNOL, V24, P309, DOI 10.1038/s41590-022-01395-9

Povoleri GAM, 2023, CELL REP, V42, DOI 10.1016/j.celrep.2023.112514

Prasad S, 2018, IMMUN INFLAMM DIS, V6, P332, DOI 10.1002/iid3.221

Prince LR, 2017, BLOOD, V130, P1014, DOI 10.1182/blood-2017-03-770164

Prinz I, 2013, EUR J IMMUNOL, V43, P1988, DOI 10.1002/eji.201343759

Purnama C, 2014, EUR J IMMUNOL, V44, P2003, DOI 10.1002/eji.201344359

Putro E, 2024, INT J MOL SCI, V25, DOI 10.3390/ijms25115594

Qin G, 2011, J VIROL, V85, P10109, DOI 10.1128/JVI.05341-11

Qin Y, 2019, EXP MOL MED, V51, DOI 10.1038/s12276-019-0329-9

Qiu ZJ, 2023, J EXP MED, V220, DOI 10.1084/jem.20210923

Quinn KM, 2018, CELL REP, V23, P3512, DOI 10.1016/j.celrep.2018.05.057

Quinn KM, 2016, P NATL ACAD SCI USA, V113, P1333, DOI 10.1073/pnas.1525167113

Ramos RN, 2022, CELL, V185, P1189, DOI 10.1016/j.cell.2022.02.021

Rana BMJ, 2019, J EXP MED, V216, P1999, DOI 10.1084/jem.20190689

Ray SJ, 2004, IMMUNITY, V20, P167, DOI 10.1016/S1074-7613(04)00021-4

Redhead ML, 2016, BIOL REPROD, V94, DOI 10.1095/biolreprod.116.138495

Reusch L, 2023, EUR J IMMUNOL, V53, DOI 10.1002/eji.202250085

Richmond JM, 2019, J INVEST DERMATOL, V139, P769, DOI 10.1016/j.jid.2018.10.032

Richmond JM, 2018, SCI TRANSL MED, V10, DOI 10.1126/scitranslmed.aam7710

Richoz N, 2022, JCI INSIGHT, V7, DOI 10.1172/jci.insight.159751

Romagnoli PA, 2017, MUCOSAL IMMUNOL, V10, P520, DOI 10.1038/mi.2016.66

Roychoudhury P, 2020, J CLIN INVEST, V130, P2903, DOI 10.1172/JCI132583

Ruf B, 2023, CELL, V186, DOI 10.1016/j.cell.2023.07.026

Ryan GE, 2021, FRONT IMMUNOL, V12, DOI 10.3389/fimmu.2021.652191

Salcher S, 2022, CANCER CELL, V40, P1503, DOI 10.1016/j.ccell.2022.10.008

Sallusto F, 1999, NATURE, V401, P708, DOI 10.1038/44385

Salou M, 2019, J EXP MED, V216, P133, DOI 10.1084/jem.20181483

Sasson SC, 2020, CELL MOL IMMUNOL, V17, P113, DOI 10.1038/s41423-019-0359-1

Sasson SC, 2021, GASTROENTEROLOGY, V161, P1229, DOI 10.1053/j.gastro.2021.06.025

Sato M, 2022, FRONT MED-LAUSANNE, V9, DOI 10.3389/fmed.2022.1036787

Savelyeva N, 2020, BLOOD, V136, P2722, DOI 10.1182/blood.2020007890

SAWYER RT, 1982, LAB INVEST, V46, P165

Scanlon ST, 2011, J EXP MED, V208, P2113, DOI 10.1084/jem.20110522

Schafer ST, 2023, CELL, V186, P2111, DOI 10.1016/j.cell.2023.04.022

Scharenberg M, 2019, FRONT IMMUNOL, V10, DOI 10.3389/fimmu.2019.01116

Schenkel JM, 2014, SCIENCE, V346, P98, DOI 10.1126/science.1254536

Schenkel JM, 2013, NAT IMMUNOL, V14, P509, DOI 10.1038/ni.2568

Schenkel JM, 2023, NAT REV IMMUNOL, V23, P807, DOI 10.1038/s41577-023-00884-8

Scheper W, 2013, ONCOIMMUNOLOGY, V2, DOI 10.4161/onci.23974

Scherer HU, 2020, J AUTOIMMUN, V110, DOI 10.1016/j.jaut.2019.102400

Schulz C, 2012, SCIENCE, V336, P86, DOI 10.1126/science.1219179

Schulz-Kuhnt A, 2020, FRONT IMMUNOL, V11, DOI 10.3389/fimmu.2020.01062

Scott CL, 2016, NAT COMMUN, V7, DOI 10.1038/ncomms10321

Seach N, 2013, J IMMUNOL, V191, P6002, DOI 10.4049/jimmunol.1301212

Seillet C, 2016, CELL REP, V17, P436, DOI 10.1016/j.celrep.2016.09.025

Serafini N, 2015, NAT REV IMMUNOL, V15, P415, DOI 10.1038/nri3855

Serriari NE, 2014, CLIN EXP IMMUNOL, V176, P266, DOI 10.1111/cei.12277

Shannon JP, 2021, IMMUNITY, V54, P276, DOI 10.1016/j.immuni.2020.12.004

Sharkey AM, 2015, J IMMUNOL, V195, P3026, DOI 10.4049/jimmunol.1501229

Sharma A, 2018, J IMMUNOL, V201, P2452, DOI 10.4049/jimmunol.1800537

Sheng JP, 2015, IMMUNITY, V43, P382, DOI 10.1016/j.immuni.2015.07.016

Sheridan BS, 2014, IMMUNITY, V40, P747, DOI 10.1016/j.immuni.2014.03.007

Shin K, 2008, J IMMUNOL, V180, P4885, DOI 10.4049/jimmunol.180.7.4885

Shiow LR, 2006, NATURE, V440, P540, DOI 10.1038/nature04606

Shook BA, 2018, SCIENCE, V362, P909, DOI 10.1126/science.aar2971

SILBERSTEIN R, 1991, BONE, V12, P227, DOI 10.1016/8756-3282(91)90068-T

Silvestre-Roig C, 2016, BLOOD, V127, P2173, DOI 10.1182/blood-2016-01-688887

Simoni Y, 2018, IMMUNOLOGY, V153, P297, DOI 10.1111/imm.12862

Simoni Y, 2017, IMMUNITY, V46, P148, DOI 10.1016/j.immuni.2016.11.005

Skon CN, 2013, NAT IMMUNOL, V14, P1285, DOI 10.1038/ni.2745

Smolen JS, 2016, LANCET, V388, P2023, DOI 10.1016/S0140-6736(16)30173-8

Sobiepanek A, 2022, INT J MOL SCI, V23, DOI 10.3390/ijms23042249

Sojka DK, 2014, SEMIN IMMUNOL, V26, P127, DOI 10.1016/j.smim.2014.01.010

Sojka DK, 2014, ELIFE, V3, DOI 10.7554/eLife.01659

Son YM, 2021, SCI IMMUNOL, V6, DOI 10.1126/sciimmunol.abb6852

Song C, 2015, J EXP MED, V212, P1869, DOI 10.1084/jem.20151403

Song XW, 2022, INT J MOL SCI, V23, DOI 10.3390/ijms232416085

SONODA T, 1983, DEV BIOL, V97, P89, DOI 10.1016/0012-1606(83)90066-0

Sontheimer RD, 2004, BEST PRACT RES CL RH, V18, P429, DOI 10.1016/j.berh.2004.03.003

Srivastava R, 2017, J VIROL, V91, DOI 10.1128/JVI.00278-17

Steel KJA, 2020, ARTHRITIS RHEUMATOL, V72, P435, DOI 10.1002/art.41156

Suárez-Fariñas M, 2011, J INVEST DERMATOL, V131, P391, DOI 10.1038/jid.2010.280

Sun HY, 2019, CANCER IMMUNOL RES, V7, P1535, DOI 10.1158/2326-6066.CIR-18-0757

Sun HY, 2019, CELL MOL IMMUNOL, V16, P205, DOI 10.1038/s41423-018-0192-y

Szabo PA, 2019, SCI IMMUNOL, V4, DOI 10.1126/sciimmunol.aas9673

Takamura S, 2016, J EXP MED, V213, P3057, DOI 10.1084/jem.20160938

Tan HX, 2022, SCI IMMUNOL, V7, DOI 10.1126/sciimmunol.abf5314

Taniguchi H, 1996, NAT MED, V2, P198, DOI 10.1038/nm0296-198

Tauber M, 2023, J EXP MED, V220, DOI 10.1084/jem.20230570

Tayade C, 2005, J LEUKOCYTE BIOL, V78, P1347, DOI 10.1189/jlb.0305142

Teijaro JR, 2011, J IMMUNOL, V187, P5510, DOI 10.4049/jimmunol.1102243

Tessmer MS, 2011, PLOS PATHOG, V7, DOI 10.1371/journal.ppat.1001254

Theoharides TC, 2015, NEW ENGL J MED, V373, P163, DOI 10.1056/NEJMra1409760

Thomas SY, 2011, J EXP MED, V208, P1179, DOI 10.1084/jem.20102630

Thomas SK, 2023, NAT COMMUN, V14, DOI 10.1038/s41467-023-41771-z

Tian J, 2023, INFLAMM RES, V72, P363, DOI 10.1007/s00011-022-01677-w

Tieu R, 2023, SCI IMMUNOL, V8, DOI 10.1126/sciimmunol.add8454

Tohyama M, 2018, EUR J IMMUNOL, V48, P168, DOI 10.1002/eji.201747017

Traina F, 2012, PLOS ONE, V7, DOI 10.1371/journal.pone.0043090

Treiner E, 2003, NATURE, V422, P164, DOI 10.1038/nature01433

TSAI M, 1991, J EXP MED, V174, P125, DOI 10.1084/jem.174.1.125

Turner DL, 2014, MUCOSAL IMMUNOL, V7, P501, DOI 10.1038/mi.2013.67

Udalova IA, 2016, NAT REV RHEUMATOL, V12, P472, DOI 10.1038/nrrheum.2016.91

Uderhardt S, 2019, CELL, V177, P541, DOI 10.1016/j.cell.2019.02.028

Ural BB, 2020, SCI IMMUNOL, V5, DOI 10.1126/sciimmunol.aax8756

Ushio A, 2018, FRONT IMMUNOL, V9, DOI 10.3389/fimmu.2018.02594

Uyar O, 2020, J VIROL, V94, DOI 10.1128/JVI.01428-20

Valent P, 2020, THERANOSTICS, V10, P10743, DOI 10.7150/thno.46719

van de Laar L, 2016, IMMUNITY, V44, P755, DOI 10.1016/j.immuni.2016.02.017

van Gisbergen KPJM, 2012, NAT IMMUNOL, V13, P864, DOI 10.1038/ni.2393

VanHorn S, 2021, DEV CELL, V56, P7, DOI 10.1016/j.devcel.2020.10.021

Vargas-Uricoechea H, 2023, CELLS-BASEL, V12, DOI 10.3390/cells12060918

Veis DJ, 2023, ANNU REV PATHOL-MECH, V18, P257, DOI 10.1146/annurev-pathmechdis-031521-040919

Verma AK, 2020, J IMMUNOL, V205, P1601, DOI 10.4049/jimmunol.2000094

Vesely MD, 2011, ANNU REV IMMUNOL, V29, P235, DOI 10.1146/annurev-immunol-031210-101324

Vincenti I, 2022, SCI TRANSL MED, V14, DOI 10.1126/scitranslmed.abl6058

Viola MF, 2023, NATURE, V618, P818, DOI 10.1038/s41586-023-06200-7

Viola MF, 2021, GUT, V70, P1383, DOI 10.1136/gutjnl-2020-323121

Virassamy B, 2023, CANCER CELL, V41, P585, DOI 10.1016/j.ccell.2023.01.004

Vivier E, 2018, CELL, V174, P1054, DOI 10.1016/j.cell.2018.07.017

Vliagoftis H, 2005, IMMUNOL REV, V206, P190, DOI 10.1111/j.0105-2896.2005.00279.x

Wakim LM, 2010, P NATL ACAD SCI USA, V107, P17872, DOI 10.1073/pnas.1010201107

Wang WB, 2023, NAT IMMUNOL, V24, P225, DOI 10.1038/s41590-022-01399-5

Wang ZS, 2023, CELL, V186, P4454, DOI 10.1016/j.cell.2023.08.019

Wang ZN, 2023, J NANOBIOTECHNOL, V21, DOI 10.1186/s12951-023-02229-y

Wanga YL, 2019, CRIT REV MICROBIOL, V45, P581, DOI 10.1080/1040841X.2019.1660615

WATANABE N, 1994, PARASITE IMMUNOL, V16, P137, DOI 10.1111/j.1365-3024.1994.tb00333.x

Watanabe R, 2015, SCI TRANSL MED, V7, DOI 10.1126/scitranslmed.3010302

Webb JR, 2015, CANCER IMMUNOL RES, V3, P926, DOI 10.1158/2326-6066.CIR-14-0239

Wei DG, 2005, J EXP MED, V202, P239, DOI 10.1084/jem.20050413

Weisberg SP, 2019, CELL REP, V29, P3916, DOI 10.1016/j.celrep.2019.11.056
[truncated: 10,081,385 more chars]
